# Tandem Palladium/Copper-Catalyzed Decarboxylative Approach to Benzoimidazo- and Imidazophenanthridine Skeletons

**DOI:** 10.1021/acs.orglett.2c03647

**Published:** 2022-12-13

**Authors:** Xin Geng, Andrey Shatskiy, Gregory R. Alvey, Jian-Quan Liu, Markus D. Kärkäs, Xiang-Shan Wang

**Affiliations:** † School of Chemistry and Materials Science, Jiangsu Key Laboratory of Green Synthesis for Functional Materials, Jiangsu Normal University, Xuzhou, Jiangsu 221116, China; ‡ Department of Chemistry, KTH Royal Institute of Technology, SE-100 44 Stockholm, Sweden

## Abstract

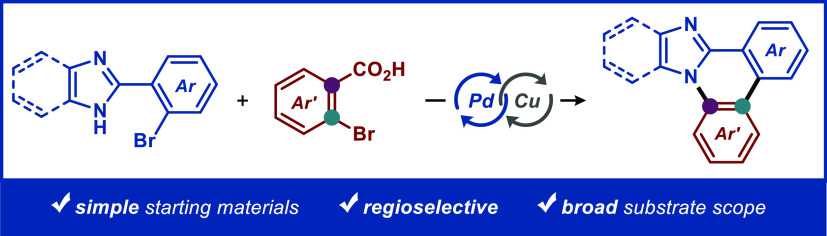

A protocol for a tandem Pd/Cu-catalyzed intermolecular cross-coupling cascade between *o*-bromobenzoic acids and 2-(2-bromoaryl)-1*H*-benzo[*d*]imidazoles or the corresponding imidazoles is presented. The protocol provides conceptually novel and controlled access to synthetically useful *N*-fused (benzo)imidazophenanthridine scaffolds with high efficiency, a broad substrate scope, and excellent functional group compatibility.

Benzoimidazo- and imidazophenanthridines represent an important class of *N*-heterocycles that display highly attractive biological, photophysical, and aggregation properties (Figure 1, top). Several (dihydro)imidazophenanthridine derivatives were identified as potent DNA-binding agents with anticancer activities.^1^ A naturally occurring alkaloid featuring an imidazophenanthridine core, namely, zephycandidine A, displayed considerable antitumor and antiacetylcholinesterase activities,^2^ prompting the synthesis and biological studies of a range of related compounds.^3^ Furthermore, several (benzo)imidazophenanthridines with extended π-systems have been applied as components of organic light-emitting diodes (OLEDs),^4^ while (dihydro)imidazophenanthridinium salts of polyoxometalates (POMs) were found to undergo highly unusual spontaneous self-assembly into microtubular structures.^5^

**Figure 1:**
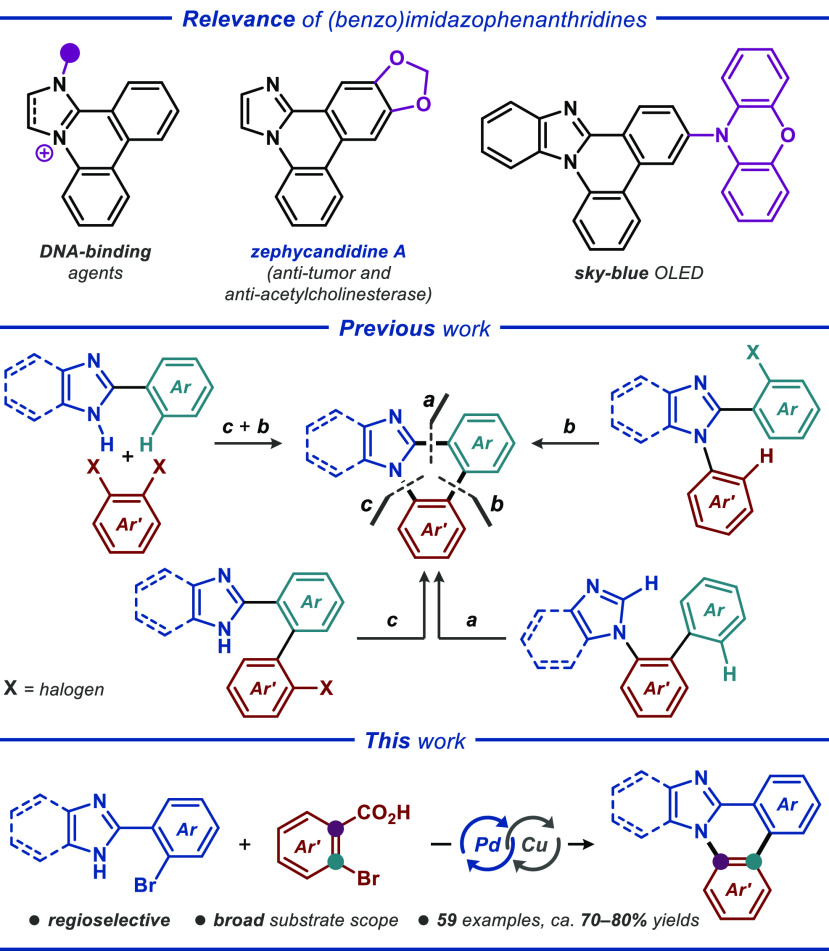
Relevance and synthesis of (benzo)imidazophenanthridine derivatives.

To date, a number of synthetic methodologies to access such heterocyclic manifolds have been disclosed (Figure 1, middle). Palladium-catalyzed cross-coupling reactions are predominant among these methods and allow the assembly of the (benzo)imidazophenanthridine core through various bond-disconnection strategies.^6^ Additionally, several photo-^7^ and electrochemical methods^8^ have been utilized to access the same type of heterocycles. Despite these developments, affording (benzo)imidazophenanthridine derivatives in a controlled and regioselective manner remains a notable synthetic challenge. Considering our long-standing interest in the development of metal-catalyzed annulation reactions,^9^ we sought to provide a straightforward and modular synthetic approach to substituted (benzo)imidazophenanthridines from simple starting materials and under mild reaction conditions.

Initially, readily prepared 2-(2-bromophenyl)-1*H*-benzo[*d*]imidazole (**1a**) and *o*-bromobenzoic acid (**2a**) were selected as model substrates for the optimization of the reaction conditions (Scheme 1, top). Several reaction parameters were surveyed, including the palladium precursor, the auxiliary catalyst, the base, the ligand, the temperature, and the solvent. A range of palladium precursors, such as Pd(OAc)_2_, PdCl_2_ and (η^3^-C_3_H_5_)_2_Pd_2_Cl_2_, and auxiliary copper catalysts, such as CuI, CuBr, Cu(OAc)_2_, and Cu(OTf)_2_, were evaluated in DMF at 80 °C (Table S3, entries 1–7, respectively). As a result, a combination of Pd(OAc)_2_ and a CuI cocatalyst displayed the best reactivity, affording the benzoimidazophenanthridine product **3a** in 35% yield (Scheme 1, entry 1), while copper(II) precursors were found less effective. The addition of commonly employed ligands, such as Ph_3_P, 1,10-phenanthroline (*o*-phen) and l-proline, had a significant influence on the reaction. Specifically, the addition of Ph_3_P increased the yield of the desired product **3a** to 51% (Scheme 1, entry 2), while *o*-phen and l-proline proved less effective (Table S3, entries 9 and 10, respectively). Screening of inorganic bases (Table S3, entries 8, 11, and 12) revealed Cs_2_CO_3_ as the optimal base. Subsequently, a survey of solvents (Table S3, entries 13–15, respectively), including DMSO, DMAc, and toluene, demonstrated their negative effect on the reaction, providing **3a** in diminished yields (12–41%). Conducting the reaction at different temperatures had a dramatic effect on the reaction outcome (Table S3, entries 8 and 16–19). Therefore, the conditions from entry 3 (Scheme 1, entry 17 from Table S3) were selected as optimal for further investigation of the disclosed transformation. Control experiments in the absence of either Pd(OAc)_2_ or CuI catalysts were conducted (Scheme 1, entries 4 and 5), demonstrating that both the catalysts were required for the efficient formation of product **3a**. A potential role of the Cu^I^ catalyst as a reductant for transforming the Pd^II^ precursor into an active Pd^0^ form was deemed unlikely, as the reaction with the Pd(PPh_3_)_4_ catalyst in the absence of the Cu catalyst furnished the desired product **3a** in a mere 19% yield (Scheme 1, entry 6). Addition of CuI to the latter reaction replenished the tandem catalytic activity, delivering product **3a** in 69% yield (Scheme 1, entry 7).

**Scheme 1:**
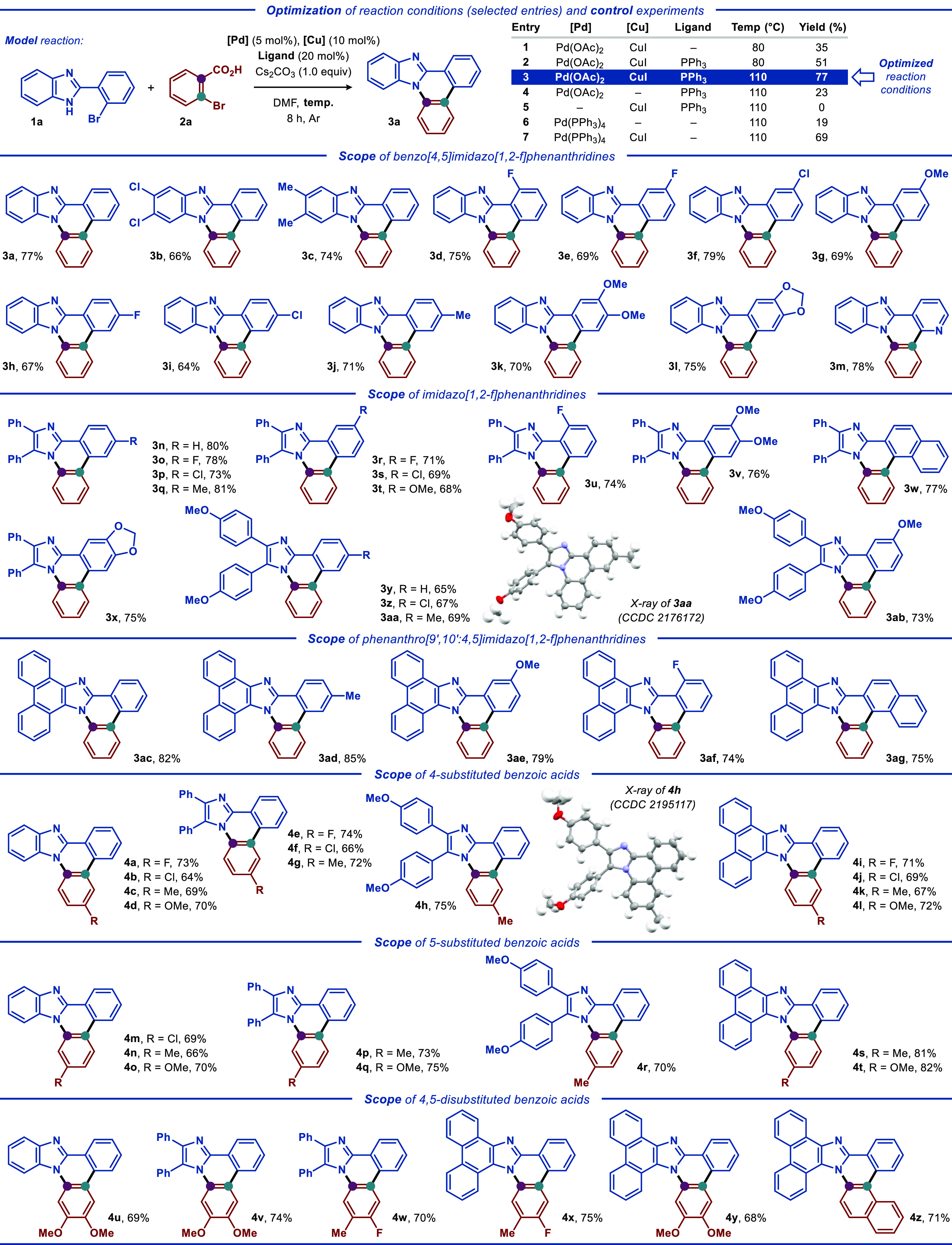
Optimization of Reaction Conditions and Reaction Scope

After the optimal reaction conditions were established, the applicability and scope of the disclosed protocol was investigated (Scheme 1). A diverse range of functionalized 2-(2-bromoaryl)-1*H*-imidazoles and the corresponding benzimidazoles **1** smoothly reacted with *ortho*-bromobenzoic acid **2a**, delivering the respective (benzo)imidazophenanthridine products **3** in generally good yields (Scheme 1). For example, 2-(2-bromophenyl)-1*H*-benzo[*d*]imidazoles **1** featuring electron-donating and electron-withdrawing substituents at the benzimidazole moiety, such as Cl and Me, were compatible with the developed protocol, affording the expected products **3b** and **3c** in 66% and 74% yields, respectively. The 2-(2-bromophenyl)-1*H*-benzo[*d*]imidazole substrates with different substituents on the benzene ring, such as F, Cl, Me, and MeO, were also efficiently converted to the desired products **3d**–**3l** in good yields (64–79%). Utilization of the pyridyl-containing substrate **1m** provided the expected product **3m** in 78% yield. Next, the broader generality of the developed protocol was demonstrated using several functionalized 2-(2-bromophenyl)- 1*H*-imidazoles as substrates, all of which smoothly reacted with **2a** and provided access to a range of *N*-fused heterocyclic scaffolds containing 2,3-diphenylimidazo (**3n**–**3ab**) and phenanthro[9′,10′:4,5]imidazo (**3ac**–**3ag**) heteroaromatic cores in generally high yields (65–85%).

Subsequently, a range of substituted *ortho*-bromobenzoic acids **2** was investigated under the optimal reaction conditions (Scheme 1). Gratifyingly, 4- and 5-substituted *ortho*-bromobenzoic acids **2** bearing electron-withdrawing (F and Cl) or electron-donating substituents (Me and MeO) smoothly reacted with substituted benzimidazole-, imidazole-, and 1*H*-phenanthro[9,10-*d*]imidazole-based substrates **1**, furnishing the desired products **4a**–**4h** in generally high yields (64–82%). Furthermore, 4,5-disubstituted *ortho*-bromobenzoic acids, as well as 1-bromo-2-naphthoic acid, were compatible with the disclosed protocol, affording the expected products **4u**–**4z** in up to 75% yields. At the same time, 1-bromo-2-naphthoic acids with electron-withdrawing substituents failed to produce the desired product. Similarly, both 3- and 6-methyl-substituted *o*-bromobenzoic acids proved inefficient and produced inseparable mixtures of products. The structures of products **3aa** and **4h** were unequivocally confirmed by single-crystal X-ray analysis (CCDC 2176172 and CCDC 2195117, respectively; see the Supporting Information for details). Notably, the substitution pattern in products **4a**–**4t** and **4w**–**4x** firmly confirms that in the disclosed transformation the C–N and C–C bonds are formed through decarboxylative and dehalogenative cross-coupling reactions, respectively.

Besides the control experiments presented in Table S3, entries 20–23, a series of additional control reactions were conducted to gain insight into the operational mechanism(s) of the disclosed transformation. Subjecting *ortho*-bromobenzoic acid **2a** to the optimized reaction conditions in the absence of **1** afforded triphenylene **5a** in 81% yield (Scheme 2, top left). The formation of such a trimerization product could indicate the involvement of benzyne as the key intermediate in both the trimerization and intermolecular cross-coupling reactions, as has been proposed for several transformations featuring similar starting materials under related conditions.^10^ However, conducting the reaction with 4- or 5-substituted *ortho*-bromobenzoic acids delivered trisubstituted triphenylene derivatives **5b** and **5c**, respectively, displaying formal C_3h_ symmetry, while the metal-catalyzed [2 + 2 + 2] cycloaddition of substituted benzynes typically would produce triphenylenes as a mixture of products with C_3h_ and C_s_ symmetries, favoring the latter.^10,11^ This observation indirectly indicates that the disclosed trimerization proceeds through three consecutive decarboxylative cross-coupling steps rather than a [2 + 2 + 2] cycloaddition. Furthermore, conducting the trimerization reaction in the absence of the Pd or Cu catalyst either prohibited the reaction (in the absence of Pd) or greatly suppressed the formation of product **5a** (17% yield in the absence of Cu), suggesting that the reaction firmly relies on tandem Pd/Cu catalysis. Next, we investigated if the C–C and C–N bonds in product **3a** could be formed independently during the tandem Pd/Cu-catalyzed reaction using benzoic acid (unsubstituted and *ortho*-chloro or bromo-substituted) and bromobenzene as the coupling partners together with bromo-substituted benzimidazole **1a** (Scheme 2, top right). As a result, no reaction was observed between **1a** and either the benzoic acids or bromobenzene under the standard reaction conditions, highlighting the crucial role of tandem Pd/Cu catalysis for both the bond-forming steps. The reactions in the absence of either Pd or Cu catalysts failed to produce any cross-coupling products and only delivered product **6a** in the absence of the Pd catalyst, albeit in a low yield (37%).

**Scheme 2:**
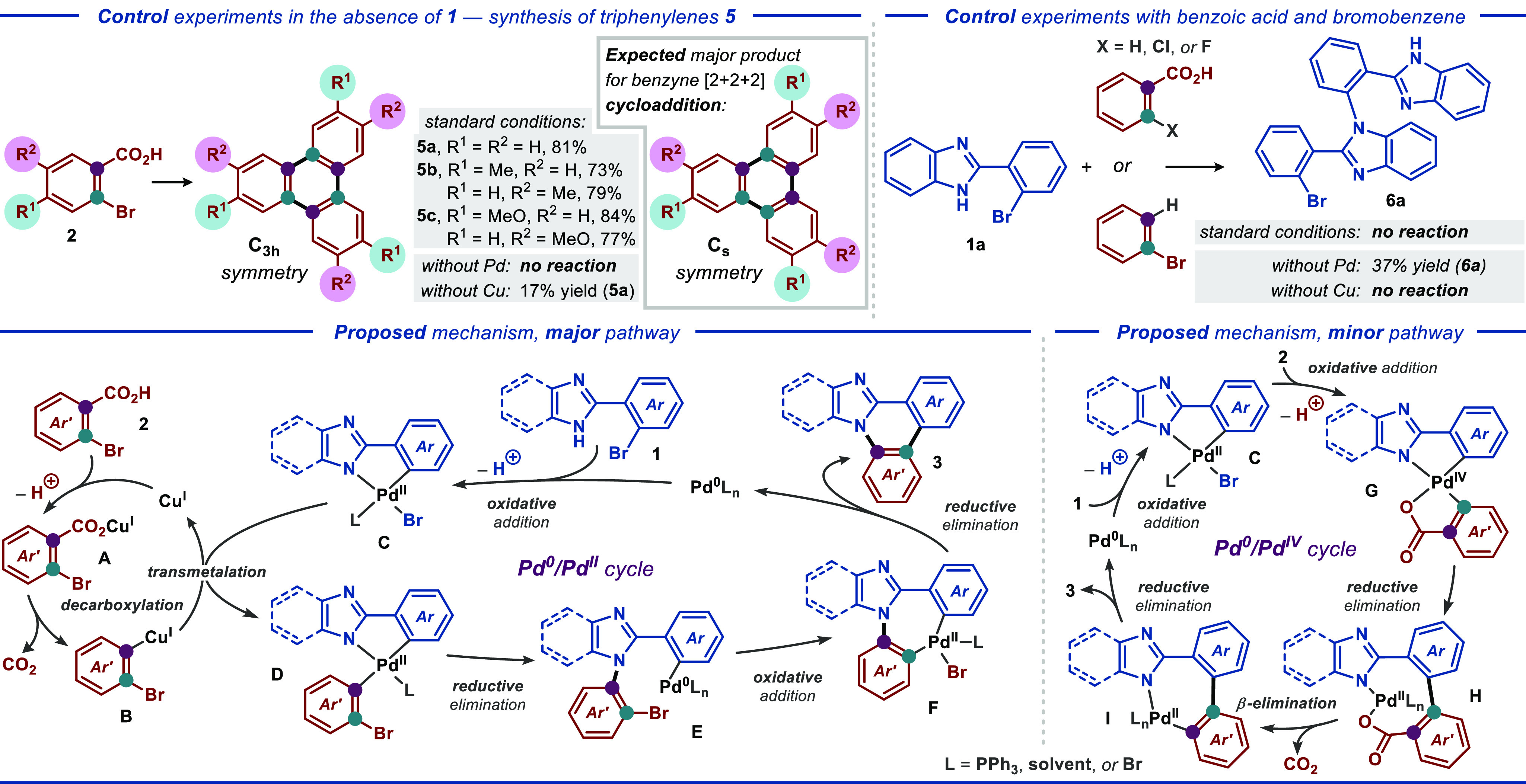
Control Experiments and Proposed Mechanistic Pathways

Based on the results of the control experiments and relevant literature precedents (vide infra), we propose plausible major and minor mechanistic pathways for the disclosed cross-coupling between *ortho*-bromobenzoic acids (**2**) and bromo-substituted (benzo)imidazoles (**1**) (Scheme 2, bottom). In the major mechanistic pathway, the Cu^I^ catalyst and *ortho*-bromobenzoic acid **2** form a carboxylate salt **A**, which eliminates CO_2_ to afford the organocuprate species **B**. In parallel, the Pd^0^ catalyst (formed *in situ*) undergoes oxidative addition to bromo-substituted (benzo)imidazole **1** to furnish the Pd^II^ metallacycle **C**. In the key step of the reaction, transmetalation between species **B** and **C** concludes the copper-mediated catalytic cycle and produces the highly electron-rich Pd^II^ species **D**. This species is then responsible for the formation of the C–N bond of the final product through reductive elimination, concomitantly producing Pd^0^ species **E**. The latter undergoes facile intramolecular oxidative addition with the C–Br bond to form the Pd^II^ species **F**. Finally, C–C bond formation takes place through reductive elimination in intermediate **F**, concluding the Pd^0^/Pd^II^ catalytic cycle, and furnishes the desired product **3**. An alternative minor mechanistic pathway enables the disclosed transformation without the involvement of Cu catalysis and becomes the major pathway under the conditions from Table S3, entries 20 and 22 (ca. 20% yield of **3a**). Here, the Pd^0^ catalyst undergoes two consecutive oxidative addition steps, first to substrate **1**, furnishing Pd^II^ intermediate **C**, and then to substrate **2**, affording Pd^IV^ intermediate **G**. The latter intermediate mediates the formation of the C–C bond through reductive elimination and provides Pd^II^ intermediate **H**. Subsequently, CO_2_ is released through β-elimination to produce Pd^II^ metallacycle **I**. Finally, reductive elimination in the latter intermediate mediates the formation of the C–N bond, concluding the Pd^0^/Pd^IV^ catalytic cycle and furnishing the desired product **3**. A number of literature precedents support the outlined tandem Pd/Cu catalytic sequence featuring a transmetalation step. Most notably, Gooßen previously demonstrated several decarboxylative cross-coupling reactions that proceed through Cu-catalyzed decarboxylation of benzoic acids, followed by transmetalation to Pd^II^ species with subsequent C–C or C–N bond-forming reactions.^12^ Considering the minor mechanistic pathway, the formation of species related to Pd^IV^ intermediate **G** and its consecutive reductive elimination and decarboxylation was demonstrated previously.^13^ The key regioselectivity-determining step in the major pathway, that is, reductive elimination in intermediate **D**, represents a highly unusual example of the preferential formation of a C–N rather than C–C bond in Pd species featuring two phenyl ligands and one amide ligand.^14^ Here, the observed selectivity can be potentially attributed to the oxidation state of the palladium center, Pd^II^, as the opposite selectivity was observed for reductive elimination from Pd^IV^ species.

In conclusion, we have developed a novel Pd-catalyzed cascade annulation reaction of bromo-substituted benzimidazoles and *o*-bromobenzoic acids, providing a convenient and modular approach to a range of functionalized (benzo)imidazophenanthridines. A mechanism based on a tandem Pd/Cu decarboxylative cross-coupling pathway is proposed. Considering the practicality of this method and the importance of (benzo)imidazophenanthridine scaffolds in materials science and medicinal chemistry, the methodology described here will undoubtedly find wide applications in future synthetic endeavors.

## Data Availability

The data underlying this study are available in the published article and its online Supporting Information.

## Supporting Information Available

Experimental procedures, characterization data, and copies of NMR spectra for all obtained products (PDF)
FAIR data, including the primary NMR FID files, for compounds **3a**–**z**, **3aa**–**3ag**, **4a**–**z**, **5a**–**c**, and **6a** (ZIP)
