# Supplementary material for: Tandem Palladium/Copper-Catalyzed Decarboxylative Approach to Benzoimidazo- and Imidazophenanthridine Skeletons
Source: Org Lett. 2022 Dec 13;24(50):9194–9. doi: 10.1021/acs.orglett.2c03647 (PMC9791681; doi:10.1021/acs.orglett.2c03647)
Supplement: Supplementary file 1 — ol2c03647_si_001.pdf [file ol2c03647_si_001.pdf]

Supporting Information

Tandem Palladium/Copper-Catalyzed Decarboxylative Approach to Benzoimidazo- and Imidazophenanthridine Skeletons

Xin Geng,† Andrey Shatskiy,‡ Gregory R. Alvey,‡ Jian-Quan Liu,†,‡,* Markus D. Kärkäs,‡,* and Xiang-Shan Wang†,*

† School of Chemistry and Materials Science, Jiangsu Normal University, Xuzhou, Jiangsu 221116, China

‡ Department of Chemistry, KTH Royal Institute of Technology, SE-100 44 Stockholm, Sweden

* E-mail: liujq316@jsnu.edu.cn (J.-Q.L.); karkas@kth.se (M.D.K.); xswang@jsnu.edu.cn (X.-S.W)

Table of Contents

I. General information S1

II. Crystallography S1

III. Optimization of reaction conditions S4

IV. Synthesis and analytical data of compounds 1 and 3 ..... S5

Benzo[4,5]imidazo[1,2-*f*]phenanthridine (**3a**) S6

1 mmol scale synthesis of benzo[4,5]imidazo[1,2-*f*]phenanthridine (**3a**) S6

11,12-Dichlorobenzo[4,5]imidazo[1,2-*f*]phenanthridine (**3b**) S7

11,12-Dimethylbenzo[4,5]imidazo[1,2-*f*]phenanthridine (**3c**) S8

8-Fluorobenzo[4,5]imidazo[1,2-*f*]phenanthridine (**3d**) S8

7-Fluorobenzo[4,5]imidazo[1,2-*f*]phenanthridine (**3e**) S9

7-Chlorobenzo[4,5]imidazo[1,2-*f*]phenanthridine (**3f**) S10

7-Methoxybenzo[4,5]imidazo[1,2-*f*]phenanthridine (**3g**) S10

6-Fluorobenzo[4,5]imidazo[1,2-*f*]phenanthridine (**3h**) S11

6-Chlorobenzo[4,5]imidazo[1,2-*f*]phenanthridine (**3i**) S12

6-Methylbenzo[4,5]imidazo[1,2-*f*]phenanthridine (**3j**) S12

6,7-Dimethoxybenzo[4,5]imidazo[1,2-*f*]phenanthridine (**3k**) S13

Benzo[4,5]imidazo[1,2-*f*][1,3]dioxolo[4,5-*j*]phenanthridine (**3l**) S14

Benzo[*h*]benzo[4,5]imidazo[2,1-*f*][1,6]naphthyridine (**3m**) S14

2,3-Diphenylimidazo[1,2-*f*]phenanthridine (**3n**) S15

10-Fluoro-2,3-diphenylimidazo[1,2-*f*]phenanthridine (**3o**) S16

10-Chloro-2,3-diphenylimidazo[1,2-*f*]phenanthridine (**3p**) S16

10-Methyl-2,3-diphenylimidazo[1,2-*f*]phenanthridine (**3q**) S17

11-Fluoro-2,3-diphenylimidazo[1,2-*f*]phenanthridine (**3r**) S18

11-Chloro-2,3-diphenylimidazo[1,2-*f*]phenanthridine (**3s**) S18

11-Methoxy-2,3-diphenylimidazo[1,2-*f*]phenanthridine (**3t**) S19

12-Fluoro-2,3-diphenylimidazo[1,2-*f*]phenanthridine (**3u**) S20

10,11-Dimethoxy-2,3-diphenylimidazo[1,2-f]phenanthridine (**3v**) S20

1,2-Diphenylbenzo[*k*]imidazo[1,2-*f*]phenanthridine (**3w**) S21

2,3-Diphenyl-[1,3]dioxolo[4,5-*j*]imidazo[1,2-*f*]phenanthridine (**3x**) S22

2,3-Bis(4-methoxyphenyl)imidazo[1,2-*f*]phenanthridine (**3y**) S22

10-Chloro-2,3-bis(4-methoxyphenyl)imidazo[1,2-*f*]phenanthridine (**3z**).... S23

2,3-Bis(4-methoxyphenyl)-10-methylimidazo[1,2-*f*]phenanthridine (**3aa**).... S24

11-Methoxy-2,3-bis(4-methoxyphenyl)imidazo[1,2-*f*]phenanthridine (**3ab**) S25

Phenanthro[9',10':4,5]imidazo[1,2-*f*]phenanthridine (**3ac**) S25

6-Methylphenanthro[9',10':4,5]imidazo[1,2-*f*]phenanthridine (**3ad**).... S26

7-Methoxyphenanthro[9',10':4,5]imidazo[1,2-*f*]phenanthridine (**3ae**) S27

8-Fluorophenanthro[9',10':4,5]imidazo[1,2-*f*]phenanthridine (**3af**).... S28

Benzo[*k*]phenanthro[9',10':4,5]imidazo[1,2-*f*]phenanthridine (**3ag**).... S28

3-Fluorobenzo[4,5]imidazo[1,2-*f*]phenanthridine (**4a**) S29

3-Chlorobenzo[4,5]imidazo[1,2-*f*]phenanthridine (**4b**).... S30

3-Methylbenzo[4,5]imidazo[1,2-*f*]phenanthridine (**4c**) S30

3-Methoxybenzo[4,5]imidazo[1,2-*f*]phenanthridine (**4d**) S31

7-Fluoro-2,3-diphenylimidazo[1,2-*f*]phenanthridine (**4e**) S32

7-Chloro-2,3-diphenylimidazo[1,2-*f*]phenanthridine (**4f**) S32

7-Methyl-2,3-diphenylimidazo[1,2-*f*]phenanthridine (**4g**) S33

2,3-Bis(4-methoxyphenyl)-7-methylimidazo[1,2-*f*]phenanthridine (**4h**) S34

3-Fluorophenanthro[9',10':4,5]imidazo[1,2-*f*]phenanthridine (**4i**) S35

3-Methylphenanthro[9',10':4,5]imidazo[1,2-*f*]phenanthridine (**4j**) S36

3-Methylphenanthro[9',10':4,5]imidazo[1,2-*f*]phenanthridine (**4k**) S36

3-Methoxyphenanthro[9',10':4,5]imidazo[1,2-*f*]phenanthridine (**4l**) S37

2-Chlorobenzo[4,5]imidazo[1,2-*f*]phenanthridine (**4m**).... S38

2-Methylbenzo[4,5]imidazo[1,2-*f*]phenanthridine (**4n**) S38

2-Methoxybenzo[4,5]imidazo[1,2-*f*]phenanthridine (**4o**) S39

6-Methyl-2,3-diphenylimidazo[1,2-*f*]phenanthridine (**4p**).... S40

6-Methoxy-2,3-diphenylimidazo[1,2-*f*]phenanthridine (**4q**) S40

2,3-Bis(4-methoxyphenyl)-6-methylimidazo[1,2-*f*]phenanthridine (**4r**) S41

2-Methylphenanthro[9',10':4,5]imidazo[1,2-*f*]phenanthridine (**4s**) S42

2-Methoxyphenanthro[9',10':4,5]imidazo[1,2-*f*]phenanthridine (**4t**).... S43

2,3-Dimethoxybenzo[4,5]imidazo[1,2-*f*]phenanthridine (**4u**) S43

6,7-Dimethoxy-2,3-diphenylimidazo[1,2-*f*]phenanthridine (**4v**) S44

7-Fluoro-6-methyl-2,3-diphenylimidazo[1,2-*f*]phenanthridine (**4w**) S45

3-Fluoro-2-methylphenanthro[9',10':4,5]imidazo[1,2-*f*]phenanthridine (**4x**) S45

2,3-Dimethoxyphenanthro[9',10':4,5]imidazo[1,2-*f*]phenanthridine (**4y**) S46

Benzo[*a*]phenanthro[9',10':4,5]imidazo[1,2-*f*]phenanthridine (**4z**) S47

Triphenylene (**5a**) S48

2,6,10-Trimethyltriphenylene (**5b**) S48

2,6,10-Trimethoxytriphenylene (**5c**) S49

1-(2-(1*H*-Benzo[*d*]imidazol-2-yl)phenyl)-2-(2-bromophenyl)-1*H*-benzo[d]imidazole (**6a**) S50

V. NMR spectra S51

Benzo[4,5]imidazo[1,2-*f*]phenanthridine (**3a**) S51

11,12-Dichlorobenzo[4,5]imidazo[1,2-*f*]phenanthridine (**3b**) S52

11,12-Dimethylbenzo[4,5]imidazo[1,2-*f*]phenanthridine (**3c**) S53

8-Fluorobenzo[4,5]imidazo[1,2-*f*]phenanthridine (**3d**) S54

7-Fluorobenzo[4,5]imidazo[1,2-*f*]phenanthridine (**3e**) S56

7-Chlorobenzo[4,5]imidazo[1,2-*f*]phenanthridine (**3f**) S58

7-Methoxybenzo[4,5]imidazo[1,2-*f*]phenanthridine (**3g**) S59

6-Fluorobenzo[4,5]imidazo[1,2-*f*]phenanthridine (**3h**) S60

6-Chlorobenzo[4,5]imidazo[1,2-*f*]phenanthridine (**3i**) S62

6-Methylbenzo[4,5]imidazo[1,2-*f*]phenanthridine (**3j**) S63

6,7-Dimethoxybenzo[4,5]imidazo[1,2-*f*]phenanthridine (**3k**) S64

Benzo[4,5]imidazo[1,2-*f*][1,3]dioxolo[4,5-*j*]phenanthridine (**3l**) S65

Benzo[*h*]benzo[4,5]imidazo[2,1-*f*][1,6]naphthyridine (**3m**) S66

2,3-Diphenylimidazo[1,2-*f*]phenanthridine (**3n**) S67

10-Fluoro-2,3-diphenylimidazo[1,2-*f*]phenanthridine (**3o**) S68

10-Chloro-2,3-diphenylimidazo[1,2-*f*]phenanthridine (**3p**) S70

10-Methyl-2,3-diphenylimidazo[1,2-*f*]phenanthridine (**3q**) S71

11-Fluoro-2,3-diphenylimidazo[1,2-*f*]phenanthridine (**3r**) S72

11-Chloro-2,3-diphenylimidazo[1,2-*f*]phenanthridine (**3s**) S74

11-Methoxy-2,3-diphenylimidazo[1,2-*f*]phenanthridine (**3t**) S75

12-Fluoro-2,3-diphenylimidazo[1,2-*f*]phenanthridine (**3u**) S76

10,11-Dimethoxy-2,3-diphenylimidazo[1,2-*f*]phenanthridine (**3v**) S78

1,2-Diphenylbenzo[k]imidazo[1,2-*f*]phenanthridine (**3w**) S79

2,3-Diphenyl-[1,3]dioxolo[4,5-j]imidazo[1,2-*f*]phenanthridine (**3x**) S80

2,3-Bis(4-methoxyphenyl)imidazo[1,2-*f*]phenanthridine (**3y**) S81

10-Chloro-2,3-bis(4-methoxyphenyl)imidazo[1,2-*f*]phenanthridine (**3z**) S82

2,3-Bis(4-methoxyphenyl)-10-methylimidazo[1,2-*f*]phenanthridine (**3aa**) S83

11-Methoxy-2,3-bis(4-methoxyphenyl)imidazo[1,2-*f*]phenanthridine (**3ab**) S84

Phenanthro[9',10':4,5]imidazo[1,2-*f*]phenanthridine (**3ac**) S85

6-Methylphenanthro[9',10':4,5]imidazo[1,2-*f*]phenanthridine (**3ad**) S86

7-Methoxyphenanthro[9',10':4,5]imidazo[1,2-*f*]phenanthridine (**3ae**) S87

8-Fluorophenanthro[9',10':4,5]imidazo[1,2-*f*]phenanthridine (**3af**) S88

Benzo[*k*]phenanthro[9',10':4,5]imidazo[1,2-*f*]phenanthridine (**3ag**) S90

3-Fluorobenzo[4,5]imidazo[1,2-*f*]phenanthridine (**4a**) S91

3-Chlorobenzo[4,5]imidazo[1,2-*f*]phenanthridine (**4b**) S93

3-Methylbenzo[4,5]imidazo[1,2-*f*]phenanthridine (**4c**) S94

3-Methoxybenzo[4,5]imidazo[1,2-*f*]phenanthridine (**4d**) S95

7-Fluoro-2,3-diphenylimidazo[1,2-*f*]phenanthridine (**4e**) S96

7-Chloro-2,3-diphenylimidazo[1,2-*f*]phenanthridine (**4f**) S98

7-Methyl-2,3-diphenylimidazo[1,2-*f*]phenanthridine (**4g**) S99

2,3-Bis(4-methoxyphenyl)-7-methylimidazo[1,2-*f*]phenanthridine (**4h**) S100

3-Fluorophenanthro[9',10':4,5]imidazo[1,2-*f*]phenanthridine (**4i**) S101

3-Methylphenanthro[9',10':4,5]imidazo[1,2-*f*]phenanthridine (**4j**) S103

3-Methylphenanthro[9',10':4,5]imidazo[1,2-*f*]phenanthridine (**4k**) S104

3-Methoxyphenanthro[9',10':4,5]imidazo[1,2-*f*]phenanthridine (**4l**) S105

2-Chlorobenzo[4,5]imidazo[1,2-*f*]phenanthridine (**4m**) S106

2-Methylbenzo[4,5]imidazo[1,2-*f*]phenanthridine (**4n**) S107

2-Methoxybenzo[4,5]imidazo[1,2-*f*]phenanthridine (**4o**) S108

6-Methyl-2,3-diphenylimidazo[1,2-*f*]phenanthridine (**4p**) S109

6-Methoxy-2,3-diphenylimidazo[1,2-*f*]phenanthridine (**4q**) S110

2,3-Bis(4-methoxyphenyl)-6-methylimidazo[1,2-*f*]phenanthridine (**4r**) S111

2-Methylphenanthro[9',10':4,5]imidazo[1,2-*f*]phenanthridine (**4s**) S112

2-Methoxyphenanthro[9',10':4,5]imidazo[1,2-*f*]phenanthridine (**4t**)..................................... S113

2,3-Dimethoxybenzo[4,5]imidazo[1,2-*f*]phenanthridine (**4u**)... S114

6,7-Dimethoxy-2,3-diphenylimidazo[1,2-*f*]phenanthridine (**4v**)... S115

7-Fluoro-6-methyl-2,3-diphenylimidazo[1,2-*f*]phenanthridine (**4w**) S116

3-Fluoro-2-methylphenanthro[9',10':4,5]imidazo[1,2-*f*]phenanthridine (**4x**) S118

2,3-Dimethoxyphenanthro[9',10':4,5]imidazo[1,2-*f*]phenanthridine (**4y**)............................... S120

Benzo[*a*]benzo[4,5]imidazo[1,2-*f*]phenanthridine (**4z**)... S121

Triphenylene (**5a**)... S122

2,6,10-Trimethyltriphenylene (**5b**) S123

2,6,10-Trimethoxytriphenylene (**5c**)... S124

1-(2-(1*H*-Benzo[*d*]imidazol-2-yl)phenyl)-2-(2-bromophenyl)-1*H*-benzo[*d*]imidazole (**6a**)........ S125

VI. References.. S126

I. General information

All reagents were purchased from commercial sources and used without treatment unless otherwise indicated. The products were purified by column chromatography over silica gel. ^{1}H NMR and ^{13}C NMR spectra were recorded at 25 °C on a Varian spectrometer at 400 MHz and 101 MHz, respectively, with TMS as the internal standard. Mass spectra were recorded on a BRUKER AutoflexIII Smartbeam MS-spectrometer. High resolution mass spectra (HRMS) were recorded on Bruck microTof using ESI-TOF method.

II. Crystallography

Compound **4h** (50 mg) was dissolved in a centrifuge tube in 150 μL $CDCl_3$. Upon standing for several days (seven days), crystals suitable for X-ray diffraction of **3aa** and **4h** were obtained. The structures of the *N*-fused heterocyclic scaffolds were further established by X-ray diffraction. Single-crystal X-ray diffraction data for the reported compound was recorded at a temperature of 296(2) K on an Oxford Diffraction Gemini R Ultra diffractometer using a ω scan technique with Mo-Kα radiation (λ = 0.71073 Å). The structures were solved by the Direct Method of SHELXS-97 and refined by full-matrix least-squares techniques using the SHELXL-97 program.[1] Non-hydrogen atoms were refined with anisotropic temperature parameters, and hydrogen atoms of the ligands were refined as rigid groups. Basic information pertaining to crystal parameters and structure refinement is summarized in Tables S1 and S2.

Table S1. Crystal structure and refinement data for compound **3aa** (thermal ellipsoids at 30% probability).

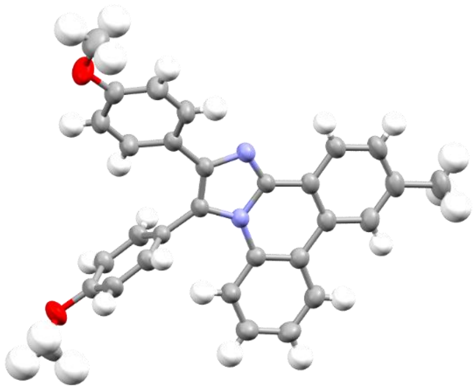

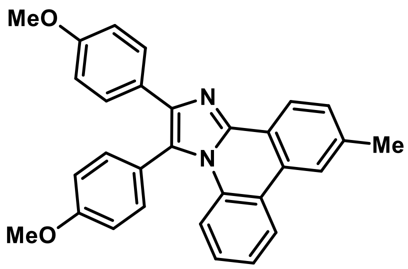

Empirical formula	$C_{30}H_{24}N_2O_2$
Temperature	296(2) K
Wavelength	0.71073 Å
Space group	P2(1)/n
Unit cell dimensions	a = 8.6940(13) Å<br>b = 14.963(2) Å<br>c = 17.715(3) Å<br>alpha = 90 deg.<br>beta = 92.820(2) deg.<br>gamma = 90.00 deg.
Volume	2301.8(6) $Å^3$
Z	4
Calculated density	1.283 Mg/m^3
Absorption coefficient	0.081 mm^{-1}
F(000)	936
Crystal size	0.126 x 0.112 x 0.098 mm
Theta range for data collection	2.30 to 27.62 deg.
Reflections collected / unique	11563 / 4051 [R(int) = 0.0214]
Data / restraints / parameters	4051 / 0 / 307
Goodness-of-fit on F^2	1.025
Final R indices [I>2sigma(I)]	R1 = 0.0458, wR2 = 0.1228
R indices (all data)	R1 = 0.0617, wR2 = 0.1341

Table S2. Crystal structure and refinement data for compound **4h** (thermal ellipsoids at 30% probability).

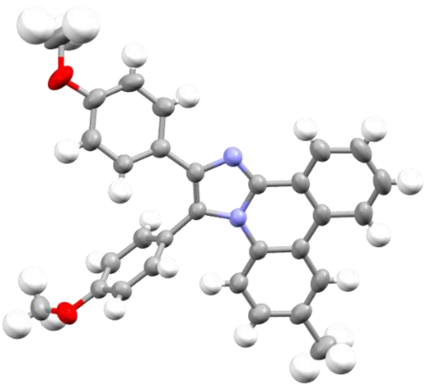

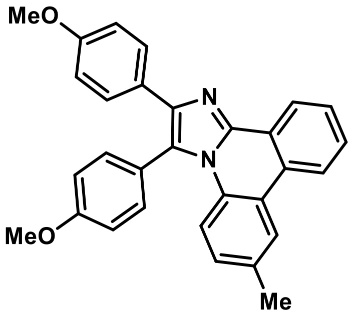

Empirical formula	$C_{30}H_{24}N_2O_2$
Temperature	296(2) K
Wavelength	0.71073 Å
Space group	P2(1)/c
Unit cell dimensions	a = 14.501(3) Å<br>b = 9.652(2) Å<br>c = 17.869(4) Å<br>alpha = 90 deg.<br>beta = 113.419(16) deg.<br>gamma = 90 deg.
Volume	2295.0(9) $Å^3$
Z	4
Calculated density	1.287 Mg/m^3
Absorption coefficient	0.081 mm^{-1}
F(000)	936
Crystal size	0.112 x 0.100 x 0.097 mm
Theta range for data collection	3.082 to 25.019 deg.
Reflections collected / unique	11399 / 4052 [R(int) = 0.0201]
Data / restraints / parameters	4052 / 0 / 309
Goodness-of-fit on F^2	1.012
Final R indices [I>2sigma(I)]	R1 = 0.0421, wR2 = 0.1080
R indices (all data)	R1 = 0.0589, wR2 = 0.1185

III. Optimization of reaction conditions

Table S3. Optimization of the reaction conditions.[a,b]

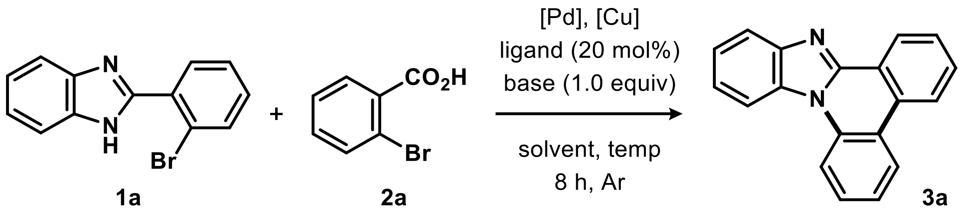

Entry	[Pd]	[Cu]	Ligand	Base	Solvent	Temp (°C)	Yield (%)[b]
1	$Pd(OAc)_2$	–	–	Cs_2CO_3	DMF	80	14
2	$Pd(OAc)_2$	CuI	–	Cs_2CO_3	DMF	80	35
3	$PdCl_2$	CuI	–	Cs_2CO_3	DMF	80	31
4	$(\eta^3\text{-}C_3H_5)_2Pd_2Cl_2$	CuI	–	Cs_2CO_3	DMF	80	28
5	$Pd(OAc)_2$	CuBr	–	Cs_2CO_3	DMF	80	6
6	$Pd(OAc)_2$	$Cu(OAc)_2$	–	Cs_2CO_3	DMF	80	0
7	$Pd(OAc)_2$	$Cu(OTf)_2$	–	Cs_2CO_3	DMF	80	0
8	$Pd(OAc)_2$	CuI	PPh_3	Cs_2CO_3	DMF	80	51
9	$Pd(OAc)_2$	CuI	*o*-phen	Cs_2CO_3	DMF	80	45
10	$Pd(OAc)_2$	CuI	L-proline	Cs_2CO_3	DMF	80	39
11	$Pd(OAc)_2$	CuI	PPh_3	K_2CO_3	DMF	80	24
12	$Pd(OAc)_2$	CuI	PPh_3	Na_2CO_3	DMF	80	17
13	$Pd(OAc)_2$	CuI	PPh_3	Cs_2CO_3	DMSO	80	41
14	$Pd(OAc)_2$	CuI	PPh_3	Cs_2CO_3	DMAc	80	26
15	$Pd(OAc)_2$	CuI	PPh_3	Cs_2CO_3	toluene	80	12
16	$Pd(OAc)_2$	CuI	PPh_3	Cs_2CO_3	DMF	100	65
**17**	**$Pd(OAc)_2$**	**CuI**	**PPh_3**	**Cs_2CO_3**	**DMF**	**110**	**77**
18	$Pd(OAc)_2$	CuI	PPh_3	Cs_2CO_3	DMF	120	79
19	$Pd(OAc)_2$	CuI	PPh_3	Cs_2CO_3	DMF	60	0
20	$Pd(OAc)_2$	–	PPh_3	Cs_2CO_3	DMF	110	23
21	–	CuI	PPh_3	Cs_2CO_3	DMF	110	0
22	$Pd(PPh_3)_4$	–	–	Cs_2CO_3	DMF	110	19
23	$Pd(PPh_3)_4$	CuI	–	Cs_2CO_3	DMF	110	69

[a] Reaction conditions: **1a** (136 mg, 0.500 mmol, 1.00 equiv), **2a** (150 mg, 0.750 mmol, 1.50 equiv), [Pd] catalyst (0.025 mmol, 0.050 equiv), [Cu] catalyst (0.050 mmol, 0.10 equiv), ligand (0.10 mmol, 0.20 equiv), base (0.500 mmol, 1.00 equiv), solvent (5.0 mL), 60–120 °C. [b] Isolated yields of **3a** after purification by column chromatography.

IV. Synthesis and analytical data of compounds 1 and 3

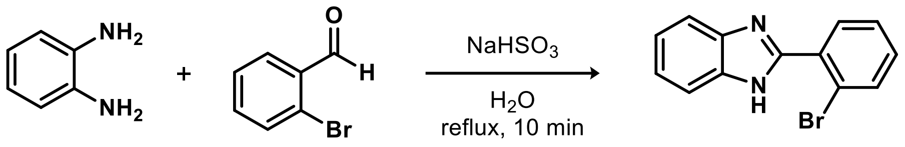

General procedure for preparation of 2-(2-bromophenyl)-1*H*-benzo[d]imidazole:[1] In a round-bottom flask (10 mL) equipped with a magnetic stirrer, a mixture of the aldehyde (0.185 g, 1.00 mmol, 1.00 equiv) and $NaHSO_3$ (1.14 g, 11.0 mmol, 11.0 equiv) in H_2O (4.0 mL) was prepared. Then, the mixture was refluxed in an oil bath and the *o*-phenylenediamine derivative (0.108 g, 1.00 mmol, 1.00 equiv) was added. The resulting mixture was monitored by TLC and stirred for the appropriate time. Upon completion of the reaction, the reaction mixture was allowed to cool to room temperature and then filtered through a glass funnel. The residues were washed with water (4 mL × 2) and dried under air.

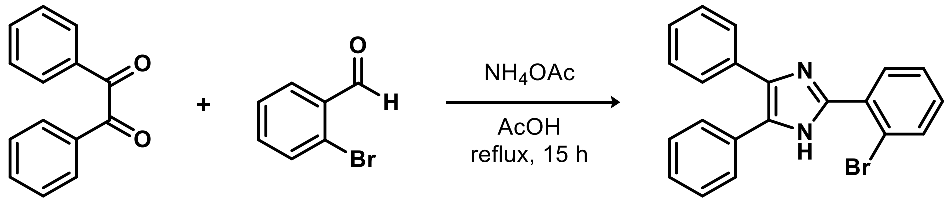

General procedure for preparation of 2-(2-bromophenyl)-4,5-diphenyl-1*H*-imidazole:[2] A mixture of the aldehyde (0.185 g, 1.00 mmol, 1.00 equiv) and the benzoin derivative (0.210 g, 1.00 mmol, 1.00 equiv) in glacial acetic acid (4.0 mL) was prepared in a round-bottom flask (10 mL) equipped with a magnetic stirrer. Then, the mixture was refluxed in an oil bath and ammonium acetate (0.231 g, 3.00 mmol, 3.00 equiv) was added. The resulting mixture was monitored by TLC and stirred for the appropriate time. Upon completion of the reaction, the reaction mixture was poured into water (20 mL). The precipitate was filtered, rinsed with water (4 mL × 2) and dried under air.

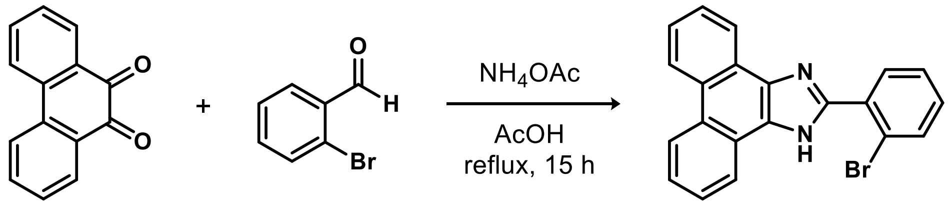

General procedure for preparation of 2-(2-bromophenyl)-1*H*-phenanthro[9,10-*d*]imidazole:[2] A mixture of the aldehyde (0.185 g, 1.00 mmol, 1.00 equiv) and the phenanthrene-9,10-dione (0.209 g,

1.00 mmol, 1.00 equiv) in glacial acetic acid (4.0 mL) was prepared in a round-bottom flask (10 mL) equipped with a magnetic stirrer. Then, the mixture was refluxed in an oil bath and ammonium acetate (0.231 g, 3.00 mmol, 3.00 equiv) was added. The resulting mixture was monitored by TLC and stirred for the appropriate time. Upon completion of the reaction, the reaction mixture was poured into water (20 mL). The precipitate was filtered, rinsed with water (4 mL × 2) and dried under air.

Note: All o-bromobenzoic acids 2 are commercially available.

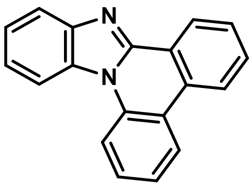

Benzo[4,5]imidazo[1,2-*f*]phenanthridine (3a)[3]

To a 10 mL Schlenk tube equipped with a magnetic stir bar was added 2-(2-bromophenyl)-1*H*-benzo[*d*]imidazole **1a** (136.5 mg, 0.500 mmol, 1.00 equiv), *o*-bromobenzoic acid **2a** (150.7 mg, 0.750 mmol, 1.50 equiv), DMF (4.0 mL), Cs_2CO_3 (163 mg, 0.500 mmol, 1.00 equiv), PPh_3 (26.2 mg, 0.100 mmol, 0.200 equiv), $Pd(OAc)_2$ (5.6 mg, 0.025 mmol, 0.050 equiv), and CuI (9.5 mg, 0.050 mmol, 0.10 equiv). The reaction mixture was stirred at 110 °C in an oil bath for about 8 h. The resulting mixture was concentrated and the residue was taken up in ethyl acetate. The organic layer was washed with brine, dried over Na_2SO_4 and concentrated. Purification of the crude product by column chromatography (silica gel; petroleum ether/ethyl acetate 10:1) afforded **3a** in 77% yield (103 mg). White solid; mp 145–147 °C; ^{1}H NMR ($CDCl_3$, 400 MHz): δ_H 8.91 (dd, *J* = 7.6, 1.2 Hz, 1H), 8.62 (d, *J* = 8.4 Hz, 1H), 8.53 (dd, *J* = 8.0, 1.6 Hz, 1H), 8.43 (d, *J* = 8.0 Hz, 1H), 8.39 (d, *J* = 8.0 Hz, 1H), 8.07 (dd, *J* = 7.6, 1.6 Hz, 1H), 7.78–7.69 (m, 3H), 7.57–7.49 (m, 3H); ^{13}C NMR ($CDCl_3$, 101 MHz): δ_C 147.5, 144.5, 134.5, 131.9, 130.5, 129.5, 129.2, 128.7, 126.1, 124.5, 124.3, 124.1, 123.5, 122.9, 122.3, 121.8, 120.4, 116.1, 113.9; HRMS (ESI-TOF, *m/z*): calcd for $C_{19}H_{13}N_2$ $[M + H]^+$, 269.1073; found, 269.1087.

1 mmol scale synthesis of benzo[4,5]imidazo[1,2-*f*]phenanthridine (3a): To a 25 mL Schlenk tube equipped with a magnetic stir bar was added 2-(2-bromophenyl)-1*H*-benzo[*d*]imidazole **1a** (273 mg, 1.00 mmol, 1.00 equiv), *o*-bromobenzoic acid **2a** (301.4 mg, 1.50 mmol, 1.50 equiv), DMF (8 mL),

Cs_2CO_3 (326 mg, 1.00 mmol, 1.00 equiv), PPh_3 (52.4 mg, 0.20 mmol, 0.20 equiv), $Pd(OAc)_2$ (11.2 mg, 0.050 mmol, 0.050 equiv), and CuI (19 mg, 0.10 mmol, 0.10 equiv). The reaction mixture was stirred at 110 °C in an oil bath for about 8 h. The resulting mixture was concentrated and the residue was taken up in ethyl acetate. The organic layer was washed with brine, dried over Na_2SO_4 and concentrated. Purification of the crude product by column chromatography (silica gel; petroleum ether/ethyl acetate 10:1) afforded **3a** as white solid in 74% yield (199 mg).

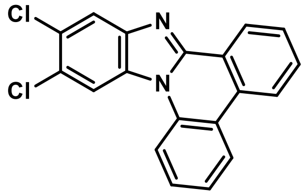

11,12-Dichlorobenzo[4,5]imidazo[1,2-*f*]phenanthridine (3b)[3]

To a 10 mL Schlenk tube equipped with a magnetic stir bar was added 2-(2-bromophenyl)-5,6-dichloro-1*H*-benzo[*d*]imidazole **1b** (171 mg, 0.500 mmol, 1.00 equiv), *o*-bromobenzoic acid **2a** (150.7 mg, 0.750 mmol, 1.50 equiv), DMF (4.0 mL), Cs_2CO_3 (163 mg, 0.500 mmol, 1.00 equiv), PPh_3 (26.2 mg, 0.10 mmol, 0.20 equiv), $Pd(OAc)_2$ (5.6 mg, 0.025 mmol, 0.050 equiv), and CuI (9.5 mg, 0.050 mmol, 0.10 equiv). The reaction mixture was stirred at 110 °C in an oil bath for about 8 h. The resulting mixture was concentrated and the residue was taken up in ethyl acetate. The organic layer was washed with brine, dried over Na_2SO_4 and concentrated. Purification of the crude product by column chromatography (silica gel; petroleum ether/ethyl acetate 10:1) afforded **3b** in 66% yield (111 mg).

White solid; mp 231–232 °C; ^{1}H NMR ($CDCl_3$, 400 MHz): δ_H 8.79 (d, J = 8.0 Hz, 1H), 8.49 (d, J = 8.0 Hz, 1H), 8.40–8.33 (m, 3H), 8.06 (s, 1H), 7.79–7.67 (m, 3H), 7.58–7.54 (m, 1H); ^{13}C NMR ($CDCl_3$, 101 MHz): δ_C 149.2, 144.0, 133.7, 131.1, 130.8, 129.7, 129.5, 128.9, 128.2, 126.5, 126.2, 125.1, 124.4, 122.9, 122.4, 121.8, 121.1, 115.7, 115.1; HRMS (ESI, *m/z*): calcd for $C_{19}H_{11}Cl_2N_2$ $[M + H]^+$, 337.0294; found, 337.0299.

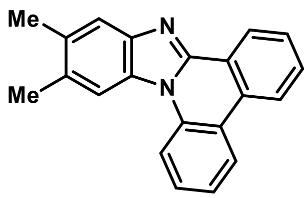

11,12-Dimethylbenzo[4,5]imidazo[1,2-*f*]phenanthridine (3c)[4]

To a 10 mL Schlenk tube equipped with a magnetic stir bar was added 2-(2-bromophenyl)-5,6-dimethyl-1*H*-benzo[*d*]imidazole **1c** (150.5 mg, 0.500 mmol, 1.00 equiv), *o*-bromobenzoic acid **2a** (150.7 mg, 0.750 mmol, 1.50 equiv), DMF (4.0 mL), Cs_2CO_3 (163 mg, 0.500 mmol, 1.00 equiv), PPh_3 (26.2 mg, 0.10 mmol, 0.20 equiv), $Pd(OAc)_2$ (5.6 mg, 0.025 mmol, 0.050 equiv), and CuI (9.5 mg, 0.050 mmol, 0.10 equiv). The reaction mixture was stirred at 110 °C in an oil bath for about 8 h. The resulting mixture was concentrated and the residue was taken up in ethyl acetate. The organic layer was washed with brine, dried over Na_2SO_4 and concentrated. Purification of the crude product by column chromatography (silica gel; petroleum ether/ethyl acetate 10:1) afforded **3c** in 74% yield (109 mg).

White solid; mp 161–162 °C; ^{1}H NMR ($CDCl_3$, 400 MHz): δ_H 8.76 (d, *J* = 7.6 Hz, 1H), 8.36–8.32 (m, 2H), 8.25 (d, *J* = 7.2 Hz, 1H), 7.92 (s, 1H), 7.70 (s, 1H), 7.66–7.56 (m, 3H), 7.40–7.35 (m, 1H), 2.45 (s, 3H), 2.40 (s, 3H); ^{13}C NMR ($CDCl_3$, 101 MHz): δ_C 146.7, 143.0, 134.4, 133.0, 131.9, 130.2, 129.9, 129.2, 128.9, 128.4, 125.8, 124.0, 123.6, 122.1, 121.4, 120.2, 115.8, 114.0, 21.0, 20.3; HRMS (ESI-TOF, *m/z*): calcd for $C_{21}H_{17}N_2$ $[M + H]^+$, 297.1386; found, 297.1394.

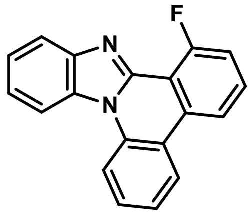

8-Fluorobenzo[4,5]imidazo[1,2-*f*]phenanthridine (3d)

To a 10 mL Schlenk tube equipped with a magnetic stir bar was added 2-(2-bromo-6-fluorophenyl)-1*H*-benzo[*d*]imidazole **1d** (145.5 mg, 0.500 mmol, 1.00 equiv), *o*-bromobenzoic acid **2a** (150.7 mg, 0.750 mmol, 1.50 equiv), DMF (4.0 mL), Cs_2CO_3 (163 mg, 0.50 mmol, 1.00 equiv), PPh_3 (26.2 mg, 0.10 mmol, 0.20 equiv), $Pd(OAc)_2$ (5.6 mg, 0.025 mmol, 0.050 equiv), and CuI (9.5 mg, 0.050 mmol, 0.10 equiv). The reaction mixture was stirred at 110 °C in an oil bath for about 8 h. The resulting mixture was concentrated and the residue was taken up in ethyl acetate. The organic layer was washed with

brine, dried over Na_2SO_4 and concentrated. Purification of the crude product by column chromatography (silica gel; petroleum ether/ethyl acetate 10:1) afforded **3d** in 75% yield (107 mg).

White solid; mp 183–185 °C; ^1H NMR ($CDCl_3$, 400 MHz): δ_H 8.57 (d, J = 8.4 Hz, 1H), 8.43 (d, J = 8.0 Hz, 1H), 8.35 (d, J = 7.6 Hz, 1H), 8.20–8.15 (m, 2H), 7.73–7.65 (m, 2H), 7.56–7.47 (m, 3H), 7.44–7.40 (m, 1H); ^{13}C NMR ($CDCl_3$, 101 MHz): δ_C 160.7 (d, J_{C-F} = 259.3 Hz), 144.8 (d, J_{C-F} = 2.9 Hz), 143.8 (d, J_{C-F} = 8.6 Hz), 134.6, 132.1 (d, J_{C-F} = 2.1 Hz), 130.8, 130.7, 129.9, 124.7, 124.6, 124.2, 123.5, 121.2, 120.9 (d, J_{C-F} = 2.4 Hz), 118.1 (d, J_{C-F} = 4.1 Hz), 116.0, 115.7 (d, J_{C-F} = 21.3 Hz), 113.7, 112.8 (d, J_{C-F} = 9.9 Hz); ^{19}F NMR ($CDCl_3$, 376 MHz): δ_F –107.5; HRMS (ESI-TOF, *m/z*): calcd for $C_{19}H_{12}FN_2$ $[M + H]^+$, 287.0979; found, 287.0993.

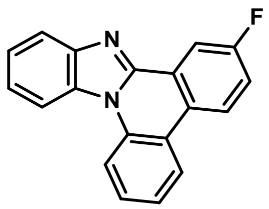

7-Fluorobenzo[4,5]imidazo[1,2-*f*]phenanthridine (3e)

To a 10 mL Schlenk tube equipped with a magnetic stir bar was added 2-(2-bromo-5-fluorophenyl)-1*H*-benzo[*d*]imidazole **1e** (145.5 mg, 0.500 mmol, 1.00 equiv), *o*-bromobenzoic acid **2a** (150.7 mg, 0.750 mmol, 1.50 equiv), DMF (4.0 mL), Cs_2CO_3 (163 mg, 0.500 mmol, 1.00 equiv), PPh_3 (26.2 mg, 0.10 mmol, 0.20 equiv), $Pd(OAc)_2$ (5.6 mg, 0.025 mmol, 0.050 equiv), and CuI (9.5 mg, 0.050 mmol, 0.10 equiv). The reaction mixture was stirred at 110 °C in an oil bath for about 8 h. The resulting mixture was concentrated and the residue was taken up in ethyl acetate. The organic layer was washed with brine, dried over Na_2SO_4 and concentrated. Purification of the crude product by column chromatography (silica gel; petroleum ether/ethyl acetate 10:1) afforded **3e** in 69% yield (98 mg).

White solid; mp 180–182 °C; ^1H NMR ($CDCl_3$, 400 MHz): δ_H 8.61 (d, J = 8.4 Hz, 1H), 8.54 (dd, J = 9.2, 2.8 Hz, 1H), 8.46–8.37 (m, 3H), 8.07 (d, J = 7.6 Hz, 1H), 7.75–7.71 (m, 1H), 7.57–7.44 (m, 4H); ^{13}C NMR ($CDCl_3$, 101 MHz): δ_C 162.6 (d, J_{C-F} = 248.2 Hz), 146.6, 144.4, 134.0, 131.9, 129.0, 125.9 (d, J_{C-F} = 2.2 Hz), 125.3 (d, J_{C-F} = 9.4 Hz), 124.8 (d, J_{C-F} = 8.3 Hz), 124.6, 124.3, 124.1, 123.3, 121.2, 120.6, 118.7 (d, J_{C-F} = 23.1 Hz), 116.1, 114.0, 111.5 (d, J_{C-F} = 23.6 Hz); ^{19}F NMR ($CDCl_3$, 376 MHz): δ_F –111.3; HRMS (ESI-TOF, *m/z*): calcd for $C_{19}H_{12}FN_2$ $[M + H]^+$, 287.0979; found, 287.0993.

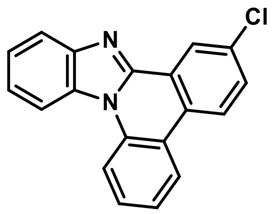

7-Chlorobenzo[4,5]imidazo[1,2-*f*]phenanthridine (3f)[3]

To a 10 mL Schlenk tube equipped with a magnetic stir bar was added 2-(2-bromo-5-chlorophenyl)-1*H*-benzo[*d*]imidazole **1f** (153.5 mg, 0.500 mmol, 1.00 equiv), *o*-bromobenzoic acid **2a** (150.7 mg, 0.750 mmol, 1.50 equiv), DMF (4.0 mL), Cs_2CO_3 (163 mg, 0.500 mmol, 1.00 equiv), PPh_3 (26.2 mg, 0.10 mmol, 0.20 equiv), $Pd(OAc)_2$ (5.6 mg, 0.025 mmol, 0.050 equiv), and CuI (9.5 mg, 0.050 mmol, 0.10 equiv). The reaction mixture was stirred at 110 °C in an oil bath for about 8 h. The resulting mixture was concentrated and the residue was taken up in ethyl acetate. The organic layer was washed with brine, dried over Na_2SO_4 and concentrated. Purification of the crude product by column chromatography (silica gel; petroleum ether/ethyl acetate 10:1) afforded **3f** in 79% yield (119 mg).

White solid; mp 216–217 °C; ^{1}H NMR ($CDCl_3$, 400 MHz): δ_H 8.76 (d, *J* = 2.0 Hz, 1H), 8.45 (d, *J* = 8.4 Hz, 1H), 8.31–8.25 (m, 2H), 8.18 (d, *J* = 8.4 Hz, 1H), 8.01 (d, *J* = 8.0 Hz, 1H), 7.66–7.58 (m, 2H), 7.52–7.42 (m, 3H); ^{13}C NMR ($CDCl_3$, 101 MHz): δ_C 146.1, 144.3, 134.7, 134.1, 131.7, 130.5, 129.4, 127.7, 125.4, 124.5, 124.3, 124.0, 123.8, 123.2, 120.8, 120.5, 115.9, 113.9; HRMS (ESI-TOF, *m/z*): calcd for $C_{19}H_{12}ClN_2$ $[M + H]^+$, 303.0684; found, 303.0691.

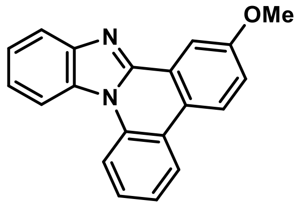

7-Methoxybenzo[4,5]imidazo[1,2-*f*]phenanthridine (3g)[3]

To a 10 mL Schlenk tube equipped with a magnetic stir bar was added 2-(2-bromo-5-methoxyphenyl)-1*H*-benzo[*d*]imidazole **1g** (151.5 mg, 0.500 mmol, 1.00 equiv), *o*-bromobenzoic acid **2a** (150.7 mg, 0.750 mmol, 1.50 equiv), DMF (4.0 mL), Cs_2CO_3 (163 mg, 0.500 mmol, 1.00 equiv), PPh_3 (26.2 mg, 0.10 mmol, 0.20 equiv), $Pd(OAc)_2$ (5.6 mg, 0.025 mmol, 0.050 equiv), and CuI (9.5 mg, 0.050 mmol, 0.10 equiv). The reaction mixture was stirred at 110 °C in an oil bath for about 8 h. The resulting mixture was concentrated and the residue was taken up in ethyl acetate. The organic layer was washed with

brine, dried over Na_2SO_4 and concentrated. Purification of the crude product by column chromatography (silica gel; petroleum ether/ethyl acetate 10:1) afforded **3g** in 69% yield (103 mg). White solid; mp 168–169 °C; ^{1}H NMR ($CDCl_3$, 400 MHz): δ_H 8.60 (d, J = 8.4 Hz, 1H), 8.44–8.39 (m, 2H), 8.33 (d, J = 9.2 Hz, 1H), 8.29 (d, J = 2.4 Hz, 1H), 8.08 (dd, J = 7.6, 1.6 Hz, 1H), 7.70–7.66 (m, 1H), 7.56–7.48 (m, 3H), 7.35 (dd, J = 9.2, 2.8 Hz, 1H), 4.07 (s, 3H); ^{13}C NMR ($CDCl_3$, 101 MHz): δ_C 160.0, 149.1, 147.5, 144.5, 133.5, 132.0, 128.1, 124.8, 124.5, 124.1, 123.7, 123.2, 122.9, 121.9, 120.6, 120.3, 116.0, 114.0, 106.5, 55.9; HRMS (ESI-TOF, *m/z*): calcd for $C_{20}H_{15}N_2O$ [M + H]$^+$, 299.1179; found, 299.1190.

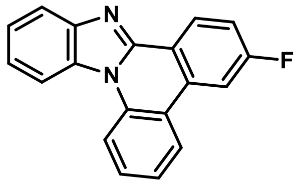

6-Fluorobenzo[4,5]imidazo[1,2-*f*]phenanthridine (3h)[5]

To a 10 mL Schlenk tube equipped with a magnetic stir bar was added 2-(2-bromo-4-fluorophenyl)-1*H*-benzo[*d*]imidazole **1h** (145.5 mg, 0.500 mmol, 1.00 equiv), *o*-bromobenzoic acid **2a** (150.7 mg, 0.750 mmol, 1.50 equiv), DMF (4.0 mL), Cs_2CO_3 (163 mg, 0.500 mmol, 1.00 equiv), PPh_3 (26.2 mg, 0.10 mmol, 0.20 equiv), $Pd(OAc)_2$ (5.6 mg, 0.025 mmol, 0.050 equiv), and CuI (9.5 mg, 0.050 mmol, 0.10 equiv). The reaction mixture was stirred at 110 °C in an oil bath for about 8 h. The resulting mixture was concentrated and the residue was taken up in ethyl acetate. The organic layer was washed with brine, dried over Na_2SO_4 and concentrated. Purification of the crude product by column chromatography (silica gel; petroleum ether/ethyl acetate 10:1) afforded **3h** in 67% yield (96 mg). White solid; mp 151–154 °C; ^{1}H NMR ($CDCl_3$, 400 MHz): δ_H 8.90 (dd, J = 9.2, 6.0 Hz, 1H), 8.62 (d, J = 8.4 Hz, 1H), 8.40 (d, J = 8.0 Hz, 1H), 8.37 (d, J = 8.0 Hz, 1H), 8.06–8.02 (m, 2H), 7.79–7.75 (m, 1H), 7.58–7.48 (m, 3H), 7.45–7.40 (m, 1H); ^{13}C NMR ($CDCl_3$, 101 MHz): δ_C 166.0, 164.3 (d, $J_{C\text{–}F}$ = 248.8 Hz), 147.1, 144.5, 134.9, 131.8, 130.0, 128.8 (d, $J_{C\text{–}F}$ = 9.5 Hz), 124.6 (d, $J_{C\text{–}F}$ = 4.6 Hz), 124.3, 123.0, 120.4, 120.1, 117.0 (d, $J_{C\text{–}F}$ = 23.5 Hz), 116.2, 113.9, 108.5 (d, $J_{C\text{–}F}$ = 23.7 Hz); ^{19}F NMR ($CDCl_3$, 376 MHz): δ_F –108.1; HRMS (ESI-TOF, *m/z*): calcd for $C_{19}H_{12}FN_2$ [M + H]$^+$, 287.0979; found, 287.0993.

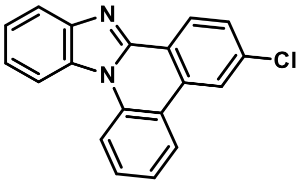

6-Chlorobenzo[4,5]imidazo[1,2-*f*]phenanthridine (3i)[3]

To a 10 mL Schlenk tube equipped with a magnetic stir bar was added 2-(2-bromo-4-chlorophenyl)-1*H*-benzo[*d*]imidazole **1i** (153.5 mg, 0.500 mmol, 1.00 equiv), *o*-bromobenzoic acid **2a** (150.7 mg, 0.750 mmol, 1.50 equiv), DMF (4.0 mL), Cs_2CO_3 (163 mg, 0.500 mmol, 1.00 equiv), PPh_3 (26.2 mg, 0.10 mmol, 0.20 equiv), $Pd(OAc)_2$ (5.6 mg, 0.025 mmol, 0.050 equiv), and CuI (9.5 mg, 0.050 mmol, 0.10 equiv). The reaction mixture was stirred at 110 °C in an oil bath for about 8 h. The resulting mixture was concentrated and the residue was taken up in ethyl acetate. The organic layer was washed with brine, dried over Na_2SO_4 and concentrated. Purification of the crude product by column chromatography (silica gel; petroleum ether/ethyl acetate 10:1) afforded **3i** in 64% yield (97 mg).

White solid; mp 197–198 °C; ^{1}H NMR ($CDCl_3$, 400 MHz): δ_H 8.82 (d, *J* = 8.4 Hz, 1H), 8.60 (d, *J* = 8.4 Hz, 1H), 8.43 (d, *J* = 8.0 Hz, 1H), 8.37–8.35 (m, 2H), 8.06–8.04 (m, 1H), 7.76 (t, *J* = 8.0 Hz, 1H), 7.66 (dd, *J* = 8.8, 2.0 Hz, 1H), 7.57–7.50 (m, 3H); ^{13}C NMR ($CDCl_3$, 101 MHz): δ_C 146.7, 144.5, 136.8, 134.7, 131.7, 130.8, 129.8, 129.0, 127.5, 124.5, 124.3, 124.27, 123.2, 122.2, 121.8, 120.5, 120.4, 116.0, 113.9; HRMS (ESI-TOF, *m/z*): calcd for $C_{19}H_{12}ClN_2$ $[M + H]^+$, 303.0684; found, 303.0691.

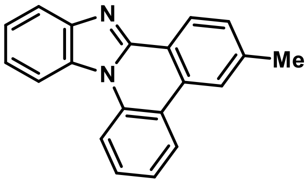

6-Methylbenzo[4,5]imidazo[1,2-*f*]phenanthridine (3j)[3]

To a 10 mL Schlenk tube equipped with a magnetic stir bar was added 2-(2-bromo-4-methylphenyl)-1*H*-benzo[*d*]imidazole **1j** (143.5 mg, 0.500 mmol, 1.00 equiv), *o*-bromobenzoic acid **2a** (150.7 mg, 0.750 mmol, 1.50 equiv), DMF (4.0 mL), Cs_2CO_3 (163 mg, 0.500 mmol, 1.00 equiv), PPh_3 (26.2 mg, 0.10 mmol, 0.20 equiv), $Pd(OAc)_2$ (5.6 mg, 0.025 mmol, 0.050 equiv), and CuI (9.5 mg, 0.050 mmol, 0.10 equiv). The reaction mixture was stirred at 110 °C in an oil bath for about 8 h. The resulting mixture was concentrated and the residue was taken up in ethyl acetate. The organic layer was washed with

brine, dried over Na_2SO_4 and concentrated. Purification of the crude product by column chromatography (silica gel; petroleum ether/ethyl acetate 10:1) afforded **3j** in 71% yield (100 mg). White solid; mp 156–157 °C; ^{1}H NMR ($CDCl_3$, 400 MHz): δ_H 8.78 (d, J = 8.4 Hz, 1H), 8.60 (d, J = 8.4 Hz, 1H), 8.52 (d, J = 8.0 Hz, 1H), 8.37 (d, J = 8.4 Hz, 1H), 8.21 (s, 1H), 8.05 (d, J = 8.0 Hz, 1H), 7.72 (t, J = 7.6 Hz, 1H), 7.55–7.47 (m, 3H), 2.62 (s, 3H); ^{13}C NMR ($CDCl_3$, 101 MHz): δ_C 147.8, 144.6, 140.7, 134.6, 131.9, 130.1, 129.5, 129.0, 126.0, 124.3, 124.2, 124.0, 122.7, 122.4, 121.7, 121.1, 120.2, 116.0, 113.8, 22.2; HRMS (ESI-TOF, *m/z*): calcd for $C_{20}H_{15}N_2$ $[M + H]^+$, 283.1230; found, 283.1242.

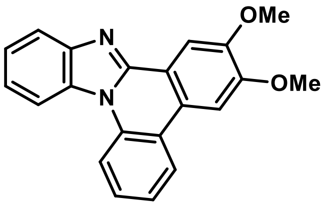

6,7-Dimethoxybenzo[4,5]imidazo[1,2-*f*]phenanthridine (3k)

To a 10 mL Schlenk tube equipped with a magnetic stir bar was added 2-(2-bromo-4,5-dimethoxyphenyl)-1*H*-benzo[*d*]imidazole **1k** (166.5 mg, 0.500 mmol, 1.00 equiv), *o*-bromobenzoic acid **2a** (150.7 mg, 0.750 mmol, 1.50 equiv), DMF (4.0 mL), Cs_2CO_3 (163 mg, 0.500 mmol, 1.00 equiv), PPh_3 (26.2 mg, 0.10 mmol, 0.20 equiv), $Pd(OAc)_2$ (5.6 mg, 0.025 mmol, 0.050 equiv), and CuI (9.5 mg, 0.050 mmol, 0.10 equiv). The reaction mixture was stirred at 110 °C in an oil bath for about 8 h. The resulting mixture was concentrated and the residue was taken up in ethyl acetate. The organic layer was washed with brine, dried over Na_2SO_4 and concentrated. Purification of the crude product by column chromatography (silica gel; petroleum ether/ethyl acetate 10:1) afforded **3k** in 70% yield (115 mg). White solid; mp 145–146 °C; ^{1}H NMR ($CDCl_3$, 400 MHz): δ_H 8.62 (d, J = 8.4 Hz, 1H), 8.41–8.37 (m, 2H), 8.25 (s, 1H), 8.05 (d, J = 8.0 Hz, 1H), 7.76 (s, 1H), 7.72–7.68 (m, 1H), 7.56–7.45 (m, 3H), 4.16 (s, 3H), 4.14 (s, 3H); ^{13}C NMR ($CDCl_3$, 101 MHz): δ_C 152.1, 150.6, 133.9, 131.9, 128.4, 124.4, 124.2, 123.7, 122.6, 121.7, 119.8, 116.2, 114.0, 111.1, 108.5, 106.7, 103.6, 100.2, 56.6, 56.2; HRMS (ESI-TOF, *m/z*): calcd for $C_{21}H_{17}N_2O_2$ $[M + H]^+$, 329.1285; found, 329.1287.

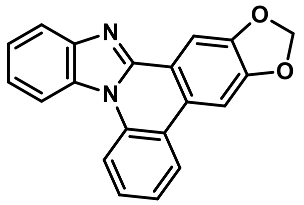

Benzo[4,5]imidazo[1,2-*f*][1,3]dioxolo[4,5-*j*]phenanthridine (3l)

To a 10 mL Schlenk tube equipped with a magnetic stir bar was added 2-(6-bromobenzo[*d*][1,3]dioxol-5-yl)-1*H*-benzo[*d*]imidazole **1l** (158.5 mg, 0.500 mmol, 1.00 equiv), *o*-bromobenzoic acid **2a** (150.7 mg, 0.750 mmol, 1.50 equiv), DMF (4.0 mL), Cs_2CO_3 (163 mg, 0.500 mmol, 1.00 equiv), PPh_3 (26.2 mg, 0.10 mmol, 0.20 equiv), $Pd(OAc)_2$ (5.6 mg, 0.025 mmol, 0.050 equiv), and CuI (9.5 mg, 0.050 mmol, 0.10 equiv). The reaction mixture was stirred at 110 °C in an oil bath for about 8 h. The resulting mixture was concentrated and the residue was taken up in ethyl acetate. The organic layer was washed with brine, dried over Na_2SO_4 and concentrated. Purification of the crude product by column chromatography (silica gel; petroleum ether/ethyl acetate 10:1) afforded **3l** in 75% yield (117 mg). White solid; mp 200–201 °C; ^{1}H NMR ($CDCl_3$, 400 MHz): δ_H 8.56 (d, *J* = 8.4 Hz, 1H), 8.34 (d, *J* = 8.4 Hz, 1H), 8.28 (d, *J* = 8.0 Hz, 1H), 8.23 (s, 1H), 8.01 (d, *J* = 8.0 Hz, 1H), 7.73 (s, 1H), 7.68–7.65 (m, 1H), 7.53–7.44 (m, 3H), 6.15 (s, 2H); ^{13}C NMR ($CDCl_3$, 101 MHz): δ_C 150.9, 148.8, 147.6, 144.5, 133.8, 131.8, 128.4, 125.8, 124.3, 124.1, 123.9, 122.5, 121.7, 120.1, 119.0, 116.0, 113.9, 104.5, 102.0, 101.4; HRMS (ESI-TOF, *m/z*): calcd for $C_{20}H_{13}N_2O_2$ $[M + H]^+$, 313.0972; found, 313.0974.

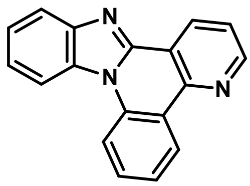

Benzo[*h*]benzo[4,5]imidazo[2,1-*f*][1,6]naphthyridine (3m)

To a 10 mL Schlenk tube equipped with a magnetic stir bar was added 2-(2-bromopyridin-3-yl)-1*H*-benzo[*d*]imidazole **1m** (158.5 mg, 0.500 mmol, 1.00 equiv), *o*-bromobenzoic acid **2a** (150.7 mg, 0.750 mmol, 1.50 equiv), DMF (4.0 mL), Cs_2CO_3 (163 mg, 0.500 mmol, 1.00 equiv), PPh_3 (26.2 mg, 0.10 mmol, 0.20 equiv), $Pd(OAc)_2$ (5.6 mg, 0.025 mmol, 0.050 equiv), and CuI (9.5 mg, 0.050 mmol, 0.10 equiv). The reaction mixture was stirred at 110 °C in an oil bath for about 8 h. The resulting mixture was concentrated and the residue was taken up in ethyl acetate. The organic layer was washed with brine,

dried over Na_2SO_4 and concentrated. Purification of the crude product by column chromatography (silica gel; petroleum ether/ethyl acetate 10:1) afforded **3m** in 78% yield (105 mg).

White solid; mp 163–164 °C; ^{1}H NMR ($CDCl_3$, 400 MHz): δ_H 9.05 (d, J = 8.0 Hz, 1H), 9.02 (d, J = 8.0 Hz, 1H), 8.95 (d, J = 4.4 Hz, 1H), 8.45 (d, J = 8.4 Hz, 1H), 8.30 (d, J = 8.0 Hz, 1H), 8.01 (d, J = 8.0 Hz, 1H), 7.75–7.72 (m, 1H), 7.58–7.46 (m, 4H); ^{13}C NMR ($CDCl_3$, 101 MHz): δ_C 151.7, 146.4, 144.5, 135.6, 133.3, 131.7, 130.8, 126.3, 124.7, 124.3, 123.4, 122.6, 120.4, 119.0, 115.3, 113.9; HRMS (ESI-TOF, *m/z*): calcd for $C_{18}H_{12}N_3$ $[M + H]^+$, 270.1026; found, 270.1045.

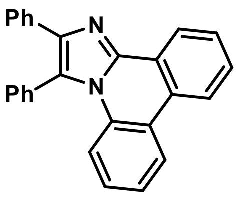

2,3-Diphenylimidazo[1,2-*f*]phenanthridine (3n)[7]

To a 10 mL Schlenk tube equipped with a magnetic stir bar was added 2-(2-bromophenyl)-4,5-diphenyl-1H-imidazole **1n** (187.6 mg, 0.500 mmol, 1.00 equiv), *o*-bromobenzoic acid **2a** (150.7 mg, 0.750 mmol, 1.50 equiv), DMF (4.0 mL), Cs_2CO_3 (163 mg, 0.500 mmol, 1.00 equiv), PPh_3 (26.2 mg, 0.10 mmol, 0.20 equiv), $Pd(OAc)_2$ (5.6 mg, 0.025 mmol, 0.050 equiv), and CuI (9.5 mg, 0.050 mmol, 0.10 equiv). The reaction mixture was stirred at 110 °C in an oil bath for about 8 h. The resulting mixture was concentrated and the residue was taken up in ethyl acetate. The organic layer was washed with brine, dried over Na_2SO_4 and concentrated. Purification of the crude product by column chromatography (silica gel; petroleum ether/ethyl acetate 10:1) afforded **3n** in 80% yield (148 mg).

White solid; mp 175–177 °C; ^{1}H NMR ($CDCl_3$, 400 MHz): δ_H 8.88–8.86 (m, 1H), 8.46 (d, J = 7.6 Hz, 1H), 8.40–8.37 (m, 1H), 7.68–7.65 (m, 2H), 7.60–7.55 (m, 7H), 7.41–7.37 (m, 1H), 7.31–7.26 (m, 2H), 7.24–7.15 (m, 3H); ^{13}C NMR ($CDCl_3$, 101 MHz): δ_C 142.5, 141.2, 134.4, 133.4, 133.1, 131.6, 129.6, 129.2, 128.6, 128.5, 128.1, 127.84, 127.81, 127.6, 126.9, 125.3, 124.6, 124.5, 124.1, 123.9, 122.8, 122.1, 117.6; HRMS (ESI-TOF, *m/z*): calcd for $C_{27}H_{19}N_2$ $[M + H]^+$, 371.1543; found, 371.1548.

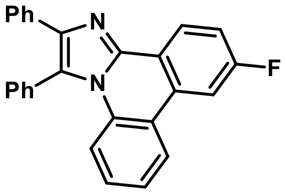

10-Fluoro-2,3-diphenylimidazo[1,2-*f*]phenanthridine (3o)

To a 10 mL Schlenk tube equipped with a magnetic stir bar was added 2-(2-bromo-4-fluorophenyl)-4,5-diphenyl-1H-imidazole **1o** (196.6 mg, 0.500 mmol, 1.00 equiv), *o*-bromobenzoic acid **2a** (150.7 mg, 0.750 mmol, 1.50 equiv), DMF (4.0 mL), Cs_2CO_3 (163 mg, 0.500 mmol, 1.00 equiv), PPh_3 (26.2 mg, 0.10 mmol, 0.20 equiv), $Pd(OAc)_2$ (5.6 mg, 0.025 mmol, 0.050 equiv), and CuI (9.5 mg, 0.050 mmol, 0.10 equiv). The reaction mixture was stirred at 110 °C in an oil bath for about 8 h. The resulting mixture was concentrated and the residue was taken up in ethyl acetate. The organic layer was washed with brine, dried over Na_2SO_4 and concentrated. Purification of the crude product by column chromatography (silica gel; petroleum ether/ethyl acetate 10:1) afforded **3o** in 78% yield (152 mg).

White solid; mp 215–217 °C; ^{1}H NMR ($CDCl_3$, 400 MHz): δ_H 8.85 (dd, *J* = 8.8, 5.6 Hz, 1H), 8.31 (d, *J* = 8.4 Hz, 1H), 7.99 (dd, *J* = 10.4, 2.4 Hz, 1H), 7.58–7.52 (m, 7H), 7.41–7.36 (m, 2H), 7.30–7.18 (m, 5H); ^{13}C NMR ($CDCl_3$, 101 MHz): δ_C 163.1 (d, $J_{C–F}$ = 246.0 Hz), 142.1, 141.2, 134.3, 133.8, 132.9, 131.6, 129.7 (d, $J_{C–F}$ = 8.7 Hz), 129.6, 129.3, 128.6, 128.1, 127.8, 127.2 (d, $J_{C–F}$ = 8.8 Hz), 127.0, 125.2, 124.6, 124.3, 122.0 (d, $J_{C–F}$ = 3.3 Hz), 120.4 (d, $J_{C–F}$ = 2.0 Hz), 117.6, 116.8 (d, $J_{C–F}$ = 23.0 Hz), 108.0 (d, $J_{C–F}$ = 23.4 Hz); ^{19}F NMR ($CDCl_3$, 376 MHz): δ_F –111.0; HRMS (ESI-TOF, *m/z*): calcd for $C_{27}H_{18}FN_2$ $[M + H]^+$, 389.1449; found, 389.1471.

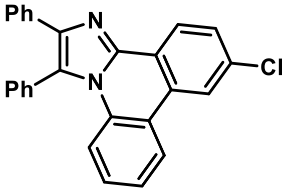

10-Chloro-2,3-diphenylimidazo[1,2-*f*]phenanthridine (3p)

To a 10 mL Schlenk tube equipped with a magnetic stir bar was added 2-(2-bromo-4-chlorophenyl)-4,5-diphenyl-1H-imidazole **1p** (204.8 mg, 0.500 mmol, 1.00 equiv), *o*-bromobenzoic acid **2a** (150.7 mg, 0.750 mmol, 1.50 equiv), DMF (4.0 mL), Cs_2CO_3 (163 mg, 0.500 mmol, 1.00 equiv), PPh_3 (26.2 mg, 0.10 mmol, 0.20 equiv), $Pd(OAc)_2$ (5.6 mg, 0.025 mmol, 0.050 equiv), and CuI (9.5 mg, 0.050 mmol, 0.10 equiv). The reaction mixture was stirred at 110 °C in an oil bath for about 8 h. The resulting mixture

was concentrated and the residue was taken up in ethyl acetate. The organic layer was washed with brine, dried over Na_2SO_4 and concentrated. Purification of the crude product by column chromatography (silica gel; petroleum ether/ethyl acetate 10:1) afforded **3p** in 73% yield (148 mg). White solid; mp 209–211 °C; ^1H NMR ($CDCl_3$, 400 MHz): δ_H 8.79 (d, J = 8.4 Hz, 1H), 8.38–8.33 (m, 2H), 7.63–7.53 (m, 8H), 7.42–7.38 (m, 1H), 7.30–7.26 (m, 2H), 7.26–7.18 (m, 3H); ^{13}C NMR ($CDCl_3$, 101 MHz): δ_C 141.9, 141.5, 134.8, 134.2, 133.7, 132.8, 131.6, 129.6, 129.4, 129.0, 128.8, 128.5, 128.1, 127.8, 127.1, 126.3, 125.5, 124.7, 124.2, 122.3, 122.1, 121.7, 117.7; HRMS (ESI-TOF, *m/z*): calcd for $C_{27}H_{18}ClN_2$ $[M + H]^+$, 405.1153; found, 405.1173.

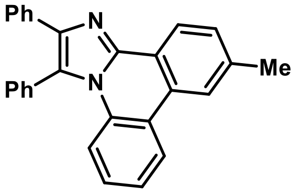

10-Methyl-2,3-diphenylimidazo[1,2-*f*]phenanthridine (3q)[7]

To a 10 mL Schlenk tube equipped with a magnetic stir bar was added 2-(2-bromo-4-methylphenyl)-4,5-diphenyl-1H-imidazole **1q** (194.6 mg, 0.500 mmol, 1.00 equiv), *o*-bromobenzoic acid **2a** (150.7 mg, 0.750 mmol, 1.00 equiv), DMF (4.0 mL), Cs_2CO_3 (163 mg, 0.500 mmol, 1.00 equiv), PPh_3 (26.2 mg, 0.10 mmol, 0.20 equiv), $Pd(OAc)_2$ (5.6 mg, 0.025 mmol, 0.050 equiv), and CuI (9.5 mg, 0.050 mmol, 0.10 equiv). The reaction mixture was stirred at 110 °C in an oil bath for about 8 h. The resulting mixture was concentrated and the residue was taken up in ethyl acetate. The organic layer was washed with brine, dried over Na_2SO_4 and concentrated. Purification of the crude product by column chromatography (silica gel; petroleum ether/ethyl acetate 10:1) afforded **3q** in 81% yield (156 mg). White solid; mp 221–223 °C; ^1H NMR ($CDCl_3$, 400 MHz): δ_H 8.73 (d, J = 8.0 Hz, 1H), 8.43 (d, J = 8.0 Hz, 1H), 8.15 (s, 1H), 7.59–7.54 (m, 7H), 7.49 (d, J = 8.0 Hz, 1H), 7.35 (t, J = 7.6 Hz, 1H), 7.28–7.25 (m, 2H), 7.23–7.12 (m, 3H), 2.58 (s, 3H); ^{13}C NMR ($CDCl_3$, 101 MHz): δ_C 142.7, 141.1, 138.6, 134.5, 133.5, 133.2, 131.6, 129.9, 129.5, 129.2, 128.1, 127.9, 127.7, 127.6, 126.8, 125.0, 124.6, 124.4, 124.0, 122.7, 122.1, 121.5, 117.6, 22.1; HRMS (ESI-TOF, *m/z*): calcd for $C_{28}H_{21}N_2$ $[M + H]^+$, 385.1699; found, 385.1711.

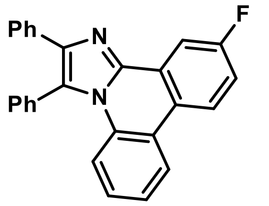

11-Fluoro-2,3-diphenylimidazo[1,2-*f*]phenanthridine (3r)

To a 10 mL Schlenk tube equipped with a magnetic stir bar was added 2-(2-bromo-5-fluorophenyl)-4,5-diphenyl-1H-imidazole **1r** (196.6 mg, 0.500 mmol, 1.00 equiv), *o*-bromobenzoic acid **2a** (150.7 mg, 0.750 mmol, 1.50 equiv), DMF (4.0 mL), Cs_2CO_3 (163 mg, 0.500 mmol, 1.00 equiv), PPh_3 (26.2 mg, 0.10 mmol, 0.20 equiv), $Pd(OAc)_2$ (5.6 mg, 0.025 mmol, 0.050 equiv), and CuI (9.5 mg, 0.050 mmol, 0.10 equiv). The reaction mixture was stirred at 110 °C in an oil bath for about 8 h. The resulting mixture was concentrated and the residue was taken up in ethyl acetate. The organic layer was washed with brine, dried over Na_2SO_4 and concentrated. Purification of the crude product by column chromatography (silica gel; petroleum ether/ethyl acetate 10:1) afforded **3r** in 71% yield (138 mg).

White solid; mp 189–191 °C; ^{1}H NMR ($CDCl_3$, 400 MHz): δ_H 8.50 (dd, J = 9.2, 2.8 Hz, 1H), 8.38–8.33 (m, 2H), 7.61–7.53 (m, 7H), 7.41–7.34 (m, 2H), 7.29–7.14 (m, 5H); ^{13}C NMR ($CDCl_3$, 101 MHz): δ_C 162.7 (d, $J_{C–F}$ = 247.2 Hz), 141.7 (d, $J_{C–F}$ = 3.5 Hz), 141.5, 134.2, 133.0, 132.9, 131.6, 129.6, 129.4, 128.2, 127.8, 127.7, 127.1, 125.7, 125.6, 124.8, 124.6, 124.1, 123.9, 122.3, 117.6, 116.9 (d, $J_{C–F}$ = 23.3 Hz), 110.0 (d, $J_{C–F}$ = 23.6 Hz); ^{19}F NMR ($CDCl_3$, 376 MHz): δ_F –111.8; HRMS (ESI-TOF, *m/z*): calcd for $C_{27}H_{18}FN_2$ [M + H]$^+$, 389.1449; found, 389.1471.

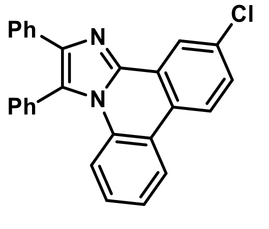

11-Chloro-2,3-diphenylimidazo[1,2-*f*]phenanthridine (3s)

To a 10 mL Schlenk tube equipped with a magnetic stir bar was added 2-(2-bromo-5-chlorophenyl)-4,5-diphenyl-1H-imidazole **1s** (204.8 mg, 0.500 mmol, 1.00 equiv), *o*-bromobenzoic acid **2a** (150.7 mg, 0.750 mmol, 1.50 equiv), DMF (4.0 mL), Cs_2CO_3 (163 mg, 0.500 mmol, 1.00 equiv), PPh_3 (26.2 mg, 0.10 mmol, 0.20 equiv), $Pd(OAc)_2$ (5.6 mg, 0.025 mmol, 0.050 equiv), and CuI (9.5 mg, 0.050 mmol, 0.10 equiv). The reaction mixture was stirred at 110 °C in an oil bath for about 8 h. The resulting mixture

was concentrated and the residue was taken up in ethyl acetate. The organic layer was washed with brine, dried over Na_2SO_4 and concentrated. Purification of the crude product by column chromatography (silica gel; petroleum ether/ethyl acetate 10:1) afforded **3s** in 69% yield (140 mg). White solid; mp 199–201 °C; ^{1}H NMR ($CDCl_3$, 400 MHz): δ_H 8.85 (d, J = 2.4 Hz, 1H), 8.40 (d, J = 8.0 Hz, 1H), 8.30 (d, J = 8.8 Hz, 1H), 7.61–7.53 (m, 8H), 7.42–7.38 (m, 1H), 7.30–7.17 (m, 5H); ^{13}C NMR ($CDCl_3$, 101 MHz): δ_C 141.6, 141.3, 134.6, 134.2, 133.3, 132.8, 131.6, 129.6, 129.4, 128.9, 128.2, 128.1, 127.8, 127.1, 126.0, 125.7, 125.1, 124.8, 124.03, 123.96, 123.8, 122.1, 117.6; HRMS (ESI-TOF, *m/z*): calcd for $C_{27}H_{18}ClN_2$ $[M + H]^+$, 405.1153; found, 405.1173.

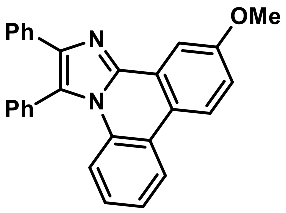

11-Methoxy-2,3-diphenylimidazo[1,2-*f*]phenanthridine (3t)

To a 10 mL Schlenk tube equipped with a magnetic stir bar was added 2-(2-bromo-5-methoxyphenyl)-4,5-diphenyl-1H-imidazole **1t** (202.6 mg, 0.500 mmol, 1.00 equiv), *o*-bromobenzoic acid **2a** (150.7 mg, 0.750 mmol, 1.50 equiv), DMF (4.0 mL), Cs_2CO_3 (163 mg, 0.500 mmol, 1.00 equiv), PPh_3 (26.2 mg, 0.10 mmol, 0.20 equiv), $Pd(OAc)_2$ (5.6 mg, 0.025 mmol, 0.050 equiv), and CuI (9.5 mg, 0.050 mmol, 0.10 equiv). The reaction mixture was stirred at 110 °C in an oil bath for about 8 h. The resulting mixture was concentrated and the residue was taken up in ethyl acetate. The organic layer was washed with brine, dried over Na_2SO_4 and concentrated. Purification of the crude product by column chromatography (silica gel; petroleum ether/ethyl acetate 10:1) afforded **3t** in 68% yield (136 mg). White solid; mp 223–225 °C; ^{1}H NMR ($CDCl_3$, 400 MHz): δ_H 8.35 (d, J = 8.4 Hz, 1H), 8.29–8.26 (m, 2H), 7.60–7.54 (m, 7H), 7.36 (t, J = 7.6 Hz, 1H), 7.28–7.19 (m, 5H), 7.13–7.01 (m, 1H), 4.07 (s, 3H); ^{13}C NMR ($CDCl_3$, 101 MHz): δ_C 159.9, 142.4, 141.2, 134.5, 133.1, 132.4, 131.6, 129.5, 129.3, 128.1, 127.9, 126.9, 126.7, 125.5, 125.2, 124.6, 124.0, 123.5, 122.9, 121.3, 118.8, 117.5, 105.1, 55.8; HRMS (ESI-TOF, *m/z*): calcd for $C_{28}H_{21}N_2O$ $[M + H]^+$, 401.1648; found, 401.1667.

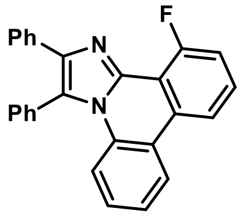

12-Fluoro-2,3-diphenylimidazo[1,2-*f*]phenanthridine (3u)

To a 10 mL Schlenk tube equipped with a magnetic stir bar was added 2-(2-bromo-6-fluorophenyl)-4,5-diphenyl-1H-imidazole **1u** (196.6 mg, 0.500 mmol, 1.00 equiv), *o*-bromobenzoic acid **2a** (150.7 mg, 0.750 mmol, 1.50 equiv), DMF (4.0 mL), Cs_2CO_3 (163 mg, 0.500 mmol, 1.00 equiv), PPh_3 (26.2 mg, 0.10 mmol, 0.20 equiv), $Pd(OAc)_2$ (5.6 mg, 0.025 mmol, 0.050 equiv), and CuI (9.5 mg, 0.050 mmol, 0.10 equiv). The reaction mixture was stirred at 110 °C in an oil bath for about 8 h. The resulting mixture was concentrated and the residue was taken up in ethyl acetate. The organic layer was washed with brine, dried over Na_2SO_4 and concentrated. Purification of the crude product by column chromatography (silica gel; petroleum ether/ethyl acetate 10:1) afforded **3u** in 74% yield (144 mg).

White solid; mp 221–223 °C; ^{1}H NMR ($CDCl_3$, 400 MHz): δ_H 8.43 (d, J = 8.0 Hz, 1H), 8.20 (d, J = 8.4 Hz, 1H), 7.62–7.52 (m, 8H), 7.43–7.37 (m, 2H), 7.31–7.17 (m, 5H); ^{13}C NMR ($CDCl_3$, 101 MHz): δ_C 159.8 (d, $J_{C–F}$ = 258.1 Hz), 146.0, 141.9, 138.3, 136.5, 134.3, 133.5, 133.0, 131.5, 130.5, 129.6, 129.3, 128.7 (d, $J_{C–F}$ = 8.2 Hz), 128.5, 128.1, 127.9, 127.0, 125.0, 124.7 (d, $J_{C–F}$ = 18.9 Hz), 122.0, 117.9 (d, $J_{C–F}$ = 1.8 Hz), 117.7, 115.4 (d, $J_{C–F}$ = 21.3 Hz); ^{19}F NMR ($CDCl_3$, 376 MHz): δ_F –108.8; HRMS (ESI-TOF, *m/z*): calcd for $C_{27}H_{18}FN_2$ $[M + H]^+$, 389.1449; found, 389.1471.

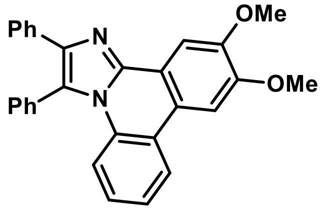

10,11-Dimethoxy-2,3-diphenylimidazo[1,2-f]phenanthridine (3v)

To a 10 mL Schlenk tube equipped with a magnetic stir bar was added 2-(2-bromo-4,5-dimethoxyphenyl)-4,5-diphenyl-1H-imidazole **1v** (217.7 mg, 0.500 mmol, 1.00 equiv), *o*-bromobenzoic acid **2a** (150.7 mg, 0.750 mmol, 1.50 equiv), DMF (4.0 mL), Cs_2CO_3 (163 mg, 0.500 mmol, 1.00 equiv), PPh_3 (26.2 mg, 0.10 mmol, 0.20 equiv), $Pd(OAc)_2$ (5.6 mg, 0.025 mmol, 0.050 equiv), and CuI (9.5 mg, 0.050 mmol, 0.10 equiv). The reaction mixture was stirred at 110 °C in an oil bath for about 8 h. The

resulting mixture was concentrated and the residue was taken up in ethyl acetate. The organic layer was washed with brine, dried over Na_2SO_4 and concentrated. Purification of the crude product by column chromatography (silica gel; petroleum ether/ethyl acetate 10:1) afforded **3v** in 76% yield (164 mg).

White solid; mp 231–232 °C; ^{1}H NMR ($CDCl_3$, 400 MHz): δ_H 8.31 (d, J = 8.0 Hz, 1H), 8.25 (s, 1H), 7.72 (s, 1H), 7.60–7.54 (m, 7H), 7.37 (t, J = 7.6 Hz, 1H), 7.31–7.18 (m, 4H), 7.15–7.11 (m, 1H), 4.16 (s, 3H), 4.11 (s, 3H); ^{13}C NMR ($CDCl_3$, 101 MHz): δ_C 150.6, 150.4, 142.4, 141.0, 134.5, 133.2, 132.8, 131.7, 129.5, 129.2, 128.1, 127.9, 126.9, 126.8, 124.9, 124.3, 123.4, 122.6, 121.8, 118.2, 117.6, 105.3, 103.4, 56.4, 56.0; HRMS (ESI-TOF, *m/z*): calcd for $C_{29}H_{23}N_2O_2$ $[M + H]^+$, 431.1754; found, 431.1769.

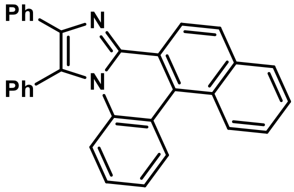

1,2-Diphenylbenzo[*k*]imidazo[1,2-*f*]phenanthridine (3w)

To a 10 mL Schlenk tube equipped with a magnetic stir bar was added 2-(1-bromonaphthalen-2-yl)-4,5-diphenyl-1H-imidazole **1w** (212.7 mg, 0.500 mmol, 1.00 equiv), *o*-bromobenzoic acid **2a** (150.7 mg, 0.750 mmol, 1.50 equiv), DMF (4.0 mL), Cs_2CO_3 (163 mg, 0.500 mmol, 1.00 equiv), PPh_3 (26.2 mg, 0.10 mmol, 0.20 equiv), $Pd(OAc)_2$ (5.6 mg, 0.025 mmol, 0.050 equiv), and CuI (9.5 mg, 0.050 mmol, 0.10 equiv). The reaction mixture was stirred at 110 °C in an oil bath for about 8 h. The resulting mixture was concentrated and the residue was taken up in ethyl acetate. The organic layer was washed with brine, dried over Na_2SO_4 and concentrated. Purification of the crude product by column chromatography (silica gel; petroleum ether/ethyl acetate 10:1) afforded **3w** in 77% yield (162 mg).

White solid; mp 243–245 °C; ^{1}H NMR ($CDCl_3$, 400 MHz): δ_H 8.89 (d, J = 8.4 Hz, 1H), 8.83 (dd, J = 8.8, 4.8 Hz, 2H), 8.03 (dd, J = 8.4, 5.6 Hz, 2H), 7.70–7.57 (m, 9H), 7.44–7.38 (m, 2H), 7.31–7.20 (m, 4H); ^{13}C NMR ($CDCl_3$, 101 MHz): δ_C 143.5, 134.5, 133.4, 132.6, 131.2, 129.7, 129.5, 129.4, 129.2, 129.1, 129.0, 128.6, 128.2, 128.1, 127.3, 127.1, 127.0, 126.8, 126.2, 125.2, 125.0, 124.04, 124.00, 123.3, 122.7, 121.6, 118.0; HRMS (ESI-TOF, *m/z*): calcd for $C_{31}H_{21}N_2$ $[M + H]^+$, 421.1699; found, 421.1715.

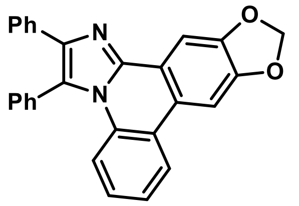

2,3-Diphenyl-[1,3]dioxolo[4,5-*j*]imidazo[1,2-*f*]phenanthridine (3x)

To a 10 mL Schlenk tube equipped with a magnetic stir bar was added 2-(6-bromobenzo[d][1,3]dioxol-5-yl)-4,5-diphenyl-1H-imidazole **1x** (209.6 mg, 0.500 mmol, 1.00 equiv), *o*-bromobenzoic acid **2a** (150.7 mg, 0.750 mmol, 1.50 equiv), DMF (4.0 mL), Cs_2CO_3 (163 mg, 0.500 mmol, 1.00 equiv), PPh_3 (26.2 mg, 0.10 mmol, 0.20 equiv), $Pd(OAc)_2$ (5.6 mg, 0.025 mmol, 0.050 equiv), and CuI (9.5 mg, 0.050 mmol, 0.10 equiv). The reaction mixture was stirred at 110 °C in an oil bath for about 8 h. The resulting mixture was concentrated and the residue was taken up in ethyl acetate. The organic layer was washed with brine, dried over Na_2SO_4 and concentrated. Purification of the crude product by column chromatography (silica gel; petroleum ether/ethyl acetate 10:1) afforded **3x** in 75% yield (155 mg).

White solid; mp 269–271 °C; ^{1}H NMR ($CDCl_3$, 400 MHz): δ_H 8.25–8.23 (m, 2H), 7.73 (s, 1H), 7.59–7.54 (m, 7H), 7.35 (t, *J* = 7.6 Hz, 1H), 7.29–7.18 (m, 4H), 7.14–7.10 (m, 1H), 6.14 (s, 2H); ^{13}C NMR ($CDCl_3$, 101 MHz): δ_C 149.5, 148.7, 142.6, 141.0, 134.5, 133.2, 132.8, 131.7, 129.5, 129.2, 128.1, 127.8, 127.0, 126.9, 124.9, 124.4, 123.7, 123.5, 122.7, 119.7, 117.5, 103.2, 101.7, 101.1; HRMS (ESI-TOF, *m/z*): calcd for $C_{28}H_{19}N_2O_2$ $[M + H]^+$, 415.1441; found, 415.1451.

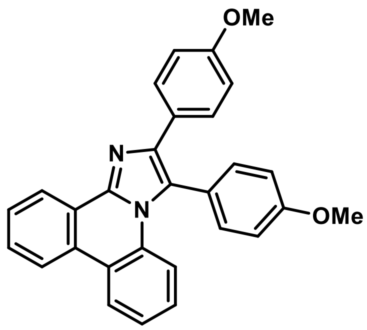

2,3-Bis(4-methoxyphenyl)imidazo[1,2-*f*]phenanthridine (3y)

To a 10 mL Schlenk tube equipped with a magnetic stir bar was added 2-(2-bromophenyl)-4,5-bis(4-methoxyphenyl)-1H-imidazole **1y** (217.7 mg, 0.500 mmol, 1.00 equiv), *o*-bromobenzoic acid **2a** (150.7 mg, 0.750 mmol, 1.50 equiv), DMF (4.0 mL), Cs_2CO_3 (163 mg, 0.500 mmol, 1.00 equiv), PPh_3 (26.2 mg, 0.10 mmol, 0.20 equiv), $Pd(OAc)_2$ (5.6 mg, 0.025 mmol, 0.050 equiv), and CuI (9.5 mg, 0.050 mmol,

0.10 equiv). The reaction mixture was stirred at 110 °C in an oil bath for about 8 h. The resulting mixture was concentrated and the residue was taken up in ethyl acetate. The organic layer was washed with brine, dried over Na_2SO_4 and concentrated. Purification of the crude product by column chromatography (silica gel; petroleum ether/ethyl acetate 10:1) afforded **3y** in 65% yield (140 mg).

White solid; mp 203–204 °C; ^{1}H NMR ($CDCl_3$, 400 MHz): δ_H 8.86–8.83 (m, 1H), 8.45 (d, J = 8.4 Hz, 1H), 8.38–8.36 (m, 1H), 7.68–7.62 (m, 2H), 7.55 (d, J = 8.4 Hz, 2H), 7.44 (d, J = 8.4 Hz, 2H), 7.40–7.37 (m, 2H), 7.22–7.18 (m, 1H), 7.09 (d, J = 8.4 Hz, 2H), 6.82 (d, J = 8.4 Hz, 2H), 3.95 (s, 3H), 3.79 (s, 3H); ^{13}C NMR ($CDCl_3$, 101 MHz): δ_C 160.1, 158.6, 142.2, 141.1, 133.6, 132.9, 128.9, 128.42, 128.37, 127.8, 127.5, 127.3, 125.2, 124.5, 124.3, 124.27, 124.0, 123.9, 122.7, 122.0, 117.4, 115.0, 113.6, 55.4, 55.2; HRMS (ESI-TOF, *m/z*): calcd for $C_{29}H_{23}N_2O_2$ $[M + H]^+$, 431.1754; found, 431.1769.

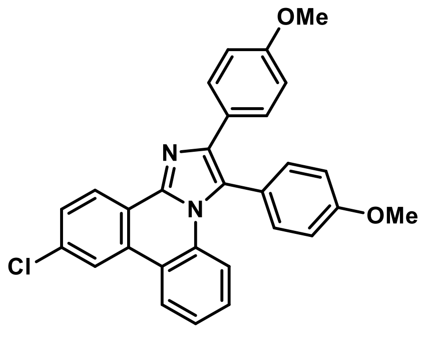

10-Chloro-2,3-bis(4-methoxyphenyl)imidazo[1,2-*f*]phenanthridine (3z)

To a 10 mL Schlenk tube equipped with a magnetic stir bar was added 2-(2-bromo-4-chlorophenyl)-4,5-bis(4-methoxyphenyl)-1H-imidazole **1z** (234.9 mg, 0.500 mmol, 1.00 equiv), *o*-bromobenzoic acid **2a** (150.7 mg, 0.750 mmol, 1.50 equiv), DMF (4.0 mL), Cs_2CO_3 (163 mg, 0.500 mmol, 1.00 equiv), PPh_3 (26.2 mg, 0.10 mmol, 0.20 equiv), $Pd(OAc)_2$ (5.6 mg, 0.025 mmol, 0.050 equiv), and CuI (9.5 mg, 0.050 mmol, 0.10 equiv). The reaction mixture was stirred at 110 °C in an oil bath for about 8 h. The resulting mixture was concentrated and the residue was taken up in ethyl acetate. The organic layer was washed with brine, dried over Na_2SO_4 and concentrated. Purification of the crude product by column chromatography (silica gel; petroleum ether/ethyl acetate 10:1) afforded **3z** in 67% yield (156 mg).

White solid; mp 186–187 °C; ^{1}H NMR ($CDCl_3$, 400 MHz): δ_H 8.76 (d, J = 8.8 Hz, 1H), 8.36–8.31 (m, 2H), 7.60 (d, J = 8.8 Hz, 1H), 7.53 (d, J = 8.8 Hz, 2H), 7.44–7.36 (m, 4H), 7.24–7.20 (m, 1H), 7.09 (d, J = 8.4 Hz, 2H), 6.81 (d, J = 8.8 Hz, 2H), 3.95 (s, 3H), 3.79 (s, 3H); ^{13}C NMR ($CDCl_3$, 101 MHz): δ_C 160.2, 158.7, 141.5, 141.4, 134.6, 133.9, 132.8, 128.9, 128.7, 128.5, 127.1, 126.1, 125.0, 124.5, 124.4, 124.1, 122.3,

122.0, 121.6, 117.5, 115.1, 113.6, 55.4, 55.2; HRMS (ESI-TOF, *m/z*): calcd for $C_{29}H_{22}ClN_2O_2$ [M + H]$^+$, 465.1364; found, 465.1366.

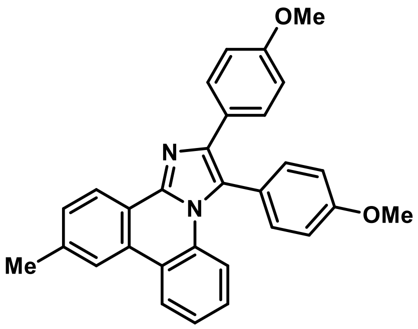

2,3-Bis(4-methoxyphenyl)-10-methylimidazo[1,2-*f*]phenanthridine (3aa)

To a 10 mL Schlenk tube equipped with a magnetic stir bar was added 2-(2-bromo-4-methylphenyl)-4,5-bis(4-methoxyphenyl)-1H-imidazole **1aa** (224.7 mg, 0.500 mmol, 1.00 equiv), *o*-bromobenzoic acid **2a** (150.7 mg, 0.750 mmol, 1.50 equiv), DMF (4.0 mL), Cs_2CO_3 (163 mg, 0.500 mmol, 1.00 equiv), PPh_3 (26.2 mg, 0.10 mmol, 0.20 equiv), $Pd(OAc)_2$ (5.6 mg, 0.025 mmol, 0.050 equiv), and CuI (9.5 mg, 0.050 mmol, 0.10 equiv). The reaction mixture was stirred at 110 °C in an oil bath for about 8 h. The resulting mixture was concentrated and the residue was taken up in ethyl acetate. The organic layer was washed with brine, dried over Na_2SO_4 and concentrated. Purification of the crude product by column chromatography (silica gel; petroleum ether/ethyl acetate 10:1) afforded **3aa** in 69% yield (153 mg). White solid; mp 133–134 °C; ^{1}H NMR ($CDCl_3$, 400 MHz): δ_H 8.73 (d, *J* = 8.0 Hz, 1H), 8.45 (d, *J* = 8.0 Hz, 1H), 8.16 (s, 1H), 7.55 (d, *J* = 8.4 Hz, 2H), 7.49 (d, *J* = 8.4 Hz, 1H), 7.44 (d, *J* = 8.0 Hz, 2H), 7.39–7.35 (m, 2H), 7.19 (d, *J* = 8.0 Hz, 1H), 7.09 (d, *J* = 8.4 Hz, 2H), 6.81 (d, *J* = 8.4 Hz, 2H), 3.95 (s, 3H), 3.79 (s, 3H), 2.59 (s, 3H); ^{13}C NMR ($CDCl_3$, 101 MHz): δC 160.1, 158.5, 142.4, 140.9, 138.4, 133.7, 132.9, 129.8, 128.9, 127.6, 127.58, 127.4, 125.3, 124.5, 124.2, 124.0, 123.9, 122.6, 122.1, 121.6, 117.4, 115.0, 113.5, 55.3, 55.2, 22.1; HRMS (ESI-TOF, *m/z*): calcd for $C_{30}H_{25}N_2O_2$ [M + H]$^+$, 445.1911; found, 445.1910.

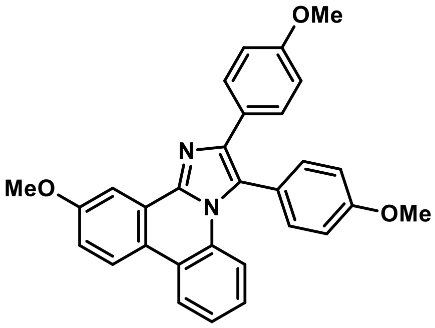

11-Methoxy-2,3-bis(4-methoxyphenyl)imidazo[1,2-*f*]phenanthridine (3ab)

To a 10 mL Schlenk tube equipped with a magnetic stir bar was added 2-(2-bromo-4-methoxyphenyl)-4,5-bis(4-methoxyphenyl)-1H-imidazole **1ab** (232.7 mg, 0.500 mmol, 1.00 equiv), *o*-bromobenzoic acid **2a** (150.7 mg, 0.750 mmol, 1.50 equiv), DMF (4.0 mL), Cs_2CO_3 (163 mg, 0.500 mmol, 1.00 equiv), PPh_3 (26.2 mg, 0.10 mmol, 0.20 equiv), $Pd(OAc)_2$ (5.6 mg, 0.025 mmol, 0.050 equiv), and CuI (9.5 mg, 0.050 mmol, 0.10 equiv). The reaction mixture was stirred at 110 °C in an oil bath for about 8 h. The resulting mixture was concentrated and the residue was taken up in ethyl acetate. The organic layer was washed with brine, dried over Na_2SO_4 and concentrated. Purification of the crude product by column chromatography (silica gel; petroleum ether/ethyl acetate 10:1) afforded **3ab** in 73% yield (168 mg).

White solid; mp 168–170 °C; ^{1}H NMR ($CDCl_3$, 400 MHz): δ_H 8.33 (d, *J* = 8.4 Hz, 1H), 8.26–8.24 (m, 2H), 7.56 (d, *J* = 8.4 Hz, 2H), 7.44 (d, *J* = 8.4 Hz, 2H), 7.36–7.33 (m, 2H), 7.26–7.21 (m, 1H), 7.15–7.07 (m, 3H), 6.84–6.80 (m, 2H), 4.05 (s, 3H), 3.94 (s, 3H), 3.79 (s, 3H); ^{13}C NMR ($CDCl_3$, 101 MHz): δ_C 160.1, 159.8, 158.6, 142.0, 141.1, 132.9, 132.6, 129.0, 127.2, 126.7, 125.2, 125.16, 124.5, 124.4, 123.9, 123.4, 122.8, 121.2, 118.6, 117.4, 115.0, 113.6, 105.0, 55.8, 55.4, 55.2; HRMS (ESI-TOF, *m/z*): calcd for $C_{30}H_{25}N_2O_3$ $[M + H]^+$, 461.1860; found, 461.1875.

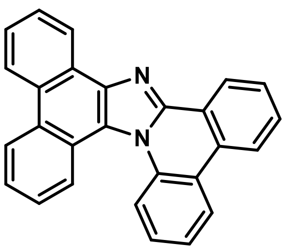

Phenanthro[9',10':4,5]imidazo[1,2-*f*]phenanthridine (3ac)[3]

To a 10 mL Schlenk tube equipped with a magnetic stir bar was added 2-(2-bromophenyl)-1H-phenanthro[9,10-d]imidazole **1ac** (186.7 mg, 0.500 mmol, 1.00 equiv), *o*-bromobenzoic acid **2a** (150.7

mg, 0.750 mmol, 1.50 equiv), DMF (4.0 mL), Cs_2CO_3 (163 mg, 0.500 mmol, 1.00 equiv), PPh_3 (26.2 mg, 0.10 mmol, 0.20 equiv), $Pd(OAc)_2$ (5.6 mg, 0.025 mmol, 0.050 equiv), and CuI (9.5 mg, 0.050 mmol, 0.10 equiv). The reaction mixture was stirred at 110 °C in an oil bath for about 8 h. The resulting mixture was concentrated and the residue was taken up in ethyl acetate. The organic layer was washed with brine, dried over Na_2SO_4 and concentrated. Purification of the crude product by column chromatography (silica gel; petroleum ether/ethyl acetate 10:1) afforded **3ac** in 82% yield (151 mg).

White solid; mp 293–294 °C; ^{1}H NMR ($CDCl_3$, 400 MHz): δ_H 8.97–8.95 (m, 2H), 8.82 (d, *J* = 8.0 Hz, 1H), 8.75 (d, *J* = 8.4 Hz, 1H), 8.50–8.47 (m, 2H), 8.45–8.42 (m, 2H), 7.81–7.77 (m, 1H), 7.74–7.72 (m, 3H), 7.66–7.56 (m, 4H); ^{13}C NMR ($CDCl_3$, 101 MHz): δ_C 147.7, 141.4, 133.5, 129.6, 129.59, 129.3, 129.0, 128.7, 127.4, 127.2, 127.1, 126.4, 125.5, 125.4, 125.2, 125.1, 124.7, 124.6, 124.1, 124.0, 123.8, 123.3, 123.12, 123.08, 123.0, 122.3, 119.5; HRMS (ESI-TOF, *m/z*): calcd for $C_{27}H_{17}N_2$ [M + H]$^+$, 369.1386; found, 369.1407.

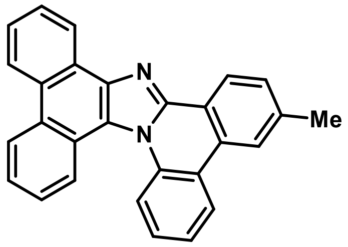

6-Methylphenanthro[9',10':4,5]imidazo[1,2-*f*]phenanthridine (3ad)

To a 10 mL Schlenk tube equipped with a magnetic stir bar was added 2-(2-bromo-4-methylphenyl)-1H-phenanthro[9,10-d]imidazole **1ad** (193.6 mg, 0.500 mmol, 1.00 equiv), *o*-bromobenzoic acid **2a** (150.7 mg, 0.750 mmol, 1.50 equiv), DMF (4.0 mL), Cs_2CO_3 (163 mg, 0.500 mmol, 1.00 equiv), PPh_3 (26.2 mg, 0.10 mmol, 0.20 equiv), $Pd(OAc)_2$ (5.6 mg, 0.025 mmol, 0.050 equiv), and CuI (9.5 mg, 0.050 mmol, 0.10 equiv). The reaction mixture was stirred at 110 °C in an oil bath for about 8 h. The resulting mixture was concentrated and the residue was taken up in ethyl acetate. The organic layer was washed with brine, dried over Na_2SO_4 and concentrated. Purification of the crude product by column chromatography (silica gel; petroleum ether/ethyl acetate 10:1) afforded **3ad** in 85% yield (163 mg).

White solid; mp 241–243 °C; ^{1}H NMR ($CDCl_3$, 400 MHz): δ_H 8.93 (d, *J* = 7.6 Hz, 1H), 8.81–8.78 (m, 2H), 8.74 (d, *J* = 8.4 Hz, 1H), 8.47–8.43 (m, 2H), 8.41–8.38 (m, 1H), 8.19 (s, 1H), 7.80–7.76 (m, 1H), 7.73–7.69 (m, 1H), 7.64–7.51 (m, 5H), 2.61 (s, 3H); ^{13}C NMR ($CDCl_3$, 101 MHz): δ_C 148.0, 141.4, 139.7, 133.6, 130.0, 129.6, 129.3, 128.8, 127.3, 127.1, 127.0, 126.3, 125.4, 125.3, 125.0, 124.9, 124.6, 124.5, 123.8,

123.3, 123.04, 123.0, 122.3, 121.8, 119.4, 22.2; HRMS (ESI-TOF, *m/z*): calcd for $C_{28}H_{19}N_2$ $[M + H]^+$, 383.1543; found, 383.1563.

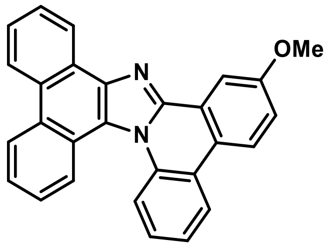

7-Methoxyphenanthro[9',10':4,5]imidazo[1,2-*f*]phenanthridine (3ae)

To a 10 mL Schlenk tube equipped with a magnetic stir bar was added 2-(2-bromo-4-methoxyphenyl)-1H-phenanthro[9,10-d]imidazole **1ae** (201.6 mg, 0.500 mmol, 1.00 equiv), *o*-bromobenzoic acid **2a** (150.7 mg, 0.750 mmol, 1.50 equiv), DMF (4.0 mL), Cs_2CO_3 (163 mg, 0.500 mmol, 1.00 equiv), PPh_3 (26.2 mg, 0.10 mmol, 0.20 equiv), $Pd(OAc)_2$ (5.6 mg, 0.025 mmol, 0.050 equiv), and CuI (9.5 mg, 0.050 mmol, 0.10 equiv). The reaction mixture was stirred at 110 °C in an oil bath for about 8 h. The resulting mixture was concentrated and the residue was taken up in ethyl acetate. The organic layer was washed with brine, dried over Na_2SO_4 and concentrated. Purification of the crude product by column chromatography (silica gel; petroleum ether/ethyl acetate 10:1) afforded **3ae** in 79% yield (157 mg). White solid; mp 239–241 °C; ^1H NMR ($CDCl_3$, 400 MHz): δ_H 8.96 (d, *J* = 8.0 Hz, 1H), 8.82 (d, *J* = 8.4 Hz, 1H), 8.75 (d, *J* = 8.0 Hz, 1H), 8.50 (d, *J* = 8.0 Hz, 1H), 8.43–8.38 (m, 2H), 8.35–8.32 (m, 2H), 7.81–7.77 (m, 1H), 7.74–7.70 (m, 1H), 7.66–7.58 (m, 2H), 7.57–7.51 (m, 2H), 7.31 (dd, *J* = 8.8 Hz, *J'* = 2.4 Hz, 1H), 4.11 (s, 3H); ^{13}C NMR ($CDCl_3$, 101 MHz): δ_C 160.0, 147.6, 141.3, 132.5, 129.7, 129.0, 127.4, 127.1, 126.4, 126.1, 125.4, 125.36, 125.2, 125.1, 124.5, 124.1, 124.06, 124.0, 123.8, 123.3, 123.2, 123.1, 123.0, 119.5, 119.4, 106.1, 55.9; HRMS (ESI-TOF, *m/z*): calcd for $C_{28}H_{19}N_2O$ $[M + H]^+$, 399.1492; found, 399.1492.

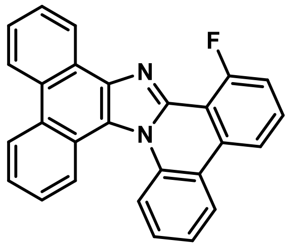

8-Fluorophenanthro[9',10':4,5]imidazo[1,2-*f*]phenanthridine (3af)

To a 10 mL Schlenk tube equipped with a magnetic stir bar was added 2-(2-bromo-6-fluorophenyl)-1H-phenanthro[9,10-d]imidazole **1af** (195.6 mg, 0.500 mmol, 1.00 equiv), *o*-bromobenzoic acid **2a** (150.7 mg, 0.750 mmol, 1.50 equiv), DMF (4.0 mL), Cs_2CO_3 (163 mg, 0.500 mmol, 1.00 equiv), PPh_3 (26.2 mg, 0.10 mmol, 0.20 equiv), $Pd(OAc)_2$ (5.6 mg, 0.025 mmol, 0.050 equiv), and CuI (9.5 mg, 0.050 mmol, 0.10 equiv). The reaction mixture was stirred at 110 °C in an oil bath for about 8 h. The resulting mixture was concentrated and the residue was taken up in ethyl acetate. The organic layer was washed with brine, dried over Na_2SO_4 and concentrated. Purification of the crude product by column chromatography (silica gel; petroleum ether/ethyl acetate 10:1) afforded **3af** in 74% yield (143 mg).

White solid; mp 284–286 °C; ^{1}H NMR ($CDCl_3$, 400 MHz): δ_H 8.99 (d, *J* = 8.0 Hz, 1H), 8.82 (d, *J* = 8.0 Hz, 1H), 8.74 (d, *J* = 8.0 Hz, 1H), 8.45–8.38 (m, 3H), 8.25 (d, *J* = 8.4 Hz, 1H), 7.81–7.77 (m, 1H), 7.75–7.67 (m, 2H), 7.66–7.63 (m, 1H), 7.61–7.53 (m, 3H), 7.48–7.43 (m, 1H); ^{13}C NMR ($CDCl_3$, 101 MHz): δ_C 160.0 (d, $J_{C–F}$ = 258.8 Hz), 144.2 (d, $J_{C–F}$ = 8.0 Hz), 141.8 (d, $J_{C–F}$ = 2.3 Hz), 133.6, 131.9 (d, $J_{C–F}$ = 1.5 Hz), 129.9 (d, $J_{C–F}$ = 8.7 Hz), 129.6, 129.3, 127.9, 127.4, 127.0, 126.6, 125.41, 125.36, 125.2, 124.6, 123.6, 123.5, 123.4, 123.3, 122.9, 122.3 (d, $J_{C–F}$ = 1.3 Hz), 119.5, 118.1 (d, $J_{C–F}$ = 3.6 Hz), 115.6 (d, $J_{C–F}$ = 20.6 Hz), 113.5, 113.4; ^{19}F NMR ($CDCl_3$, 376 MHz): δ_F –108.4; HRMS (ESI-TOF, *m/z*): calcd for $C_{27}H_{16}FN_2$ $[M + H]^+$, 387.1292; found, 387.1313.

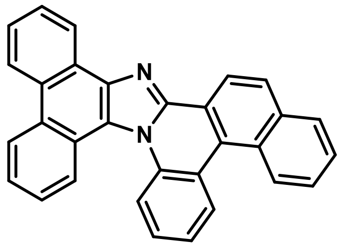

Benzo[*k*]phenanthro[9',10':4,5]imidazo[1,2-*f*]phenanthridine (3ag)

To a 10 mL Schlenk tube equipped with a magnetic stir bar was added 2-(1-bromonaphthalen-2-yl)-1*H*-phenanthro[9,10-*d*]imidazole **1ag** (211.5 mg, 0.500 mmol, 1.00 equiv), *o*-bromobenzoic acid **2a** (150.7 mg, 0.750 mmol, 1.50 equiv), DMF (4.0 mL), Cs_2CO_3 (163 mg, 0.500 mmol, 1.00 equiv), PPh_3

(26.2 mg, 0.10 mmol, 0.20 equiv), $Pd(OAc)_2$ (5.6 mg, 0.025 mmol, 0.050 equiv), and CuI (9.5 mg, 0.050 mmol, 0.10 equiv). The reaction mixture was stirred at 110 °C in an oil bath for about 8 h. The resulting mixture was concentrated and the residue was taken up in ethyl acetate. The organic layer was washed with brine, dried over Na_2SO_4 and concentrated. Purification of the crude product by column chromatography (silica gel; petroleum ether/ethyl acetate 10:1) afforded **3ag** in 75% yield (157 mg).

White solid; mp 216–217 °C; ^{1}H NMR ($CDCl_3$, 400 MHz): δ_H 9.90–8.96 (m, 2H), 8.91 (d, *J* = 8.4 Hz, 1H), 8.84 (d, *J* = 7.6 Hz, 2H), 8.77 (d, *J* = 8.4 Hz, 1H), 8.61–8.59 (m, 1H), 8.48–8.45 (m, 1H), 8.09 (d, *J* = 8.4 Hz, 1H), 8.06 (d, *J* = 8.0 Hz, 1H), 7.82–7.79 (m, 1H), 7.75–7.72 (m, 2H), 7.68–7.64 (m, 3H), 7.62–7.54 (m, 2H); ^{13}C NMR ($CDCl_3$, 101 MHz): δ_C 148.6, 141.9, 134.8, 133.6, 130.0, 129.6, 129.4, 129.1, 129.0, 128.9, 127.4, 127.3, 127.2, 127.1, 127.0, 126.7, 126.5, 125.7, 125.2, 125.1, 124.5, 123.7, 123.6, 123.4, 123.1, 123.08, 122.9, 121.8, 119.3; HRMS (ESI-TOF, *m/z*): calcd for $C_{31}H_{19}N_2$ $[M + H]^+$, 419.1543; found, 419.1524.

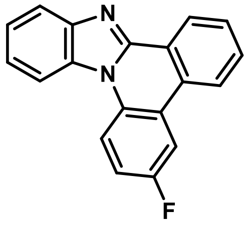

3-Fluorobenzo[4,5]imidazo[1,2-*f*]phenanthridine (4a)[6]

To a 10 mL Schlenk tube equipped with a magnetic stir bar was added 2-(2-bromophenyl)-1*H*-benzo[*d*]imidazole **1a** (136.5 mg, 0.500 mmol, 1.00 equiv), 2-bromo-4-fluorobenzoic acid **2b** (164.3 mg, 0.750 mmol, 1.50 equiv), DMF (4.0 mL), Cs_2CO_3 (163 mg, 0.500 mmol, 1.00 equiv), PPh_3 (26.2 mg, 0.10 mmol, 0.20 equiv), $Pd(OAc)_2$ (5.6 mg, 0.025 mmol, 0.050 equiv), and CuI (9.5 mg, 0.050 mmol, 0.10 equiv). The reaction mixture was stirred at 110 °C in an oil bath for about 8 h. The resulting mixture was concentrated and the residue was taken up in ethyl acetate. The organic layer was washed with brine, dried over Na_2SO_4 and concentrated. Purification of the crude product by column chromatography (silica gel; petroleum ether/ethyl acetate 10:1) afforded **4a** in 73% yield (104 mg).

White solid; mp 196–198 °C; ^{1}H NMR ($CDCl_3$, 400 MHz): δ_H 8.85 (d, *J* = 8.4 Hz, 1H), 8.45 (dd, *J* = 9.2, 6.0 Hz, 1H), 8.31–8.23 (m, 3H), 8.05 (d, *J* = 7.2 Hz, 1H), 7.76–7.65 (m, 2H), 7.56–7.48 (m, 2H), 7.26–7.22 (m, 1H); ^{13}C NMR ($CDCl_3$, 101 MHz): δ_C 162.8 (d, $J_{C–F}$ = 247.2 Hz), 147.6, 144.5, 135.2 (d, $J_{C–F}$ = 10.1 Hz), 131.6, 130.6, 129.0, 128.5, 126.1, 126.0, 124.5, 123.2, 122.8, 122.1, 120.5, 118.1, 113.5, 112.0 (d, $J_{C–F}$

= 22.0 Hz), 103.3 (d, $J_{C–F}$ = 26.9 Hz); ^{19}F NMR (CDCl$_3$, 376 MHz): δ_F –109.4; HRMS (ESI-TOF, *m/z*): calcd for $C_{19}H_{12}FN_2$ [M + H]$^+$, 287.0979; found, 287.0993.

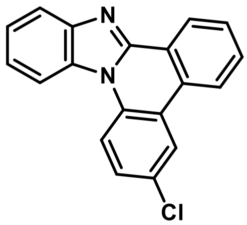

3-Chlorobenzo[4,5]imidazo[1,2-*f*]phenanthridine (4b)[6]

To a 10 mL Schlenk tube equipped with a magnetic stir bar was added 2-(2-bromophenyl)-1*H*-benzo[*d*]imidazole **1a** (136.5 mg, 0.500 mmol, 1.00 equiv), 2-bromo-4-chlorobenzoic acid **2c** (176.3 mg, 0.750 mmol, 1.50 equiv), DMF (4.0 mL), Cs_2CO_3 (163 mg, 0.500 mmol, 1.00 equiv), PPh_3 (26.2 mg, 0.10 mmol, 0.20 equiv), Pd(OAc)$_2$ (5.6 mg, 0.025 mmol, 0.050 equiv), and CuI (9.5 mg, 0.050 mmol, 0.10 equiv). The reaction mixture was stirred at 110 °C in an oil bath for about 8 h. The resulting mixture was concentrated and the residue was taken up in ethyl acetate. The organic layer was washed with brine, dried over Na_2SO_4 and concentrated. Purification of the crude product by column chromatography (silica gel; petroleum ether/ethyl acetate 10:1) afforded **4b** in 64% yield (97 mg).

White solid; mp 205–207 °C; ^{1}H NMR (CDCl$_3$, 400 MHz): δ_H 8.88 (dd, *J* = 7.6, 2.0 Hz, 1H), 8.51 (d, *J* = 8.8 Hz, 1H), 8.44 (d, *J* = 2.4 Hz, 1H), 8.34–8.27 (m, 2H), 8.06 (dd, *J* = 8.0, 1.6 Hz, 1H), 7.79–7.70 (m, 2H), 7.66 (dd, *J* = 9.2, 2.4 Hz, 1H), 7.56–7.47 (m, 2H); ^{13}C NMR (CDCl$_3$, 101 MHz): δ_C 147.2, 144.5, 132.9, 131.7, 130.6, 130.1, 129.3, 129.0, 128.4, 126.1, 124.4, 124.1, 123.7, 123.3, 123.2, 122.4, 120.6, 117.2, 113.6; HRMS (ESI-TOF, *m/z*): calcd for $C_{19}H_{12}ClN_2$ [M + H]$^+$, 303.0684; found, 303.0691.

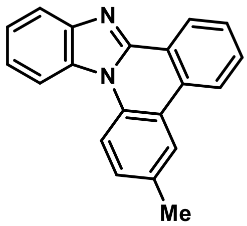

3-Methylbenzo[4,5]imidazo[1,2-*f*]phenanthridine (4c)[3]

To a 10 mL Schlenk tube equipped with a magnetic stir bar was added 2-(2-bromophenyl)-1*H*-benzo[*d*]imidazole **1a** (136.5 mg, 0.500 mmol, 1.00 equiv), 2-bromo-4-methylbenzoic acid **2d** (161.3 mg, 0.750 mmol, 1.00 equiv), DMF (4.0 mL), Cs_2CO_3 (163 mg, 0.500 mmol, 1.00 equiv), PPh_3 (26.2 mg,

0.10 mmol, 0.20 equiv), $Pd(OAc)_2$ (5.6 mg, 0.025 mmol, 0.050 equiv), and CuI (9.5 mg, 0.050 mmol, 0.10 equiv). The reaction mixture was stirred at 110 °C in an oil bath for about 8 h. The resulting mixture was concentrated and the residue was taken up in ethyl acetate. The organic layer was washed with brine, dried over Na_2SO_4 and concentrated. Purification of the crude product by column chromatography (silica gel; petroleum ether/ethyl acetate 10:1) afforded **4c** in 69% yield (97 mg).

White solid; mp 201–202 °C; ^{1}H NMR ($CDCl_3$, 400 MHz): δ_H 8.86 (d, *J* = 8.0 Hz, 1H), 8.35–8.31 (m, 4H), 8.05 (d, *J* = 7.6 Hz, 1H), 7.73–7.63 (m, 2H), 7.54–7.46 (m, 2H), 7.30 (d, *J* = 7.6 Hz, 1H), 2.61 (s, 3H); ^{13}C NMR ($CDCl_3$, 101 MHz): δ_C 147.7, 144.6, 139.6, 134.5, 131.9, 130.4, 129.7, 128.1, 126.0, 125.6, 124.0, 123.1, 122.8, 122.0, 120.3, 119.2, 116.3, 114.0, 22.0; HRMS (ESI-TOF, *m/z*): calcd for $C_{20}H_{15}N_2$ $[M + H]^+$, 283.1230; found, 283.1242.

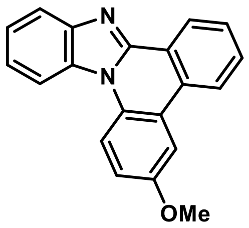

3-Methoxybenzo[4,5]imidazo[1,2-*f*]phenanthridine (4d)[3]

To a 10 mL Schlenk tube equipped with a magnetic stir bar was added 2-(2-bromophenyl)-1*H*-benzo[*d*]imidazole **1a** (136.5 mg, 0.500 mmol, 1.00 equiv), 2-bromo-4-methoxybenzoic acid **2e** (173.3 mg, 0.750 mmol, 1.50 equiv), DMF (4.0 mL), Cs_2CO_3 (163 mg, 0.500 mmol, 1.00 equiv), PPh_3 (26.2 mg, 0.10 mmol, 0.20 equiv), $Pd(OAc)_2$ (5.6 mg, 0.025 mmol, 0.050 equiv), and CuI (9.5 mg, 0.050 mmol, 0.10 equiv). The reaction mixture was stirred at 110 °C in an oil bath for about 8 h. The resulting mixture was concentrated and the residue was taken up in ethyl acetate. The organic layer was washed with brine, dried over Na_2SO_4 and concentrated. Purification of the crude product by column chromatography (silica gel; petroleum ether/ethyl acetate 10:1) afforded **4d** in 70% yield (104 mg).

White solid; mp 180–182 °C; ^{1}H NMR ($CDCl_3$, 400 MHz): δ_H 8.83 (d, *J* = 7.6 Hz, 1H), 8.39 (d, *J* = 9.2 Hz, 1H), 8.23 (d, *J* = 8.0 Hz, 2H), 8.03 (d, *J* = 8.0 Hz, 1H), 7.81 (d, *J* = 2.8 Hz, 1H), 7.70–7.64 (m, 2H), 7.51–7.42 (m, 2H), 7.19 (dd, *J* = 8.8, 2.8 Hz, 1H), 3.94 (s, 3H); ^{13}C NMR ($CDCl_3$, 101 MHz): δ_C 156.2, 147.0, 144.3, 131.7, 130.3, 129.2, 128.72, 128.66, 126.1, 123.9, 123.0, 122.7, 122.3, 120.3, 117.1, 115.8, 113.6, 108.0, 55.7; HRMS (ESI-TOF, *m/z*): calcd for $C_{20}H_{15}N_2O$ $[M + H]^+$, 299.1179; found, 299.1190.

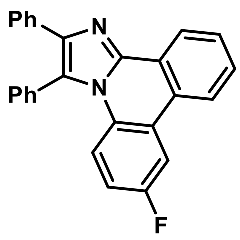

7-Fluoro-2,3-diphenylimidazo[1,2-*f*]phenanthridine (4e)[7]

To a 10 mL Schlenk tube equipped with a magnetic stir bar was added 2-(2-bromophenyl)-4,5-diphenyl-1*H*-imidazole **1n** (187.5 mg, 0.500 mmol, 1.00 equiv), 2-bromo-4-fluorobenzoic acid **2b** (164.3 mg, 0.750 mmol, 1.50 equiv), DMF (4.0 mL), Cs_2CO_3 (163 mg, 0.500 mmol, 1.00 equiv), PPh_3 (26.2 mg, 0.10 mmol, 0.20 equiv), $Pd(OAc)_2$ (5.6 mg, 0.025 mmol, 0.050 equiv), and CuI (9.5 mg, 0.050 mmol, 0.10 equiv). The reaction mixture was stirred at 110 °C in an oil bath for about 8 h. The resulting mixture was concentrated and the residue was taken up in ethyl acetate. The organic layer was washed with brine, dried over Na_2SO_4 and concentrated. Purification of the crude product by column chromatography (silica gel; petroleum ether/ethyl acetate 10:1) afforded **4e** in 74% yield (144 mg).

White solid; mp 192–194 °C; ^{1}H NMR ($CDCl_3$, 400 MHz): δ_H 8.84 (d, *J* = 7.6 Hz, 1H), 8.21 (d, *J* = 8.0 Hz, 1H), 8.04 (dd, *J* = 10.4, 2.8 Hz, 1H), 7.70–7.62 (m, 2H), 7.59–7.51 (m, 7H), 7.27–7.19 (m, 4H), 6.90–6.85 (m, 1H); ^{13}C NMR ($CDCl_3$, 101 MHz): δ_C 159.4 (d, $J_{C–F}$ = 243.1 Hz), 142.1, 141.3, 134.3, 132.8, 131.6, 129.8, 129.7, 129.4, 129.1, 128.7, 128.1, 127.8, 127.0, 126.9, 125.2, 124.8 (d, $J_{C–F}$ = 7.9 Hz), 124.7, 124.1, 122.3, 119.2 (d, $J_{C–F}$ = 8.2 Hz), 115.3 (d, $J_{C–F}$ = 23.2 Hz), 109.9 (d, $J_{C–F}$ = 23.7 Hz); ^{19}F NMR ($CDCl_3$, 376 MHz): δ_F –116.8; HRMS (ESI-TOF, *m/z*): calcd for $C_{27}H_{18}FN_2$ $[M + H]^+$, 389.1449; found, 389.1471.

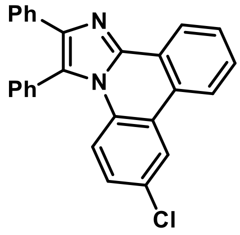

7-Chloro-2,3-diphenylimidazo[1,2-*f*]phenanthridine (4f)

To a 10 mL Schlenk tube equipped with a magnetic stir bar was added 2-(2-bromophenyl)-4,5-diphenyl-1*H*-imidazole **1n** (187.5 mg, 0.500 mmol, 1.00 equiv), 2-bromo-4-chlorobenzoic acid **2c** (176.3 mg, 0.750 mmol, 1.50 equiv), DMF (4.0 mL), Cs_2CO_3 (163 mg, 0.500 mmol, 1.00 equiv), PPh_3 (26.2 mg, 0.10 mmol, 0.20 equiv), $Pd(OAc)_2$ (5.6 mg, 0.025 mmol, 0.050 equiv), and CuI (9.5 mg, 0.050 mmol, 0.10 equiv). The reaction mixture was stirred at 110 °C in an oil bath for about 8 h. The resulting

mixture was concentrated and the residue was taken up in ethyl acetate. The organic layer was washed with brine, dried over Na_2SO_4 and concentrated. Purification of the crude product by column chromatography (silica gel; petroleum ether/ethyl acetate 10:1) afforded **4f** in 66% yield (134 mg). White solid; mp 183–184 °C; ^{1}H NMR (CDCl$_3$, 400 MHz): δ_H 8.85 (dd, *J* = 7.6, 2.0 Hz, 1H), 8.38 (d, *J* = 2.4 Hz, 1H), 8.29 (d, *J* = 8.0 Hz, 1H), 7.72–7.64 (m, 2H), 7.60–7.51 (m, 7H), 7.28–7.26 (m, 1H), 7.26–7.19 (m, 3H), 7.12 (dd, *J* = 9.2, 2.4 Hz, 1H); ^{13}C NMR (CDCl$_3$, 101 MHz): δ_C 142.3, 141.4, 134.2, 132.7, 131.8, 131.5, 130.4, 129.7, 129.5, 129.2, 129.0, 128.8, 128.2, 127.8, 127.1, 126.6, 125.3, 124.7, 124.4, 124.1, 123.8, 122.2, 118.8; HRMS (ESI-TOF, *m/z*): calcd for $C_{27}H_{18}ClN_2$ [M + H]$^+$, 405.1153; found, 405.1173.

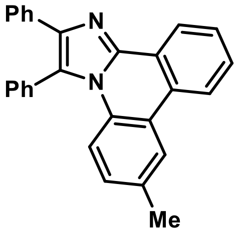

7-Methyl-2,3-diphenylimidazo[1,2-*f*]phenanthridine (4g)[7]

To a 10 mL Schlenk tube equipped with a magnetic stir bar was added 2-(2-bromophenyl)-4,5-diphenyl-1*H*-imidazole **1n** (187.5 mg, 0.500 mmol, 1.00 equiv), 2-bromo-4-methylbenzoic acid **2d** (161.3 mg, 0.750 mmol, 1.50 equiv), DMF (4.0 mL), Cs_2CO_3 (163 mg, 0.500 mmol, 1.00 equiv), PPh_3 (26.2 mg, 0.10 mmol, 0.20 equiv), $Pd(OAc)_2$ (5.6 mg, 0.025 mmol, 0.050 equiv), and CuI (9.5 mg, 0.050 mmol, 0.10 equiv). The reaction mixture was stirred at 110 °C in an oil bath for about 8 h. The resulting mixture was concentrated and the residue was taken up in ethyl acetate. The organic layer was washed with brine, dried over Na_2SO_4 and concentrated. Purification of the crude product by column chromatography (silica gel; petroleum ether/ethyl acetate 10:1) afforded **4g** in 72% yield (138 mg). White solid; mp 196–198 °C; ^{1}H NMR (CDCl$_3$, 400 MHz): δ_H 8.86–8.84 (m, 1H), 8.37–8.35 (m, 1H), 8.23 (s, 1H), 7.67–7.63 (m, 2H), 7.60–7.53 (m, 7H), 7.27–7.26 (m, 1H), 7.23–7.14 (m, 3H), 6.99 (d, *J* = 8.4Hz, 1H), 2.46 (s, 3H); ^{13}C NMR (CDCl$_3$, 101 MHz): δ_C 142.4, 141.1, 134.5, 134.1, 133.2, 131.6, 131.3, 129.5, 129.2, 128.9, 128.5, 128.3, 128.1, 127.9, 127.6, 126.9, 125.2, 124.6, 124.1, 123.9, 122.7, 122.1, 117.4, 21.2; HRMS (ESI-TOF, *m/z*): calcd for $C_{28}H_{21}N_2$ [M + H]$^+$, 385.1699; found, 385.1711.

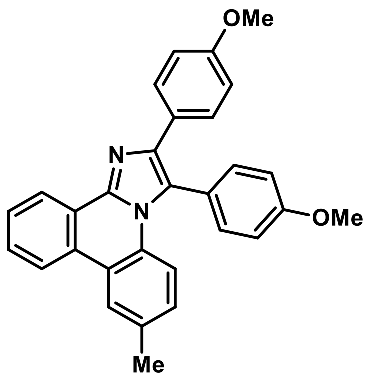

2,3-Bis(4-methoxyphenyl)-7-methylimidazo[1,2-*f*]phenanthridine (4h)

To a 10 mL Schlenk tube equipped with a magnetic stir bar was added 2-(2-bromophenyl)-4,5-bis(4-methoxyphenyl)-1*H*-imidazole **1y** (217.5 mg, 0.500 mmol, 1.00 equiv), 2-bromo-4-methylbenzoic acid **2d** (161.3 mg, 0.750 mmol, 1.50 equiv), DMF (4.0 mL), Cs_2CO_3 (163 mg, 0.500 mmol, 1.00 equiv), PPh_3 (26.2 mg, 0.10 mmol, 0.20 equiv), $Pd(OAc)_2$ (5.6 mg, 0.025 mmol, 0.050 equiv), and CuI (9.5 mg, 0.050 mmol, 0.10 equiv). The reaction mixture was stirred at 110 °C in an oil bath for about 8 h. The resulting mixture was concentrated and the residue was taken up in ethyl acetate. The organic layer was washed with brine, dried over Na_2SO_4 and concentrated. Purification of the crude product by column chromatography (silica gel; petroleum ether/ethyl acetate 10:1) afforded **4h** in 75% yield (167 mg). White solid; mp 232–234 °C; ^{1}H NMR ($CDCl_3$, 400 MHz): δ_H 8.84–8.81 (m, 1H), 8.35–8.33 (m, 1H), 8.21 (s, 1H), 7.65–7.59 (m, 2H), 7.55 (d, *J* = 8.4 Hz, 2H), 7.42 (d, *J* = 8.8 Hz, 2H), 7.22 (d, *J* = 8.4 Hz, 1H), 7.07 (d, *J* = 8.4 Hz, 2H), 7.01 (d, *J* = 8.4 Hz, 1H), 6.81 (d, *J* = 8.4 Hz, 2H), 3.94 (s, 3H), 3.78 (s, 3H), 2.45 (s, 3H); ^{13}C NMR ($CDCl_3$, 101 MHz): δ_C 160.1, 158.5, 142.0, 140.9, 133.8, 132.9, 131.5, 128.9, 128.86, 128.3, 128.2, 127.5, 127.4, 125.3, 124.5, 124.1, 124.0, 123.9, 122.5, 122.0, 117.2, 114.9, 113.5, 55.3, 55.2, 21.2; HRMS (ESI-TOF, *m/z*): calcd for $C_{30}H_{25}N_2O_2$ $[M + H]^+$, 445.1911; found, 445.1910.

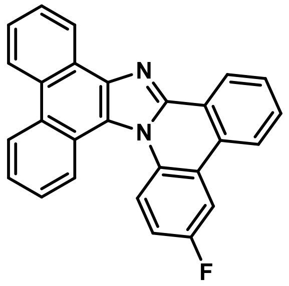

3-Fluorophenanthro[9',10':4,5]imidazo[1,2-*f*]phenanthridine (4i)

To a 10 mL Schlenk tube equipped with a magnetic stir bar was added 2-(2-bromophenyl)-1*H*-phenanthro[9,10-*d*]imidazole **1ac** (186.5 mg, 0.500 mmol, 1.00 equiv), 2-bromo-4-fluorobenzoic acid **2b** (164.3 mg, 0.750 mmol, 1.50 equiv), DMF (4.0 mL), Cs_2CO_3 (163 mg, 0.500 mmol, 1.00 equiv), PPh_3 (26.2 mg, 0.10 mmol, 0.20 equiv), $Pd(OAc)_2$ (5.6 mg, 0.025 mmol, 0.050 equiv), and CuI (9.5 mg, 0.050 mmol, 0.10 equiv). The reaction mixture was stirred at 110 °C in an oil bath for about 8 h. The resulting mixture was concentrated and the residue was taken up in ethyl acetate. The organic layer was washed with brine, dried over Na_2SO_4 and concentrated. Purification of the crude product by column chromatography (silica gel; petroleum ether/ethyl acetate 10:1) afforded **4i** in 71% yield (137 mg).

White solid; mp 267–268 °C; ^{1}H NMR ($CDCl_3$, 400 MHz): δ_H 8.94 (d, *J* = 7.2 Hz, 2H), 8.82 (d, *J* = 8.4 Hz, 1H), 8.75 (d, *J* = 8.0 Hz, 1H), 8.44–8.41 (m, 2H), 8.32 (d, *J* = 7.6 Hz, 1H), 8.12 (d, *J* = 9.6 Hz, 1H), 7.81–7.71 (m, 4H), 7.67–7.58 (m, 2H), 7.32–7.28 (m, 1H); ^{13}C NMR ($CDCl_3$, 101 MHz): δ_C 160.0 (d, J_{C-F} = 254.2 Hz), 147.3, 141.4, 130.0, 129.7, 129.6, 129.3, 129.1, 128.7, 127.5, 127.0, 126.5, 125.6, 125.5, 125.2, 124.7, 124.4, 124.0, 123.6, 123.3, 123.1, 122.75, 122.71, 122.5, 121.0 (d, J_{C-F} = 8.1 Hz), 114.7 (d, J_{C-F} = 23.7 Hz), 110.6 (d, J_{C-F} = 23.8 Hz); ^{19}F NMR ($CDCl_3$, 376 MHz): δ_F –109.3; HRMS (ESI-TOF, *m/z*): calcd for $C_{27}H_{16}FN_2$ $[M + H]^+$, 387.1292; found, 387.1313.

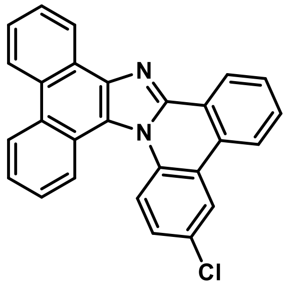

3-Methylphenanthro[9',10':4,5]imidazo[1,2-*f*]phenanthridine (4j)

To a 10 mL Schlenk tube equipped with a magnetic stir bar was added 2-(2-bromophenyl)-1*H*-phenanthro[9,10-*d*]imidazole **1ac** (186.5 mg, 0.500 mmol, 1.00 equiv), 2-bromo-4-chlorobenzoic acid **2c** (176.3 mg, 0.750 mmol, 1.50 equiv), DMF (4.0 mL), Cs_2CO_3 (163 mg, 0.500 mmol, 1.00 equiv), PPh_3 (26.2 mg, 0.10 mmol, 0.20 equiv), $Pd(OAc)_2$ (5.6 mg, 0.025 mmol, 0.050 equiv), and CuI (9.5 mg, 0.050 mmol, 0.10 equiv). The reaction mixture was stirred at 110 °C in an oil bath for about 8 h. The resulting mixture was concentrated and the residue was taken up in ethyl acetate. The organic layer was washed with brine, dried over Na_2SO_4 and concentrated. Purification of the crude product by column chromatography (silica gel; petroleum ether/ethyl acetate 10:1) afforded **4j** in 69% yield (139 mg). White solid; mp 281–283 °C; ^{1}H NMR ($CDCl_3$, 400 MHz): δ_H 8.93–8.90 (m, 2H), 8.81 (d, *J* = 8.0 Hz, 1H), 8.74 (d, *J* = 8.4 Hz, 1H), 8.42–8.34 (m, 4H), 7.80–7.70 (m, 4H), 7.66–7.57 (m, 2H), 7.53 (d, *J* = 8.8 Hz, 1H); ^{13}C NMR ($CDCl_3$, 101 MHz): δ_C 147.4, 141.5, 132.0, 130.8, 129.8, 129.7, 129.3, 129.1, 128.3, 127.5, 127.2, 127.0, 126.6, 125.5, 125.3, 124.7, 124.6, 124.4, 124.36, 124.0, 123.5, 123.3, 123.1, 122.8, 122.4, 120.6; HRMS (ESI-TOF, *m/z*): calcd for $C_{27}H_{16}ClN_2$ [M + H]$^+$, 403.0997; found, 403.0982.

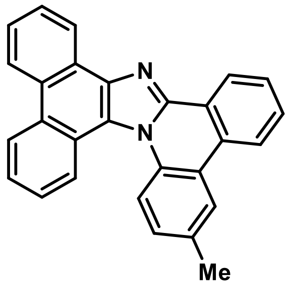

3-Methylphenanthro[9',10':4,5]imidazo[1,2-*f*]phenanthridine (4k)

To a 10 mL Schlenk tube equipped with a magnetic stir bar was added 2-(2-bromophenyl)-1*H*-phenanthro[9,10-*d*]imidazole **1ac** (186.5 mg, 0.500 mmol, 1.00 equiv), 2-bromo-4-methylbenzoic acid **2d** (161.3 mg, 0.750 mmol, 1.50 equiv), DMF (4.0 mL), Cs_2CO_3 (163 mg, 0.500 mmol, 1.00 equiv), PPh_3 (26.2 mg, 0.10 mmol, 0.20 equiv), $Pd(OAc)_2$ (5.6 mg, 0.025 mmol, 0.050 equiv), and CuI (9.5 mg, 0.050

mmol, 0.10 equiv). The reaction mixture was stirred at 110 °C in an oil bath for about 8 h. The resulting mixture was concentrated and the residue was taken up in ethyl acetate. The organic layer was washed with brine, dried over Na_2SO_4 and concentrated. Purification of the crude product by column chromatography (silica gel; petroleum ether/ethyl acetate 10:1) afforded **4k** in 67% yield (128 mg).

White solid; mp 221–223 °C; ^1H NMR ($CDCl_3$, 400 MHz): δ_H 8.96–8.93 (m, 2H), 8.82 (d, J = 8.0 Hz, 1H), 8.75 (d, J = 8.0 Hz, 1H), 8.50 (d, J = 8.0 Hz, 1H), 8.45–8.42 (m, 1H), 8.33 (d, J = 8.4 Hz, 1H), 8.28 (s, 1H), 7.79 (t, J = 7.6 Hz, 1H), 7.73–7.70 (m, 3H), 7.66–7.57 (m, 2H), 7.39 (d, J = 8.0 Hz, 1H), 2.61 (s, 3H); ^{13}C NMR ($CDCl_3$, 101 MHz): δ_C 147.6, 141.2, 134.9, 131.5, 129.6, 129.5, 129.3, 128.9, 128.5, 128.2, 127.4, 127.1, 126.3, 125.4, 125.3, 125.0, 124.7, 124.5, 124.2, 124.0, 123.8, 123.3, 123.1, 123.0, 122.98, 122.2, 119.3, 21.4; HRMS (ESI-TOF, *m/z*): calcd for $C_{28}H_{19}N_2$ $[M + H]^+$, 383.1543; found, 383.1563.

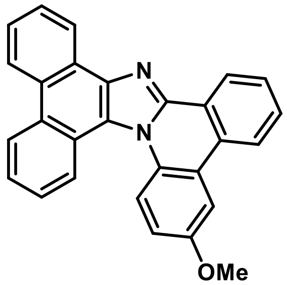

3-Methoxyphenanthro[9',10':4,5]imidazo[1,2-*f*]phenanthridine (4l)

To a 10 mL Schlenk tube equipped with a magnetic stir bar was added 2-(2-bromophenyl)-1*H*-phenanthro[9,10-*d*]imidazole **1ac** (186.5 mg, 0.500 mmol, 1.00 equiv), 2-bromo-4-methoxybenzoic acid **2e** (173.3 mg, 0.750 mmol, 1.50 equiv), DMF (4.0 mL), Cs_2CO_3 (163 mg, 0.500 mmol, 1.00 equiv), PPh_3 (26.2 mg, 0.10 mmol, 0.20 equiv), $Pd(OAc)_2$ (5.6 mg, 0.025 mmol, 0.050 equiv), and CuI (9.5 mg, 0.050 mmol, 0.10 equiv). The reaction mixture was stirred at 110 °C in an oil bath for about 8 h. The resulting mixture was concentrated and the residue was taken up in ethyl acetate. The organic layer was washed with brine, dried over Na_2SO_4 and concentrated. Purification of the crude product by column chromatography (silica gel; petroleum ether/ethyl acetate 10:1) afforded **4l** in 72% yield (143 mg).

White solid; mp 175–177 °C; ^1H NMR ($CDCl_3$, 400 MHz): δ_H 8.95–8.91 (m, 2H), 8.80 (d, J = 8.0 Hz, 1H), 8.73 (d, J = 8.0 Hz, 1H), 8.47 (d, J = 8.0 Hz, 1H), 8.36–8.33 (m, 2H), 7.88 (d, J = 2.8 Hz, 1H), 7.80–7.76 (m, 1H), 7.72–7.68 (m, 3H), 7.64–7.55 (m, 2H), 7.14 (dd, J = 8.8, 2.4 Hz, 1H), 4.01 (s, 3H); ^{13}C NMR ($CDCl_3$, 101 MHz): δ_C 156.9, 147.1, 141.1, 129.5, 129.4, 129.1, 128.9, 128.7, 127.8, 127.3, 127.2, 126.3,

125.5, 125.4, 125.0, 124.5, 124.4, 124.3, 123.9, 123.8, 123.2, 123.0, 122.9, 122.3, 120.6, 114.0, 108.1, 55.8; HRMS (ESI-TOF, *m/z*): calcd for $C_{28}H_{19}N_2O$ [M + H]$^+$, 399.1492; found, 399.1492.

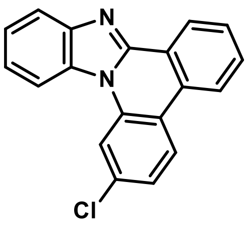

2-Chlorobenzo[4,5]imidazo[1,2-*f*]phenanthridine (4m)[6]

To a 10 mL Schlenk tube equipped with a magnetic stir bar was added 2-(2-bromophenyl)-1*H*-benzo[*d*]imidazole **1a** (136.5 mg, 0.500 mmol, 1.00 equiv), 2-bromo-5-chlorobenzoic acid **2f** (176.6 mg, 0.750 mmol, 1.50 equiv), DMF (4.0 mL), Cs_2CO_3 (163 mg, 0.500 mmol, 1.00 equiv), PPh_3 (26.2 mg, 0.10 mmol, 0.20 equiv), $Pd(OAc)_2$ (5.6 mg, 0.025 mmol, 0.050 equiv), and CuI (9.5 mg, 0.050 mmol, 0.10 equiv). The reaction mixture was stirred at 110 °C in an oil bath for about 8 h. The resulting mixture was concentrated and the residue was taken up in ethyl acetate. The organic layer was washed with brine, dried over Na_2SO_4 and concentrated. Purification of the crude product by column chromatography (silica gel; petroleum ether/ethyl acetate 10:1) afforded **4m** in 69% yield (105 mg).

White solid; mp 215–217 °C; ^{1}H NMR ($CDCl_3$, 400 MHz): δ_H 8.88 (d, *J* = 8.0 Hz, 1H), 8.51 (d, *J* = 8.8 Hz, 1H), 8.45 (d, *J* = 2.0 Hz, 1H), 8.33 (d, *J* = 8.0 Hz, 1H), 8.29 (d, *J* = 8.0 Hz, 1H), 8.06 (d, *J* = 8.0 Hz, 1H), 7.79–7.71 (m, 2H), 7.67 (dd, *J* = 9.2, 2.4 Hz, 1H), 7.56–7.48 (m, 2H); ^{13}C NMR ($CDCl_3$, 101 MHz): δ_C 147.3, 144.5, 132.9, 131.7, 130.7, 130.2, 129.4, 129.1, 128.4, 126.2, 124.4, 124.2, 123.8, 123.4, 123.2, 122.4, 120.6, 117.3, 113.7; HRMS (ESI-TOF, *m/z*): calcd for $C_{19}H_{12}ClN_2$ [M + H]$^+$, 303.0684; found, 303.0691.

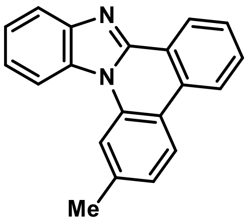

2-Methylbenzo[4,5]imidazo[1,2-*f*]phenanthridine (4n)[3]

To a 10 mL Schlenk tube equipped with a magnetic stir bar was added 2-(2-bromophenyl)-1*H*-benzo[*d*]imidazole **1a** (136.5 mg, 0.500 mmol, 1.00 equiv), 2-bromo-5-methylbenzoic acid **2g** (161.3 mg, 0.750 mmol, 1.50 equiv), DMF (4.0 mL), Cs_2CO_3 (163 mg, 0.500 mmol, 1.00 equiv), PPh_3 (26.2 mg,

0.10 mmol, 0.20 equiv), $Pd(OAc)_2$ (5.6 mg, 0.025 mmol, 0.050 equiv), and CuI (9.5 mg, 0.050 mmol, 0.10 equiv). The reaction mixture was stirred at 110 °C in an oil bath for about 8 h. The resulting mixture was concentrated and the residue was taken up in ethyl acetate. The organic layer was washed with brine, dried over Na_2SO_4 and concentrated. Purification of the crude product by column chromatography (silica gel; petroleum ether/ethyl acetate 10:1) afforded **4n** in 66% yield (93 mg).
White solid; mp 184–186 °C; ^{1}H NMR ($CDCl_3$, 400 MHz): δ_H 8.89 (d, J = 8.0 Hz, 1H), 8.48 (d, J = 8.4 Hz, 1H), 8.41 (d, J = 8.0 Hz, 1H), 8.35 (d, J = 8.0 Hz, 1H), 8.30 (s, 1H), 8.06 (d, J = 7.6 Hz, 1H), 7.77–7.67 (m, 2H), 7.54–7.46 (m, 3H), 2.59 (s, 3H); ^{13}C NMR ($CDCl_3$, 101 MHz): δ_C 144.6, 139.6, 137.3, 135.5, 135.3, 130.5, 130.1, 129.8, 128.2, 126.0, 125.7, 125.6, 124.1, 124.06, 122.8, 122.1, 120.3, 116.4, 114.0, 22.0; HRMS (ESI-TOF, *m/z*): calcd for $C_{20}H_{15}N_2$ $[M + H]^+$, 283.1230; found, 283.1242.

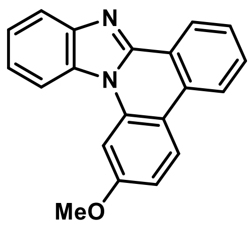

2-Methoxybenzo[4,5]imidazo[1,2-*f*]phenanthridine (4o)[3]

To a 10 mL Schlenk tube equipped with a magnetic stir bar was added 2-(2-bromophenyl)-1*H*-benzo[*d*]imidazole **1a** (136.5 mg, 0.500 mmol, 1.00 equiv), 2-bromo-5-methoxybenzoic acid **2h** (173.3 mg, 0.750 mmol, 1.50 equiv), DMF (4.0 mL), Cs_2CO_3 (163 mg, 0.500 mmol, 1.00 equiv), PPh_3 (26.2 mg, 0.10 mmol, 0.20 equiv), $Pd(OAc)_2$ (5.6 mg, 0.025 mmol, 0.050 equiv), and CuI (9.5 mg, 0.050 mmol, 0.10 equiv). The reaction mixture was stirred at 110 °C in an oil bath for about 8 h. The resulting mixture was concentrated and the residue was taken up in ethyl acetate. The organic layer was washed with brine, dried over Na_2SO_4 and concentrated. Purification of the crude product by column chromatography (silica gel; petroleum ether/ethyl acetate 10:1) afforded **4o** in 70% yield (104 mg).
White solid; mp 217–219 °C; ^{1}H NMR ($CDCl_3$, 400 MHz): δ_H 8.89 (d, J = 8.0 Hz, 1H), 8.52 (d, J = 9.2 Hz, 1H), 8.33 (t, J = 8.4 Hz, 2H), 8.06 (d, J = 8.0 Hz, 1H), 7.94 (d, J = 2.8 Hz, 1H), 7.77–7.68 (m, 2H), 7.54–7.45 (m, 2H), 7.30 (dd, J = 9.2, 2.8 Hz, 1H), 4.01 (s, 3H); ^{13}C NMR ($CDCl_3$, 101 MHz): δ_C 156.3, 147.1, 144.4, 131.8, 130.3, 129.3, 128.8, 126.1, 123.9, 123.7, 123.1, 122.8, 122.4, 120.4, 117.2, 115.9, 113.6, 108.2, 108.1, 55.7; HRMS (ESI-TOF, *m/z*): calcd for $C_{20}H_{15}N_2O$ $[M + H]^+$, 299.1179; found, 299.1190.

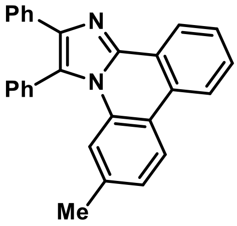

6-Methyl-2,3-diphenylimidazo[1,2-*f*]phenanthridine (4p)

To a 10 mL Schlenk tube equipped with a magnetic stir bar was added 2-(2-bromophenyl)-4,5-diphenyl-1*H*-imidazole **1n** (187.5 mg, 0.500 mmol, 1.00 equiv), 2-bromo-5-methylbenzoic acid **2g** (161.3 mg, 0.750 mmol, 1.50 equiv), DMF (4.0 mL), Cs_2CO_3 (163 mg, 0.500 mmol, 1.00 equiv), PPh_3 (26.2 mg, 0.10 mmol, 0.20 equiv), $Pd(OAc)_2$ (5.6 mg, 0.025 mmol, 0.050 equiv), and CuI (9.5 mg, 0.050 mmol, 0.10 equiv). The reaction mixture was stirred at 110 °C in an oil bath for about 8 h. The resulting mixture was concentrated and the residue was taken up in ethyl acetate. The organic layer was washed with brine, dried over Na_2SO_4 and concentrated. Purification of the crude product by column chromatography (silica gel; petroleum ether/ethyl acetate 10:1) afforded **4p** in 73% yield (140 mg).

White solid; mp 201–203 °C; ^{1}H NMR ($CDCl_3$, 400 MHz): δ_H 8.85 (dd, *J* = 6.4, 3.6 Hz, 1H), 8.34–8.30 (m, 2H), 7.66–7.55 (m, 9H), 7.28–7.26 (m, 1H), 7.26–7.18 (m, 3H), 6.99 (s, 1H), 2.11 (s, 3H); ^{13}C NMR ($CDCl_3$, 101 MHz): δ_C 142.7, 141.0, 138.0, 134.5, 133.3, 133.28, 131.6, 129.4, 129.2, 128.6, 128.1, 128.0, 127.8, 126.9, 125.7, 125.3, 124.6, 123.8, 123.5, 121.8, 120.2, 118.1, 21.6; HRMS (ESI-TOF, *m/z*): calcd for $C_{28}H_{21}N_2$ $[M + H]^+$, 385.1699; found, 385.1711.

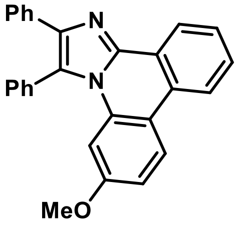

6-Methoxy-2,3-diphenylimidazo[1,2-*f*]phenanthridine (4q)

To a 10 mL Schlenk tube equipped with a magnetic stir bar was added 2-(2-bromophenyl)-4,5-diphenyl-1*H*-imidazole **1n** (187.5 mg, 0.500 mmol, 1.00 equiv), 2-bromo-5-methoxybenzoic acid **2h** (173.3 mg, 0.750 mmol, 1.50 equiv), DMF (4.0 mL), Cs_2CO_3 (163 mg, 0.500 mmol, 1.00 equiv), PPh_3 (26.2 mg, 0.10 mmol, 0.20 equiv), $Pd(OAc)_2$ (5.6 mg, 0.025 mmol, 0.050 equiv), and CuI (9.5 mg, 0.050 mmol, 0.10 equiv). The reaction mixture was stirred at 110 °C in an oil bath for about 8 h. The resulting mixture was concentrated and the residue was taken up in ethyl acetate. The organic layer was

washed with brine, dried over Na_2SO_4 and concentrated. Purification of the crude product by column chromatography (silica gel; petroleum ether/ethyl acetate 10:1) afforded **4q** in 75% yield (150 mg). White solid; mp 215–216 °C; ^{1}H NMR ($CDCl_3$, 400 MHz): δ_H 8.85–8.83 (m, 1H), 8.32–8.25 (m, 2H), 7.65–7.55 (m, 9H), 7.28–7.19 (m, 3H), 6.96 (dd, *J* = 8.8, 2.4 Hz, 1H), 6.90 (d, *J* = 2.4 Hz, 1H), 3.30 (s, 3H); ^{13}C NMR ($CDCl_3$, 101 MHz): δ_C 159.1, 142.9, 141.1, 134.43, 134.4, 133.5, 132.1, 129.5, 129.2, 128.7, 128.1, 127.9, 127.8, 127.4, 127.0, 125.2, 124.9, 124.6, 122.7, 121.5, 115.8, 113.8, 100.8, 54.6; HRMS (ESI-TOF, *m/z*): calcd for $C_{28}H_{21}N_2O$ [M + H]$^+$, 401.1648; found, 401.1667.

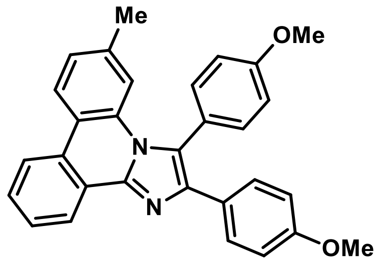

2,3-Bis(4-methoxyphenyl)-6-methylimidazo[1,2-*f*]phenanthridine (4r)

To a 10 mL Schlenk tube equipped with a magnetic stir bar was added 2-(2-bromophenyl)-4,5-bis(4-methoxyphenyl)-1*H*-imidazole **1y** (217.5 mg, 0.500 mmol, 1.00 equiv), 2-bromo-5-methylbenzoic acid **2g** (161.3 mg, 0.750 mmol, 1.50 equiv), DMF (4.0 mL), Cs_2CO_3 (163 mg, 0.500 mmol, 1.00 equiv), PPh_3 (26.2 mg, 0.10 mmol, 0.20 equiv), $Pd(OAc)_2$ (5.6 mg, 0.025 mmol, 0.050 equiv), and CuI (9.5 mg, 0.050 mmol, 0.10 equiv). The reaction mixture was stirred at 110 °C in an oil bath for about 8 h. The resulting mixture was concentrated and the residue was taken up in ethyl acetate. The organic layer was washed with brine, dried over Na_2SO_4 and concentrated. Purification of the crude product by column chromatography (silica gel; petroleum ether/ethyl acetate 10:1) afforded **4r** in 70% yield (155 mg). White solid; mp 209–211 °C; ^{1}H NMR ($CDCl_3$, 400 MHz): δ_H 8.84–8.81 (m, 1H), 8.33–8.30 (m, 2H), 7.63–7.57 (m, 4H), 7.44 (d, *J* = 8.4 Hz, 2H), 7.19 (d, *J* = 8.4 Hz, 1H), 7.10 (d, *J* = 8.8 Hz, 3H), 6.82 (d, *J* = 8.8 Hz, 2H), 3.95 (s, 3H), 3.79 (s, 3H), 2.16 (s, 3H); ^{13}C NMR ($CDCl_3$, 101 MHz): δ_C 160.2, 158.6, 142.4, 140.9, 137.9, 133.5, 132.9, 128.9, 128.4, 127.9, 127.7, 127.4, 125.5, 125.4, 124.5, 124.2, 123.8, 123.5, 121.8, 120.1, 118.0, 114.9, 113.6, 55.4, 55.2, 21.8; HRMS (ESI-TOF, *m/z*): calcd for $C_{30}H_{25}N_2O_2$ [M + H]$^+$, 445.1911; found, 445.1910.

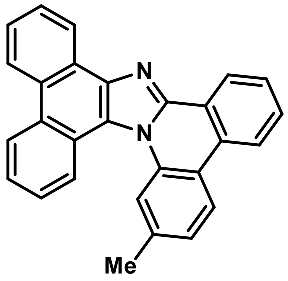

2-Methylphenanthro[9',10':4,5]imidazo[1,2-*f*]phenanthridine (4s)[3]

To a 10 mL Schlenk tube equipped with a magnetic stir bar was added 2-(2-bromophenyl)-1*H*-phenanthro[9,10-*d*]imidazole **1ac** (186.5 mg, 0.500 mmol, 1.00 equiv), 2-bromo-5-methylbenzoic acid **2g** (161.3 mg, 0.750 mmol, 1.50 equiv), DMF (4.0 mL), Cs_2CO_3 (163 mg, 0.500 mmol, 1.00 equiv), PPh_3 (26.2 mg, 0.10 mmol, 0.20 equiv), $Pd(OAc)_2$ (5.6 mg, 0.025 mmol, 0.050 equiv), and CuI (9.5 mg, 0.050 mmol, 0.10 equiv). The reaction mixture was stirred at 110 °C in an oil bath for about 8 h. The resulting mixture was concentrated and the residue was taken up in ethyl acetate. The organic layer was washed with brine, dried over Na_2SO_4 and concentrated. Purification of the crude product by column chromatography (silica gel; petroleum ether/ethyl acetate 10:1) afforded **4s** in 81% yield (155 mg).

White solid; mp 258–260 °C; ^{1}H NMR ($CDCl_3$, 400 MHz): δ_H 8.97–8.91 (m, 2H), 8.83 (d, *J* = 8.0 Hz, 1H), 8.76 (d, *J* = 8.4 Hz, 1H), 8.50 (d, *J* = 8.0 Hz, 1H), 8.40–8.37 (m, 1H), 8.34 (d, *J* = 8.0 Hz, 1H), 8.26 (s, 1H), 7.81–7.77 (m, 1H), 7.74–7.68 (m, 3H), 7.66–7.56 (m, 2H), 7.37 (d, *J* = 8.4 Hz, 1H), 2.53 (s, 3H); ^{13}C NMR ($CDCl_3$, 101 MHz): δ_C 147.9, 141.4, 137.7, 133.5, 129.6, 129.5, 129.49, 129.0, 128.2, 127.4, 127.1, 126.4, 125.4, 125.2, 125.0, 124.5, 124.47, 124.0, 123.9, 123.8, 123.3, 123.2, 123.0, 122.0, 120.6, 119.8, 21.6; HRMS (ESI-TOF, *m/z*): calcd for $C_{28}H_{19}N_2$ $[M + H]^+$, 383.1543; found, 383.1563.

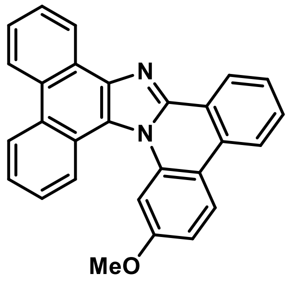

2-Methoxyphenanthro[9',10':4,5]imidazo[1,2-*f*]phenanthridine (4t)

To a 10 mL Schlenk tube equipped with a magnetic stir bar was added 2-(2-bromophenyl)-1*H*-phenanthro[9,10-*d*]imidazole **1ac** (186.5 mg, 0.500 mmol, 1.00 equiv), 2-bromo-5-methoxybenzoic acid **2h** (173.3 mg, 0.750 mmol, 1.50 equiv), DMF (4.0 mL), Cs_2CO_3 (163 mg, 0.500 mmol, 1.00 equiv), PPh_3 (26.2 mg, 0.10 mmol, 0.20 equiv), $Pd(OAc)_2$ (5.6 mg, 0.025 mmol, 0.050 equiv), and CuI (9.5 mg, 0.050 mmol, 0.10 equiv). The reaction mixture was stirred at 110 °C in an oil bath for about 8 h. The resulting mixture was concentrated and the residue was taken up in ethyl acetate. The organic layer was washed with brine, dried over Na_2SO_4 and concentrated. Purification of the crude product by column chromatography (silica gel; petroleum ether/ethyl acetate 10:1) afforded **4t** in 82% yield (163 mg).

White solid; mp 175–177 °C; ^{1}H NMR ($CDCl_3$, 400 MHz): δ_H 8.96 (d, *J* = 8.0 Hz, 1H), 8.91 (d, *J* = 8.0 Hz, 1H), 8.82 (d, *J* = 8.8 Hz, 1H), 8.75 (d, *J* = 8.0 Hz, 1H), 8.50–8.48 (m, 1H), 8.36 (d, *J* = 9.2 Hz, 1H), 8.32 (d, *J* = 8.0 Hz, 1H), 7.86 (d, *J* = 2.4 Hz, 1H), 7.81–7.77 (m, 1H), 7.74–7.65 (m, 3H), 7.64–7.59 (m, 2H), 7.13 (dd, *J* = 8.9, 2.2 Hz, 1H), 3.86 (s, 3H); ^{13}C NMR ($CDCl_3$, 101 MHz): δ_C 158.8, 148.2, 141.5, 134.7, 129.6, 129.5, 129.0, 127.5, 127.4, 127.1, 126.5, 126.0, 125.4, 125.1, 125.0, 124.6, 123.8, 123.7, 123.4, 123.0, 121.7, 116.2, 113.5, 103.5, 55.7; HRMS (ESI-TOF, *m/z*): calcd for $C_{28}H_{19}N_2O$ $[M + H]^+$, 399.1492; found, 399.1492.

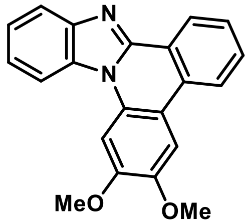

2,3-Dimethoxybenzo[4,5]imidazo[1,2-*f*]phenanthridine (4u)

To a 10 mL Schlenk tube equipped with a magnetic stir bar was added 2-(2-bromophenyl)-1*H*-benzo[*d*]imidazole **1a** (136.5 mg, 0.500 mmol, 1.00 equiv), 2-bromo-4,5-dimethoxybenzoic acid **2i**

(150.7 mg, 0.750 mmol, 1.50 equiv), DMF (4.0 mL), Cs_2CO_3 (163 mg, 0.500 mmol, 1.00 equiv), PPh_3 (26.2 mg, 0.10 mmol, 0.20 equiv), $Pd(OAc)_2$ (5.6 mg, 0.025 mmol, 0.050 equiv), and CuI (9.5 mg, 0.050 mmol, 0.10 equiv). The reaction mixture was stirred at 110 °C in an oil bath for about 8 h. The resulting mixture was concentrated and the residue was taken up in ethyl acetate. The organic layer was washed with brine, dried over Na_2SO_4 and concentrated. Purification of the crude product by column chromatography (silica gel; petroleum ether/ethyl acetate 10:1) afforded **4u** in 69% yield (113 mg).

White solid; mp 212–213 °C; ^{1}H NMR ($CDCl_3$, 400 MHz): δ_H 8.81 (d, *J* = 8.0 Hz, 1H), 8.15 (t, *J* = 8.4 Hz, 2H), 8.05 (d, *J* = 8.0 Hz, 1H), 7.90 (s, 1H), 7.70–7.67 (m, 2H), 7.62–7.58 (m, 1H), 7.54–7.50 (m, 1H), 7.44 (t, *J* = 7.6 Hz, 1H), 4.12 (s, 3H), 4.03 (s, 3H); ^{13}C NMR ($CDCl_3$, 101 MHz): δ_C 150.3, 147.5, 146.3, 144.5, 131.4, 130.2, 129.4, 129.0, 127.6, 126.1, 124.0, 122.5, 122.48, 121.6, 120.4, 114.7, 113.1, 105.7, 99.3, 56.3, 56.2; HRMS (ESI-TOF, *m/z*): calcd for $C_{21}H_{17}N_2O_2$ [M + H]$^+$, 329.1285; found, 329.1287.

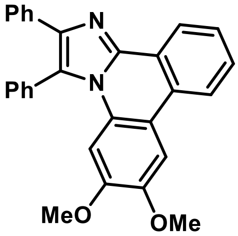

6,7-Dimethoxy-2,3-diphenylimidazo[1,2-*f*]phenanthridine (4v)

To a 10 mL Schlenk tube equipped with a magnetic stir bar was added 2-(2-bromophenyl)-4,5-diphenyl-1*H*-imidazole **1n** (187.5 mg, 0.500 mmol, 1.00 equiv), 2-bromo-4,5-dimethoxybenzoic acid **2i** (150.7 mg, 0.750 mmol, 1.50 equiv), DMF (4.0 mL), Cs_2CO_3 (163 mg, 0.500 mmol, 1.00 equiv), PPh_3 (26.2 mg, 0.10 mmol, 0.20 equiv), $Pd(OAc)_2$ (5.6 mg, 0.025 mmol, 0.050 equiv), and CuI (9.5 mg, 0.050 mmol, 0.10 equiv). The reaction mixture was stirred at 110 °C in an oil bath for about 8 h. The resulting mixture was concentrated and the residue was taken up in ethyl acetate. The organic layer was washed with brine, dried over Na_2SO_4 and concentrated. Purification of the crude product by column chromatography (silica gel; petroleum ether/ethyl acetate 10:1) afforded **4v** in 74% yield (159 mg).

White solid; mp 225–227 °C; ^{1}H NMR ($CDCl_3$, 400 MHz): δ_H 8.87–8.85 (m, 1H), 8.23–8.21 (m, 1H), 7.77 (s, 1H), 7.66–7.52 (m, 9H), 7.28–7.18 (m, 3H), 6.94 (s, 1H), 4.02 (s, 3H), 3.29 (s, 3H); ^{13}C NMR ($CDCl_3$, 101 MHz): δ_C 149.0, 146.4, 142.4, 141.0, 134.5, 133.6, 132.3, 129.5, 129.2, 128.4, 128.1, 128.0, 127.8, 127.5, 127.4, 126.9, 124.7, 124.5, 123.0, 121.5, 115.7, 105.2, 100.9, 56.1, 55.0; HRMS (ESI-TOF, *m/z*): calcd for $C_{29}H_{23}N_2O_2$ [M + H]$^+$, 387.129; found, 431.1769.

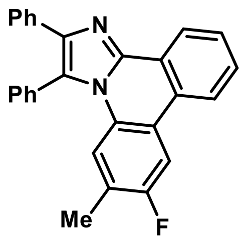

7-Fluoro-6-methyl-2,3-diphenylimidazo[1,2-*f*]phenanthridine (4w)

To a 10 mL Schlenk tube equipped with a magnetic stir bar was added 2-(2-bromophenyl)-4,5-diphenyl-1*H*-imidazole **1n** (187.5 mg, 0.500 mmol, 1.00 equiv), 2-bromo-4-fluoro-5-methylbenzoic acid **2j** (174.7 mg, 0.750 mmol, 1.50 equiv), DMF (4.0 mL), Cs_2CO_3 (163 mg, 0.500 mmol, 1.00 equiv), PPh_3 (26.2 mg, 0.10 mmol, 0.20 equiv), $Pd(OAc)_2$ (5.6 mg, 0.025 mmol, 0.050 equiv), and CuI (9.5 mg, 0.050 mmol, 0.10 equiv). The reaction mixture was stirred at 110 °C in an oil bath for about 8 h. The resulting mixture was concentrated and the residue was taken up in ethyl acetate. The organic layer was washed with brine, dried over Na_2SO_4 and concentrated. Purification of the crude product by column chromatography (silica gel; petroleum ether/ethyl acetate 10:1) afforded **4w** in 74% yield (148 mg).

White solid; mp 231–233 °C; ^{1}H NMR ($CDCl_3$, 400 MHz): δ_H 8.86–8.83 (m, 1H), 8.22–8.20 (m, 1H), 7.99 (d, J = 11.2 Hz, 1H), 7.69–7.53 (m, 9H), 7.28–7.26 (m, 1H), 7.26–7.19 (m, 2H), 6.99 (d, J = 6.8 Hz, 1H), 2.04 (s, 3H); ^{13}C NMR ($CDCl_3$, 101 MHz): δ_C 158.3 (d, $J_{C–F}$ = 242.4 Hz), 142.2, 140.9, 134.4, 133.0, 131.6, 129.6, 129.5, 129.3, 128.6, 128.1, 127.8, 127.1 (d, $J_{C–F}$ = 2.4 Hz), 126.9, 125.4, 125.2, 124.6, 123.8, 122.5 (d, $J_{C–F}$ = 7.8 Hz), 122.0, 120.5, 120.4, 109.3 (d, $J_{C–F}$ = 24.6 Hz), 15.1 (d, $J_{C–F}$ = 2.1 Hz); ^{19}F NMR ($CDCl_3$, 376 MHz): δ_F –120.8; HRMS (ESI-TOF, *m/z*): calcd for $C_{28}H_{20}FN_2$ [M + H]$^+$, 403.1605; found, 403.1628.

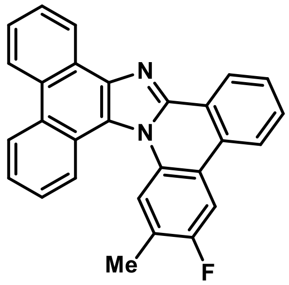

3-Fluoro-2-methylphenanthro[9',10':4,5]imidazo[1,2-*f*]phenanthridine (4x)

To a 10 mL Schlenk tube equipped with a magnetic stir bar was added 2-(2-bromophenyl)-1*H*-phenanthro[9,10-*d*]imidazole **1ac** (186.5 mg, 0.500 mmol, 1.00 equiv), 2-bromo-4-fluoro-5-

methylbenzoic acid **2j** (174.7 mg, 0.750 mmol, 1.50 equiv), DMF (4.0 mL), Cs_2CO_3 (163 mg, 0.500 mmol, 1.00 equiv), PPh_3 (26.2 mg, 0.10 mmol, 0.20 equiv), $Pd(OAc)_2$ (5.6 mg, 0.025 mmol, 0.050 equiv), and CuI (9.5 mg, 0.050 mmol, 0.10 equiv). The reaction mixture was stirred at 110 °C in an oil bath for about 8 h. The resulting mixture was concentrated and the residue was taken up in ethyl acetate. The organic layer was washed with brine, dried over Na_2SO_4 and concentrated. Purification of the crude product by column chromatography (silica gel; petroleum ether/ethyl acetate 10:1) afforded **4x** in 75% yield (150 mg).

White solid; mp 276–278 °C; ^{1}H NMR ($CDCl_3$, 400 MHz): δ_H 8.93–8.87 (m, 2H), 8.81 (d, J = 8.4 Hz, 1H), 8.73 (d, J = 8.0 Hz, 1H), 8.42 (d, J = 8.0 Hz, 1H), 8.24–8.22 (m, 2H), 7.99 (d, J = 10.8 Hz, 1H), 7.79–7.76 (m, 1H), 7.73–7.68 (m, 3H), 7.65–7.61 (m, 1H), 7.58–7.55 (m, 1H), 2.43 (s, 3H); ^{13}C NMR ($CDCl_3$, 101 MHz): δ_C 158.6 (d, J_{C-F} = 243.3 Hz), 147.5, 141.3, 129.6, 129.57, 129.0, 128.8, 127.4, 127.1, 126.4, 125.5, 125.2, 125.1, 124.9, 124.6, 124.0, 123.9, 123.6, 123.3, 123.0, 122.8, 122.6 (d, J_{C-F} = 7.8 Hz), 122.2, 122.0, 121.9, 110.0 (d, J_{C-F} = 24.7 Hz), 15.0 (d, J_{C-F} = 2.9 Hz); ^{19}F NMR ($CDCl_3$, 376 MHz): δ_F –120.2; HRMS (ESI-TOF, *m/z*): calcd for $C_{28}H_{18}FN_2$ $[M + H]^+$, 401.1449; found, 401.1438.

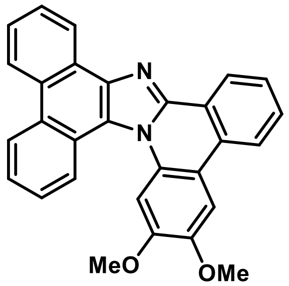

2,3-Dimethoxyphenanthro[9',10':4,5]imidazo[1,2-*f*]phenanthridine (4y)

To a 10 mL Schlenk tube equipped with a magnetic stir bar was added 2-(2-bromophenyl)-1*H*-phenanthro[9,10-*d*]imidazole **1ac** (186.5 mg, 0.500 mmol, 1.00 equiv), 2-bromo-4,5-dimethoxybenzoic acid **2i** (150.7 mg, 0.750 mmol, 1.50 equiv), DMF (4.0 mL), Cs_2CO_3 (163 mg, 0.500 mmol, 1.00 equiv), PPh_3 (26.2 mg, 0.10 mmol, 0.20 equiv), $Pd(OAc)_2$ (5.6 mg, 0.025 mmol, 0.050 equiv), and CuI (9.5 mg, 0.050 mmol, 0.10 equiv). The reaction mixture was stirred at 110 °C in an oil bath for about 8 h. The resulting mixture was concentrated and the residue was taken up in ethyl acetate. The organic layer was washed with brine, dried over Na_2SO_4 and concentrated. Purification of the crude product by column chromatography (silica gel; petroleum ether/ethyl acetate 10:1) afforded **4y** in 68% yield (145 mg).

White solid; mp 297–298 °C; ^{1}H NMR ($CDCl_3$, 400 MHz): δ_H 8.97 (d, J = 7.6 Hz, 2H), 8.85 (d, J = 8.4 Hz, 1H), 8.76 (d, J = 8.4 Hz, 1H), 8.51 (d, J = 7.6 Hz, 1H), 8.33 (d, J = 8.0 Hz, 1H), 7.88 (d, J = 11.6 Hz, 2H), 7.82–7.78 (m, 1H), 7.74–7.70 (m, 3H), 7.65–7.62 (m, 2H), 4.14 (s, 3H), 3.94 (s, 3H); ^{13}C NMR ($CDCl_3$, 101 MHz): δ_C 148.6, 147.5, 147.0, 141.3, 129.5, 129.4, 129.2, 129.0, 128.2, 127.6, 127.4, 127.2, 126.4, 125.5, 125.0, 124.9, 124.7, 123.7, 123.4, 123.2, 123.0, 122.0, 121.7, 116.1, 106.0, 103.1, 56.4, 56.2; HRMS (ESI-TOF, *m/z*): calcd for $C_{29}H_{21}N_2O_2$ [M + H]$^+$, 429.1598; found, 429.1606.

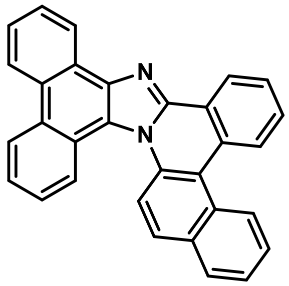

Benzo[*a*]phenanthro[9',10':4,5]imidazo[1,2-*f*]phenanthridine (4z)

To a 10 mL Schlenk tube equipped with a magnetic stir bar was added 2-(2-bromophenyl)-1*H*-benzo[*d*]imidazole **1a** (136.5 mg, 0.500 mmol, 1.00 equiv), 1-bromo-2-naphthoic acid **2k** (188.3 mg, 0.750 mmol, 1.50 equiv), DMF (4.0 mL), Cs_2CO_3 (163 mg, 0.500 mmol, 1.00 equiv), PPh_3 (26.2 mg, 0.10 mmol, 0.20 equiv), $Pd(OAc)_2$ (5.6 mg, 0.025 mmol, 0.050 equiv), and CuI (9.5 mg, 0.050 mmol, 0.10 equiv). The reaction mixture was stirred at 110 °C in an oil bath for about 8 h. The resulting mixture was concentrated and the residue was taken up in ethyl acetate. The organic layer was washed with brine, dried over Na_2SO_4 and concentrated. Purification of the crude product by column chromatography (silica gel; petroleum ether/ethyl acetate 10:1) afforded **4z** in 71% yield (113 mg).

White solid; mp 300–302 °C; ^{1}H NMR ($CDCl_3$, 400 MHz): δ_H 9.09–9.07 (m, 2H), 9.00 (d, J = 8.0 Hz, 1H), 8.95–8 93 (m, 1H), 8.84 (d, J = 8.0 Hz, 1H), 8.78 (d, J = 8.0 Hz, 1H), 8.49–8.45 (m, 2H), 8.06 (d, J = 8.0 Hz, 1H), 8.00 (d, J = 8.8 Hz, 1H), 7.83–7.73 (m, 5H), 7.68–7.58 (m, 3H); ^{13}C NMR ($CDCl_3$, 101 MHz): δ_C 147.8, 132.0, 131.95, 130.4, 129.7, 129.3, 129.2, 128.9, 128.88, 128.7, 128.2, 128.1, 127.7, 127.4, 127.35, 126.8, 126.5, 125.7, 125.5, 125.0, 124.99, 124.5, 124.0, 123.7, 123.4, 123.3, 123.2, 123.1, 118.8, 118.4; HRMS (ESI-TOF, *m/z*): calcd for $C_{31}H_{19}N_2$ [M + H]$^+$, 419.1543; found, 419.1524.

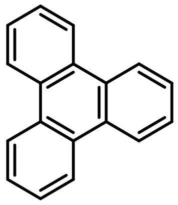

Triphenylene (5a)[8]

To a 10 mL Schlenk tube equipped with a magnetic stir bar was added 2-bromobenzoic acid **2a** (201 mg, 1.00 mmol, 2.00 equiv), DMF (4.0 mL), Cs_2CO_3 (163 mg, 0.500 mmol, 1.00 equiv), PPh_3 (26.2 mg, 0.10 mmol, 0.20 equiv), $Pd(OAc)_2$ (5.6 mg, 0.025 mmol, 0.050 equiv), and CuI (9.5 mg, 0.050 mmol, 0.10 equiv). The reaction mixture was stirred at 110 °C in an oil bath for about 8 h. The resulting mixture was concentrated and the residue was taken up in ethyl acetate. The organic layer was washed with brine, dried over Na_2SO_4 and concentrated. Purification of the crude product by column chromatography (silica gel; petroleum ether/ethyl acetate 60:1) afforded **5a** in 81% yield (91 mg).

White solid; mp 196–197 °C; ^{1}H NMR ($CDCl_3$, 400 MHz): δ_H 8.68–8 66 (m, 6H), 7.68–7.66 (m, 6H); ^{13}C NMR ($CDCl_3$, 101 MHz): δ_C 129.8, 127.2, 123.3; HRMS (ESI-TOF, *m/z*): calcd for $C_{18}H_{13}$ $[M + H]^+$, 229.1012; found, 229.1094.

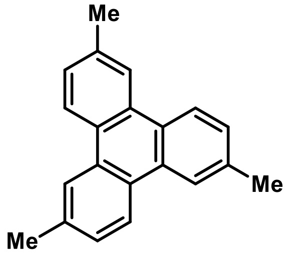

2,6,10-Trimethyltriphenylene (5b)[8]

From 2-bromo-4-methylbenzoic acid 2d: To a 10 mL Schlenk tube equipped with a magnetic stir bar was added 2-bromo-4-methylbenzoic acid **2d** (213.1 mg, 1.00 mmol, 2.00 equiv), DMF (4.0 mL), Cs_2CO_3 (163 mg, 0.500 mmol, 1.00 equiv), PPh_3 (26.2 mg, 0.10 mmol, 0.20 equiv), $Pd(OAc)_2$ (5.6 mg, 0.025 mmol, 0.050 equiv), and CuI (9.5 mg, 0.050 mmol, 0.10 equiv). The reaction mixture was stirred at 110 °C in an oil bath for about 8 h. The resulting mixture was concentrated and the residue was taken up in ethyl acetate. The organic layer was washed with brine, dried over Na_2SO_4 and concentrated. Purification of the crude product by column chromatography (silica gel; petroleum ether/ethyl acetate 60:1) afforded **5b** in 73% yield (99 mg).

From 2-bromo-5-methylbenzoic acid 2g: To a 10 mL Schlenk tube equipped with a magnetic stir bar was added 2-bromo-5-methylbenzoic acid **2g** (213.1 mg, 1.00 mmol, 2.00 equiv), DMF (4.0 mL), Cs_2CO_3 (163 mg, 0.500 mmol, 1.00 equiv), PPh_3 (26.2 mg, 0.10 mmol, 0.20 equiv), $Pd(OAc)_2$ (5.6 mg, 0.025 mmol, 0.050 equiv), and CuI (9.5 mg, 0.050 mmol, 0.10 equiv). The reaction mixture was stirred at 110 °C in an oil bath for about 8 h. The resulting mixture was concentrated and the residue was taken up in ethyl acetate. The organic layer was washed with brine, dried over Na_2SO_4 and concentrated. Purification of the crude product by column chromatography (silica gel; petroleum ether/ethyl acetate 60:1) afforded **5b** in 79% yield (107 mg).

White solid; mp 142 °C; ^1H NMR ($CDCl_3$, 400 MHz): δ_H 8.52–8.46 (m, 3H), 8.42–8.38 (m, 3H), 7.46–7.41 (m, 3H), 2.60–2.59 (m, 9H); ^{13}C NMR ($CDCl_3$, 101 MHz): δ_C 136.5, 136.3, 136.2, 129.8, 129.5, 129.3, 128.5, 128.4, 128.2, 127.7, 127.5, 127.2, 123.22, 123.17, 123.01, 122.99, 122.95, 21.8; HRMS (ESI-TOF, *m/z*): calcd for $C_{21}H_{19}$ $[M + H]^+$, 271.1481; found, 271.1512.

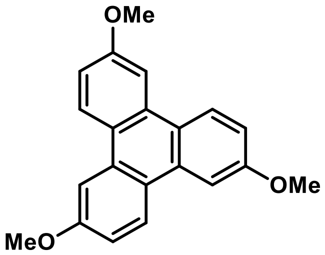

2,6,10-Trimethoxytriphenylene (5c)[9]

From 2-bromo-4-methoxybenzoic acid 2e: To a 10 mL Schlenk tube equipped with a magnetic stir bar was added 2-bromo-4-methoxybenzoic acid **2e** (231.0 mg, 1.00 mmol, 2.00 equiv), DMF (4.0 mL), Cs_2CO_3 (163 mg, 0.500 mmol, 1.00 equiv), PPh_3 (26.2 mg, 0.10 mmol, 0.20 equiv), $Pd(OAc)_2$ (5.6 mg, 0.025 mmol, 0.050 equiv), and CuI (9.5 mg, 0.050 mmol, 0.10 equiv). The reaction mixture was stirred at 110 °C in an oil bath for about 8 h. The resulting mixture was concentrated and the residue was taken up in ethyl acetate. The organic layer was washed with brine, dried over Na_2SO_4 and concentrated. Purification of the crude product by column chromatography (silica gel; petroleum ether/ethyl acetate 60:1) afforded **5c** in 84% yield (133 mg).

From 2-bromo-5-methoxybenzoic acid 2h: To a 10 mL Schlenk tube equipped with a magnetic stir bar was added 2-bromo-5-methoxybenzoic acid **2h** (231.0 mg, 1.00 mmol, 2.00 equiv), DMF (4.0 mL), Cs_2CO_3 (163 mg, 0.500 mmol, 1.00 equiv), PPh_3 (26.2 mg, 0.10 mmol, 0.20 equiv), $Pd(OAc)_2$ (5.6 mg,

0.025 mmol, 0.050 equiv), and CuI (9.5 mg, 0.050 mmol, 0.10 equiv). The reaction mixture was stirred at 110 °C in an oil bath for about 8 h. The resulting mixture was concentrated and the residue was taken up in ethyl acetate. The organic layer was washed with brine, dried over Na_2SO_4 and concentrated. Purification of the crude product by column chromatography (silica gel; petroleum ether/ethyl acetate 60:1) afforded **5c** in 77% yield (122 mg).

White solid; mp 126–128 °C; ^{1}H NMR ($CDCl_3$, 400 MHz): δ_H 8.49–8.41 (m, 3H), 7.95–7.92 (m, 3H), 7.27–7.24 (m, 2H), 7.20 (dd, J = 8.8, 2.4 Hz, 1H), 4.02–4.00 (m, 9H); ^{13}C NMR ($CDCl_3$, 101 MHz): δ_C 158.8, 158.2, 158.0, 131.3, 130.2, 129.8, 125.0, 124.4, 124.37, 124.3, 124.0, 123.0, 115.7, 115.4, 114.9, 106.1, 105.3, 55.5, 55.49, 55.4; HRMS (ESI-TOF, *m/z*): calcd for $C_{21}H_{19}O_3$ [M + H]$^+$, 319.1329; found, 319.1398.

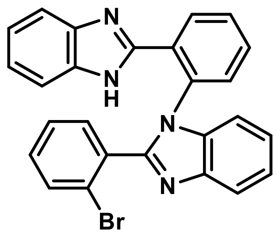

1-(2-(1*H*-Benzo[*d*]imidazol-2-yl)phenyl)-2-(2-bromophenyl)-1*H*-benzo[d]imidazole (6a)

To a 10 mL Schlenk tube equipped with a magnetic stir bar was added 2-(2-bromophenyl)-1*H*-benzo[*d*]imidazole **1a** (136.5 mg, 0.500 mmol, 1.00 equiv), *o*-bromobenzoic acid **2a** (150.7 mg, 0.750 mmol, 1.50 equiv), DMF (4.0 mL), Cs_2CO_3 (163 mg, 0.500 mmol, 1.00 equiv), PPh_3 (26.2 mg, 0.10 mmol, 0.20 equiv) and CuI (9.5 mg, 0.050 mmol, 0.10 equiv). The reaction mixture was stirred at 110 °C in an oil bath for about 8 h. The resulting mixture was concentrated and the residue was taken up in ethyl acetate. The organic layer was washed with brine, dried over Na_2SO_4 and concentrated. Purification of the crude product by column chromatography (silica gel; petroleum ether/ethyl acetate 10:1) afforded **6a** in 37% yield (42 mg).

White solid; mp 267–269 °C; ^{1}H NMR ($CDCl_3$, 400 MHz): δ_H 12.60 (s, 1H), 7.90–7.88 (m, 1H), 7.75 (d, J = 8.0 Hz, 1H), 7.67–7.65 (m, 2H), 7.60–7.57 (m, 2H), 7.37 (s, 2H), 7.28–7.22 (m, 4H), 7.07–7.05 (m, 2H) 7.00–6.96 (m, 2H); ^{13}C NMR (DMSO-D_6, 101 MHz): δ_C 151.2, 148.4, 143.6, 142.6, 136.1, 134.3, 133.5, 132.3, 132.0, 131.9, 131.3, 130.6, 130.5, 130.0, 129.5, 128.5, 126.7, 123.1, 122.73, 122.68, 122.1, 121.5, 119.4, 119.1, 111.3, 110.2, 39.5; HRMS (ESI-TOF, *m/z*): calcd for $C_{26}H_{17}BrN_4Na$ [M + Na]$^+$, 487.0529; found, 487.0521.

V. NMR spectra

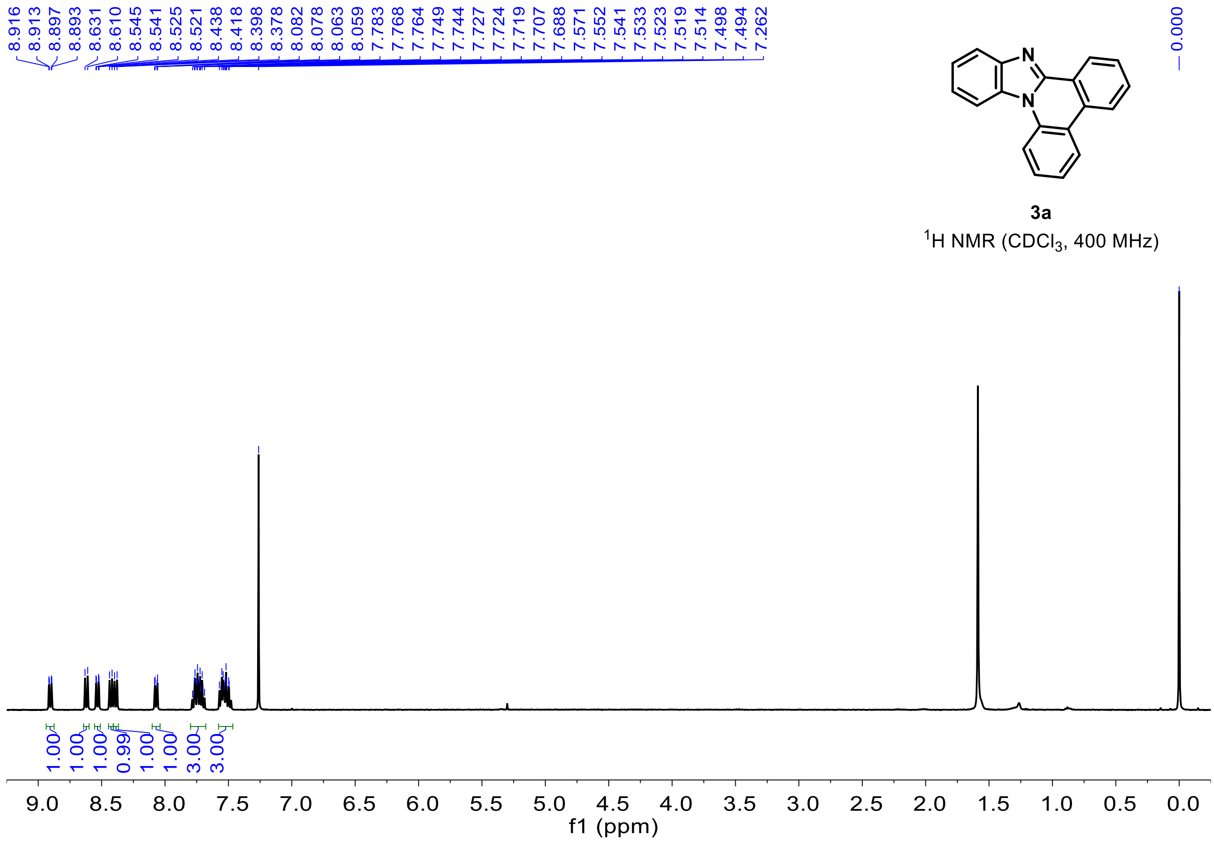

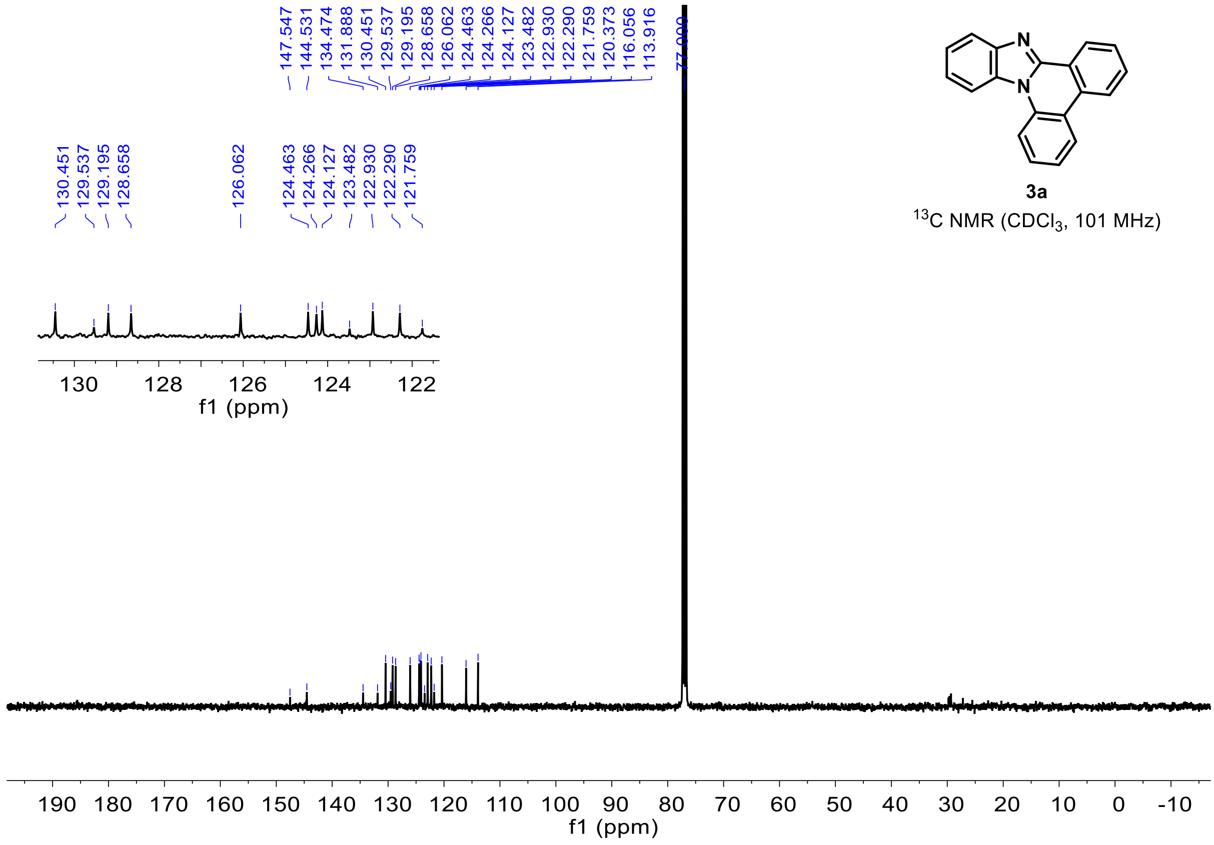

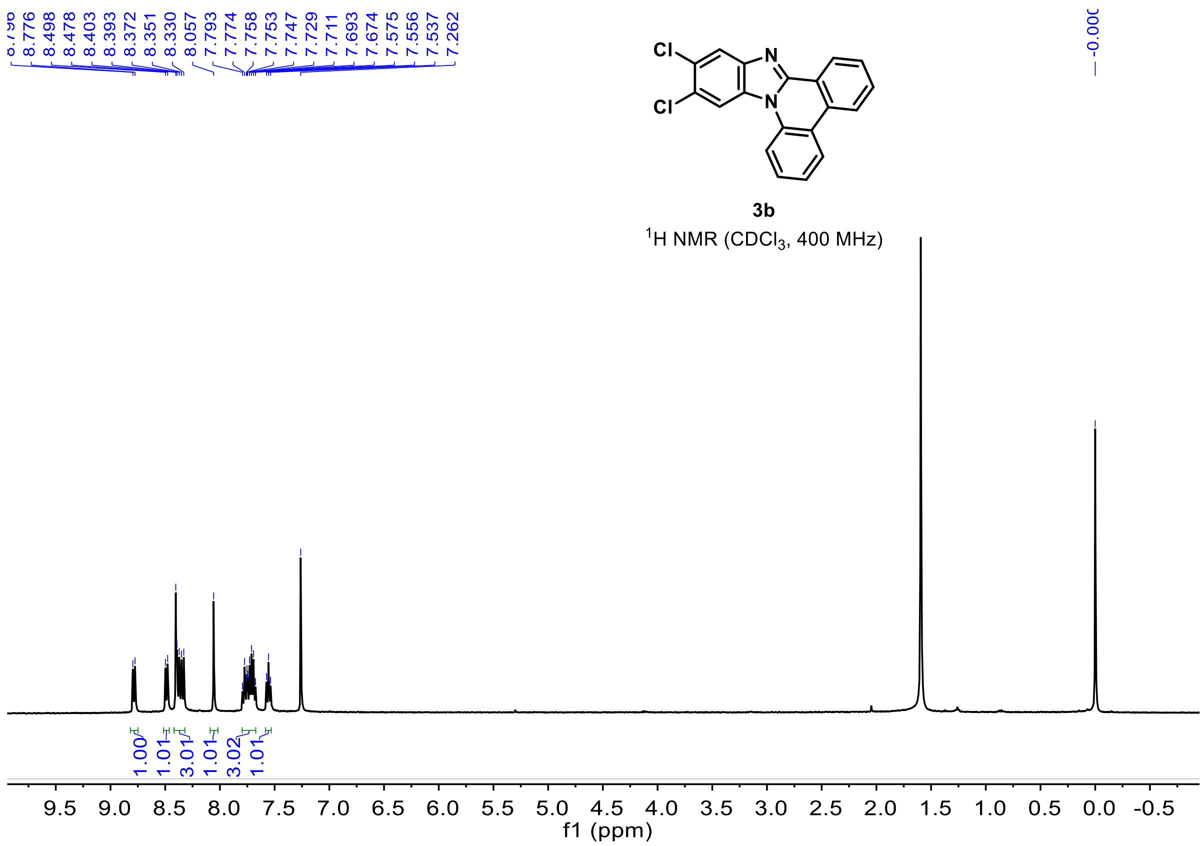
8.776
8.498
8.478
8.403
8.393
8.372
8.351
8.330
8.057
7.793
7.774
7.758
7.753
7.747
7.729
7.711
7.693
7.674
7.575
7.556
7.537
7.262
-0.000
Cl
Cl
N
N
3b
^{1}H NMR ($CDCl_3$, 400 MHz)
1.00
1.01
3.01
1.01
3.02
1.01
f1 (ppm)

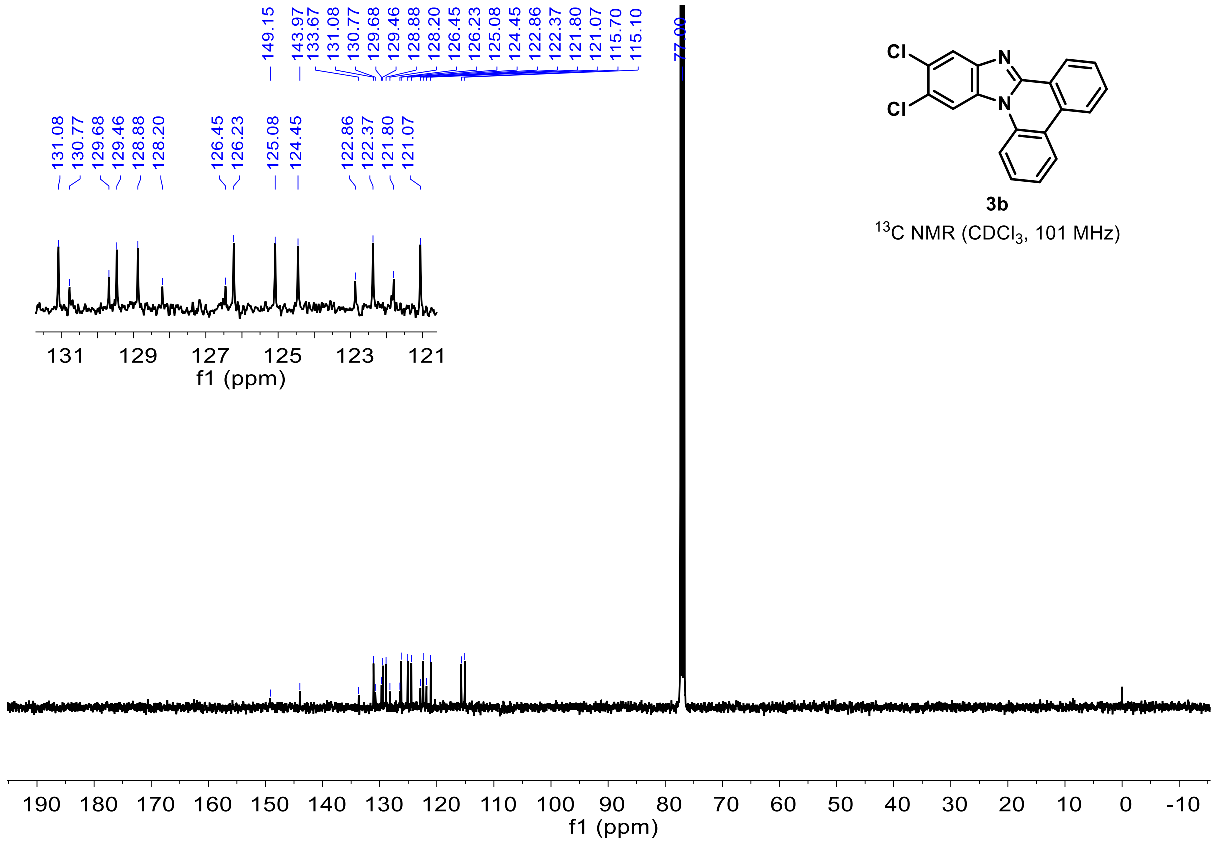
149.15
143.97
133.67
131.08
130.77
129.68
129.46
128.88
128.20
126.45
126.23
125.08
124.45
122.86
122.37
121.80
121.07
115.70
115.10
77.00
Cl
Cl
N
N
3b
^{13}C NMR ($CDCl_3$, 101 MHz)
f1 (ppm)

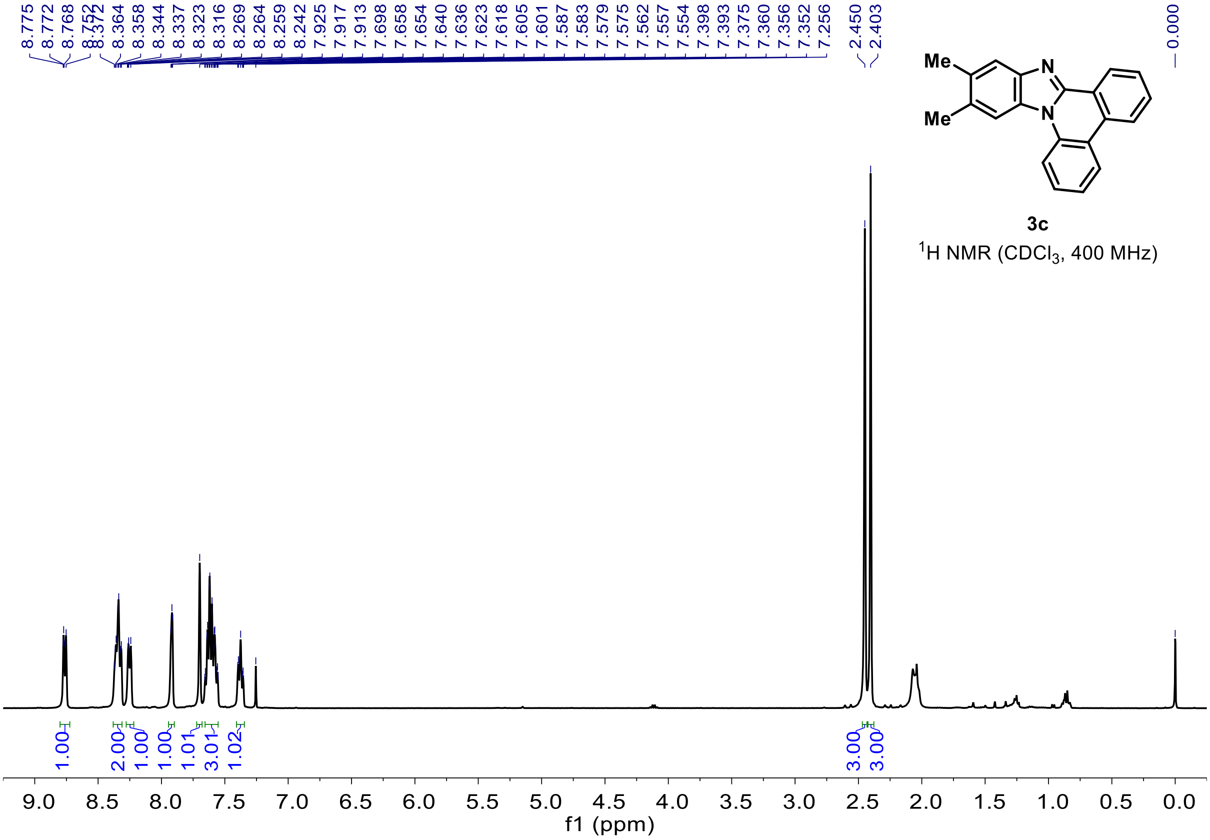

3c

^{1}H NMR ($CDCl_3$, 400 MHz)

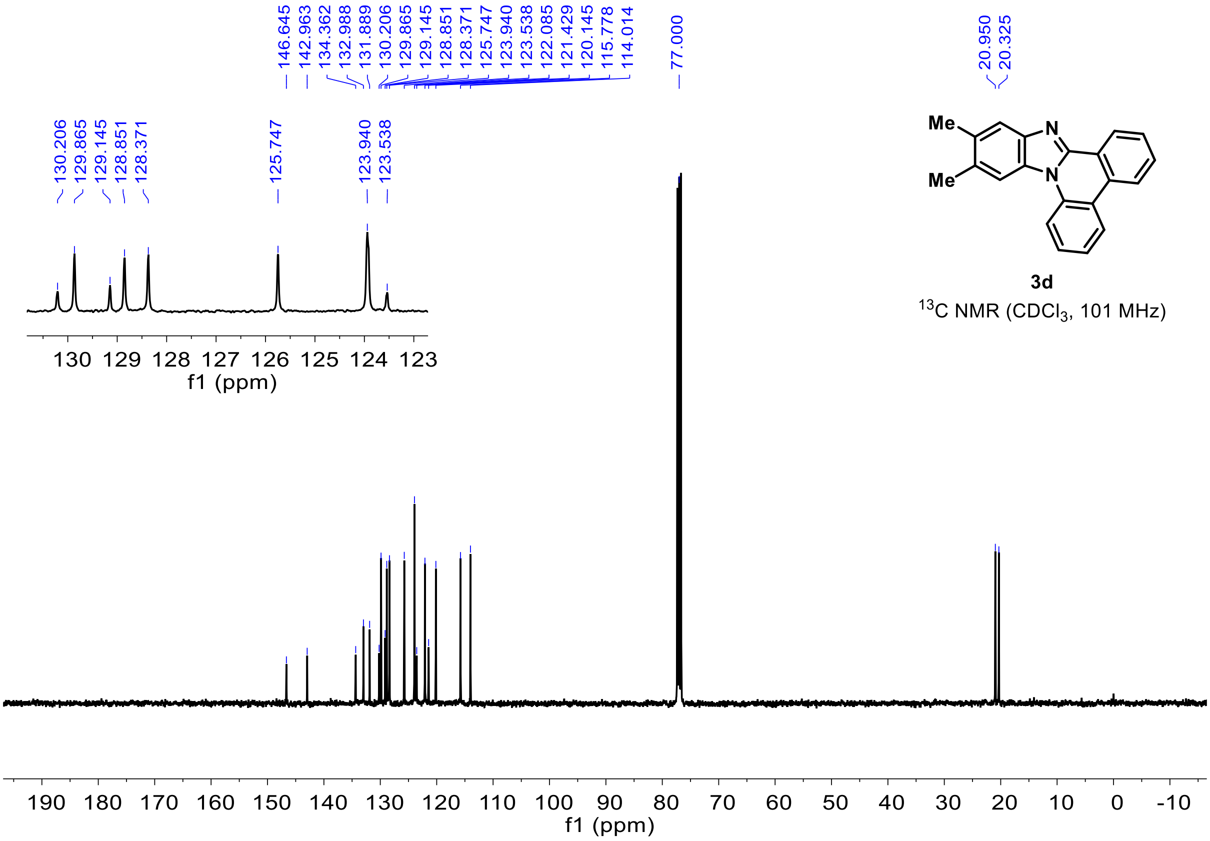

3d

^{13}C NMR ($CDCl_3$, 101 MHz)

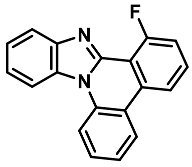

3d

^{1}H NMR (CDCl$_3$, 400 MHz)

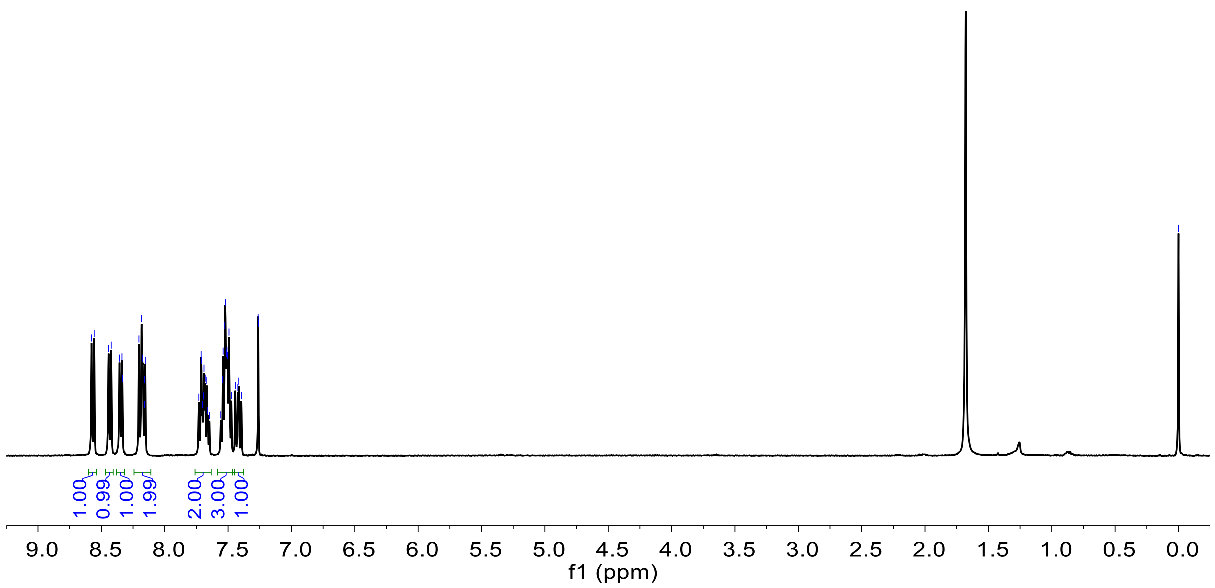

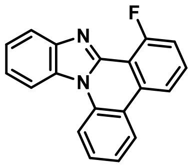

3d

^{13}C NMR (CDCl$_3$, 101 MHz)

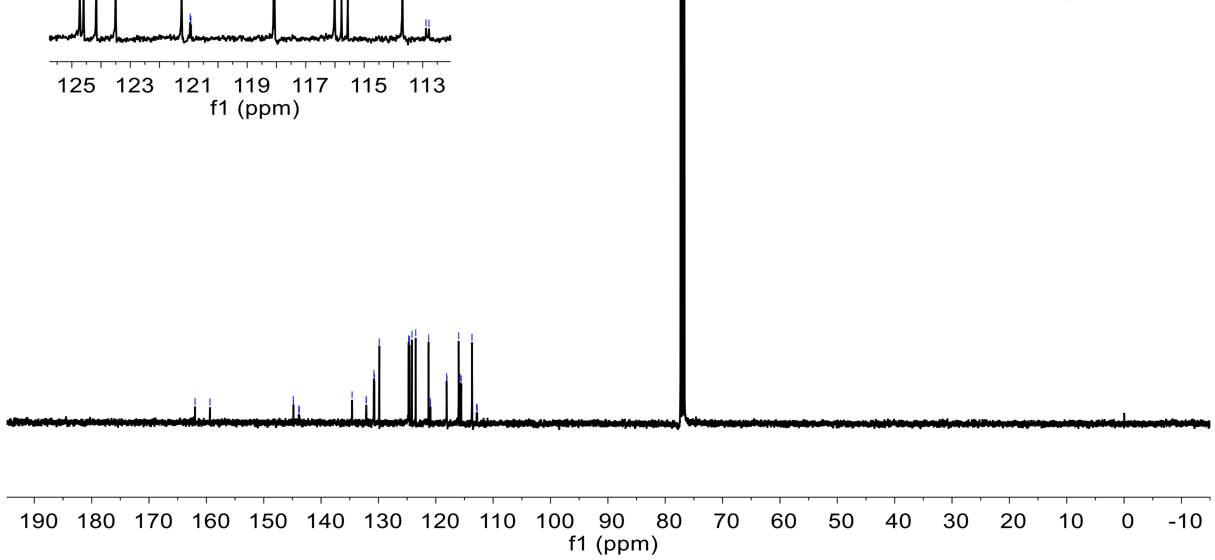

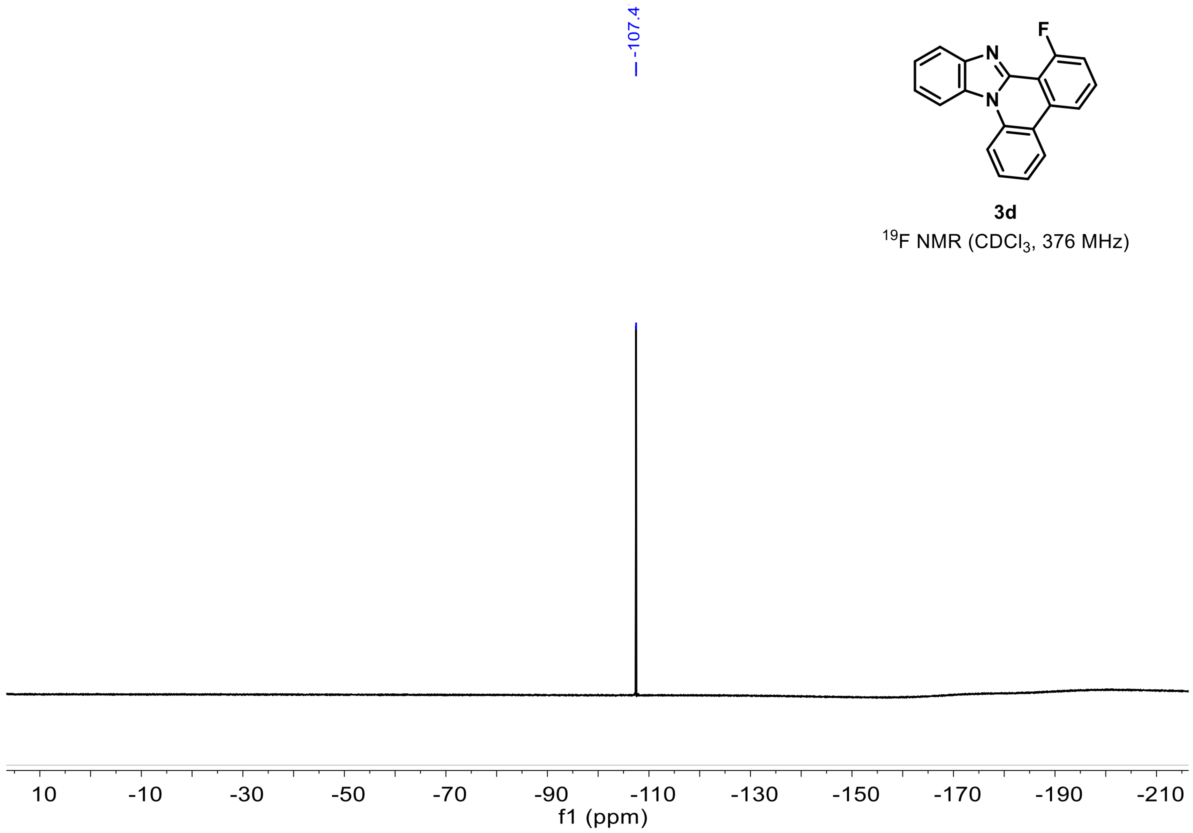

3d

^{19}F NMR ($CDCl_3$, 376 MHz)

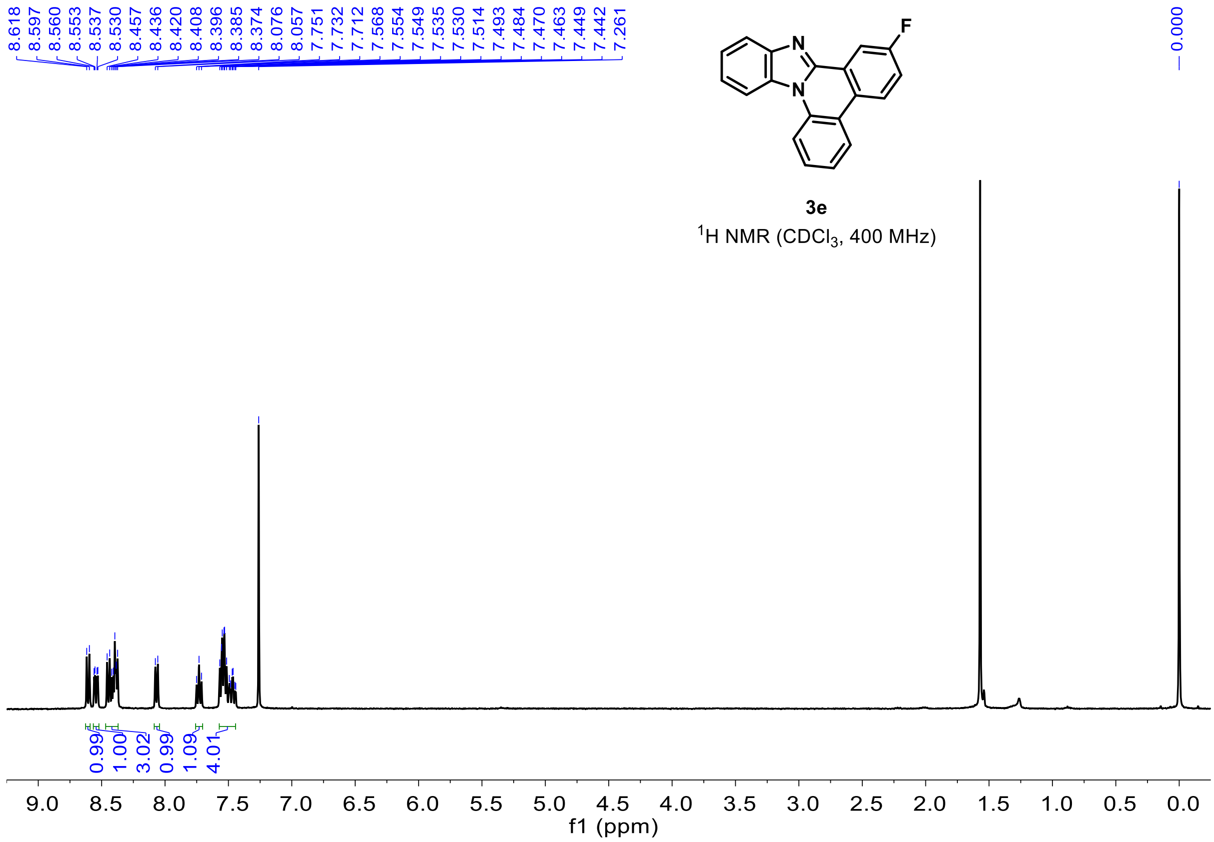

3e

^{1}H NMR ($CDCl_3$, 400 MHz)

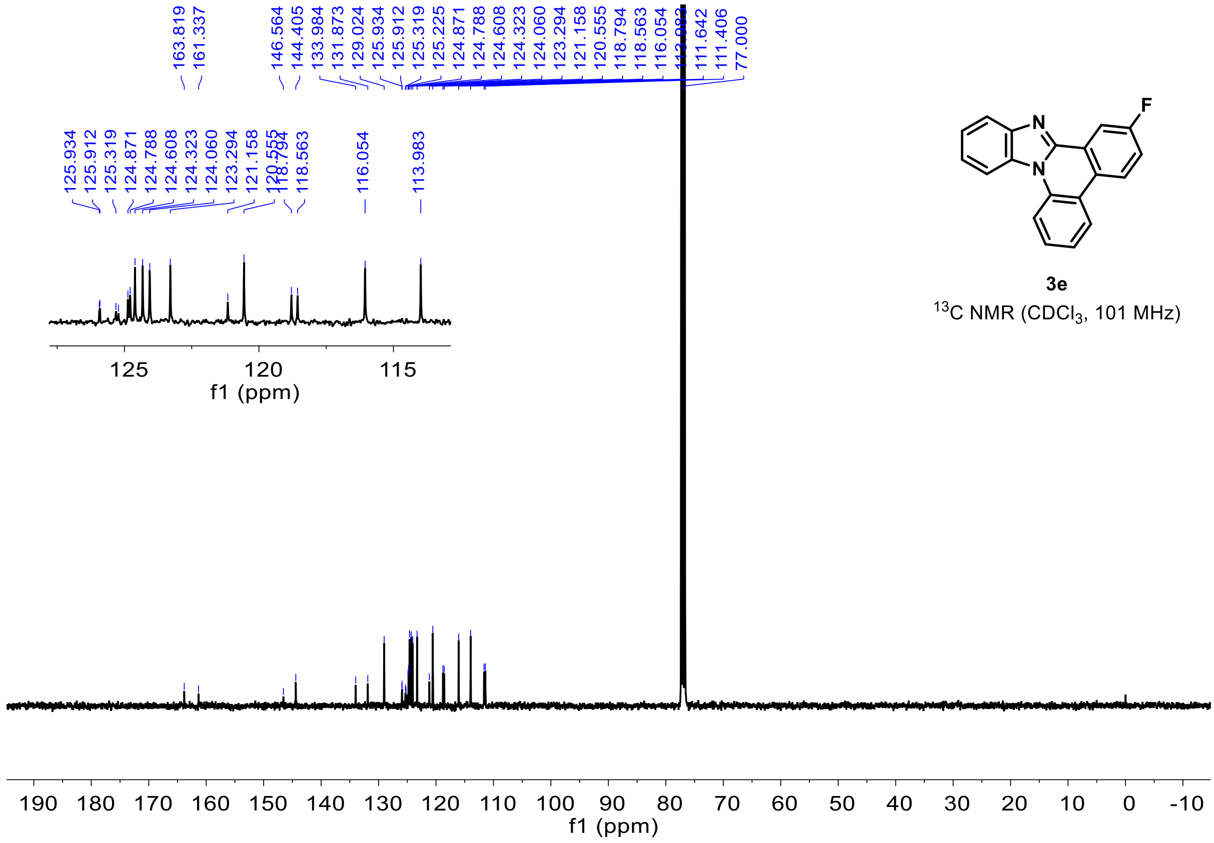

3e

^{13}C NMR ($CDCl_3$, 101 MHz)

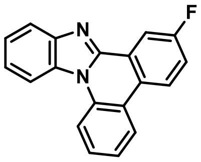

3e

^{19}F NMR ($CDCl_3$, 376 MHz)

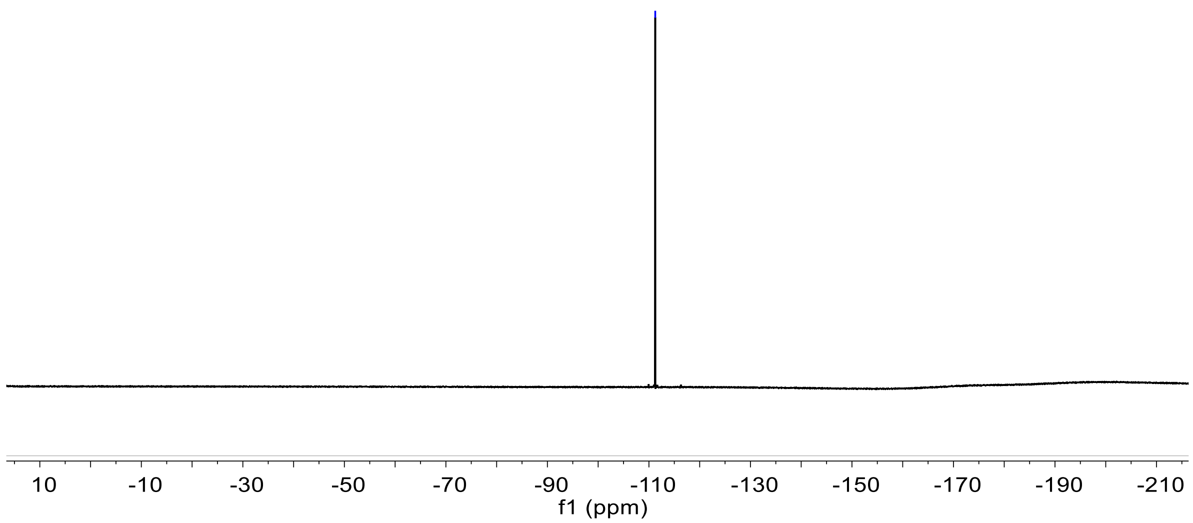

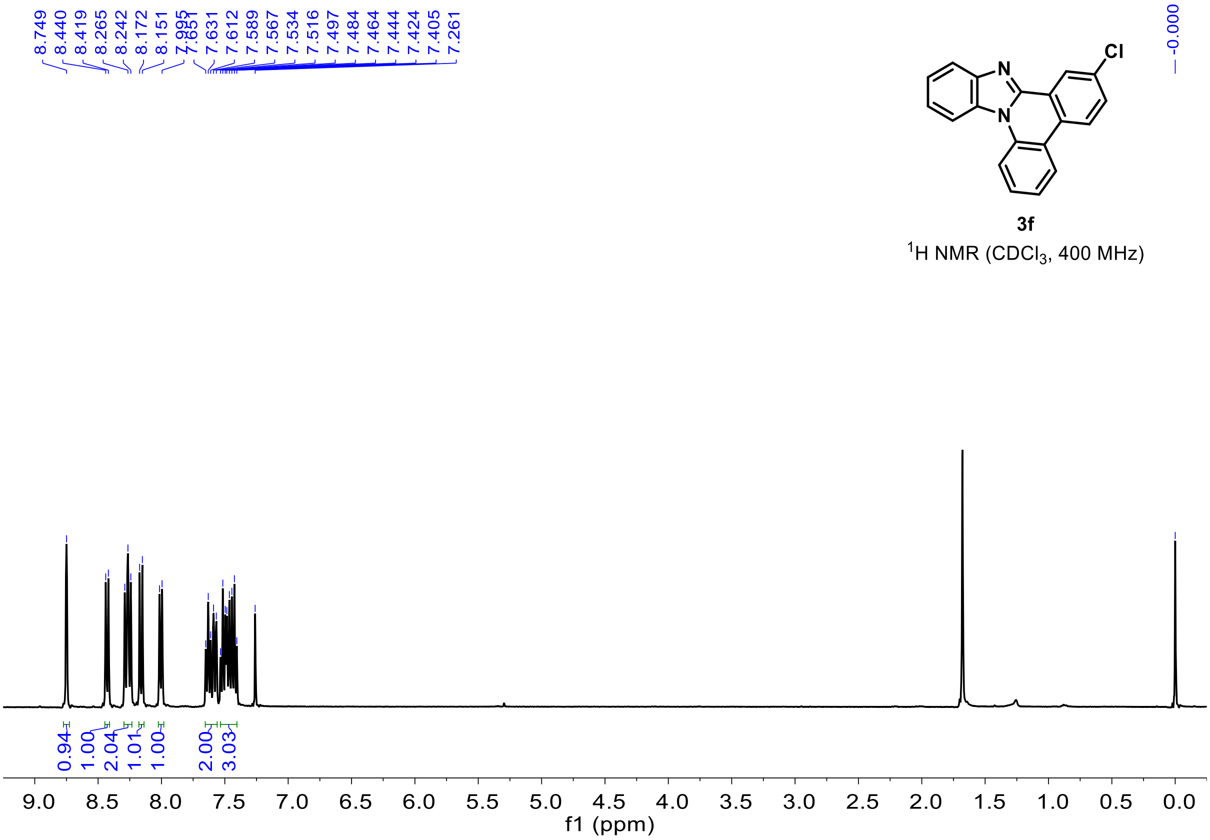

3f

^{1}H NMR ($CDCl_3$, 400 MHz)

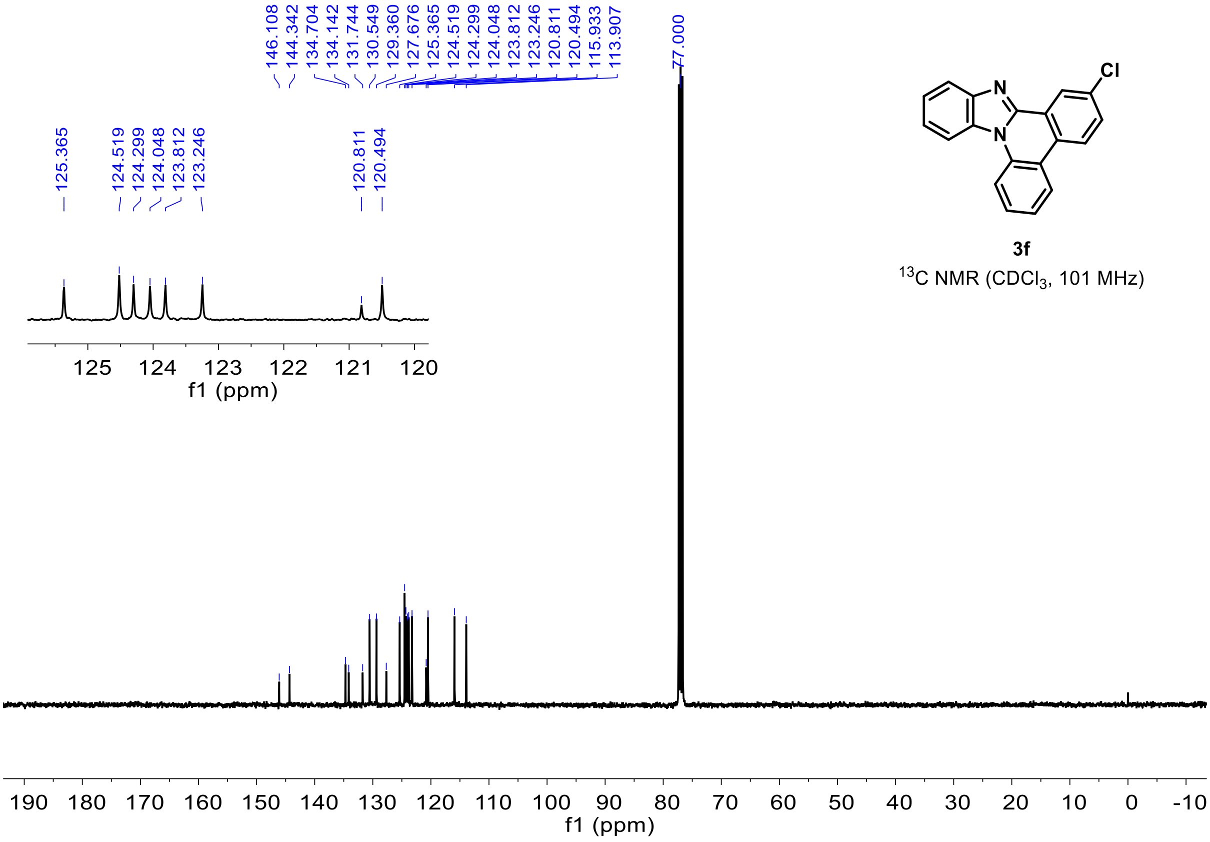

3f

^{13}C NMR ($CDCl_3$, 101 MHz)

3g

^{1}H NMR ($CDCl_3$, 400 MHz)

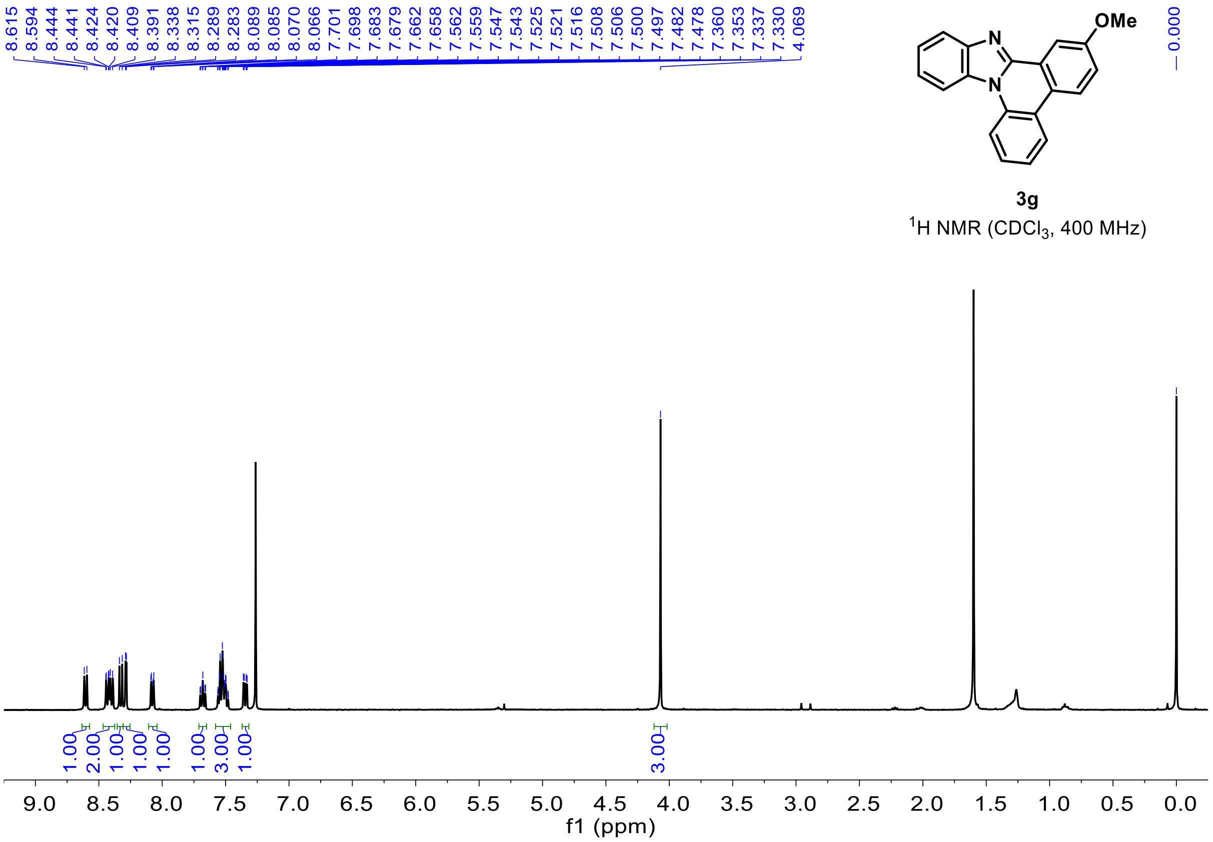

3g

^{13}C NMR ($CDCl_3$, 101 MHz)

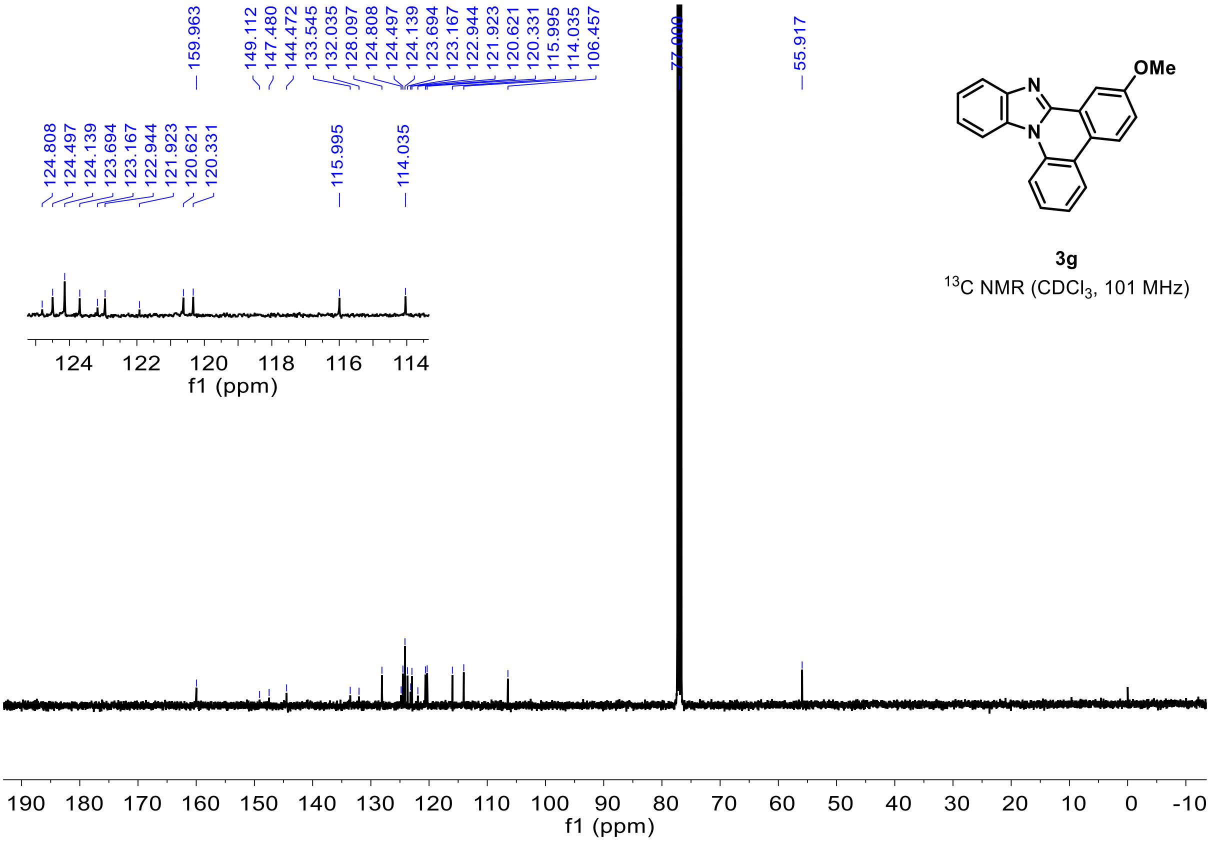

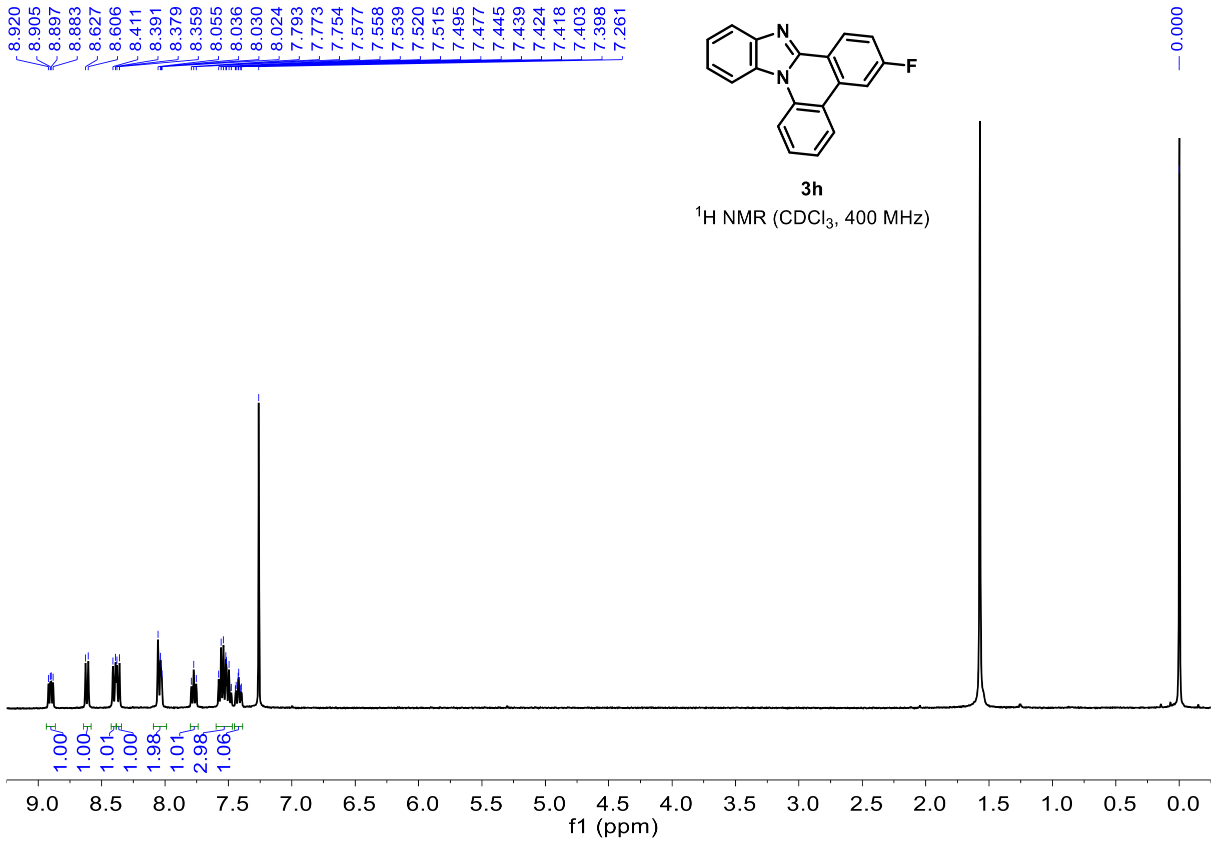

3h

^{1}H NMR ($CDCl_3$, 400 MHz)

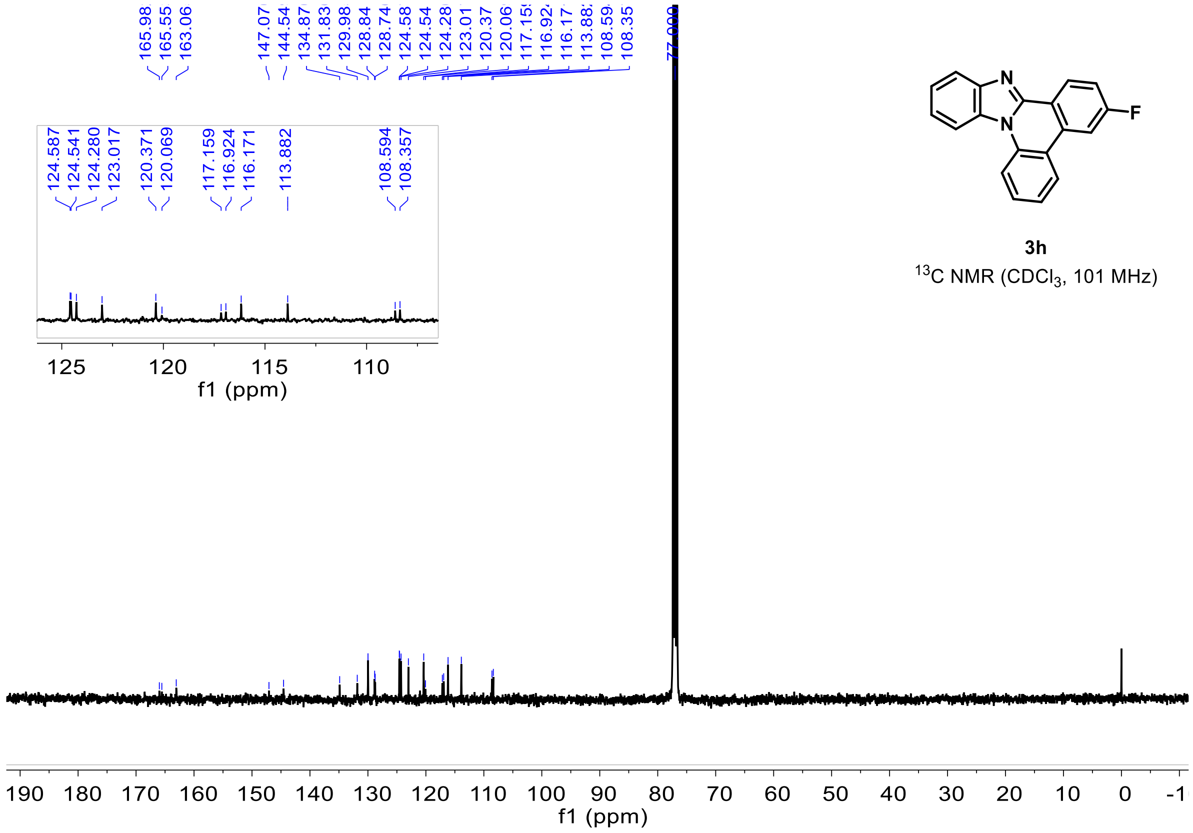

3h

^{13}C NMR ($CDCl_3$, 101 MHz)

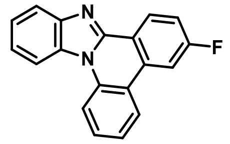

3h

^{19}F NMR ($CDCl_3$, 376 MHz)

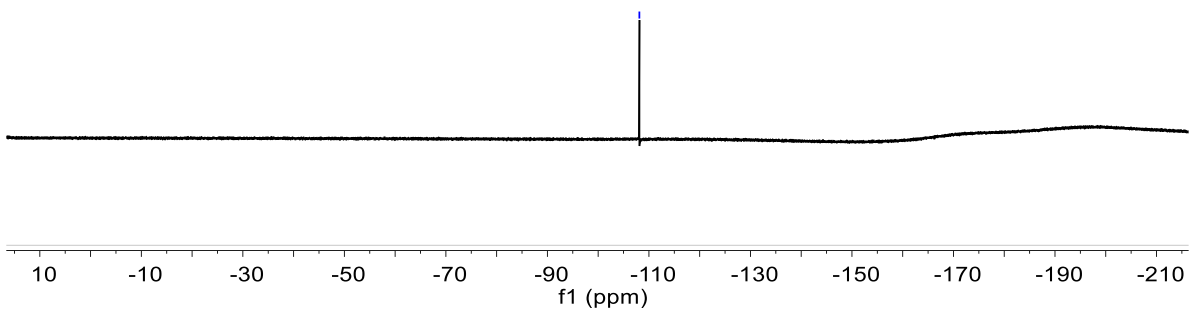

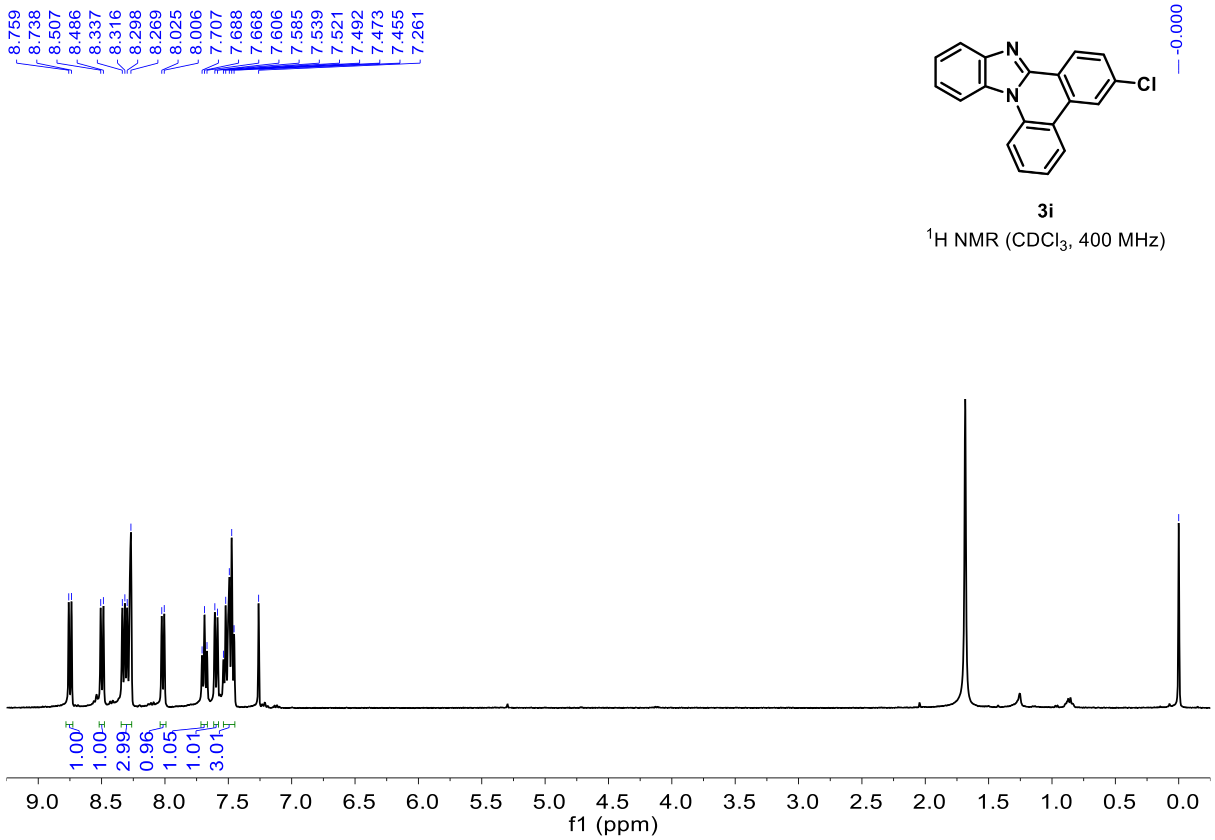

3i

^{1}H NMR ($CDCl_3$, 400 MHz)

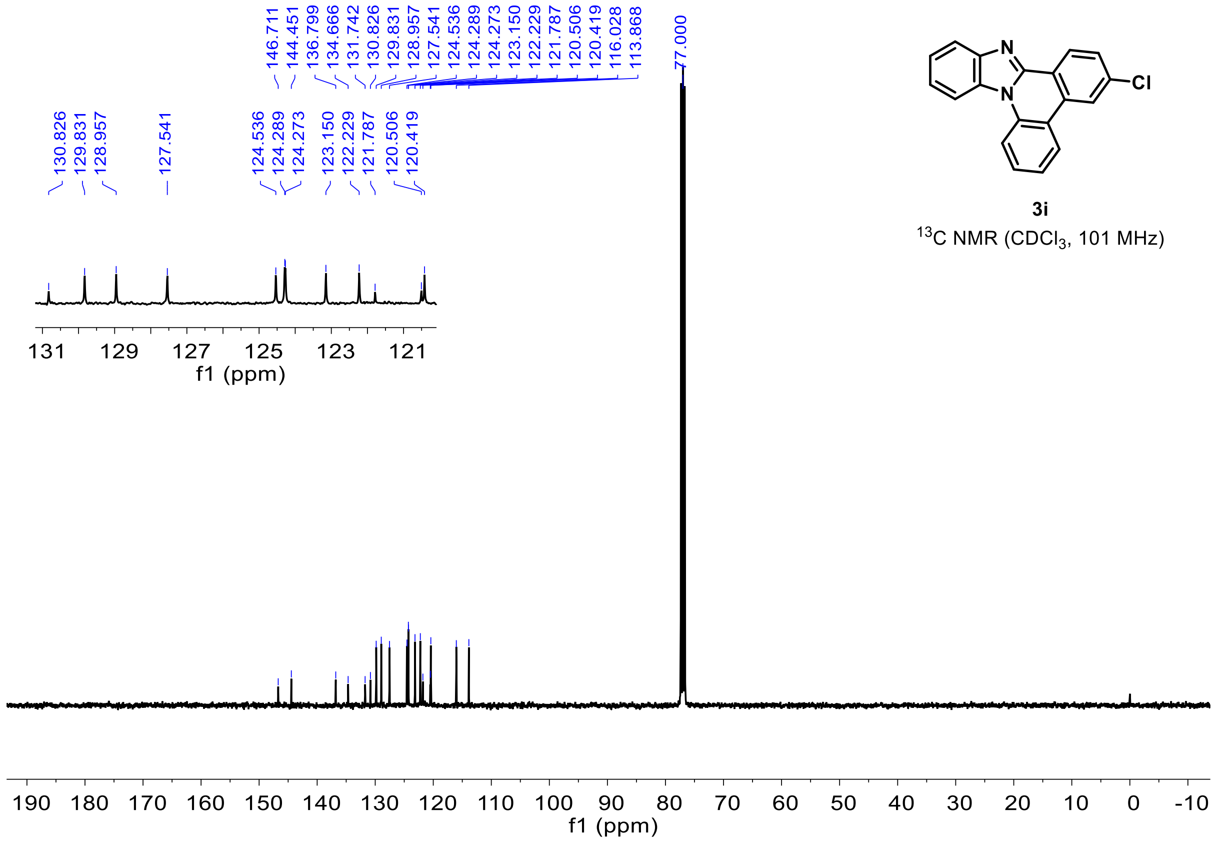

3i

^{13}C NMR ($CDCl_3$, 101 MHz)

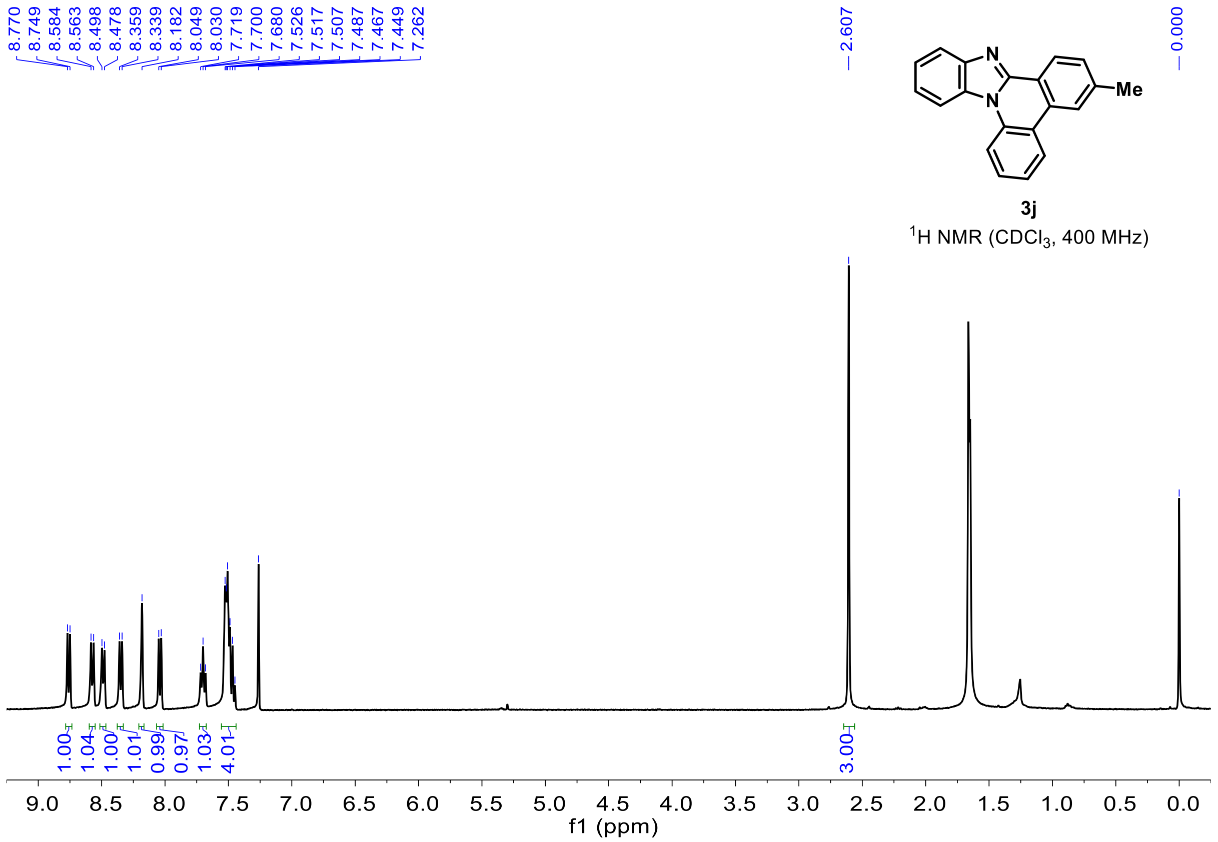
8.770
8.749
8.584
8.563
8.498
8.478
8.359
8.339
8.182
8.049
8.030
7.719
7.700
7.680
7.526
7.517
7.507
7.487
7.467
7.449
7.262
2.607
0.000
N
N
Me
3j
^{1}H NMR (CDCl$_3$, 400 MHz)
1.00
1.04
1.00
1.01
0.99
0.97
1.03
4.01
3.00
9.0 8.5 8.0 7.5 7.0 6.5 6.0 5.5 5.0 4.5 4.0 3.5 3.0 2.5 2.0 1.5 1.0 0.5 0.0
f1 (ppm)

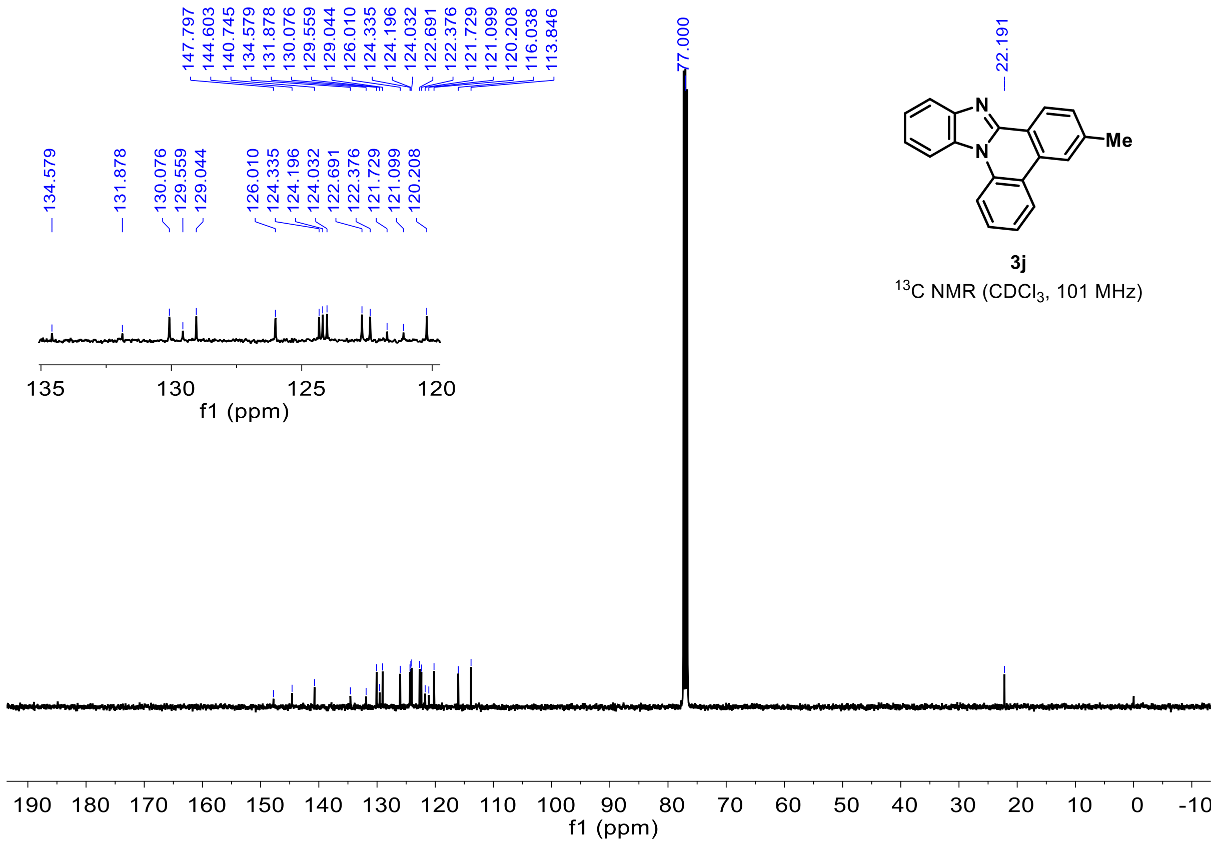
147.797
144.603
140.745
134.579
131.878
130.076
129.559
129.044
126.010
124.335
124.196
124.032
122.691
122.376
121.729
121.099
120.208
116.038
113.846
77.000
22.191
N
N
Me
3j
^{13}C NMR (CDCl$_3$, 101 MHz)
134.579
131.878
130.076
129.559
129.044
126.010
124.335
124.196
124.032
122.691
122.376
121.729
121.099
120.208
135 130 125 120
f1 (ppm)
190 180 170 160 150 140 130 120 110 100 90 80 70 60 50 40 30 20 10 0 -10
f1 (ppm)

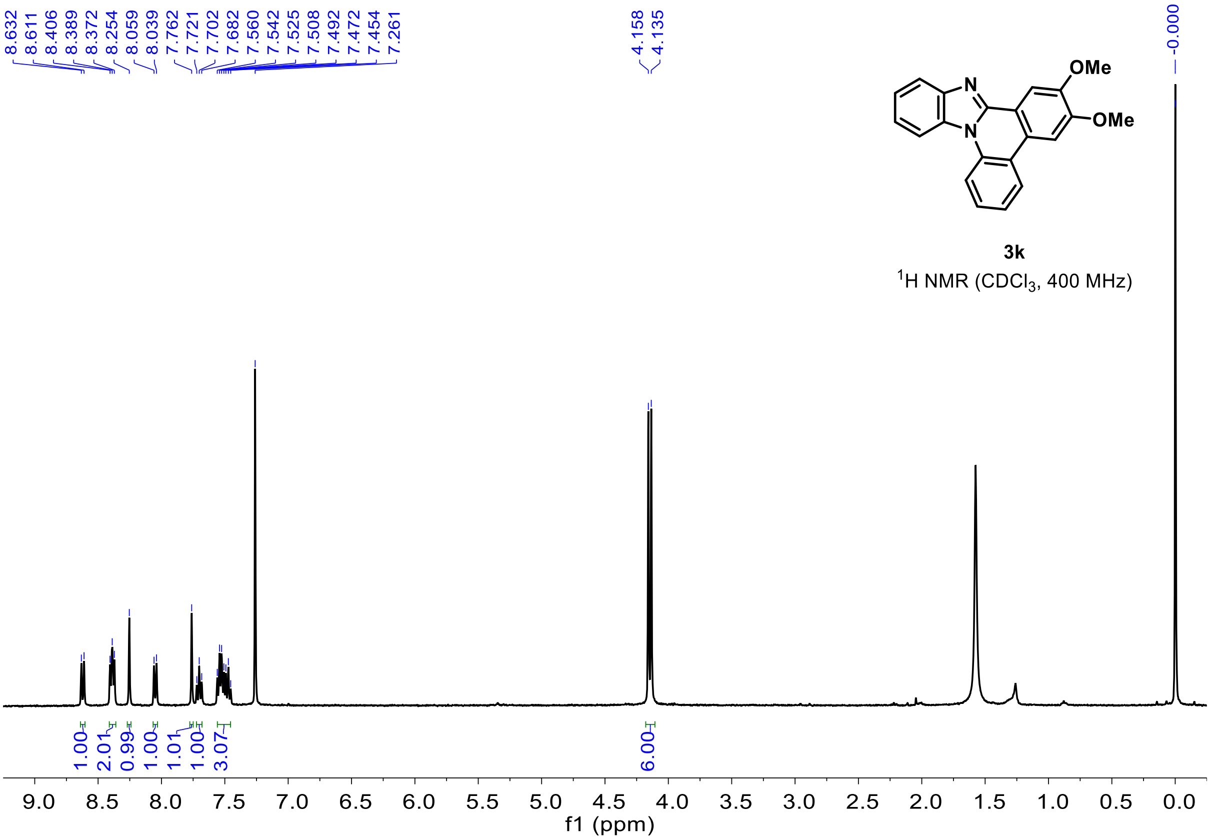

3k

^{1}H NMR ($CDCl_3$, 400 MHz)

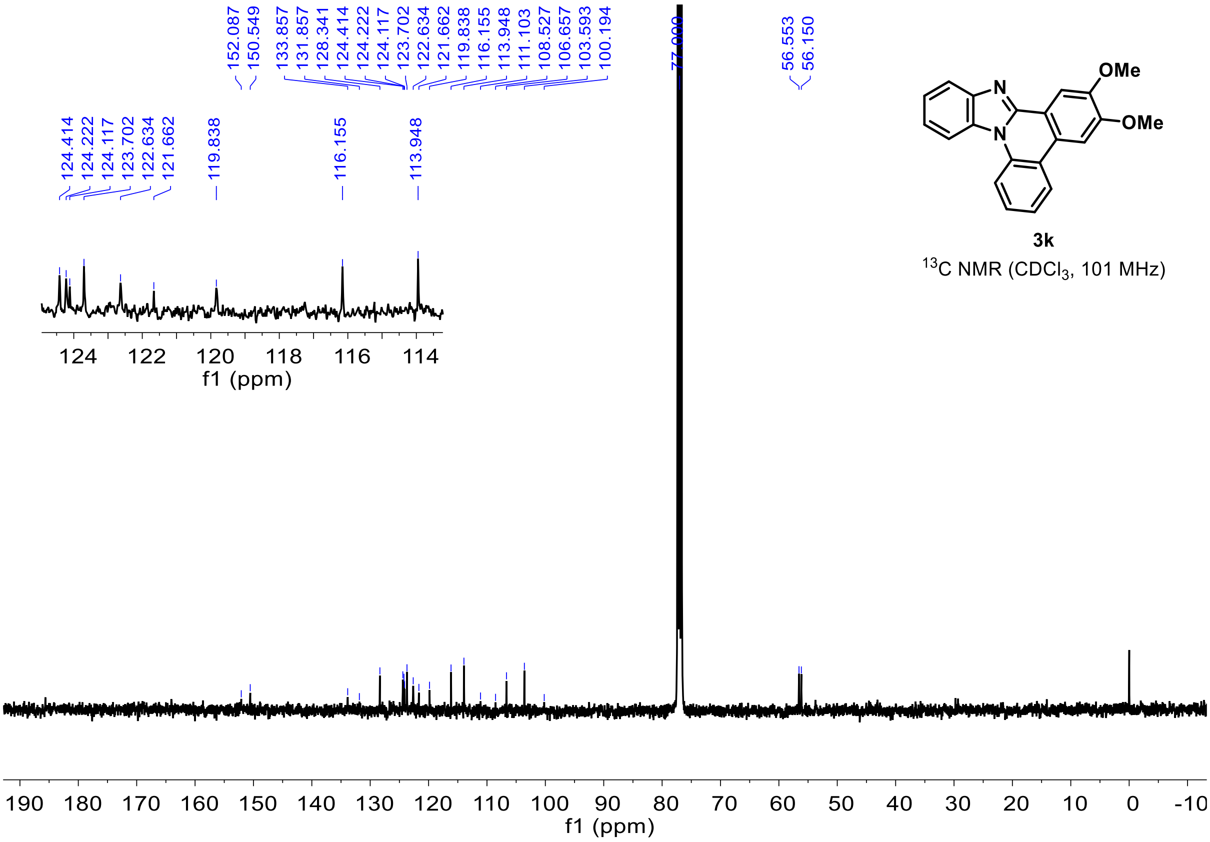

3k

^{13}C NMR ($CDCl_3$, 101 MHz)

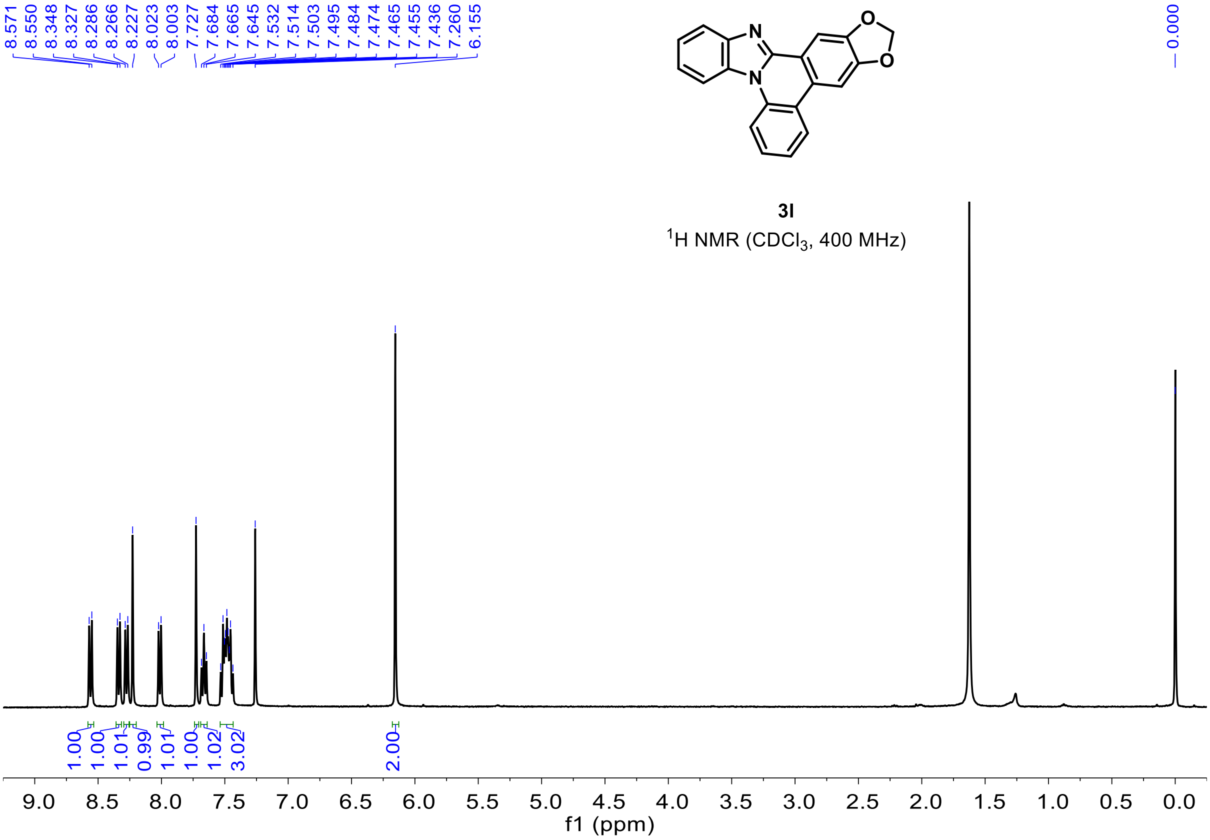

3l

^{1}H NMR ($CDCl_3$, 400 MHz)

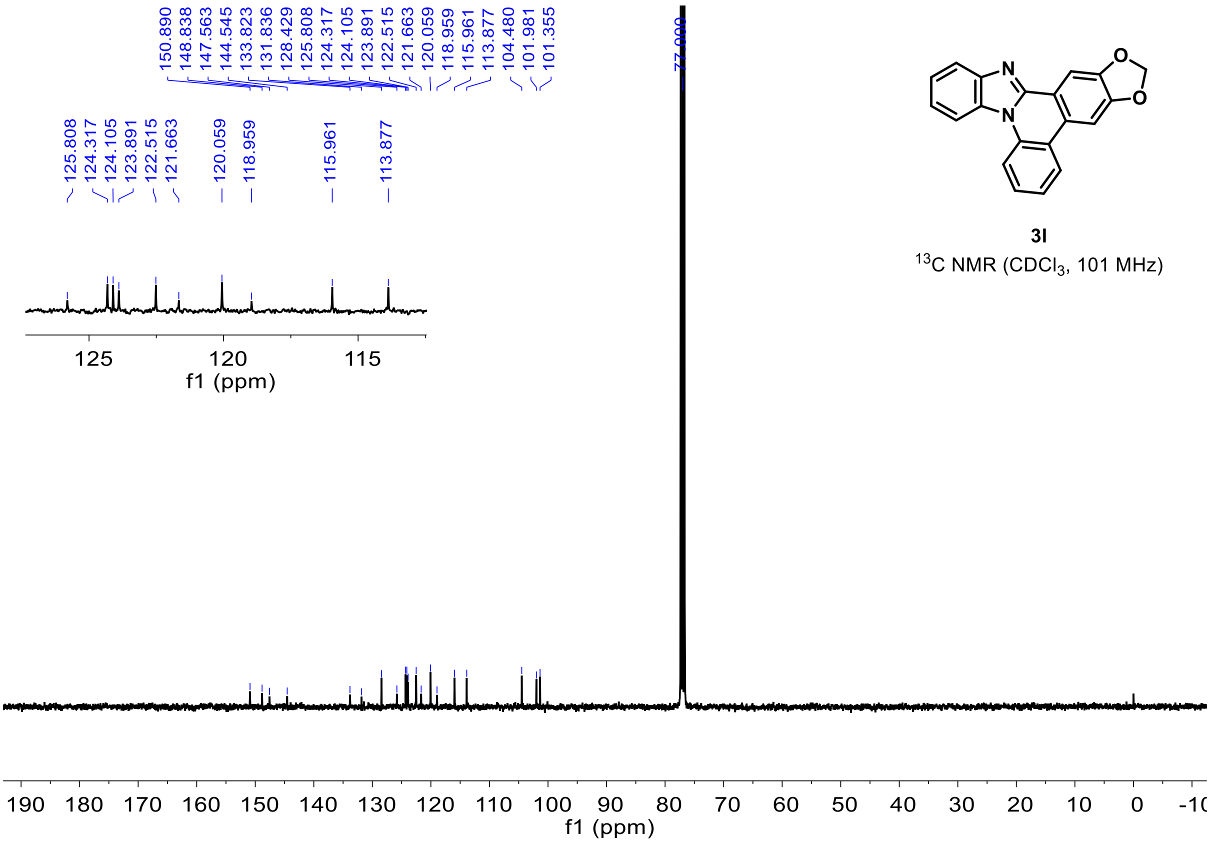

3l

^{13}C NMR ($CDCl_3$, 101 MHz)

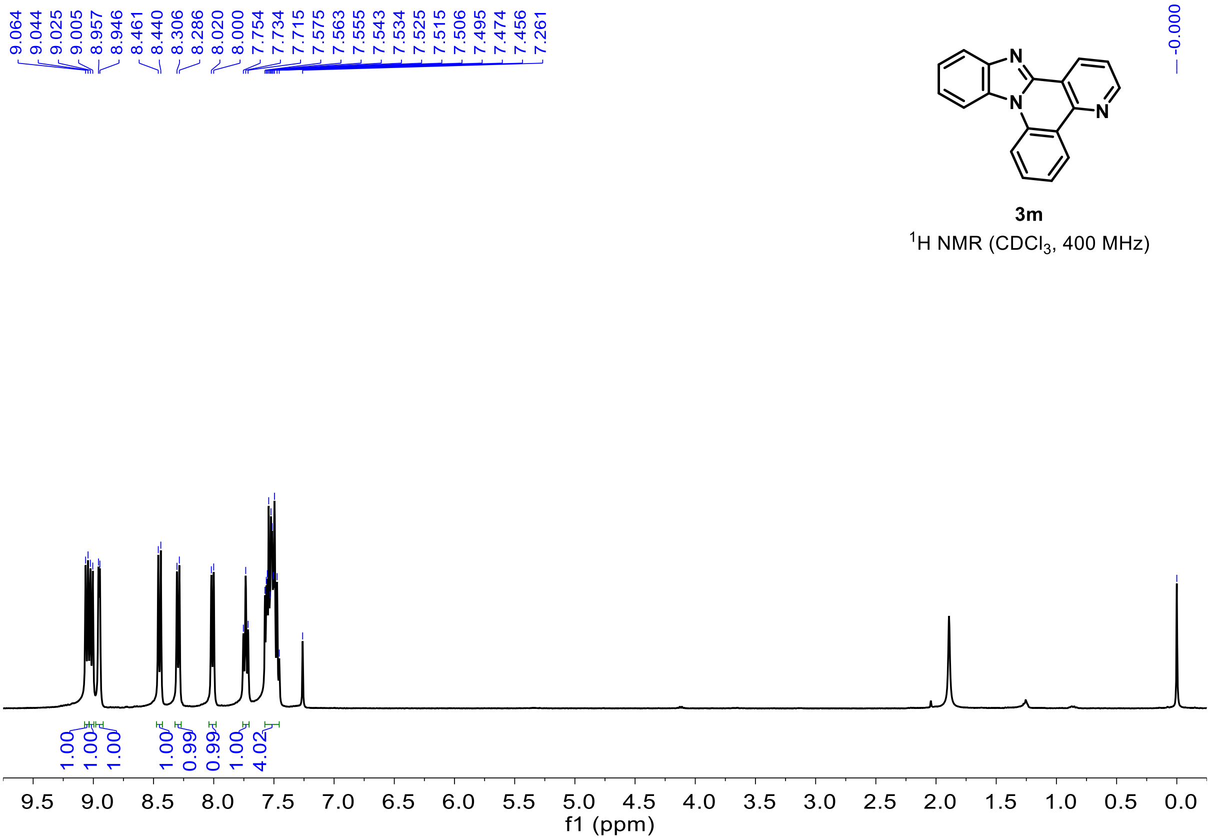

3m

^{1}H NMR ($CDCl_3$, 400 MHz)

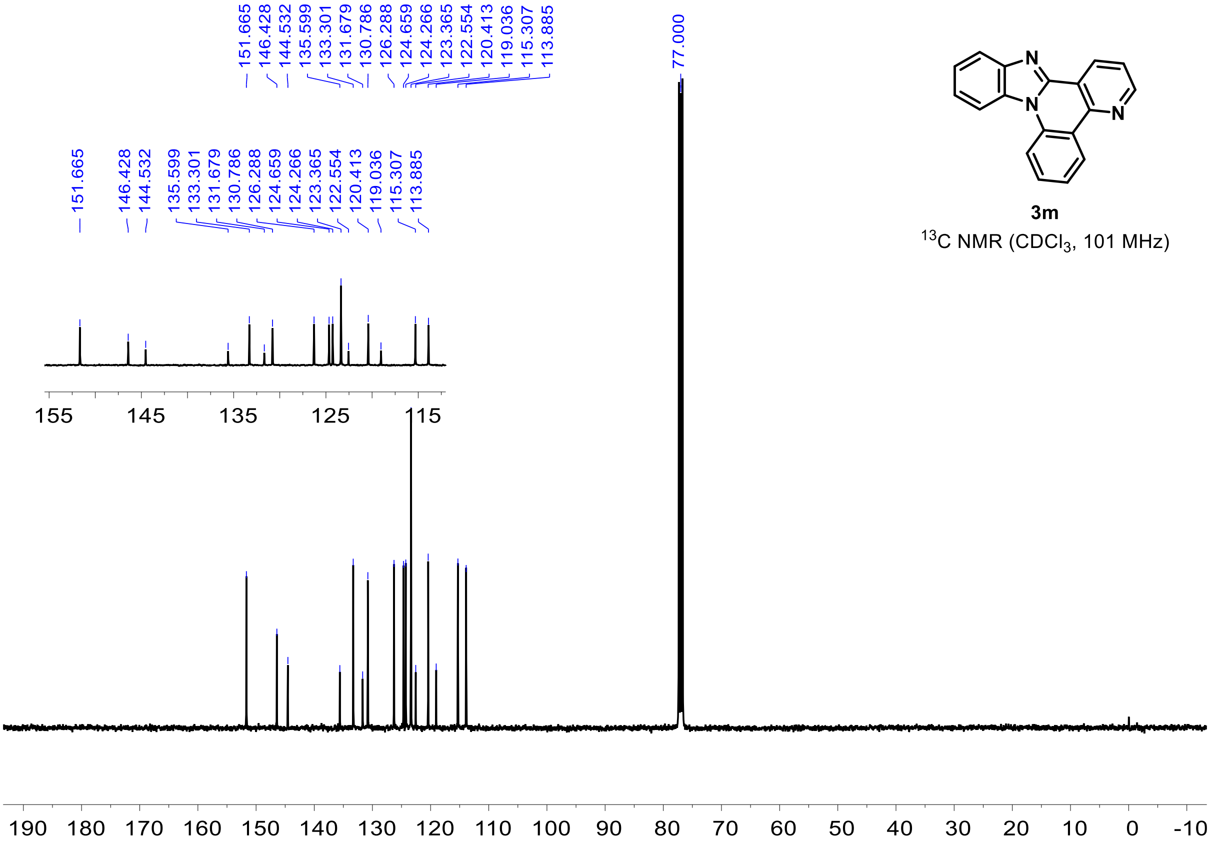

3m

^{13}C NMR ($CDCl_3$, 101 MHz)

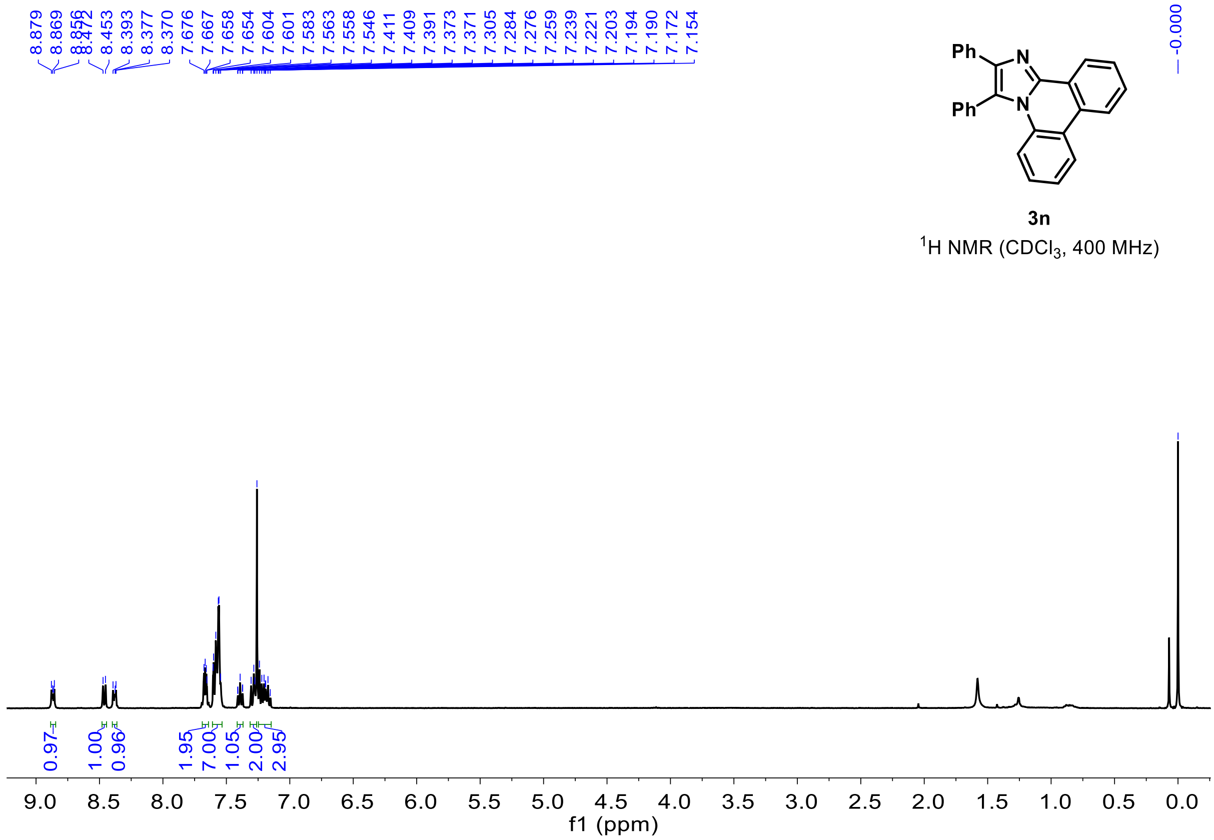
8.879
8.869
8.856
8.472
8.453
8.393
8.377
8.370
7.676
7.667
7.658
7.654
7.604
7.601
7.583
7.563
7.558
7.546
7.411
7.409
7.391
7.373
7.371
7.305
7.284
7.276
7.259
7.239
7.221
7.203
7.194
7.190
7.172
7.154
-0.000
Ph
Ph
N
N
3n
1H NMR (CDCl3, 400 MHz)
0.97
1.00
0.96
1.95
7.00
1.05
2.00
2.95
9.0 8.5 8.0 7.5 7.0 6.5 6.0 5.5 5.0 4.5 4.0 3.5 3.0 2.5 2.0 1.5 1.0 0.5 0.0
f1 (ppm)

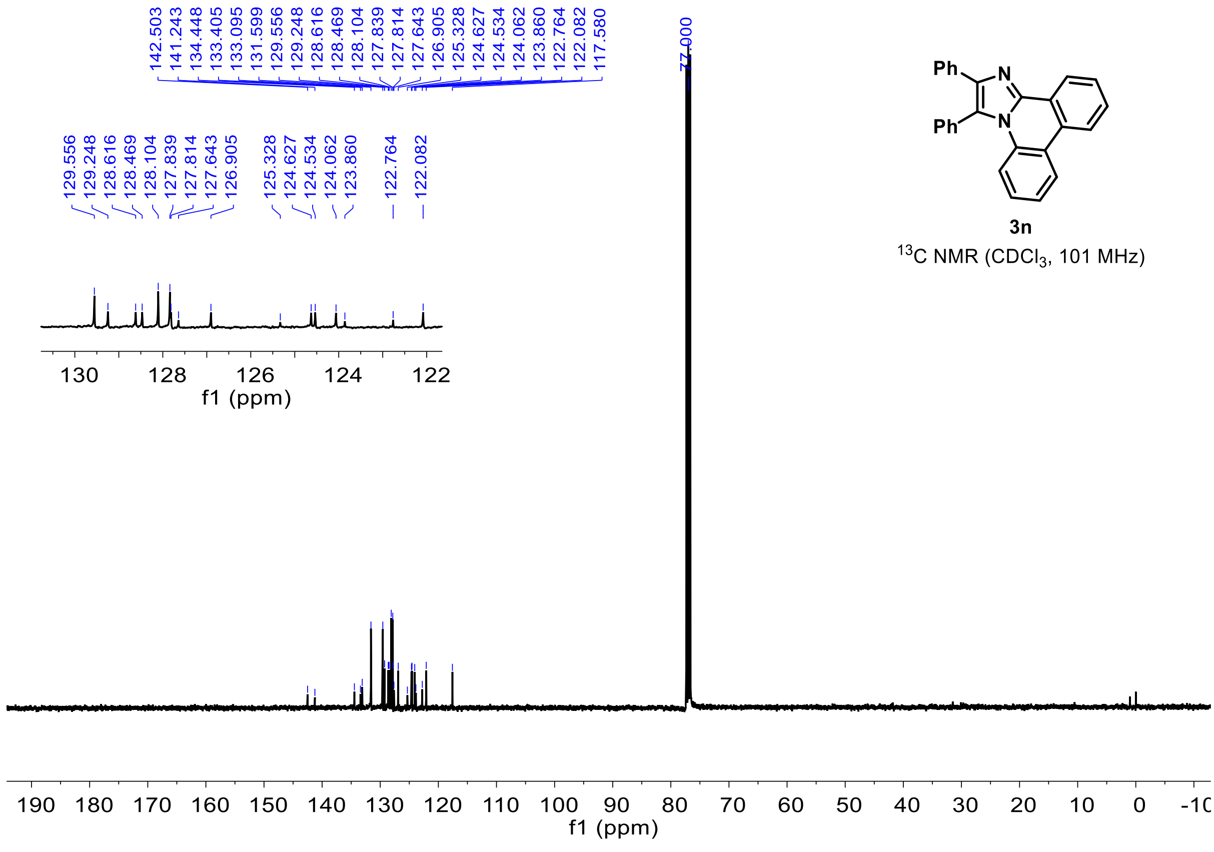
142.503
141.243
134.448
133.405
133.095
131.599
129.556
129.248
128.616
128.469
128.104
127.839
127.814
127.643
126.905
125.328
124.627
124.534
124.062
123.860
122.764
122.082
117.580
77.000
Ph
Ph
N
N
3n
13C NMR (CDCl3, 101 MHz)
130 128 126 124 122
f1 (ppm)
190 180 170 160 150 140 130 120 110 100 90 80 70 60 50 40 30 20 10 0 -10
f1 (ppm)

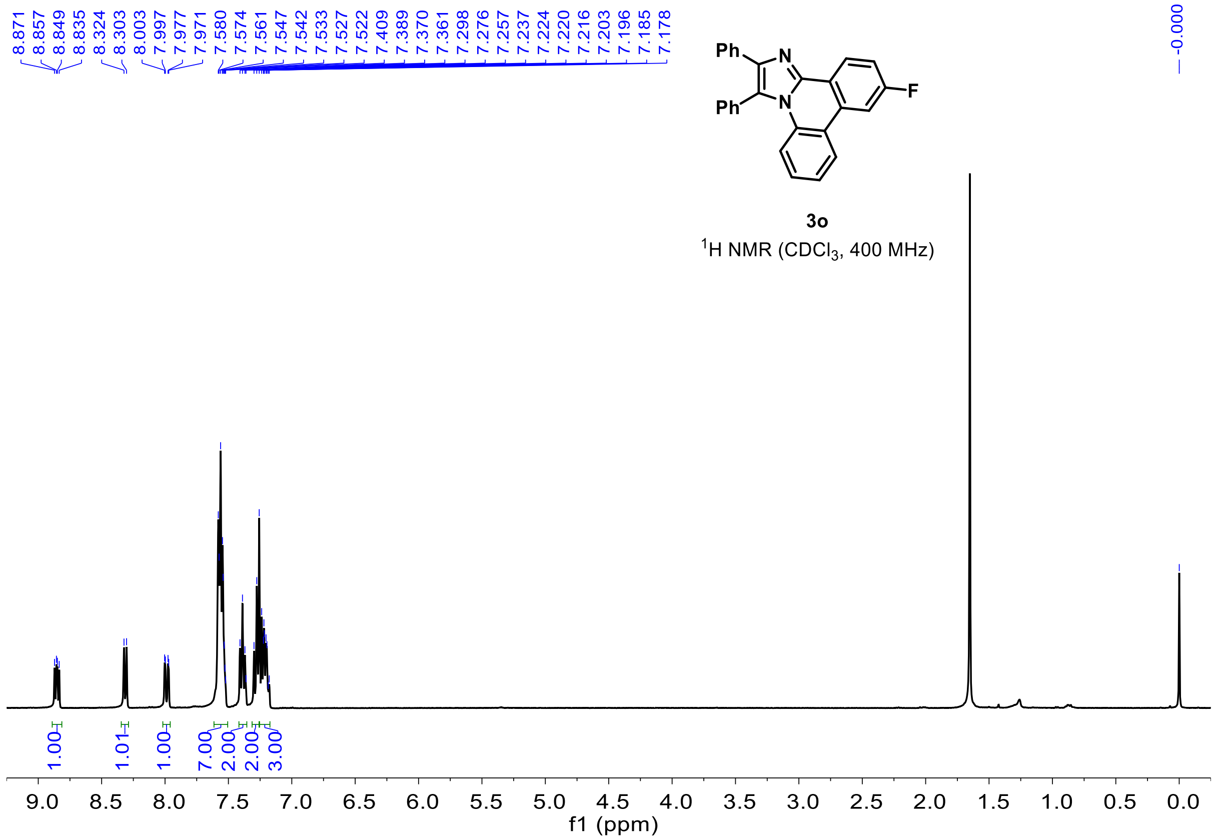
8.871
8.857
8.849
8.835
8.324
8.303
8.003
7.997
7.977
7.971
7.580
7.574
7.561
7.547
7.542
7.533
7.527
7.522
7.409
7.389
7.370
7.361
7.298
7.276
7.257
7.237
7.224
7.220
7.216
7.203
7.196
7.185
7.178
-0.000
Ph
N
F
Ph
N
3o
^{1}H NMR (CDCl$_3$, 400 MHz)
1.00
1.01
1.00
7.00
2.00
2.00
3.00
9.0 8.5 8.0 7.5 7.0 6.5 6.0 5.5 5.0 4.5 4.0 3.5 3.0 2.5 2.0 1.5 1.0 0.5 0.0
f1 (ppm)

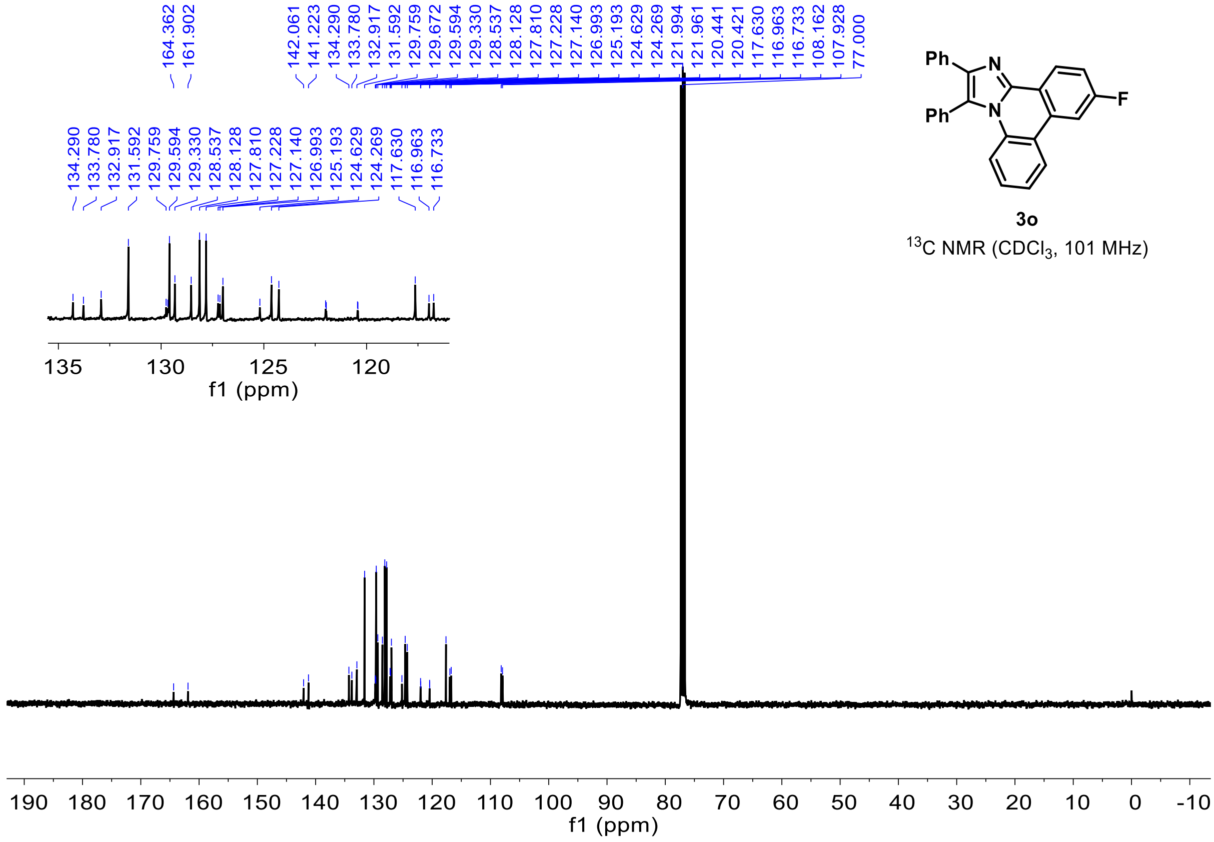
164.362
161.902
142.061
141.223
134.290
133.780
132.917
131.592
129.759
129.672
129.594
129.330
128.537
128.128
127.810
127.228
127.140
126.993
125.193
124.629
124.269
121.994
121.961
120.441
120.421
117.630
116.963
116.733
108.162
107.928
77.000
Ph
N
F
Ph
N
3o
^{13}C NMR (CDCl$_3$, 101 MHz)
135 130 125 120
f1 (ppm)
190 180 170 160 150 140 130 120 110 100 90 80 70 60 50 40 30 20 10 0 -10
f1 (ppm)

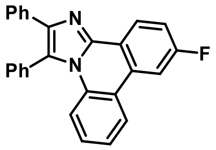

3o

^{19}F NMR ($CDCl_3$, 376 MHz)

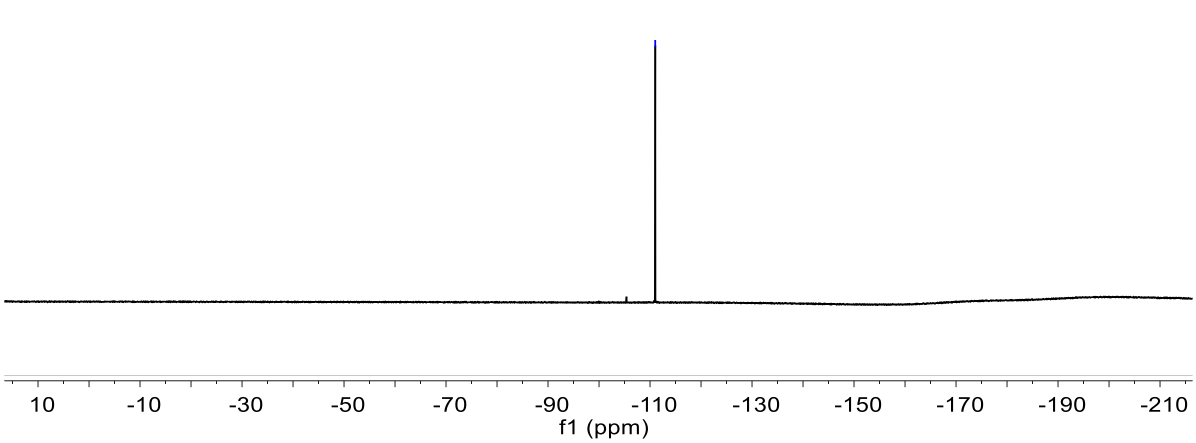

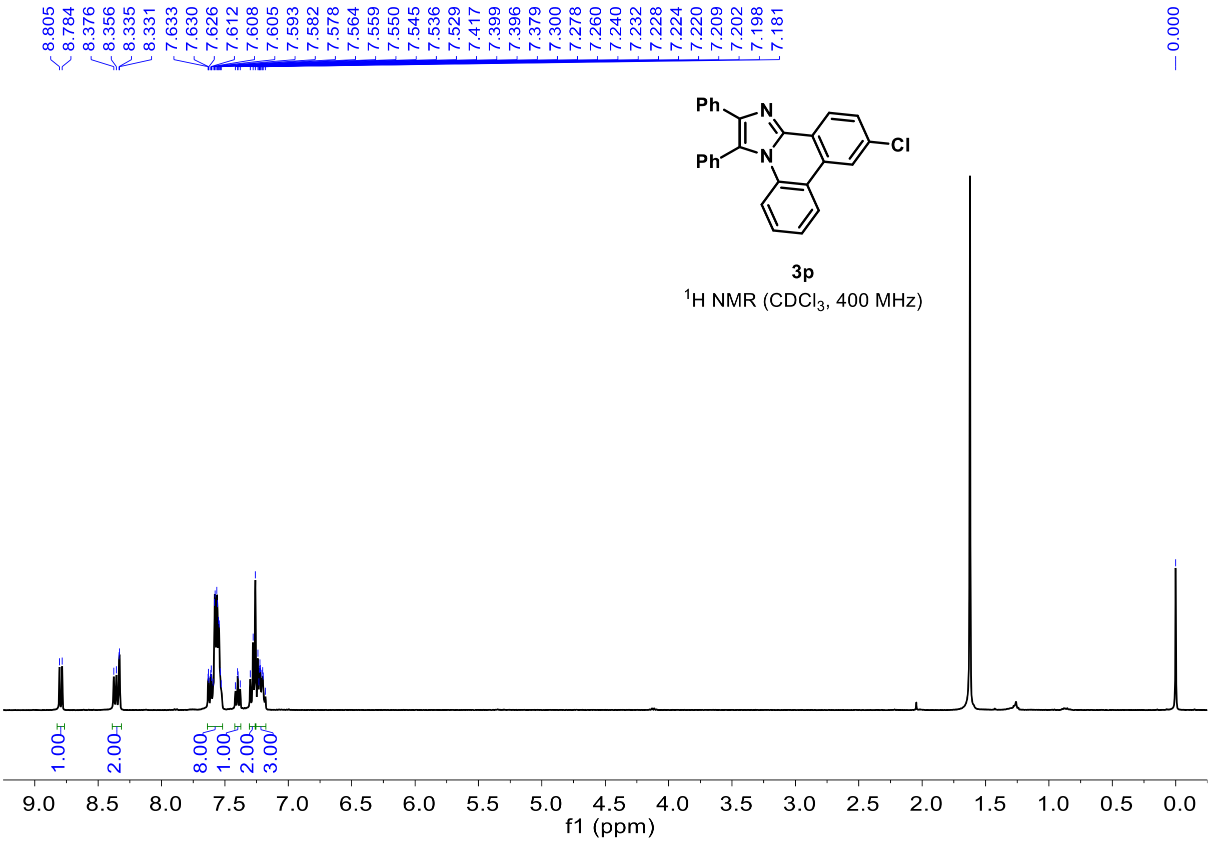
8.805
8.784
8.376
8.356
8.335
8.331
7.633
7.630
7.626
7.612
7.608
7.605
7.593
7.582
7.578
7.564
7.559
7.550
7.545
7.536
7.529
7.417
7.399
7.396
7.379
7.300
7.278
7.260
7.240
7.232
7.228
7.224
7.220
7.209
7.202
7.198
7.181
0.000
Ph
N
Cl
Ph
N
3p
1H NMR (CDCl3, 400 MHz)
1.00
2.00
8.00
1.00
2.00
3.00
9.0 8.5 8.0 7.5 7.0 6.5 6.0 5.5 5.0 4.5 4.0 3.5 3.0 2.5 2.0 1.5 1.0 0.5 0.0
f1 (ppm)

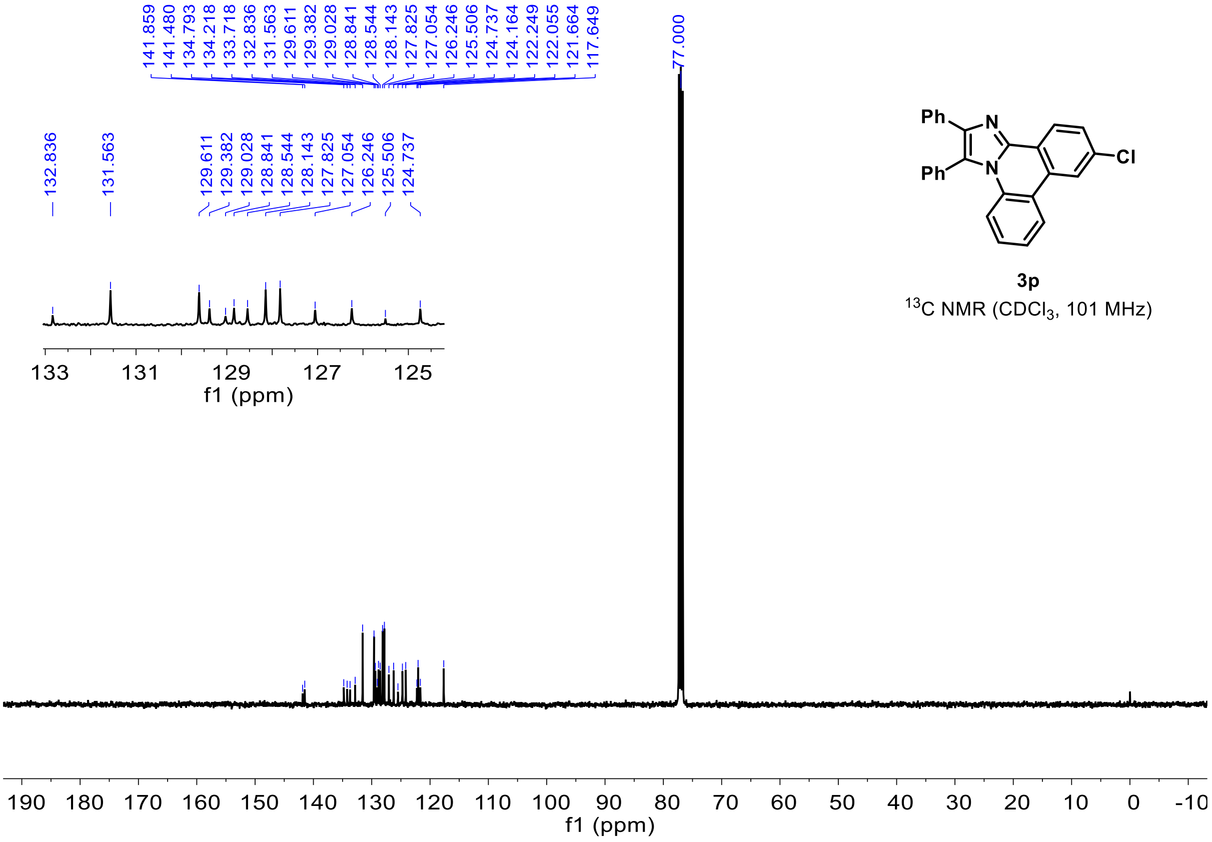
141.859
141.480
134.793
134.218
133.718
132.836
131.563
129.611
129.382
129.028
128.841
128.544
128.143
127.825
127.054
126.246
125.506
124.737
124.164
122.249
122.055
121.664
117.649
77.000
132.836
131.563
129.611
129.382
129.028
128.841
128.544
128.143
127.825
127.054
126.246
125.506
124.737
133 131 129 127 125
f1 (ppm)
Ph
N
Cl
Ph
N
3p
13C NMR (CDCl3, 101 MHz)
190 180 170 160 150 140 130 120 110 100 90 80 70 60 50 40 30 20 10 0 -10
f1 (ppm)

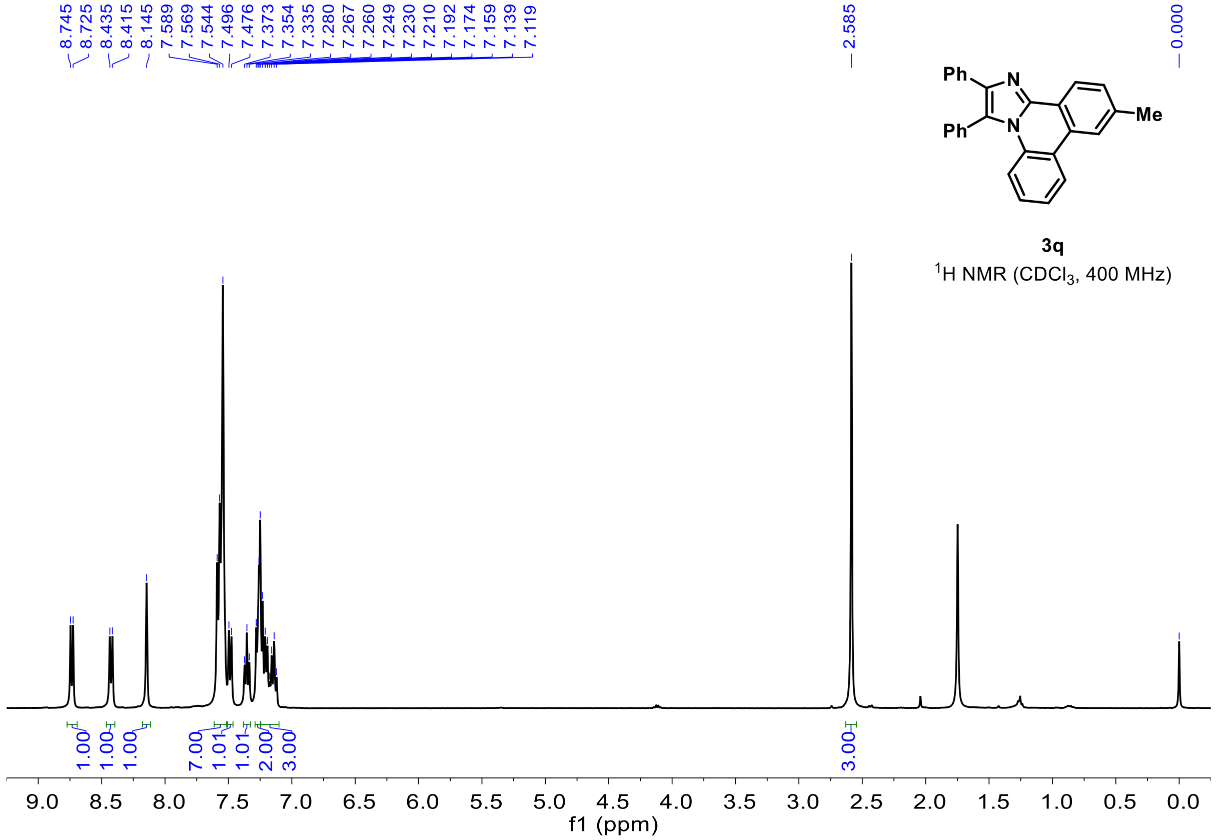
8.745
8.725
8.435
8.415
8.145
7.589
7.569
7.544
7.496
7.476
7.373
7.354
7.335
7.280
7.267
7.260
7.249
7.230
7.210
7.192
7.174
7.159
7.139
7.119
2.585
0.000
Ph
Ph
N
N
Me
3q
¹H NMR (CDCl₃, 400 MHz)
1.00
1.00
1.00
7.00
1.01
1.01
2.00
3.00
3.00
9.0 8.5 8.0 7.5 7.0 6.5 6.0 5.5 5.0 4.5 4.0 3.5 3.0 2.5 2.0 1.5 1.0 0.5 0.0
f1 (ppm)

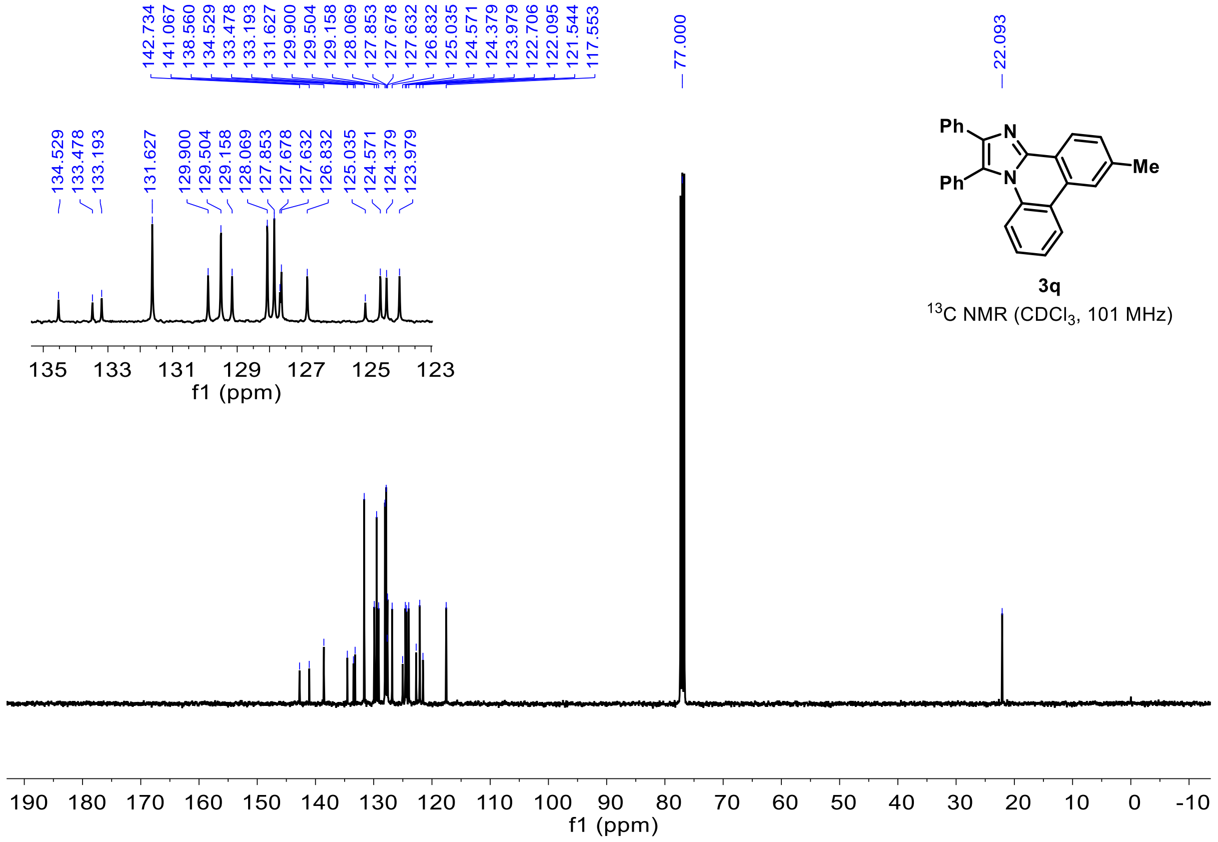
142.734
141.067
138.560
134.529
133.478
133.193
131.627
129.900
129.504
129.158
128.069
127.853
127.678
127.632
126.832
125.035
124.571
124.379
123.979
122.706
122.095
121.544
117.553
77.000
22.093
134.529
133.478
133.193
131.627
129.900
129.504
129.158
128.069
127.853
127.678
127.632
126.832
125.035
124.571
124.379
123.979
135 133 131 129 127 125 123
f1 (ppm)
Ph
Ph
N
N
Me
3q
¹³C NMR (CDCl₃, 101 MHz)
190 180 170 160 150 140 130 120 110 100 90 80 70 60 50 40 30 20 10 0 -10
f1 (ppm)

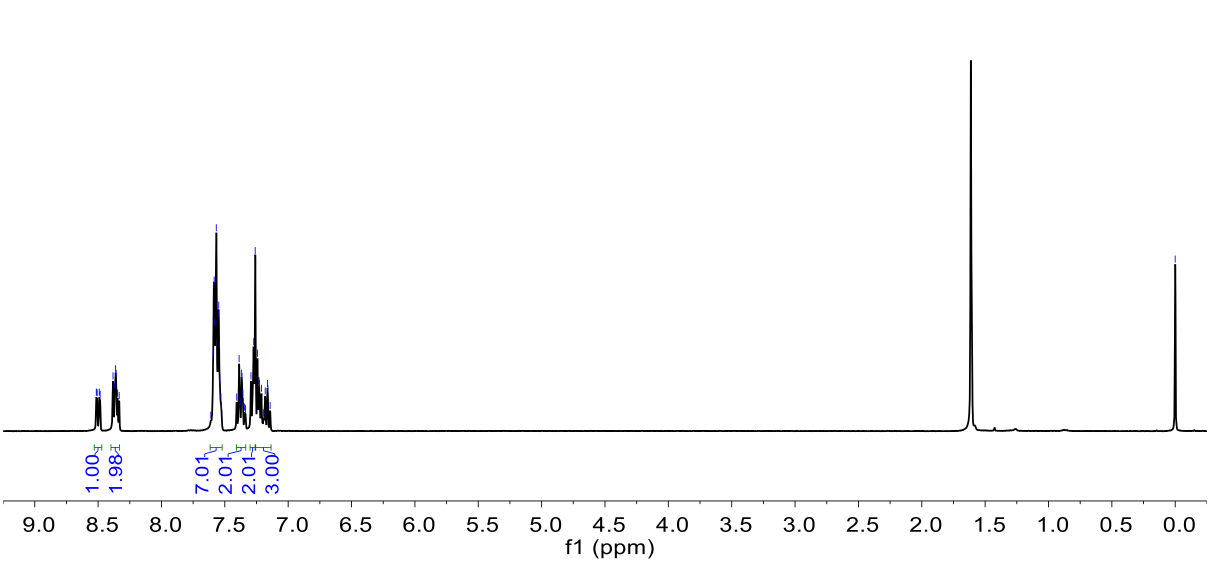

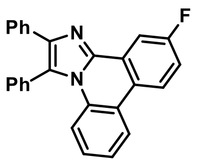

3r

^{1}H NMR ($CDCl_3$, 400 MHz)

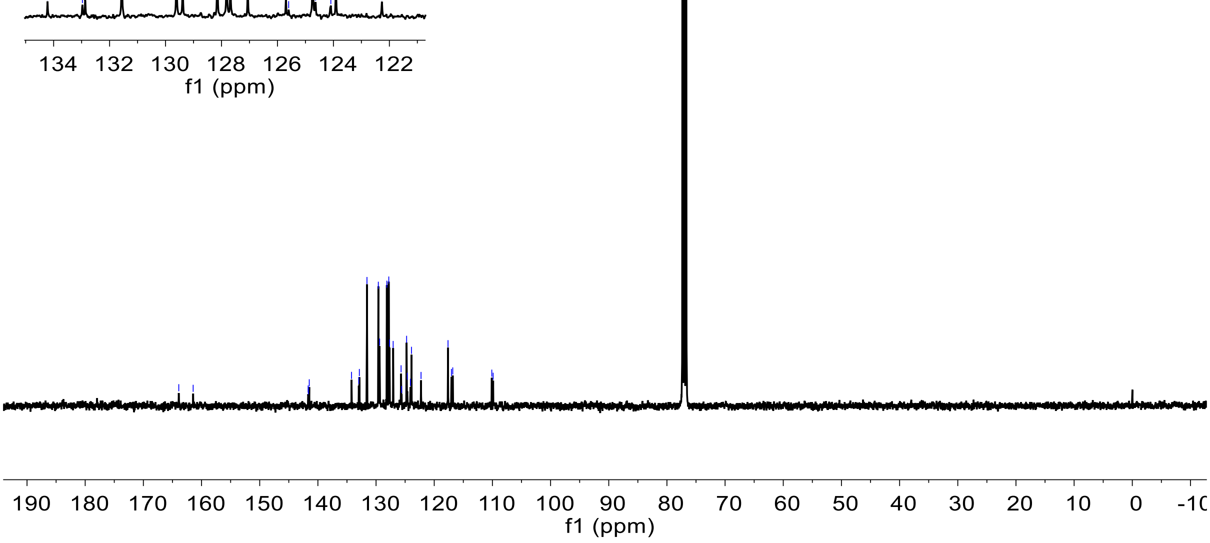

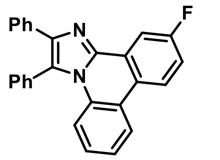

3r

^{13}C NMR ($CDCl_3$, 101 MHz)

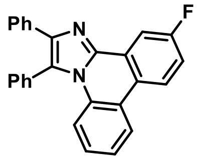

3r

^{19}F NMR ($CDCl_3$, 376 MHz)

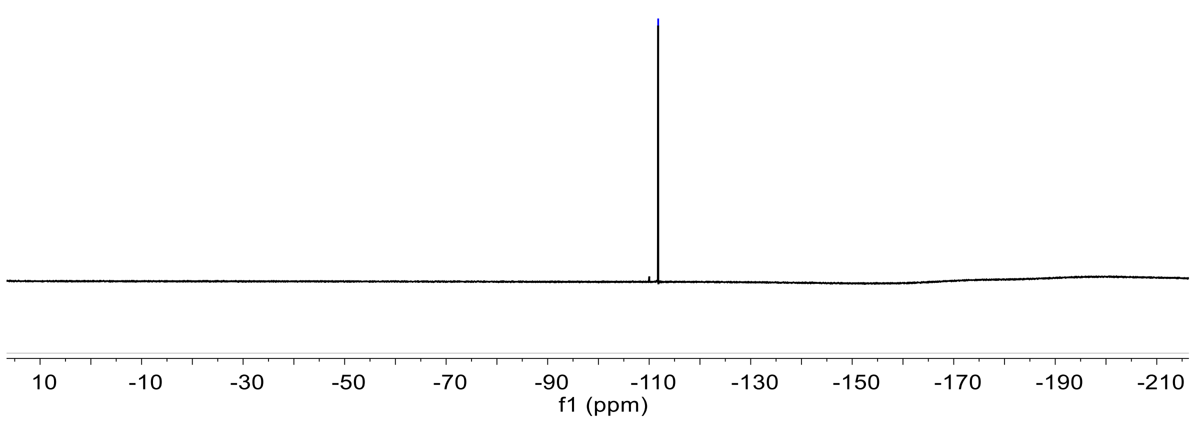

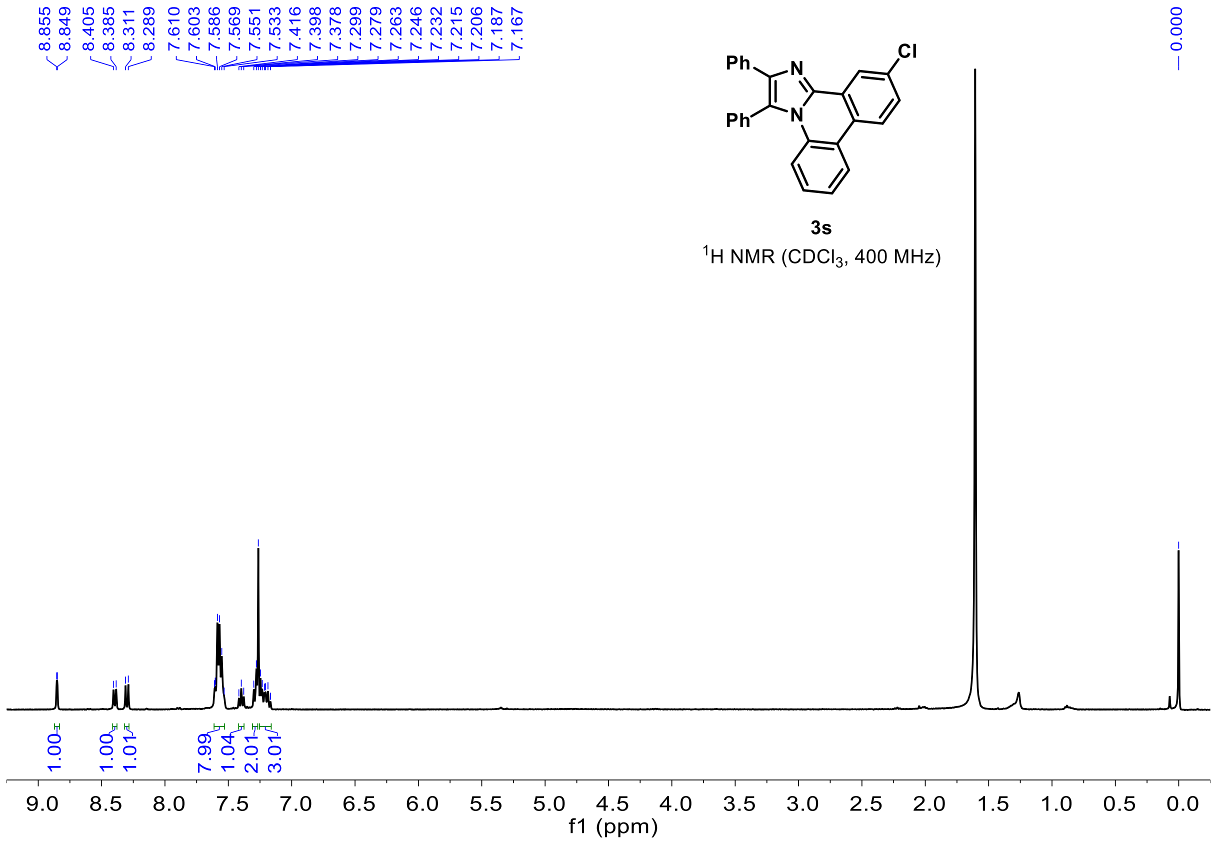

3s

^{1}H NMR ($CDCl_3$, 400 MHz)

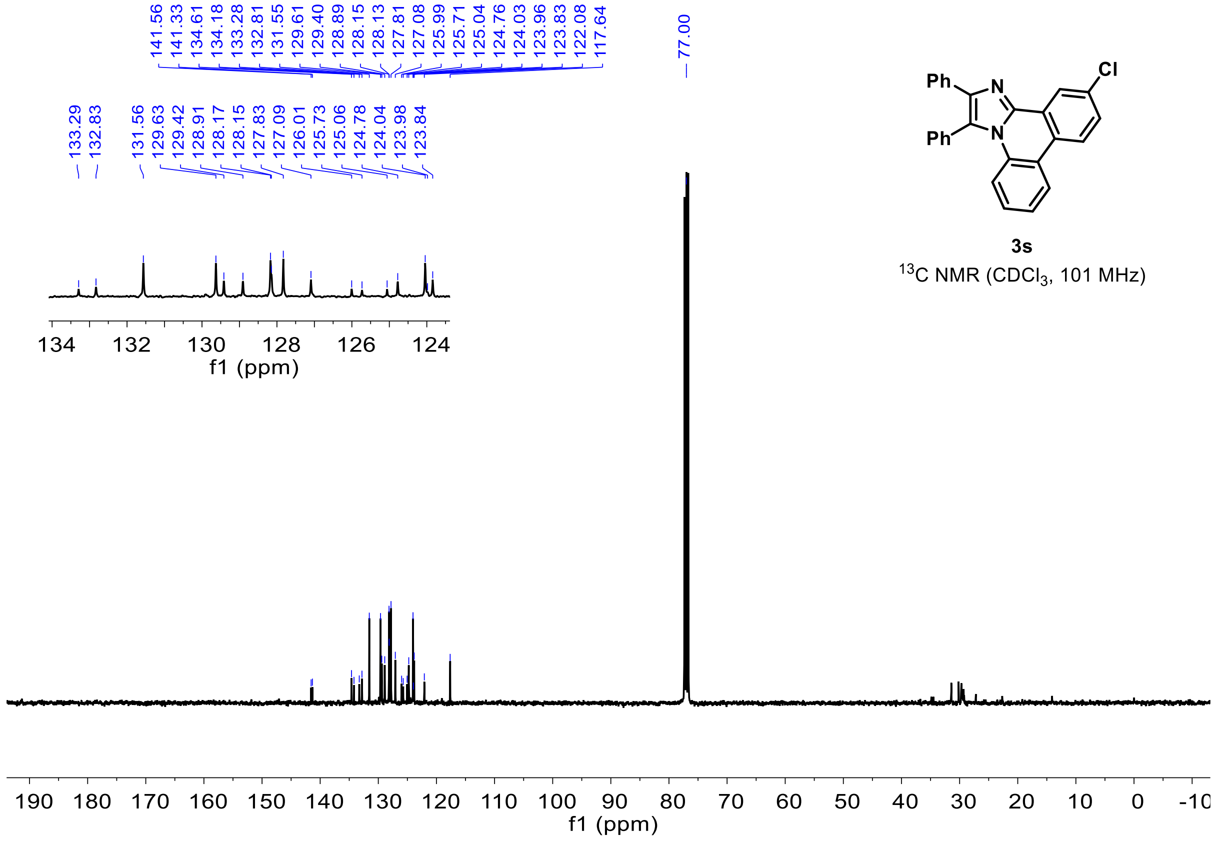

3s

^{13}C NMR ($CDCl_3$, 101 MHz)

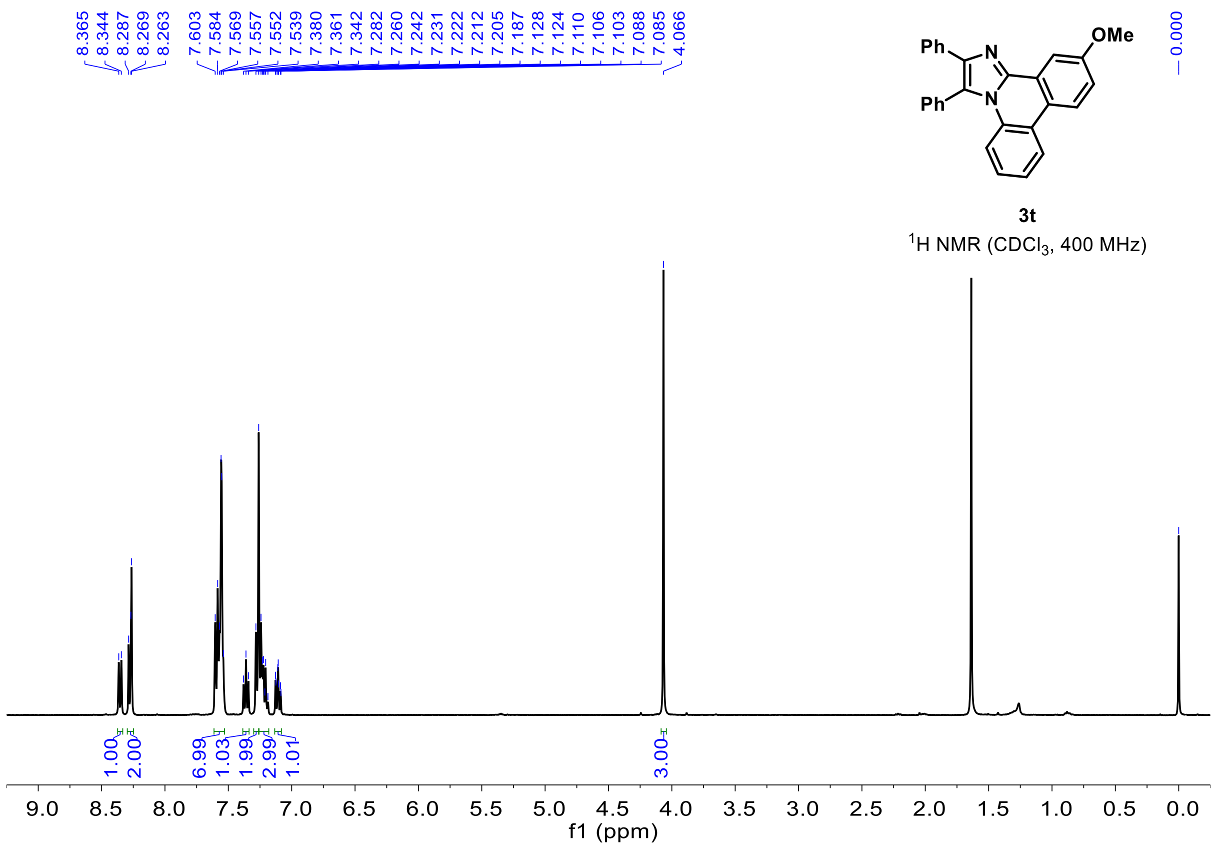
3t
^{1}H NMR (CDCl$_3$, 400 MHz)
f1 (ppm)

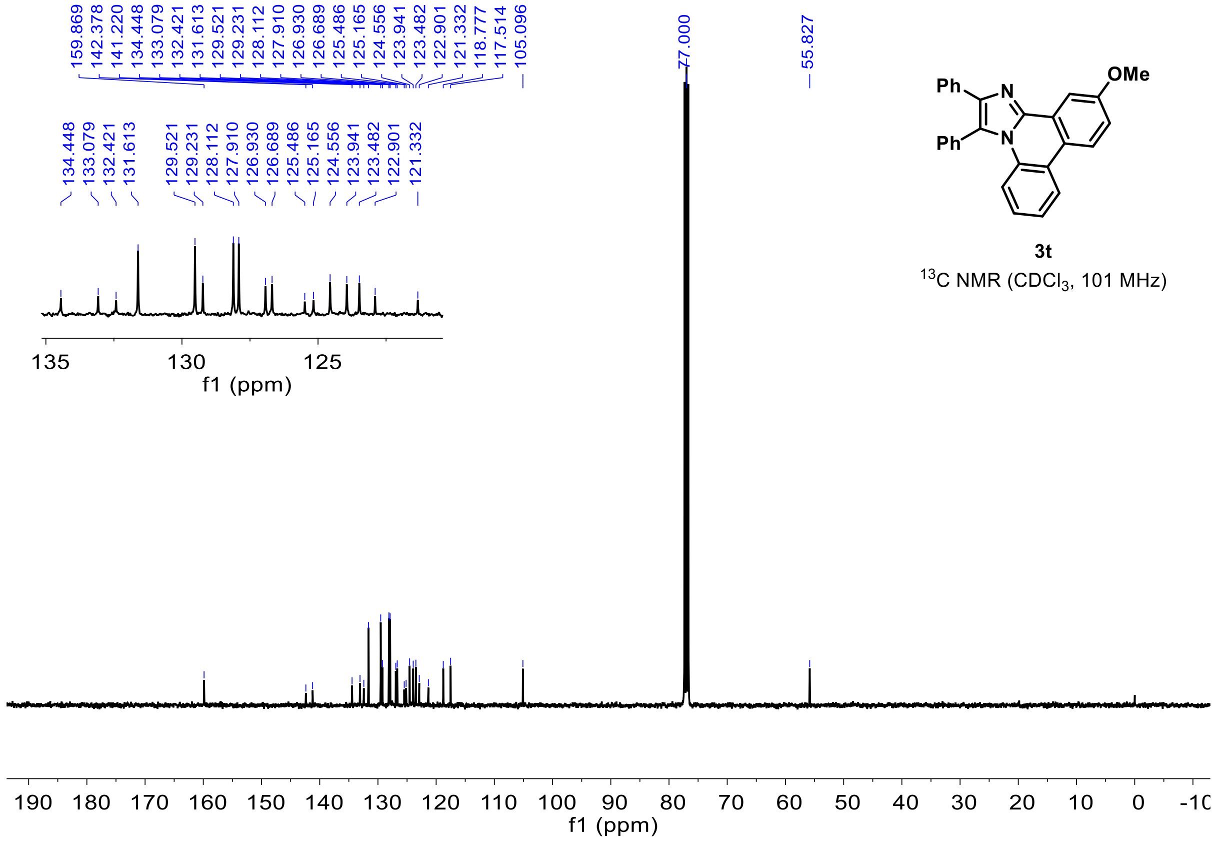
3t
^{13}C NMR (CDCl$_3$, 101 MHz)
f1 (ppm)

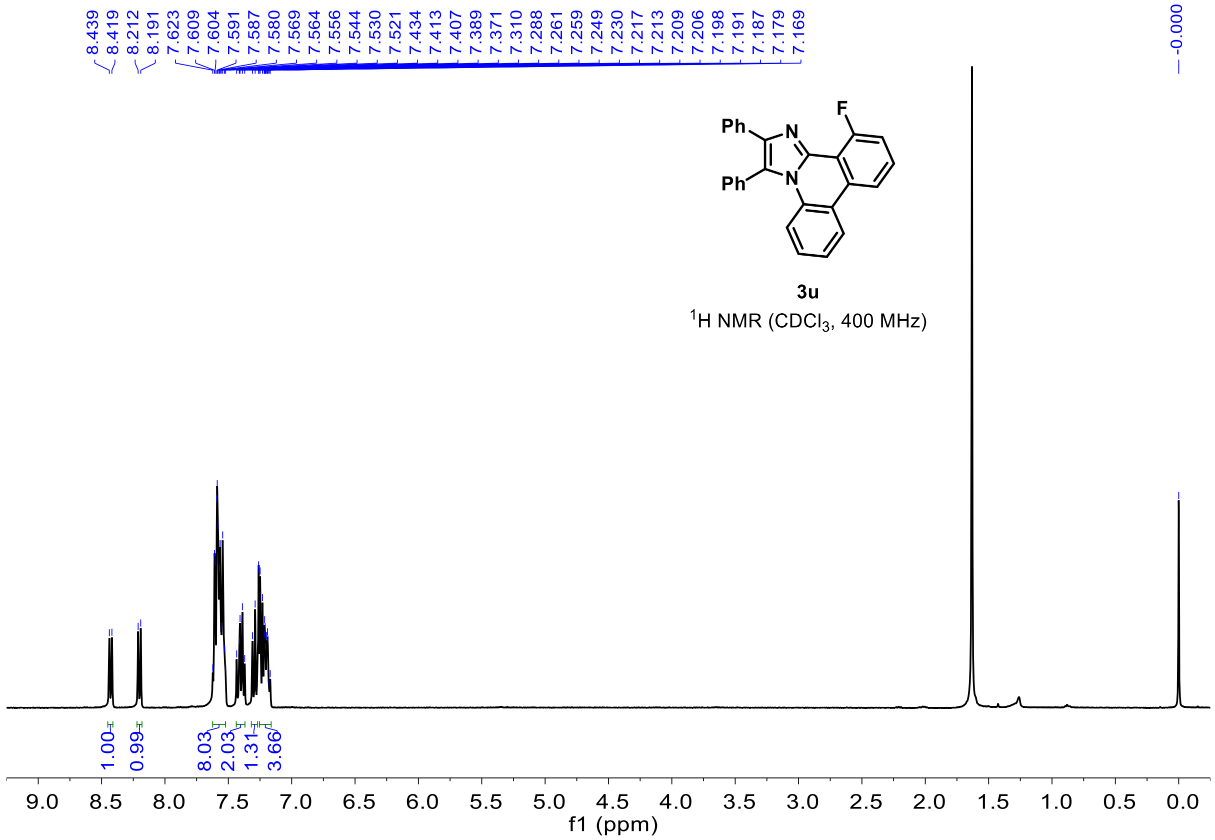
8.439
8.419
8.212
8.191
7.623
7.609
7.604
7.591
7.587
7.580
7.569
7.564
7.556
7.544
7.530
7.521
7.434
7.413
7.407
7.389
7.371
7.310
7.288
7.261
7.259
7.249
7.230
7.217
7.213
7.209
7.206
7.198
7.191
7.187
7.179
7.169
-0.000
F
Ph
N
Ph
N
3u
1H NMR (CDCl3, 400 MHz)
1.00
0.99
8.03
2.03
1.31
3.66
9.0 8.5 8.0 7.5 7.0 6.5 6.0 5.5 5.0 4.5 4.0 3.5 3.0 2.5 2.0 1.5 1.0 0.5 0.0
f1 (ppm)

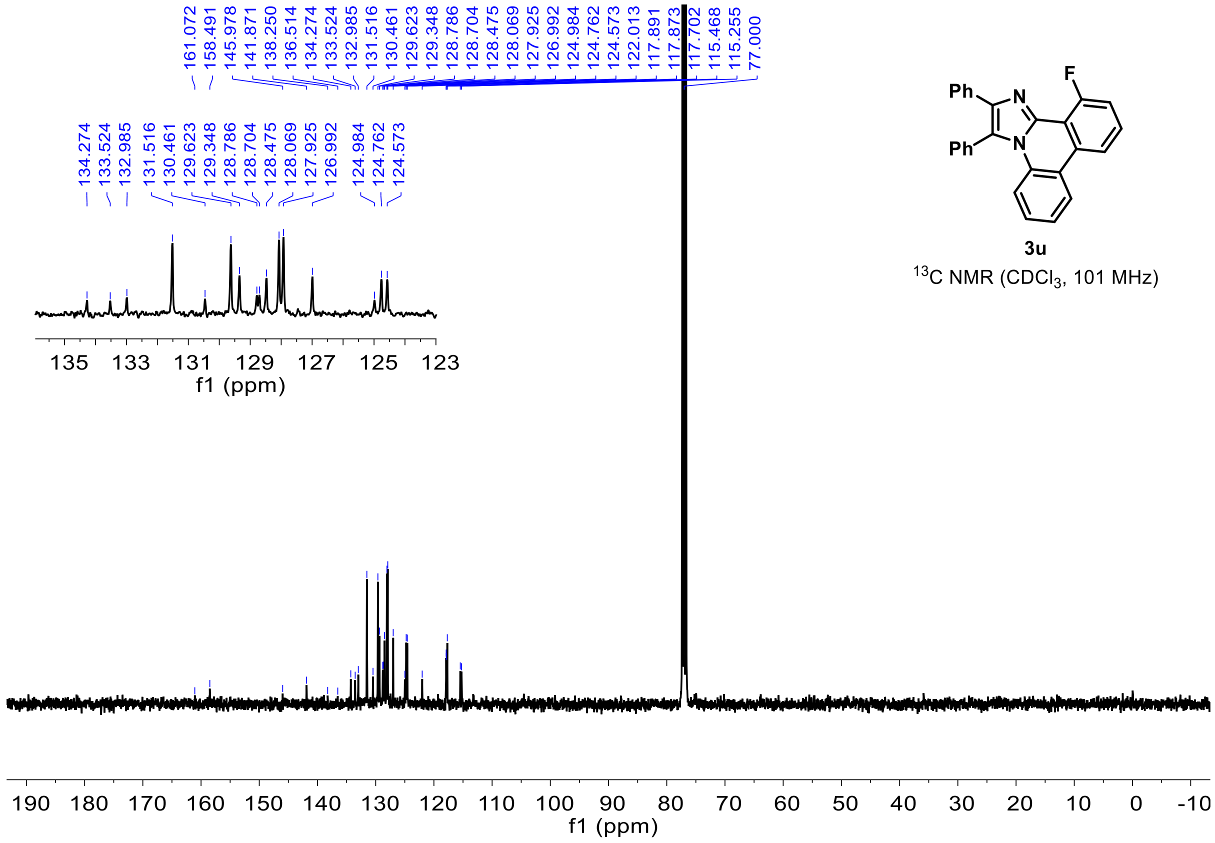
161.072
158.491
145.978
141.871
138.250
136.514
134.274
133.524
132.985
131.516
130.461
129.623
129.348
128.786
128.704
128.475
128.069
127.925
126.992
124.984
124.762
124.573
122.013
117.891
117.873
117.702
115.468
115.255
77.000
134.274
133.524
132.985
131.516
130.461
129.623
129.348
128.786
128.704
128.475
128.069
127.925
126.992
124.984
124.762
124.573
135 133 131 129 127 125 123
f1 (ppm)
F
Ph
N
Ph
N
3u
13C NMR (CDCl3, 101 MHz)
190 180 170 160 150 140 130 120 110 100 90 80 70 60 50 40 30 20 10 0 -10
f1 (ppm)

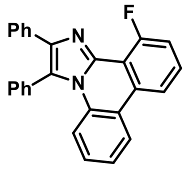

3u

^{19}F NMR (CDCl$_3$, 376 MHz)

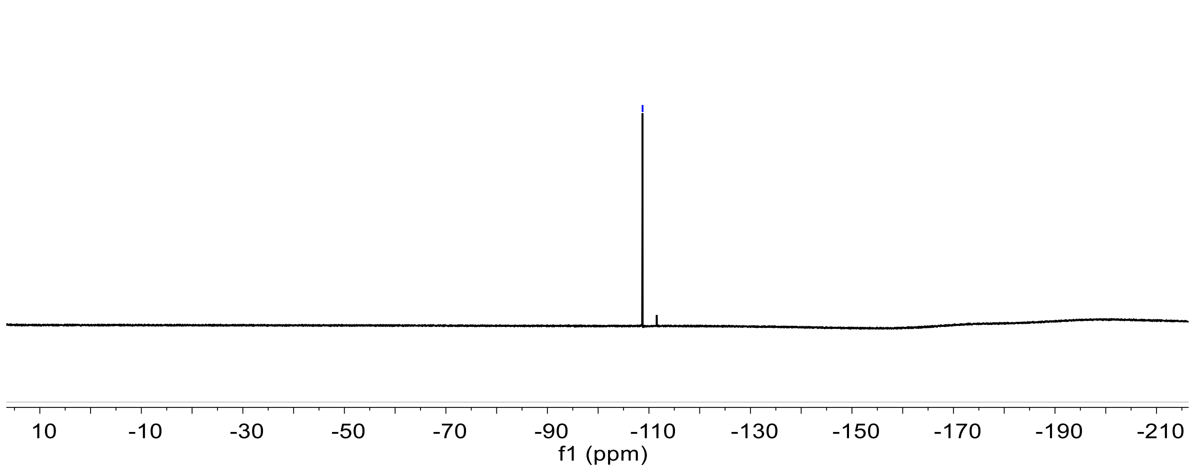

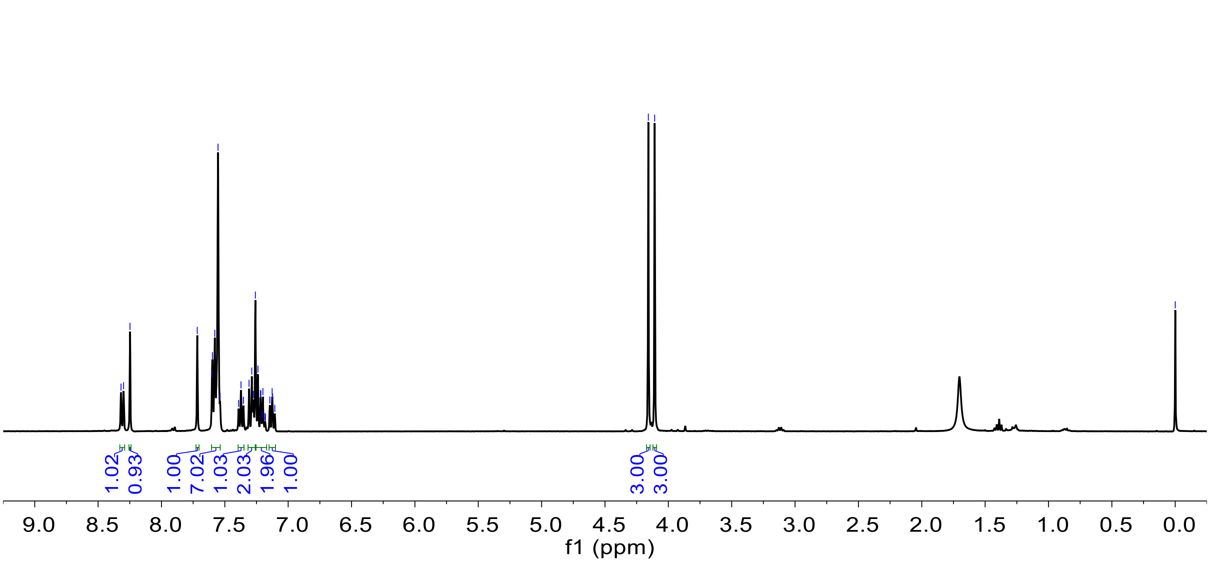

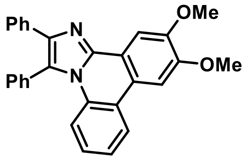

3v

^{1}H NMR (CDCl$_3$, 400 MHz)

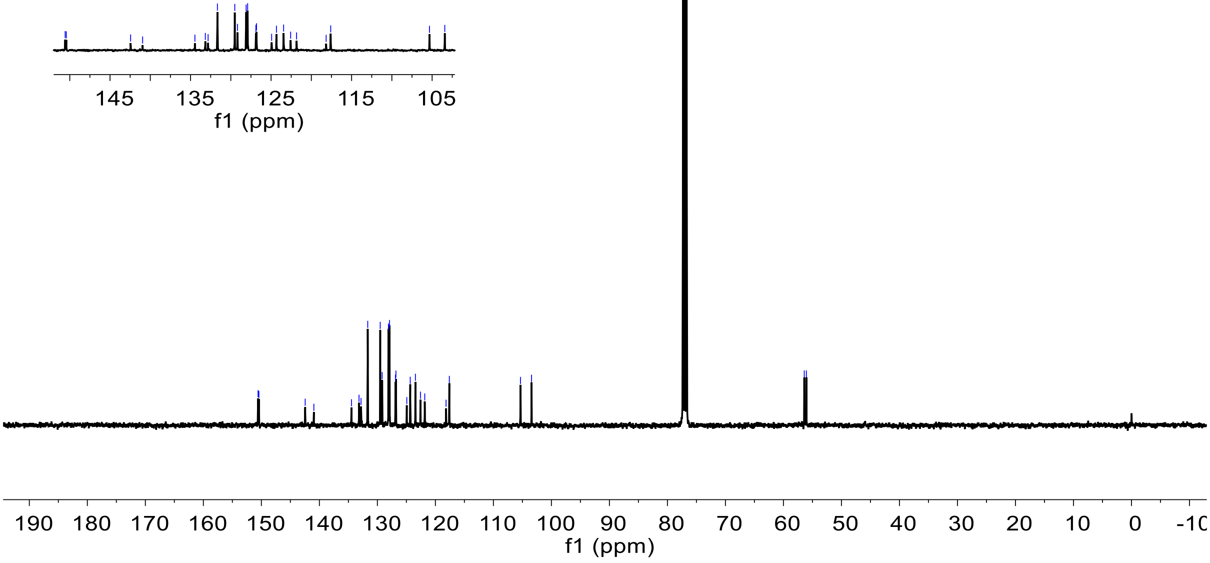

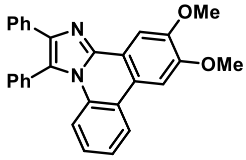

3v

^{13}C NMR (CDCl$_3$, 101 MHz)

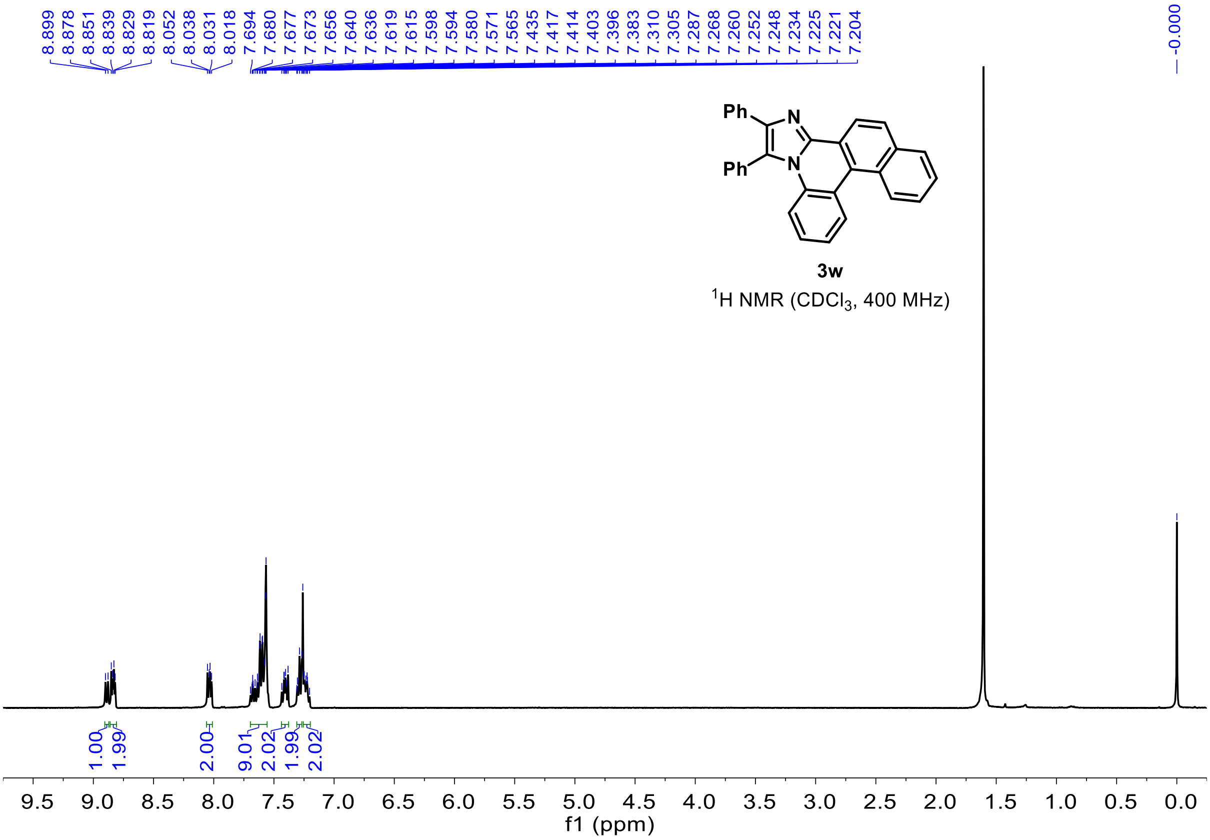

3w

^{1}H NMR ($CDCl_3$, 400 MHz)

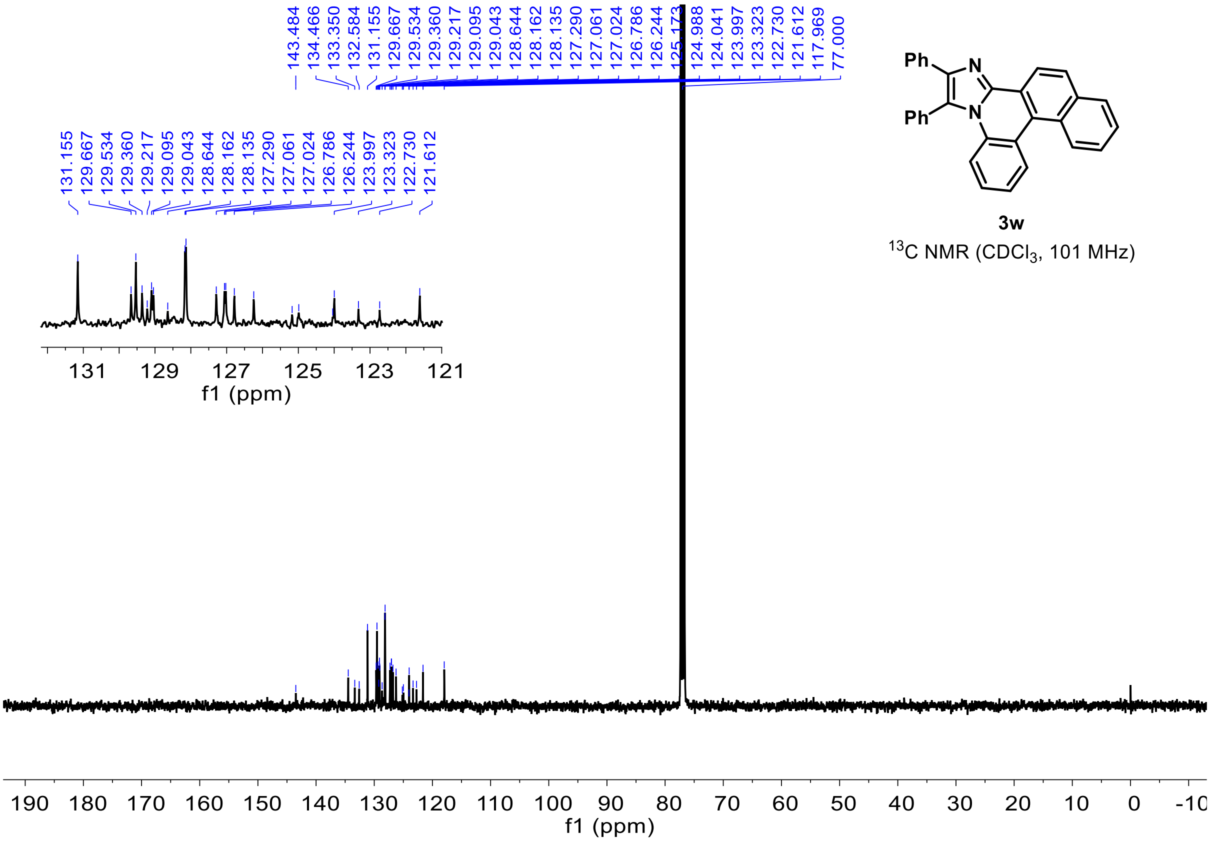

3w

^{13}C NMR ($CDCl_3$, 101 MHz)

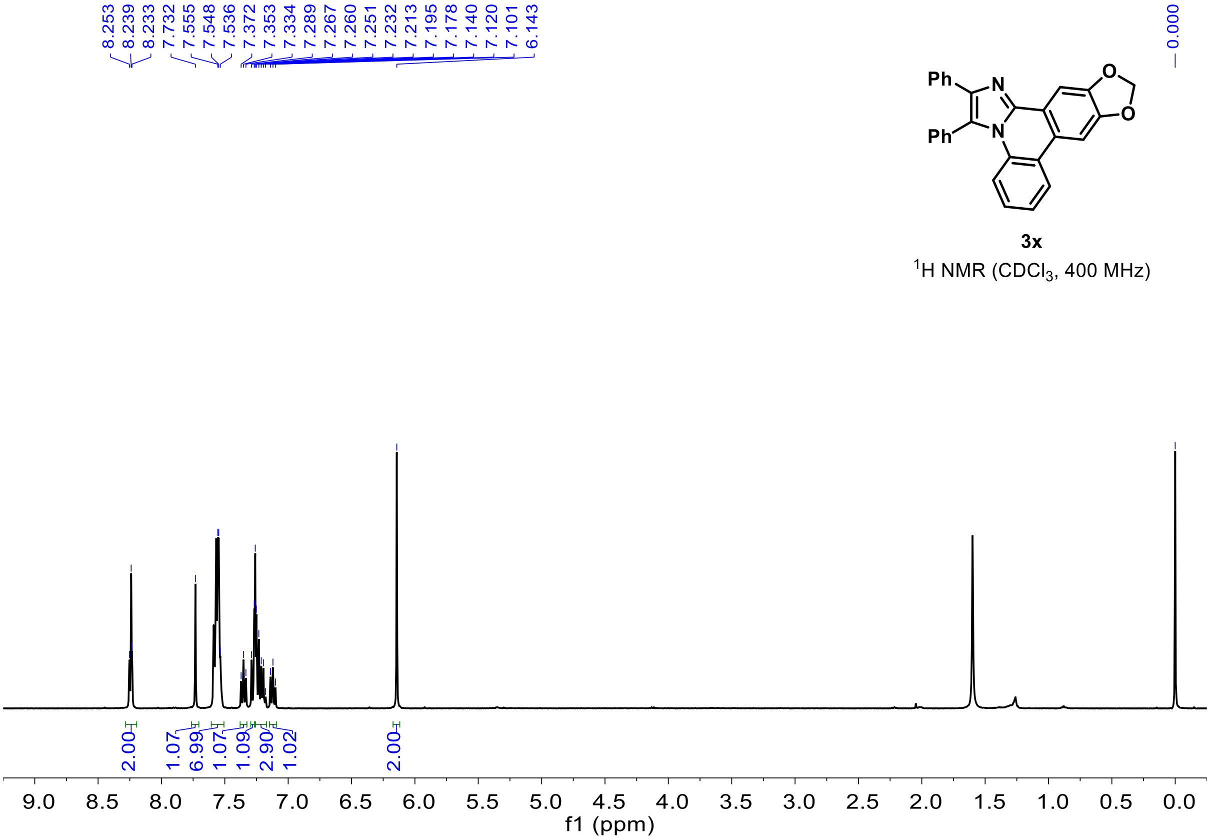
8.253
8.239
8.233
7.732
7.555
7.548
7.536
7.372
7.353
7.334
7.289
7.267
7.260
7.251
7.232
7.213
7.195
7.178
7.140
7.120
7.101
6.143
0.000
Ph
N
O
O
Ph
N
3x
^{1}H NMR (CDCl$_3$, 400 MHz)
2.00
1.07
6.99
1.07
1.09
2.90
1.02
2.00
9.0 8.5 8.0 7.5 7.0 6.5 6.0 5.5 5.0 4.5 4.0 3.5 3.0 2.5 2.0 1.5 1.0 0.5 0.0
f1 (ppm)

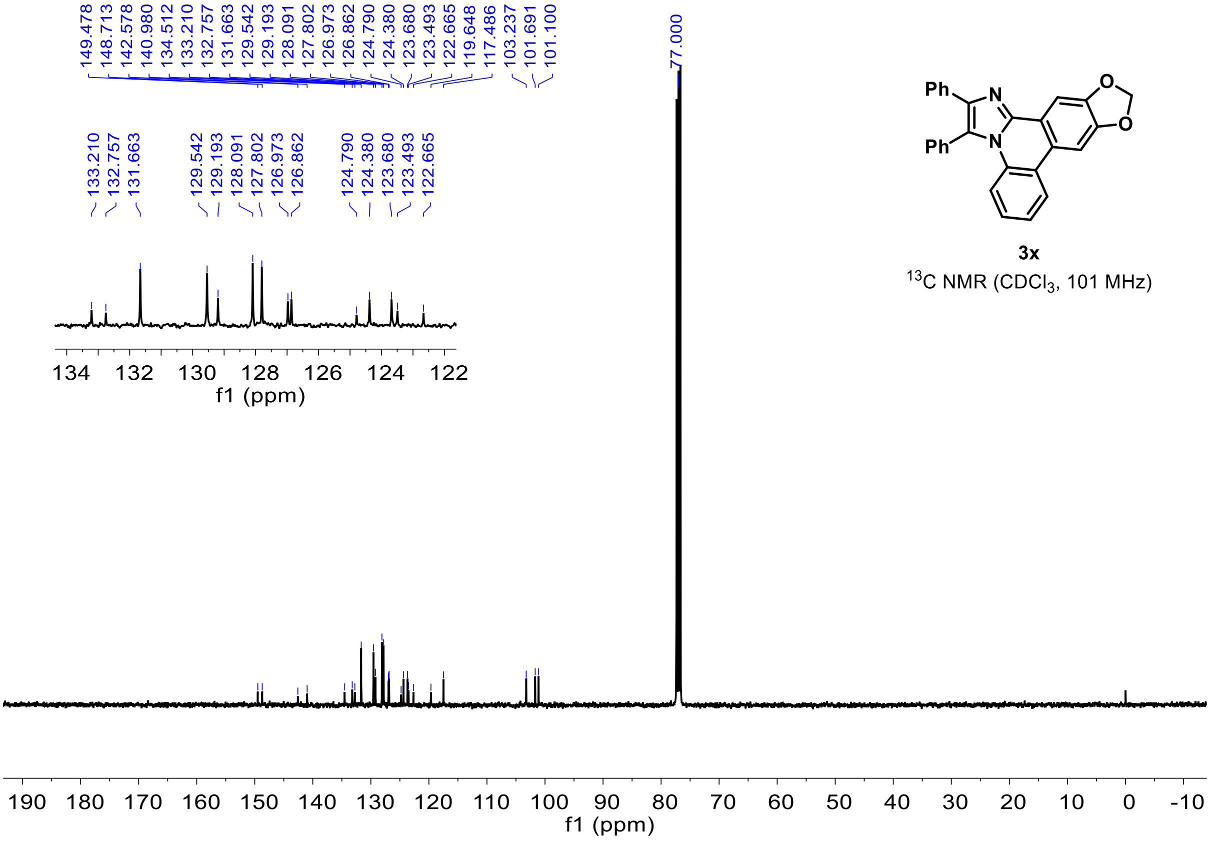
149.478
148.713
142.578
140.980
134.512
133.210
132.757
131.663
129.542
129.193
128.091
127.802
126.973
126.862
124.790
124.380
123.680
123.493
122.665
119.648
117.486
103.237
101.691
101.100
77.000
Ph
N
O
O
Ph
N
3x
^{13}C NMR (CDCl$_3$, 101 MHz)
134 132 130 128 126 124 122
f1 (ppm)
190 180 170 160 150 140 130 120 110 100 90 80 70 60 50 40 30 20 10 0 -10
f1 (ppm)

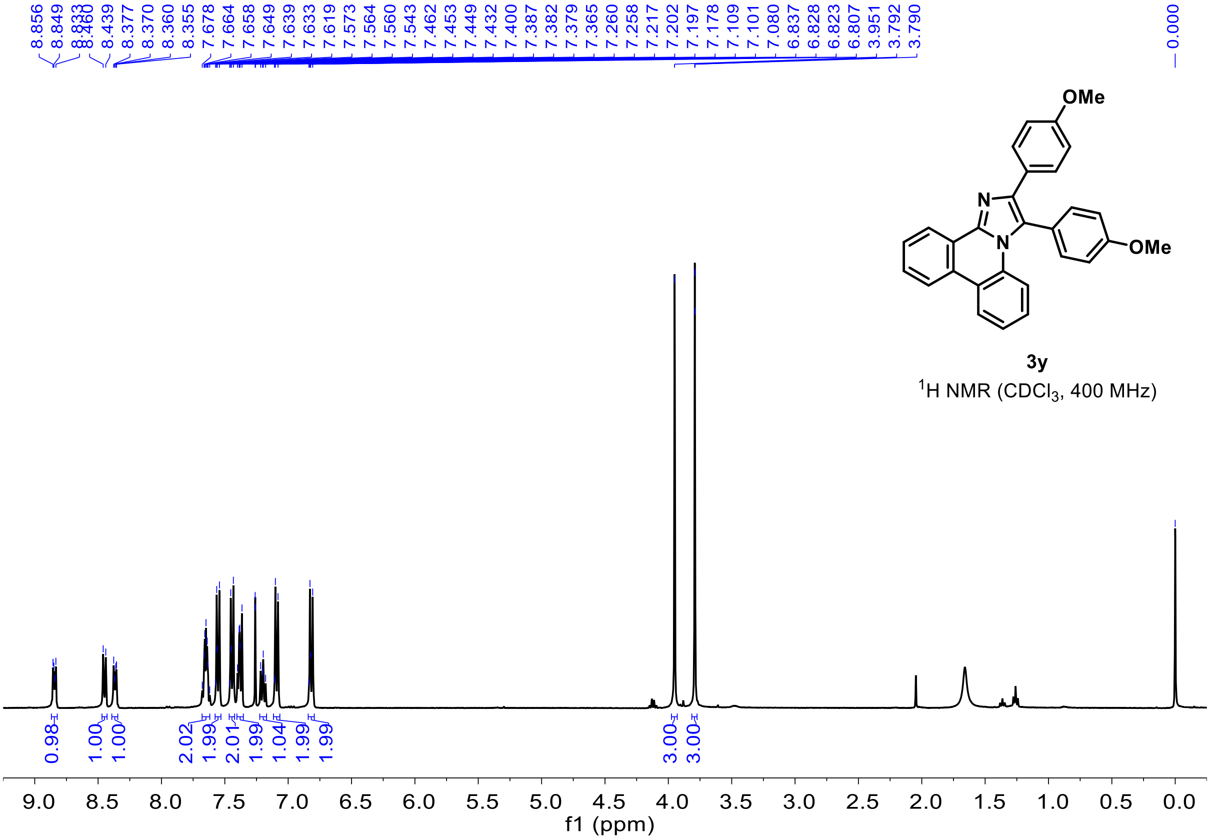

3y

^{1}H NMR (CDCl$_3$, 400 MHz)

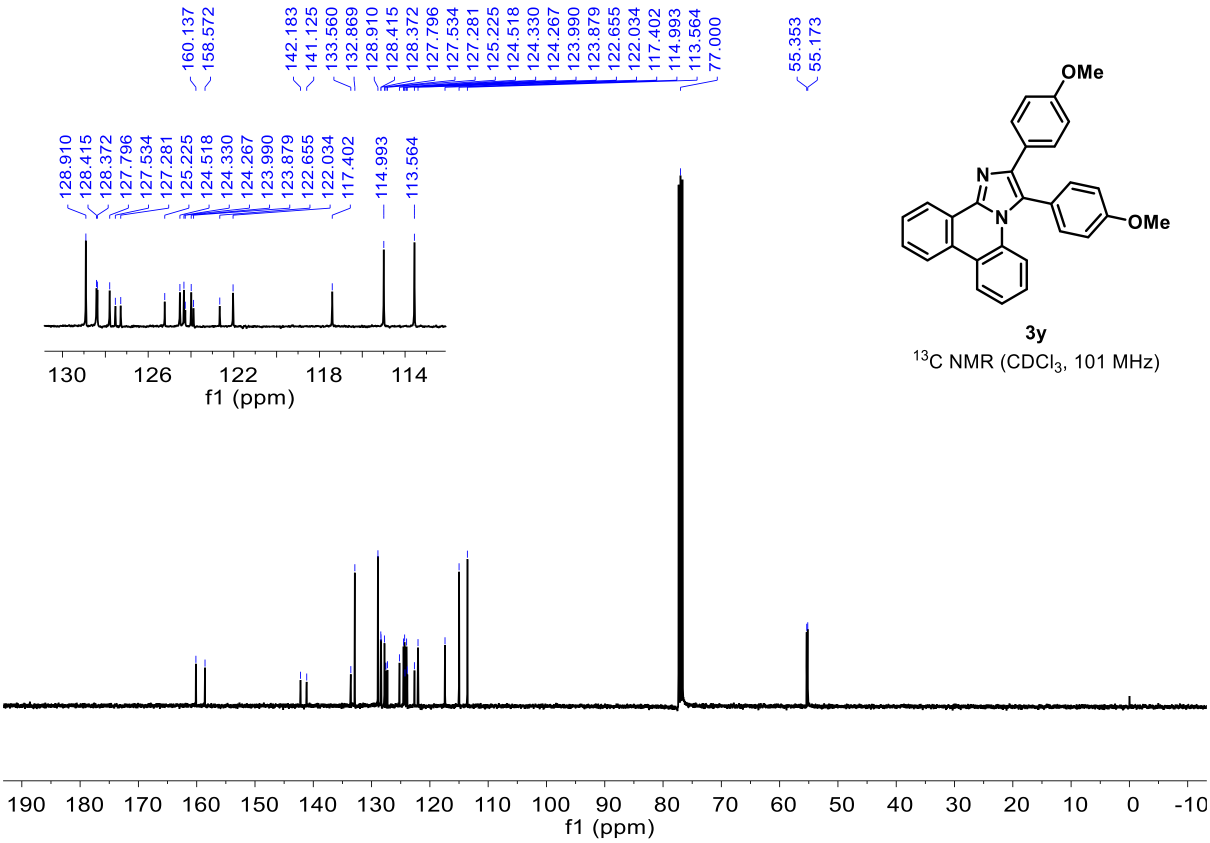

3y

^{13}C NMR (CDCl$_3$, 101 MHz)

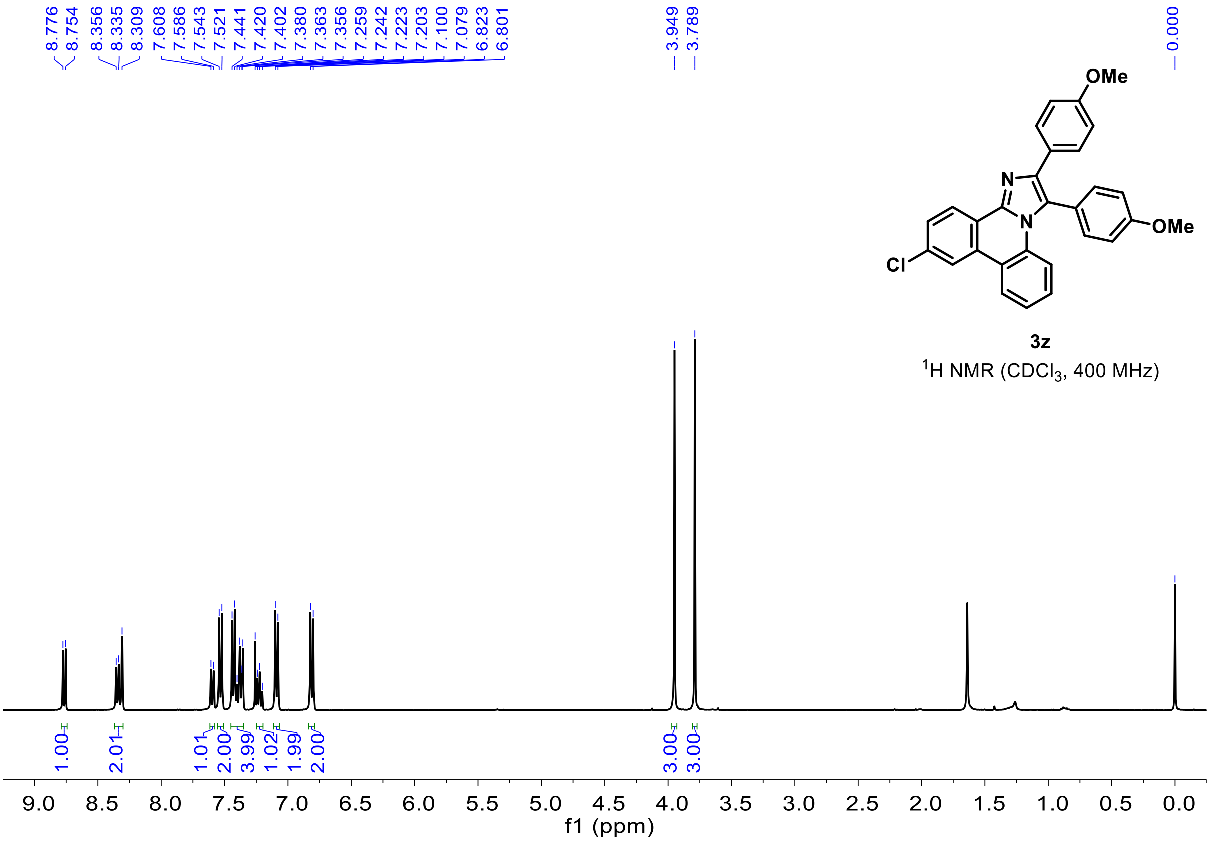

3z

^{1}H NMR ($CDCl_3$, 400 MHz)

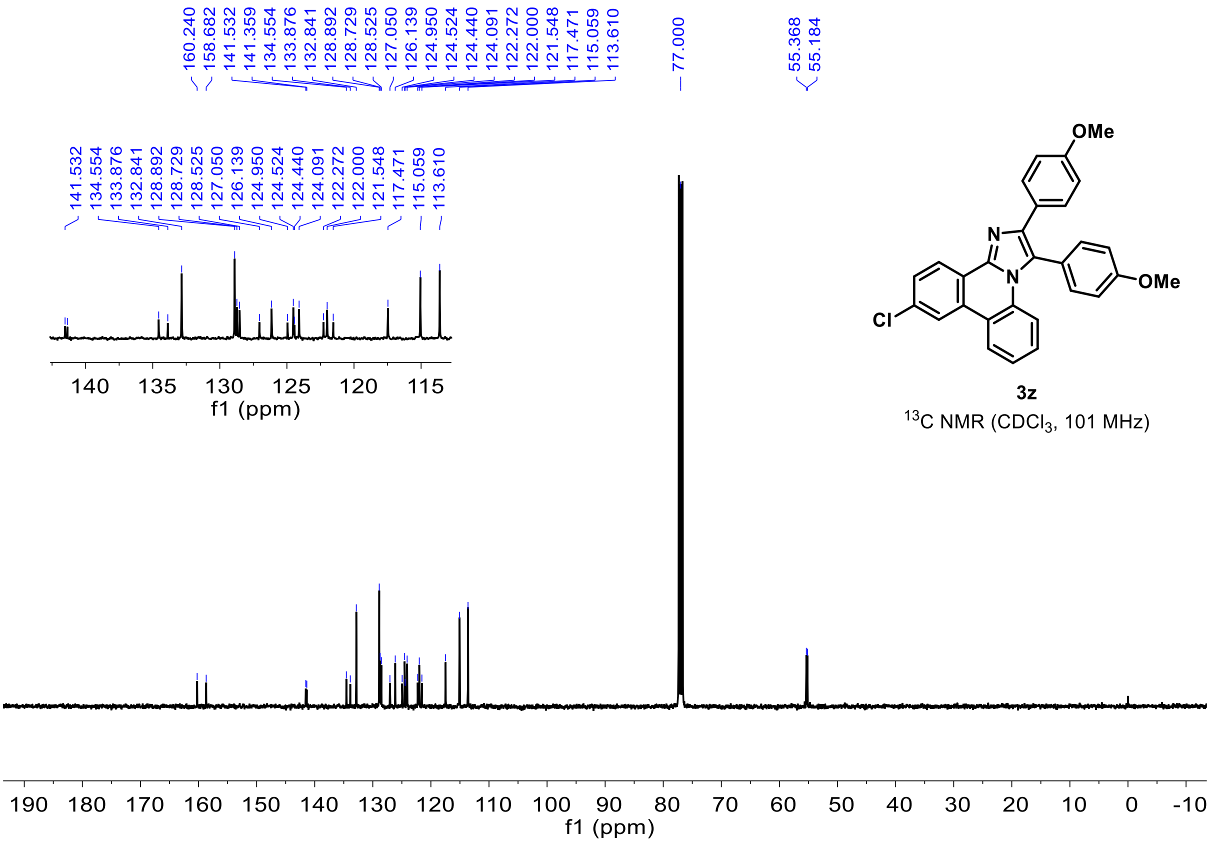

3z

^{13}C NMR ($CDCl_3$, 101 MHz)

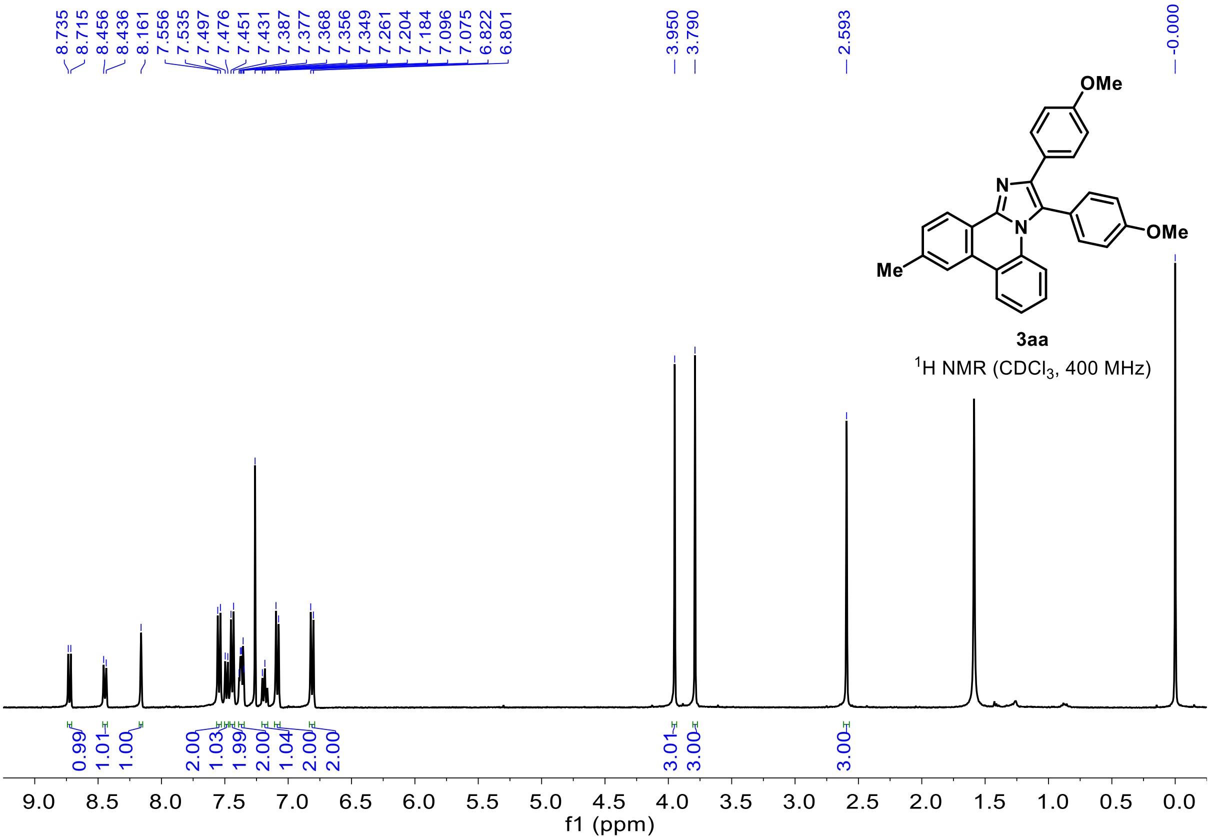
8.735
8.715
8.456
8.436
8.161
7.556
7.535
7.497
7.476
7.451
7.431
7.387
7.377
7.368
7.356
7.349
7.261
7.204
7.184
7.096
7.075
6.822
6.801
3.950
3.790
2.593
-0.000
OMe
N
N
OMe
Me
3aa
^{1}H NMR (CDCl$_3$, 400 MHz)
0.99
1.01
1.00
2.00
1.03
1.99
2.00
1.04
2.00
2.00
3.01
3.00
3.00
9.0 8.5 8.0 7.5 7.0 6.5 6.0 5.5 5.0 4.5 4.0 3.5 3.0 2.5 2.0 1.5 1.0 0.5 0.0
f1 (ppm)

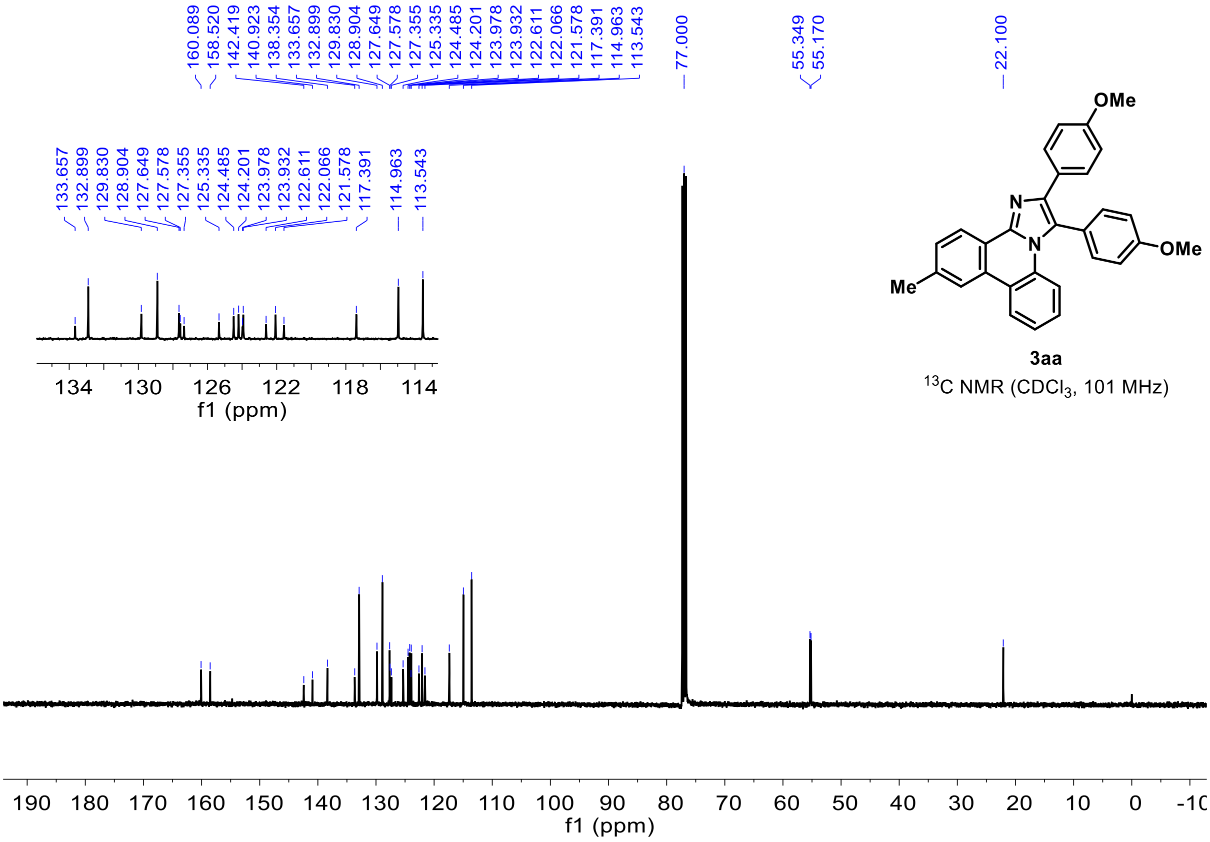
160.089
158.520
142.419
140.923
138.354
133.657
132.899
129.830
128.904
127.649
127.578
127.355
125.335
124.485
124.201
123.978
123.932
122.611
122.066
121.578
117.391
114.963
113.543
77.000
55.349
55.170
22.100
133.657
132.899
129.830
128.904
127.649
127.578
127.355
125.335
124.485
124.201
123.978
123.932
122.611
122.066
121.578
117.391
114.963
113.543
134 130 126 122 118 114
f1 (ppm)
OMe
N
N
OMe
Me
3aa
^{13}C NMR (CDCl$_3$, 101 MHz)
190 180 170 160 150 140 130 120 110 100 90 80 70 60 50 40 30 20 10 0 -10
f1 (ppm)

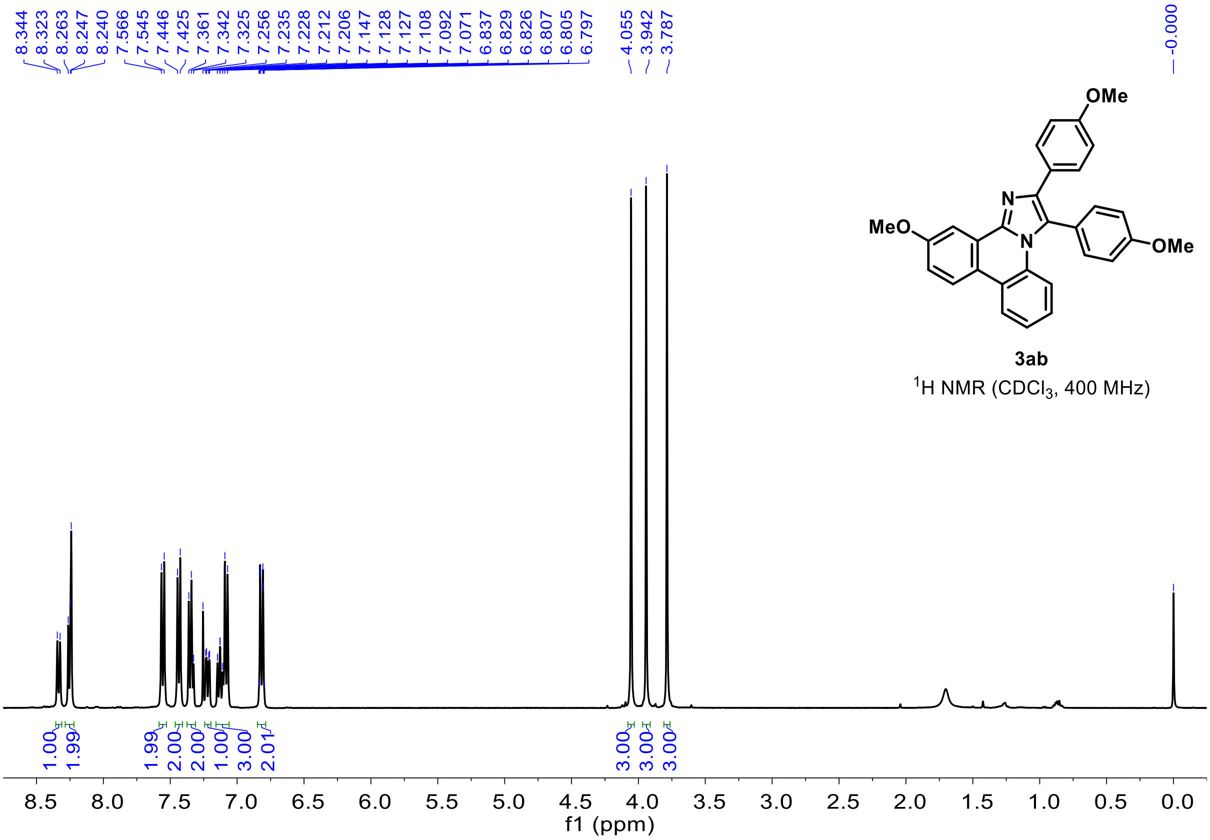

3ab

^{1}H NMR ($CDCl_3$, 400 MHz)

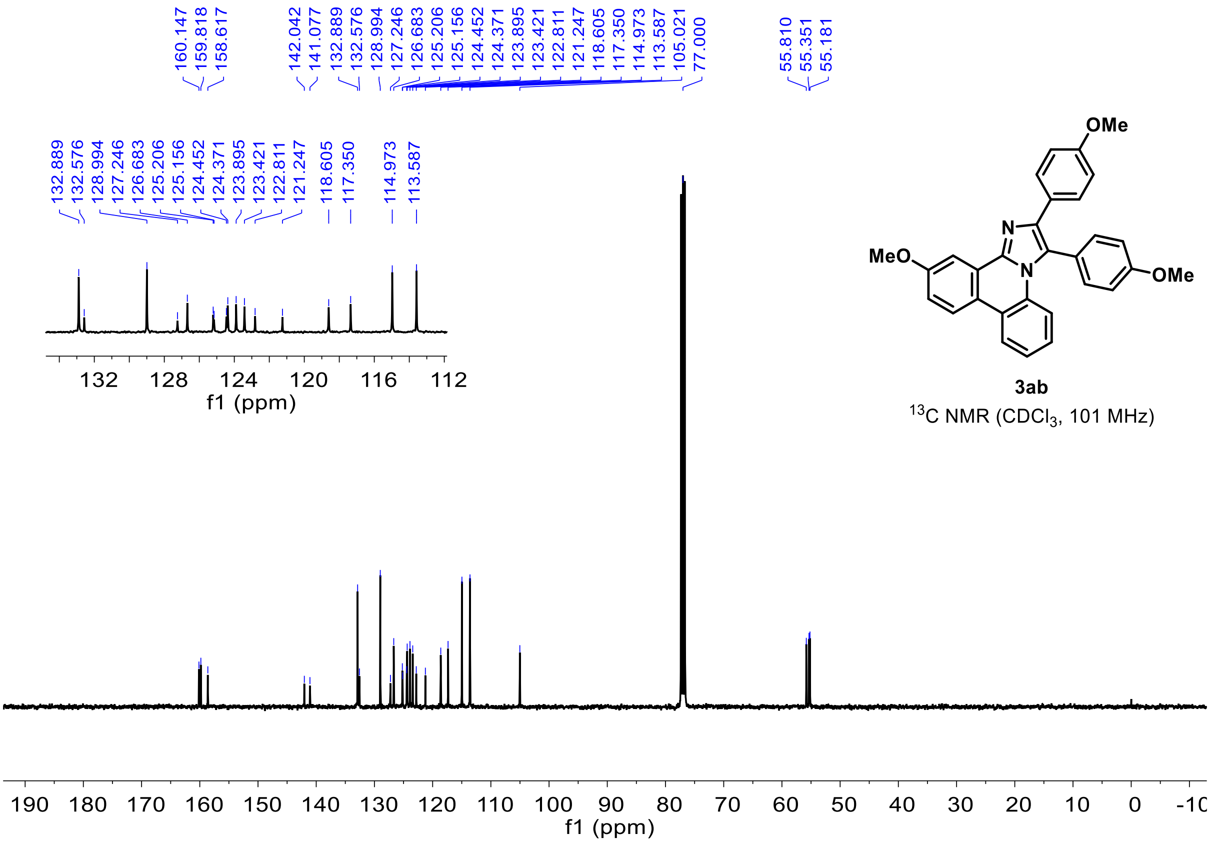

3ab

^{13}C NMR ($CDCl_3$, 101 MHz)

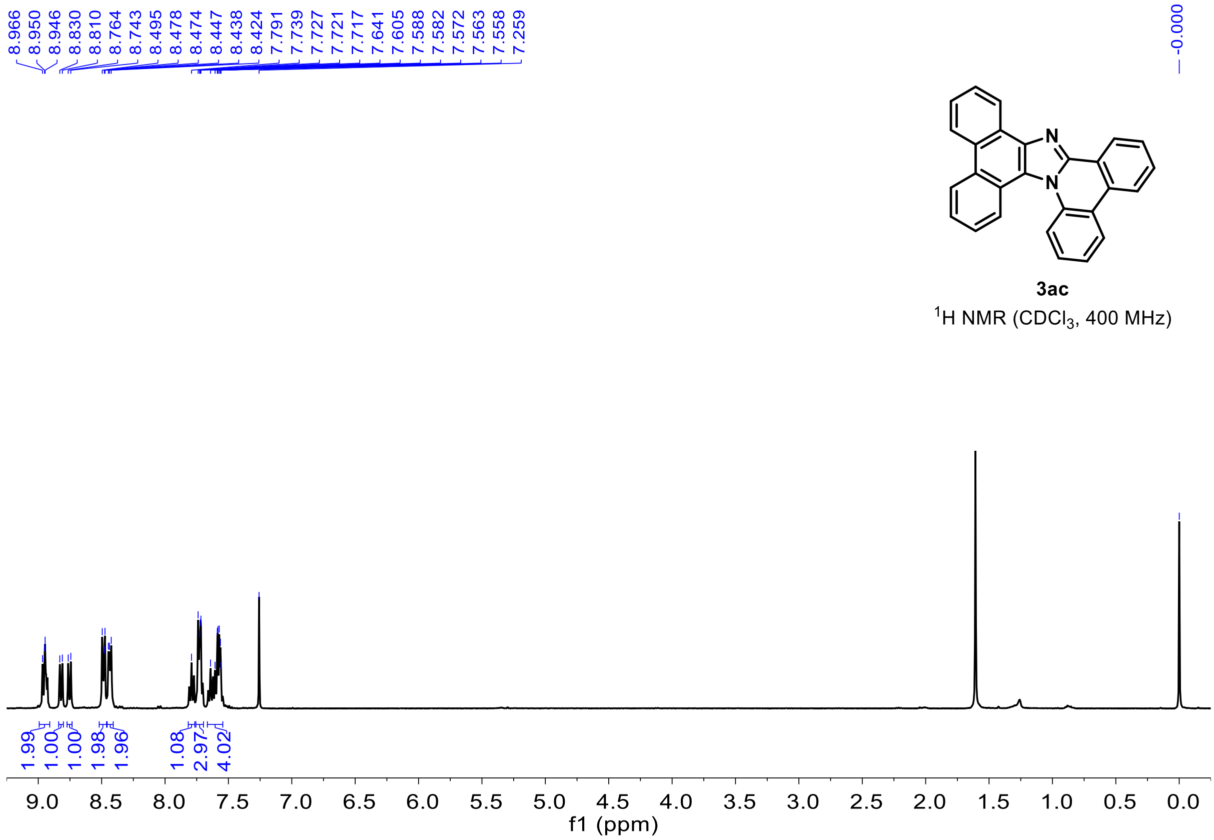
8.966 8.950 8.946 8.830 8.810 8.764 8.743 8.495 8.478 8.474 8.447 8.438 8.424 7.791 7.739 7.727 7.721 7.717 7.641 7.605 7.588 7.582 7.572 7.563 7.558 7.259
-0.000
3ac
^{1}H NMR ($CDCl_3$, 400 MHz)
1.99 1.00 1.00 1.98 1.96 1.08 2.97 4.02
9.0 8.5 8.0 7.5 7.0 6.5 6.0 5.5 5.0 4.5 4.0 3.5 3.0 2.5 2.0 1.5 1.0 0.5 0.0
f1 (ppm)

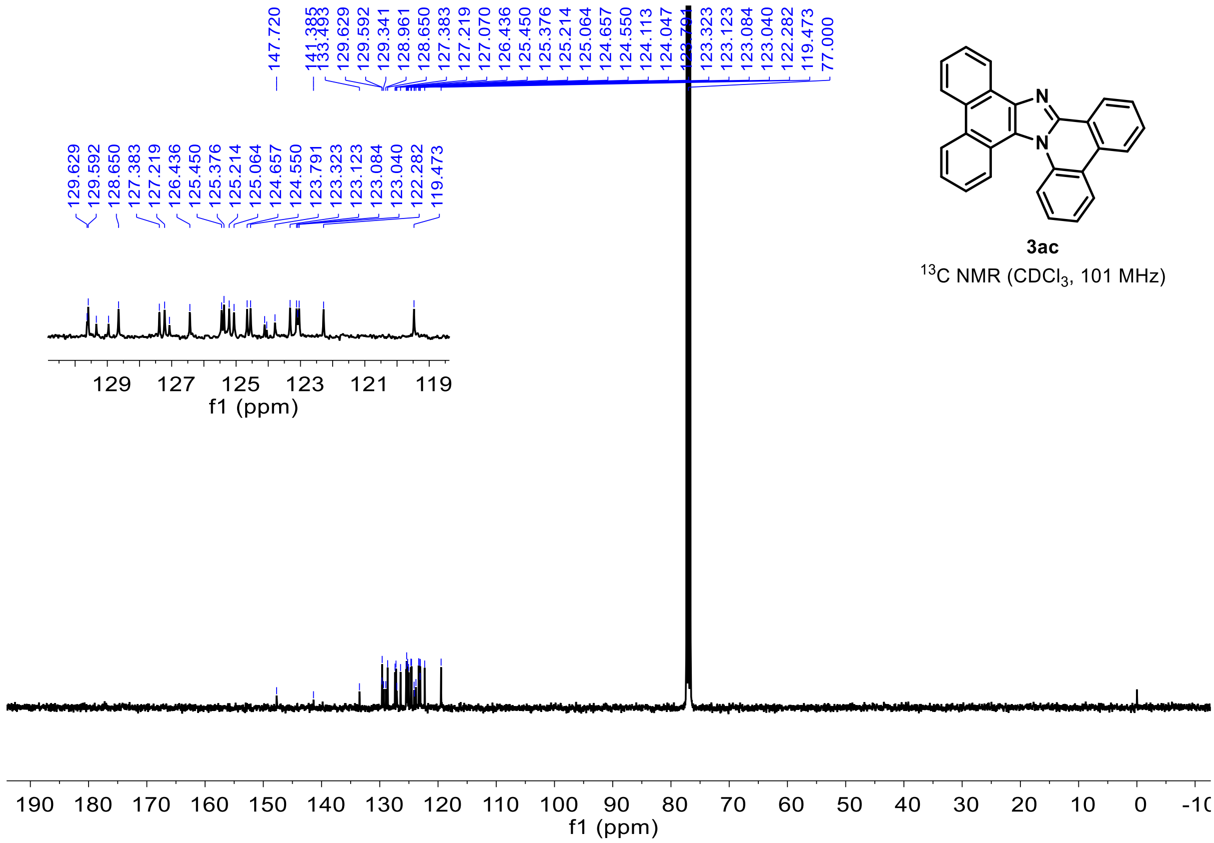
147.720
141.385 133.493 129.629 129.592 129.341 128.961 128.650 127.383 127.219 127.070 126.436 125.450 125.376 125.214 125.064 124.657 124.550 124.113 124.047 123.791 123.323 123.123 123.084 123.040 122.282 119.473 77.000
129.629 129.592 128.650 127.383 127.219 126.436 125.450 125.376 125.214 125.064 124.657 124.550 123.791 123.323 123.123 123.084 123.040 122.282 119.473
129 127 125 123 121 119
f1 (ppm)
3ac
^{13}C NMR ($CDCl_3$, 101 MHz)
190 180 170 160 150 140 130 120 110 100 90 80 70 60 50 40 30 20 10 0 -10
f1 (ppm)

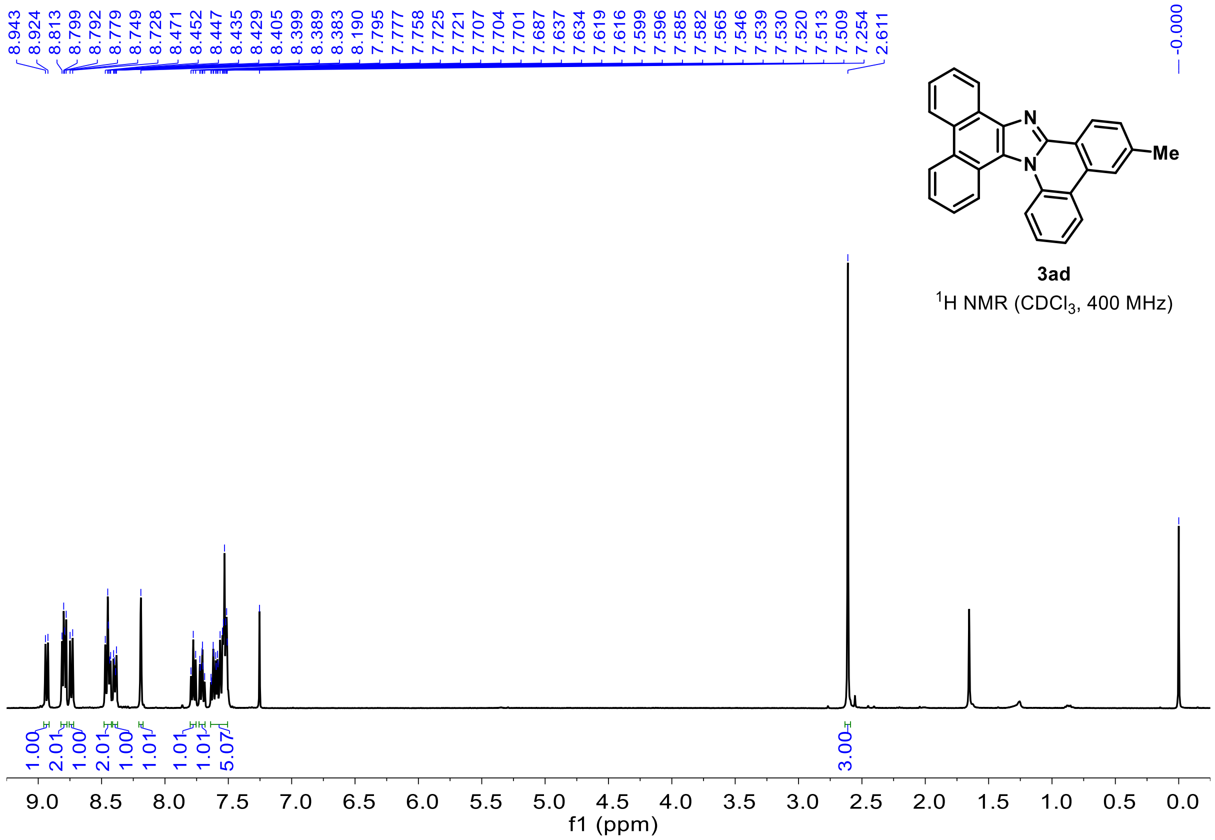
8.943
8.924
8.813
8.799
8.792
8.779
8.749
8.728
8.471
8.452
8.447
8.435
8.429
8.405
8.399
8.389
8.383
8.190
7.795
7.777
7.758
7.725
7.721
7.707
7.704
7.701
7.687
7.637
7.634
7.619
7.616
7.599
7.596
7.585
7.582
7.565
7.546
7.539
7.530
7.520
7.513
7.509
7.254
2.611
-0.000
N
N
Me
3ad
1H NMR (CDCl3, 400 MHz)
1.00
2.01
1.00
2.01
1.00
1.01
1.01
1.01
5.07
3.00
9.0 8.5 8.0 7.5 7.0 6.5 6.0 5.5 5.0 4.5 4.0 3.5 3.0 2.5 2.0 1.5 1.0 0.5 0.0
f1 (ppm)

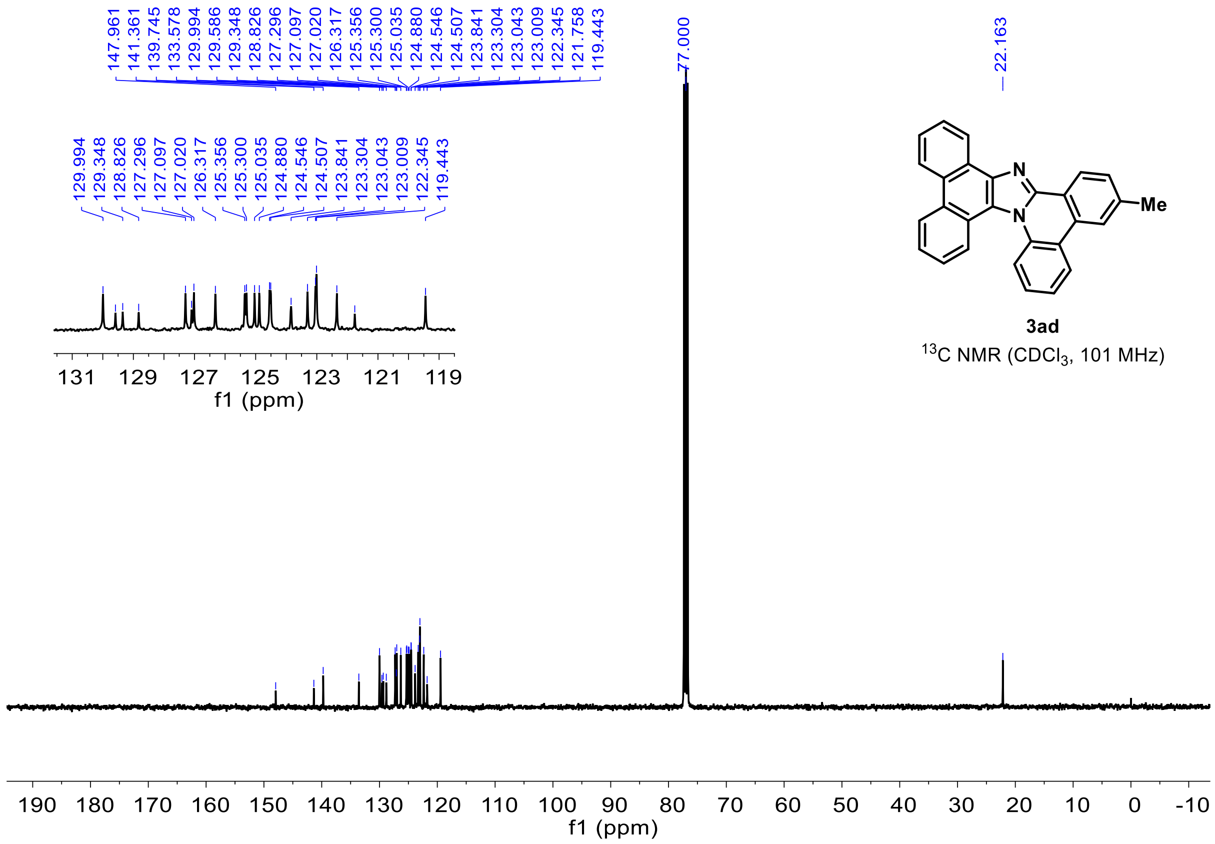
147.961
141.361
139.745
133.578
129.994
129.586
129.348
128.826
127.296
127.097
127.020
126.317
125.356
125.300
125.035
124.880
124.546
124.507
123.841
123.304
123.043
123.009
122.345
121.758
119.443
77.000
22.163
129.994
129.348
128.826
127.296
127.097
127.020
126.317
125.356
125.300
125.035
124.880
124.546
124.507
123.841
123.304
123.043
123.009
122.345
119.443
131 129 127 125 123 121 119
f1 (ppm)
N
N
Me
3ad
13C NMR (CDCl3, 101 MHz)
190 180 170 160 150 140 130 120 110 100 90 80 70 60 50 40 30 20 10 0 -10
f1 (ppm)

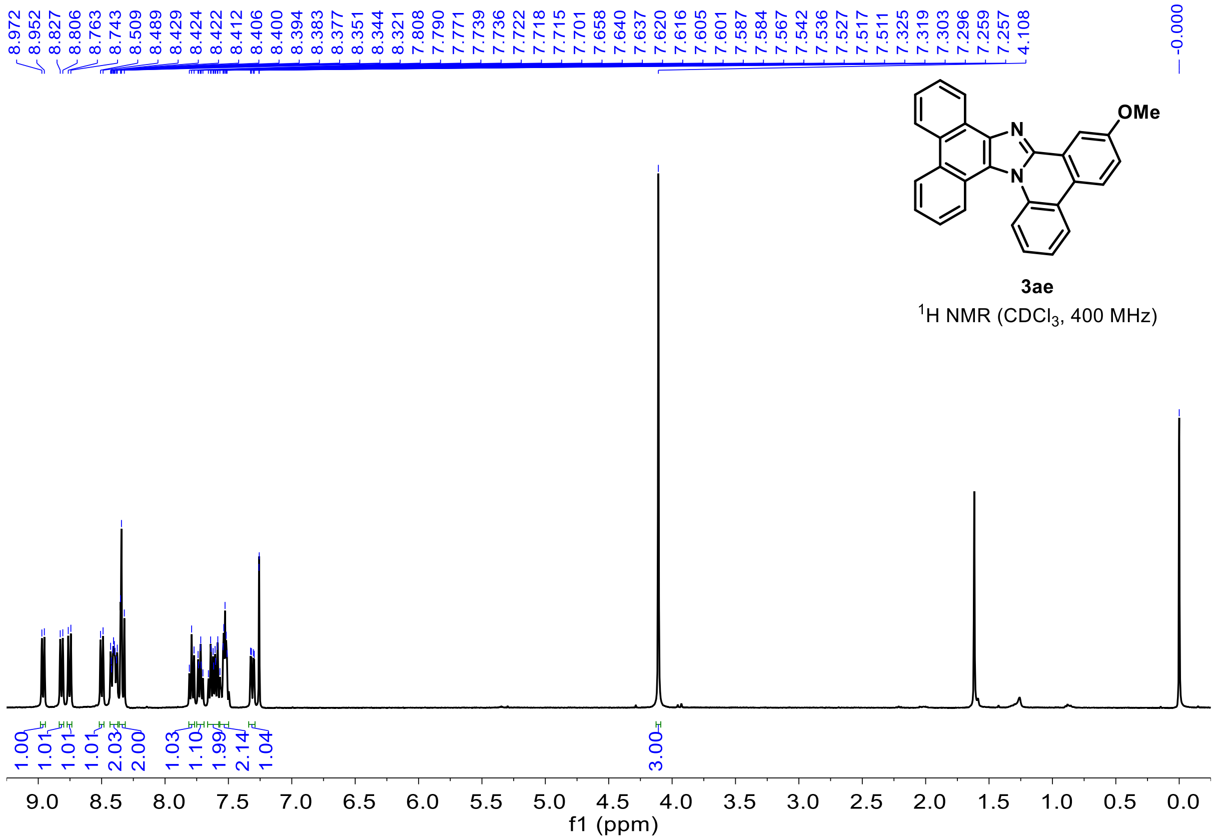
8.972 8.952 8.827 8.806 8.763 8.743 8.509 8.489 8.429 8.424 8.422 8.412 8.406 8.400 8.394 8.383 8.377 8.351 8.344 8.321 7.808 7.790 7.771 7.739 7.736 7.722 7.718 7.715 7.701 7.658 7.640 7.637 7.620 7.616 7.605 7.601 7.587 7.584 7.567 7.542 7.536 7.527 7.517 7.511 7.325 7.319 7.303 7.296 7.259 7.257 4.108
-0.000
OMe
N
N
3ae
1H NMR (CDCl3, 400 MHz)
1.00 1.01 1.01 1.01 2.03 2.00 1.03 1.10 1.99 2.14 1.04
3.00
9.0 8.5 8.0 7.5 7.0 6.5 6.0 5.5 5.0 4.5 4.0 3.5 3.0 2.5 2.0 1.5 1.0 0.5 0.0
f1 (ppm)

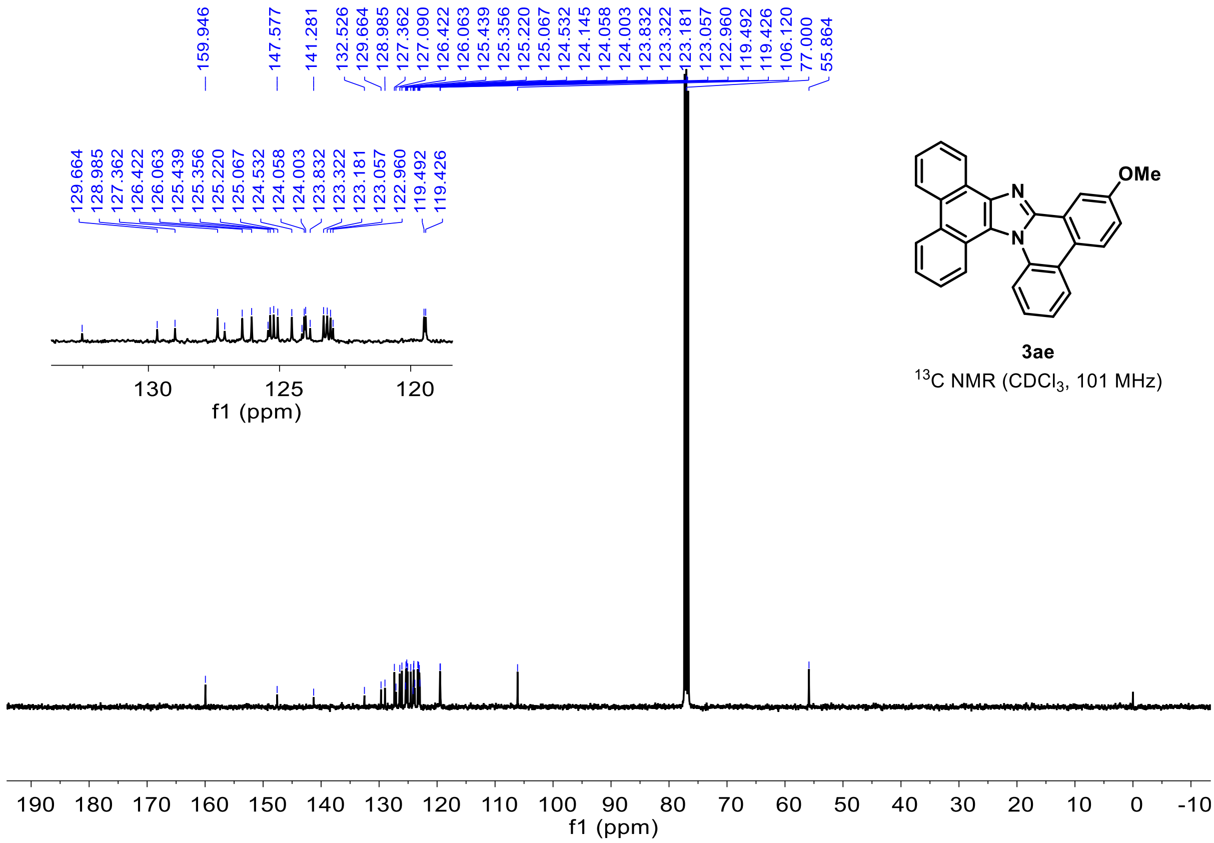
159.946
147.577
141.281
132.526 129.664 128.985 127.362 127.090 126.422 126.063 125.439 125.356 125.220 125.067 124.532 124.145 124.058 124.003 123.832 123.322 123.181 123.057 122.960 119.492 119.426 106.120 77.000 55.864
129.664 128.985 127.362 126.422 126.063 125.439 125.356 125.220 125.067 124.532 124.058 124.003 123.832 123.322 123.181 123.057 122.960 119.492 119.426
130 125 120
f1 (ppm)
OMe
N
N
3ae
13C NMR (CDCl3, 101 MHz)
190 180 170 160 150 140 130 120 110 100 90 80 70 60 50 40 30 20 10 0 -10
f1 (ppm)

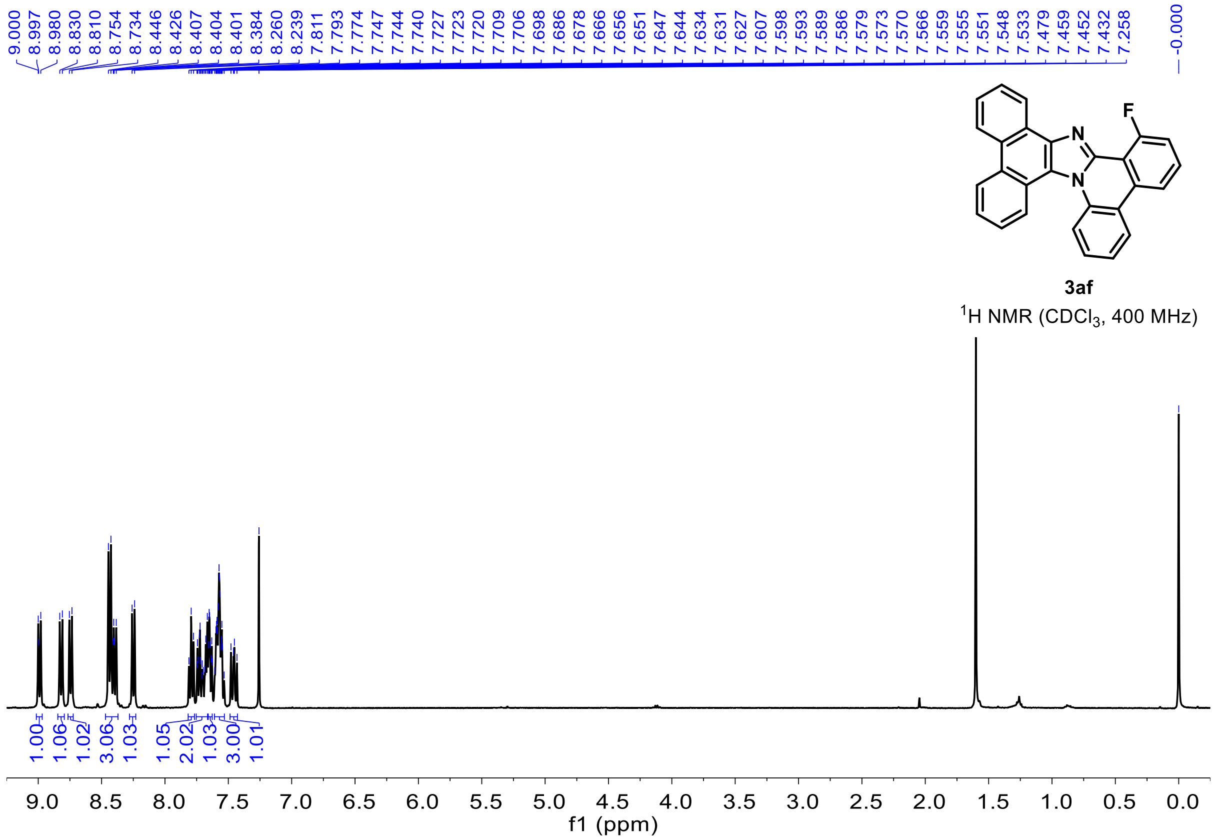
9.000 8.997 8.980 8.830 8.810 8.754 8.734 8.446 8.426 8.407 8.404 8.401 8.384 8.260 8.239 7.811 7.793 7.774 7.747 7.744 7.740 7.727 7.723 7.720 7.709 7.706 7.698 7.686 7.678 7.666 7.656 7.651 7.647 7.644 7.634 7.631 7.627 7.607 7.598 7.593 7.589 7.586 7.579 7.573 7.570 7.566 7.559 7.555 7.551 7.548 7.533 7.479 7.459 7.452 7.432 7.258
-0.000
F
N
N
3af
^{1}H NMR ($CDCl_3$, 400 MHz)
1.00 1.06 1.02 3.06 1.03 1.05 2.02 1.03 3.00 1.01
9.0 8.5 8.0 7.5 7.0 6.5 6.0 5.5 5.0 4.5 4.0 3.5 3.0 2.5 2.0 1.5 1.0 0.5 0.0
f1 (ppm)

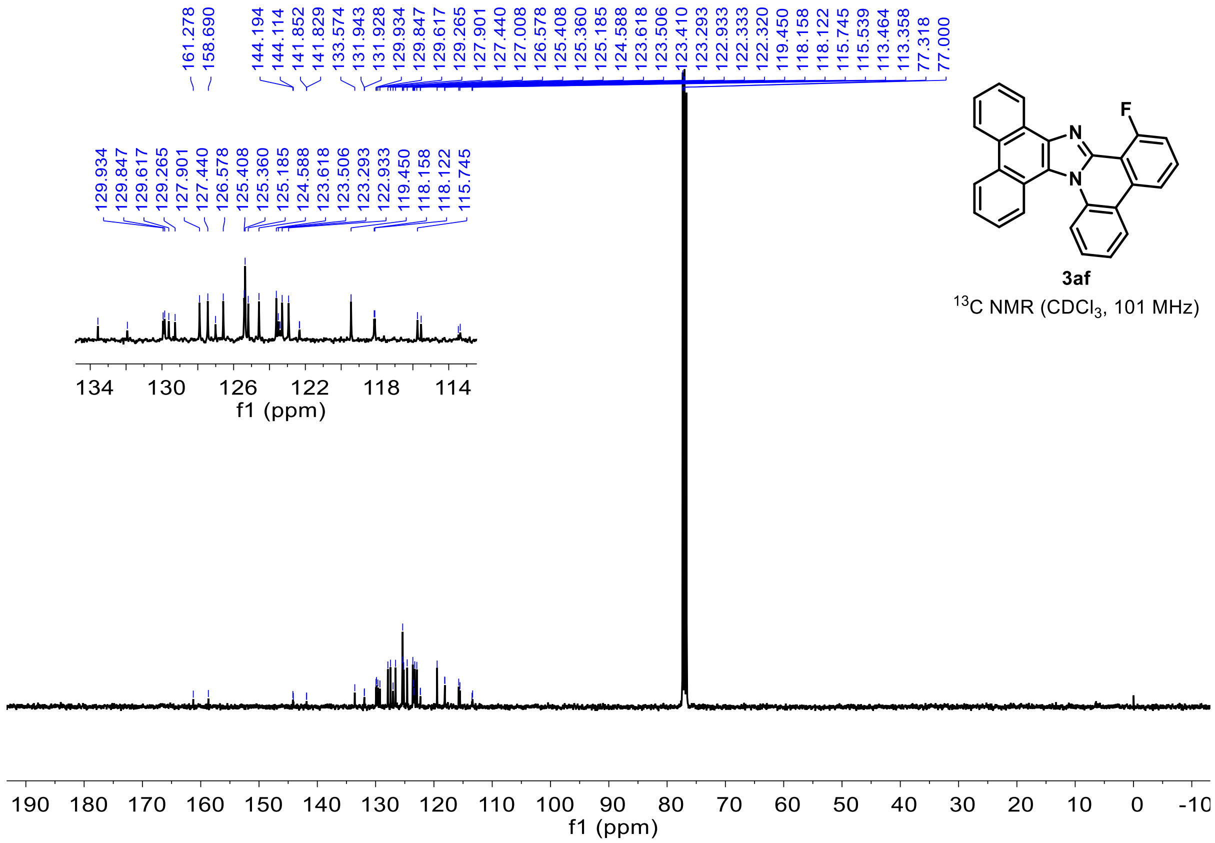
161.278 158.690
144.194 144.114 141.852 141.829 133.574 131.943 131.928 129.934 129.847 129.617 129.265 127.901 127.440 127.008 126.578 125.408 125.360 125.185 124.588 123.618 123.506 123.410 123.293 122.933 122.333 122.320 119.450 118.158 118.122 115.745 115.539 113.464 113.358 77.318 77.000
129.934 129.847 129.617 129.265 127.901 127.440 126.578 125.408 125.360 125.185 124.588 123.618 123.506 123.293 122.933 119.450 118.158 118.122 115.745
134 130 126 122 118 114
f1 (ppm)
F
N
N
3af
^{13}C NMR ($CDCl_3$, 101 MHz)
190 180 170 160 150 140 130 120 110 100 90 80 70 60 50 40 30 20 10 0 -10
f1 (ppm)

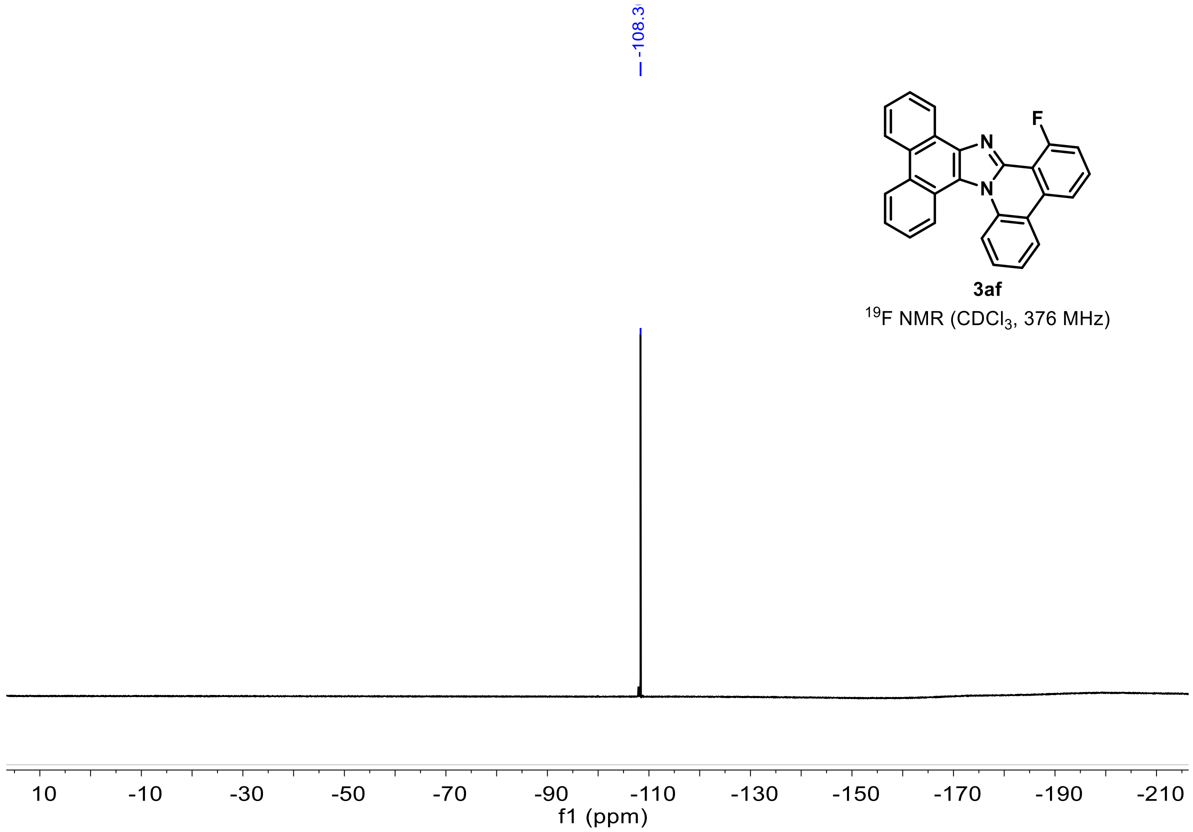

3af

^{19}F NMR ($CDCl_3$, 376 MHz)

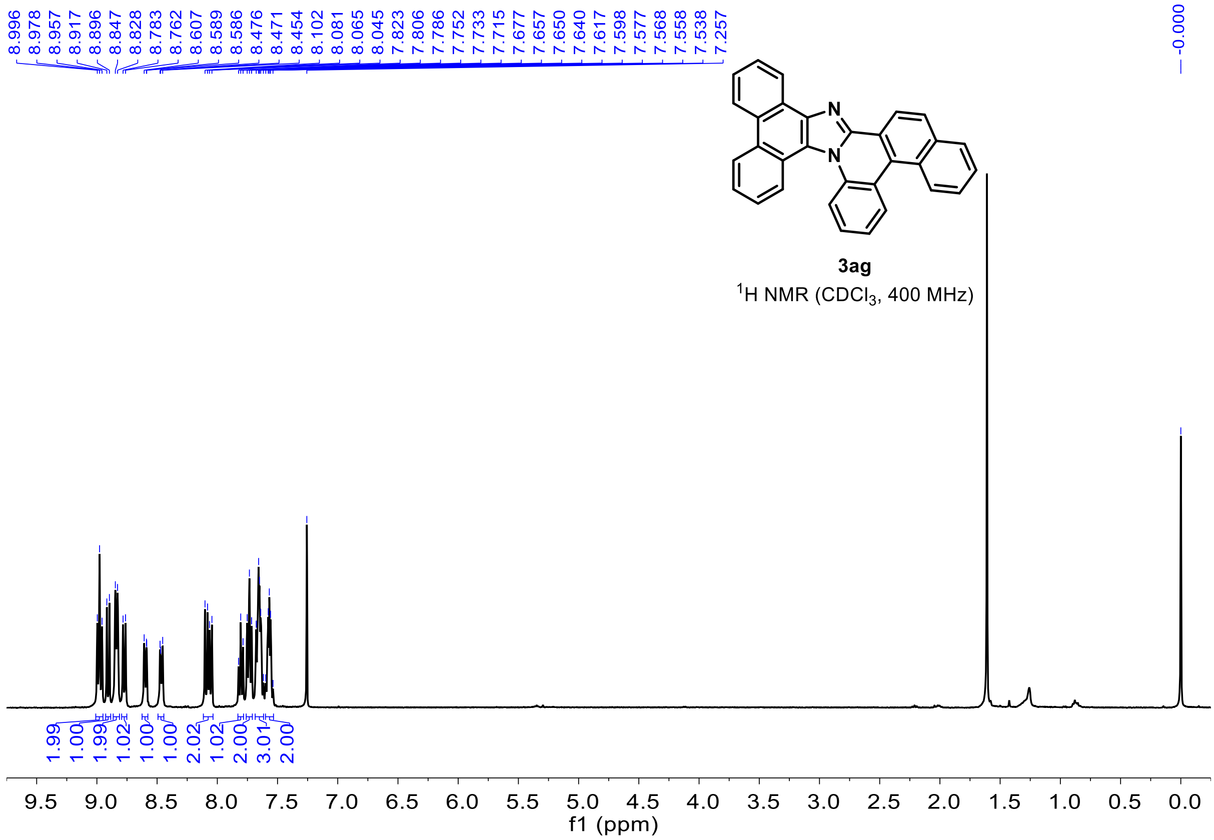
8.996 8.978 8.957 8.917 8.896 8.847 8.828 8.783 8.762 8.607 8.589 8.586 8.476 8.471 8.454 8.102 8.081 8.065 8.045 7.823 7.806 7.786 7.752 7.733 7.715 7.677 7.657 7.650 7.640 7.617 7.598 7.577 7.568 7.558 7.538 7.257
-0.000
3ag
^{1}H NMR ($CDCl_3$, 400 MHz)
1.99 1.00 1.99 1.02 1.00 1.00 2.02 1.02 2.00 3.01 2.00
9.5 9.0 8.5 8.0 7.5 7.0 6.5 6.0 5.5 5.0 4.5 4.0 3.5 3.0 2.5 2.0 1.5 1.0 0.5 0.0
f1 (ppm)

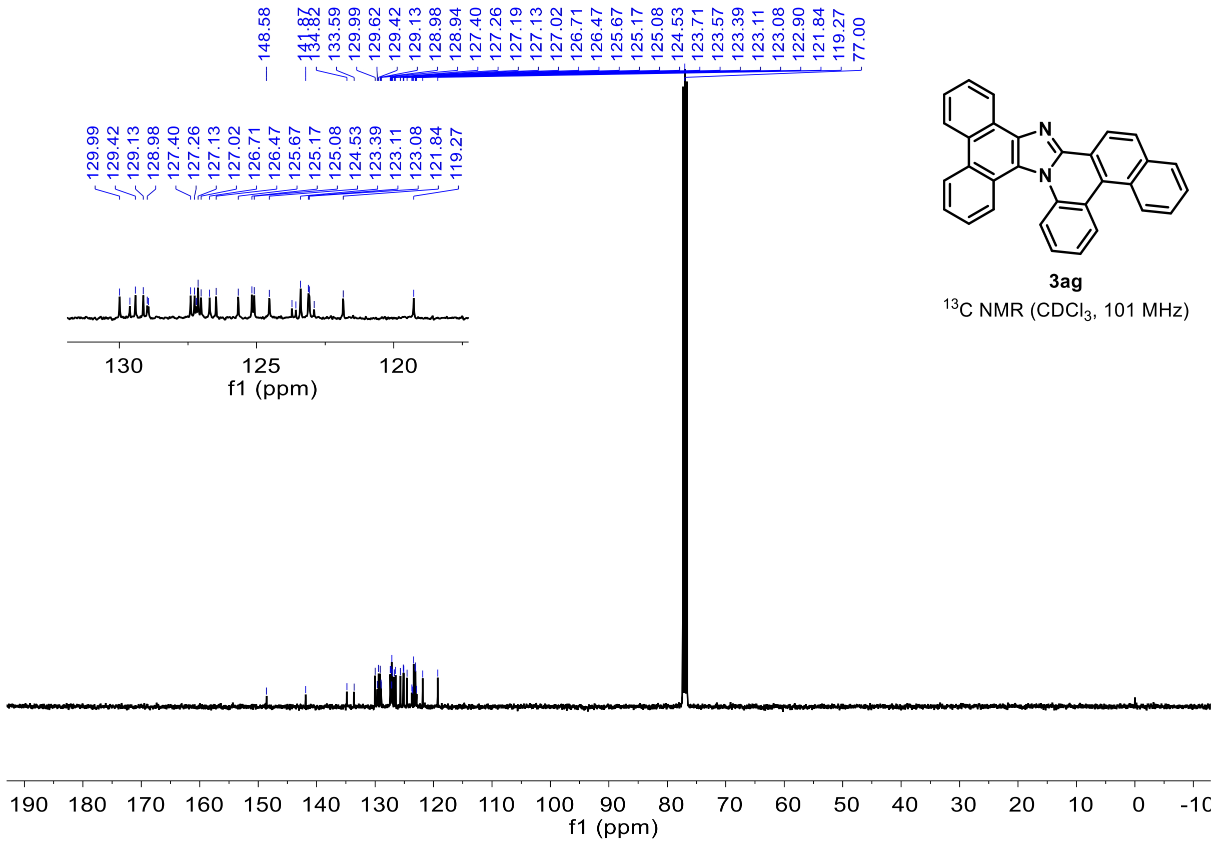
148.58
141.87 134.82 133.59 129.99 129.62 129.42 129.13 128.98 128.94 127.40 127.26 127.19 127.13 127.02 126.71 126.47 125.67 125.17 125.08 124.53 123.71 123.57 123.39 123.11 123.08 122.90 121.84 119.27 77.00
129.99 129.42 129.13 128.98 127.40 127.26 127.13 127.02 126.71 126.47 125.67 125.17 125.08 124.53 123.39 123.11 123.08 121.84 119.27
130 125 120
f1 (ppm)
3ag
^{13}C NMR ($CDCl_3$, 101 MHz)
190 180 170 160 150 140 130 120 110 100 90 80 70 60 50 40 30 20 10 0 -10
f1 (ppm)

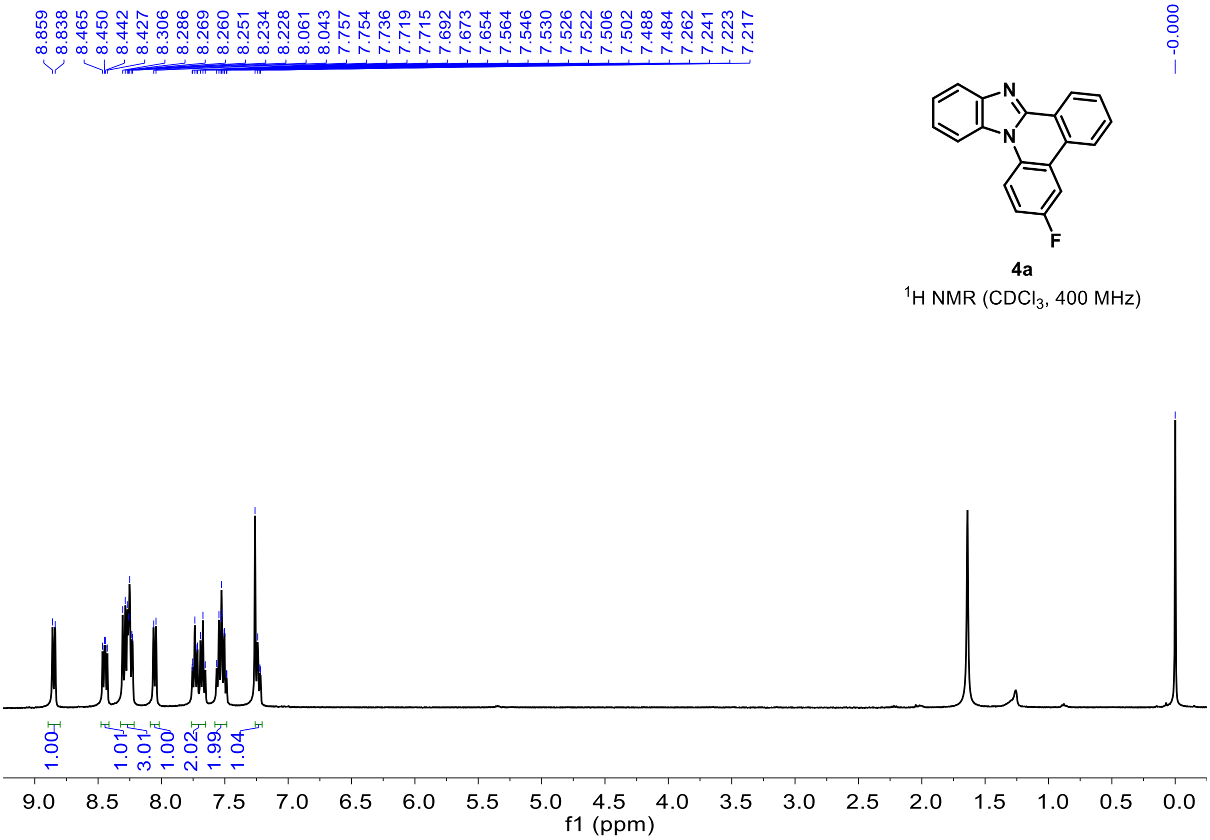
8.859
8.838
8.465
8.450
8.442
8.427
8.306
8.286
8.269
8.260
8.251
8.234
8.228
8.061
8.043
7.757
7.754
7.736
7.719
7.715
7.692
7.673
7.654
7.564
7.546
7.530
7.526
7.522
7.506
7.502
7.488
7.484
7.262
7.241
7.223
7.217
-0.000
N
N
F
4a
^{1}H NMR ($CDCl_3$, 400 MHz)
1.00
1.01
3.01
1.00
2.02
1.99
1.04
9.0 8.5 8.0 7.5 7.0 6.5 6.0 5.5 5.0 4.5 4.0 3.5 3.0 2.5 2.0 1.5 1.0 0.5 0.0
f1 (ppm)

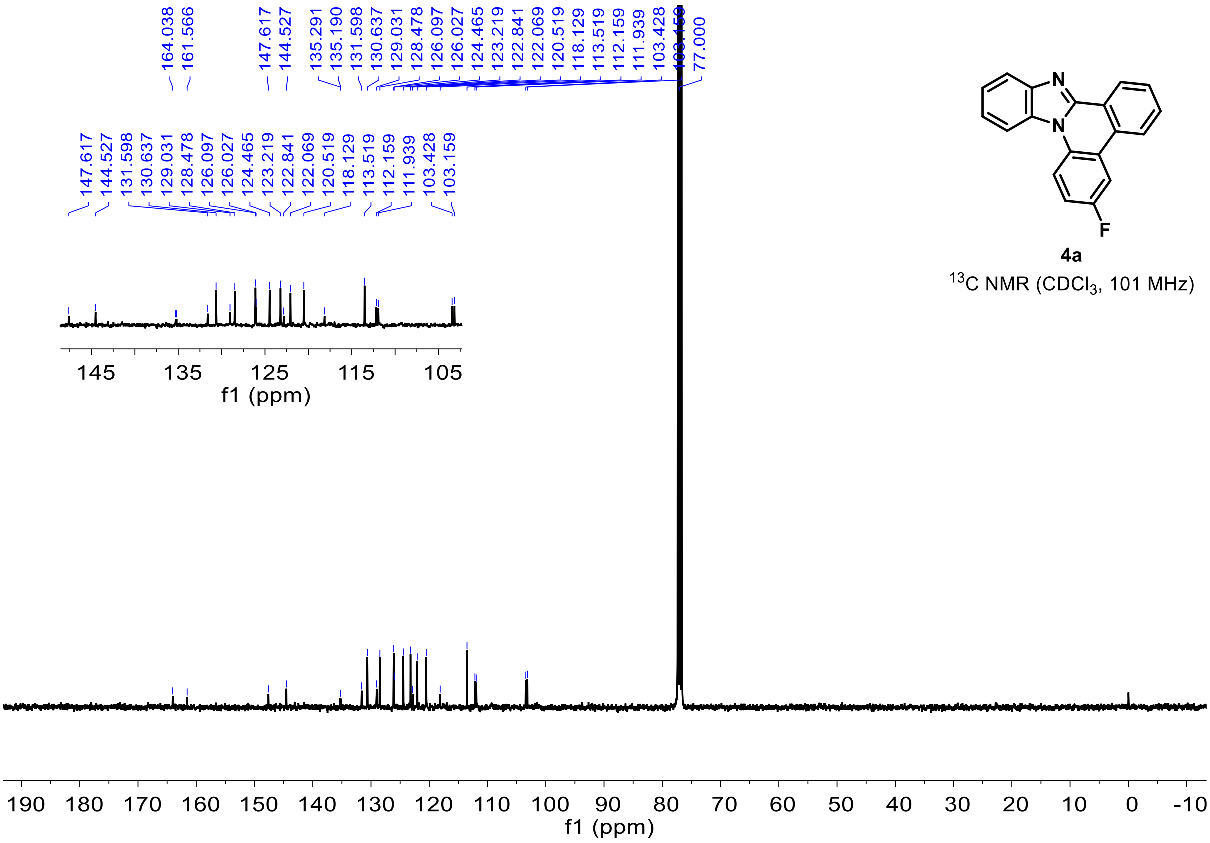
164.038
161.566
147.617
144.527
135.291
135.190
131.598
130.637
129.031
128.478
126.097
126.027
124.465
123.219
122.841
122.069
120.519
118.129
113.519
112.159
111.939
103.428
77.000
147.617
144.527
131.598
130.637
129.031
128.478
126.097
126.027
124.465
123.219
122.841
122.069
120.519
118.129
113.519
112.159
111.939
103.428
103.159
145 135 125 115 105
f1 (ppm)
N
N
F
4a
^{13}C NMR ($CDCl_3$, 101 MHz)
190 180 170 160 150 140 130 120 110 100 90 80 70 60 50 40 30 20 10 0 -10
f1 (ppm)

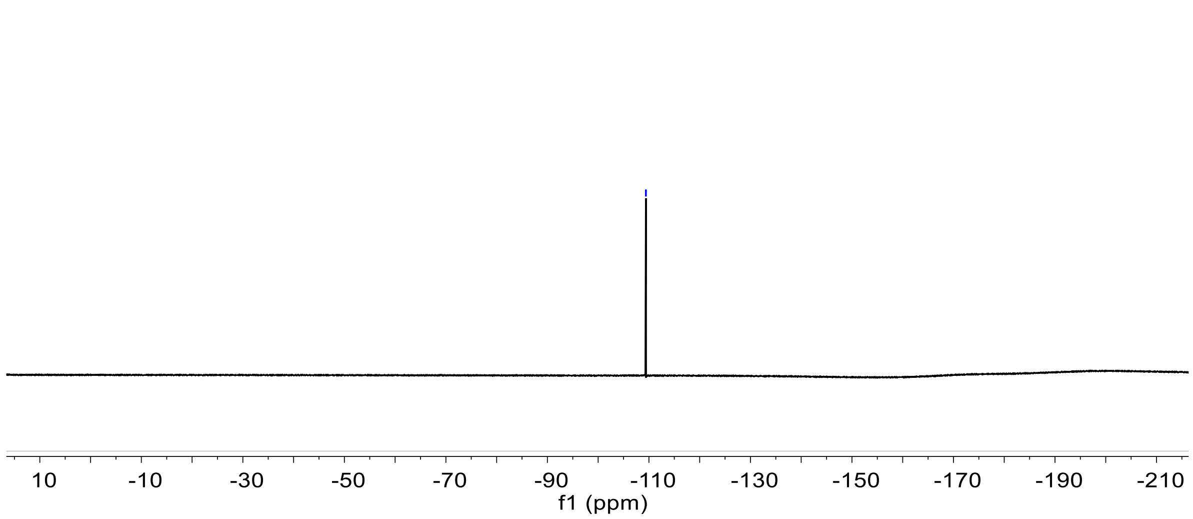

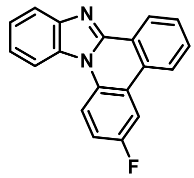

4a

^{19}F NMR ($CDCl_3$, 376 MHz)

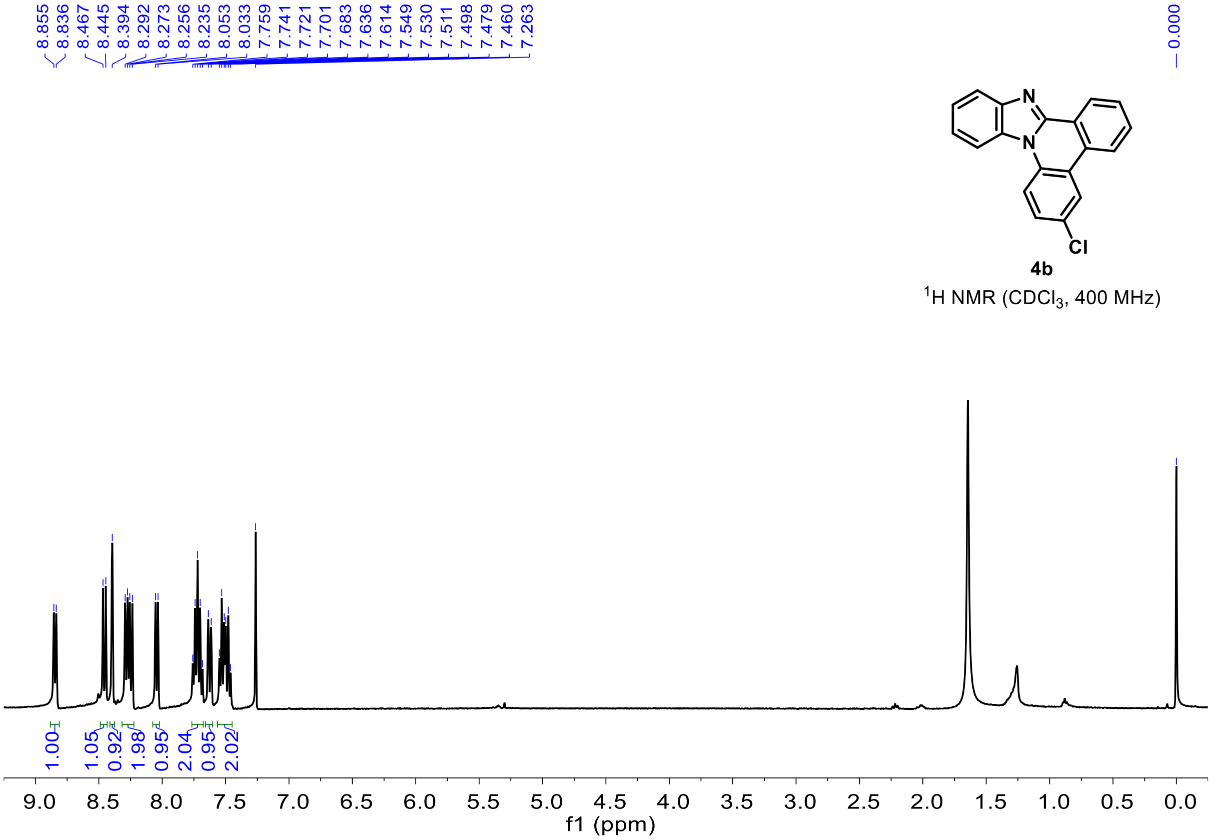

4b

^{1}H NMR ($CDCl_3$, 400 MHz)

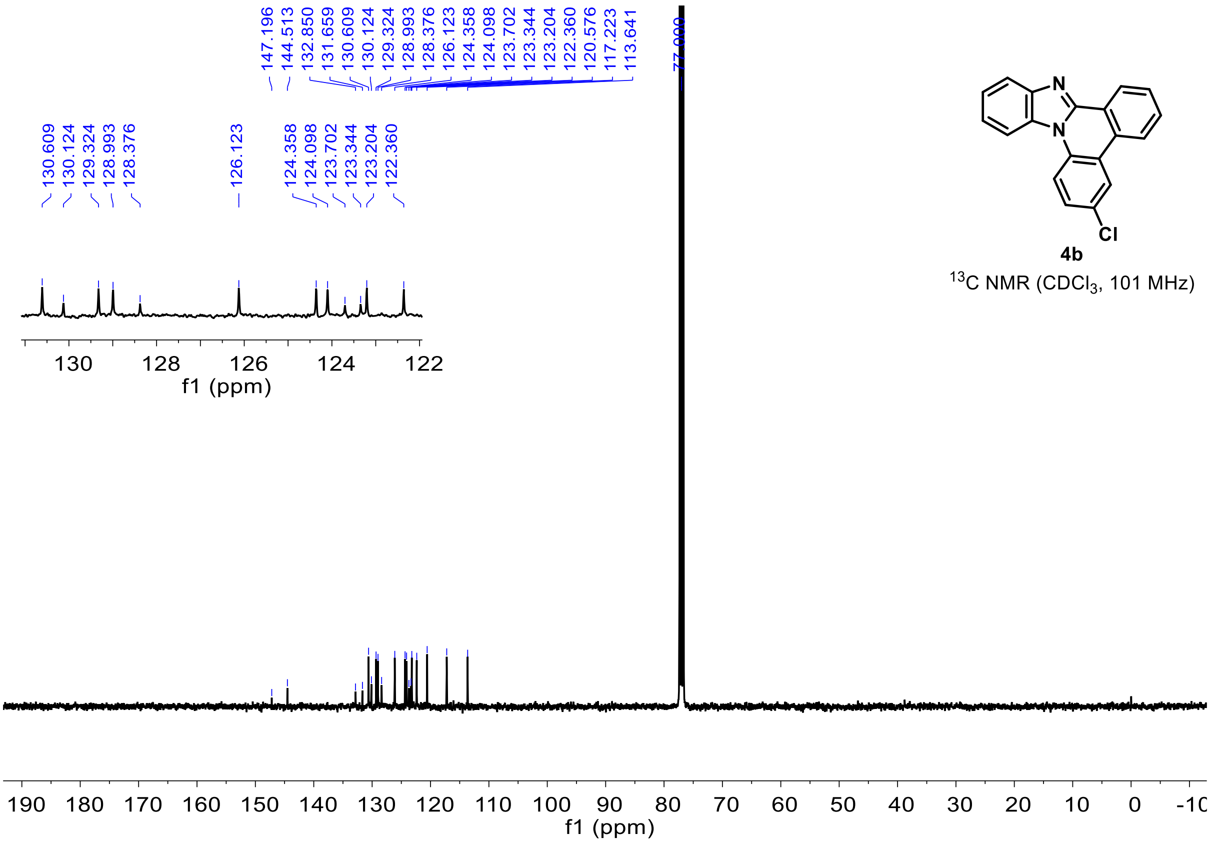

4b

^{13}C NMR ($CDCl_3$, 101 MHz)

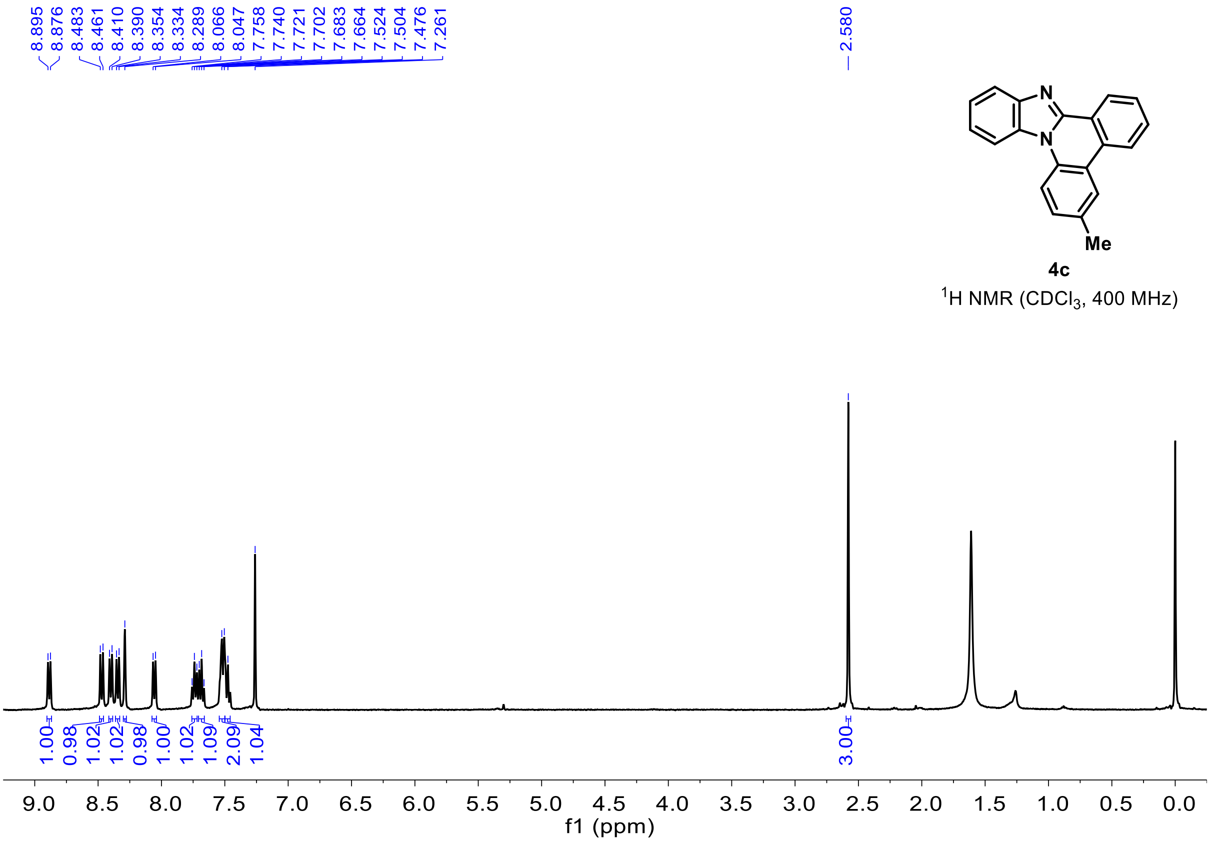
8.895
8.876
8.483
8.461
8.410
8.390
8.354
8.334
8.289
8.066
8.047
7.758
7.740
7.721
7.702
7.683
7.664
7.524
7.504
7.476
7.261
2.580
N
N
Me
4c
^{1}H NMR ($CDCl_3$, 400 MHz)
1.00
0.98
1.02
1.02
0.98
1.00
1.02
1.09
2.09
1.04
3.00
f1 (ppm)

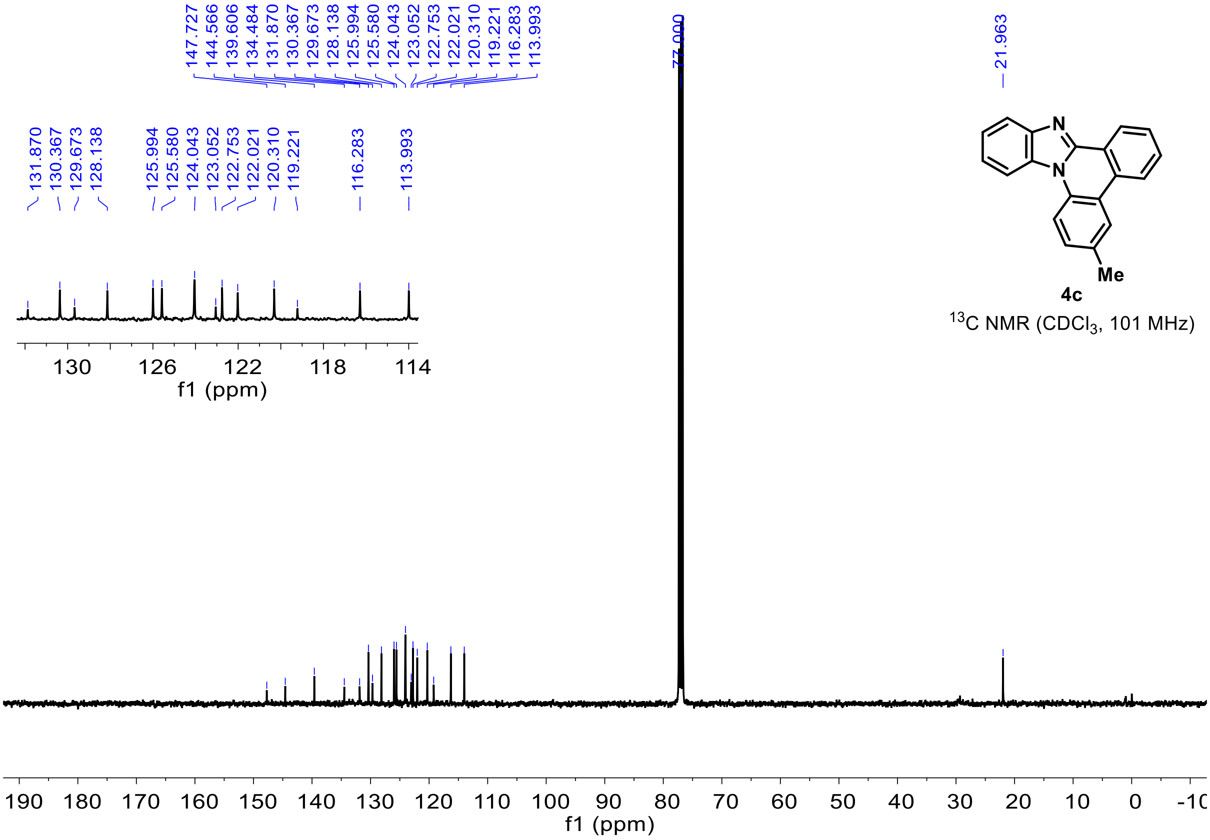
147.727
144.566
139.606
134.484
131.870
130.367
129.673
128.138
125.994
125.580
124.043
123.052
122.753
122.021
120.310
119.221
116.283
113.993
77.000
21.963
N
N
Me
4c
^{13}C NMR ($CDCl_3$, 101 MHz)
f1 (ppm)

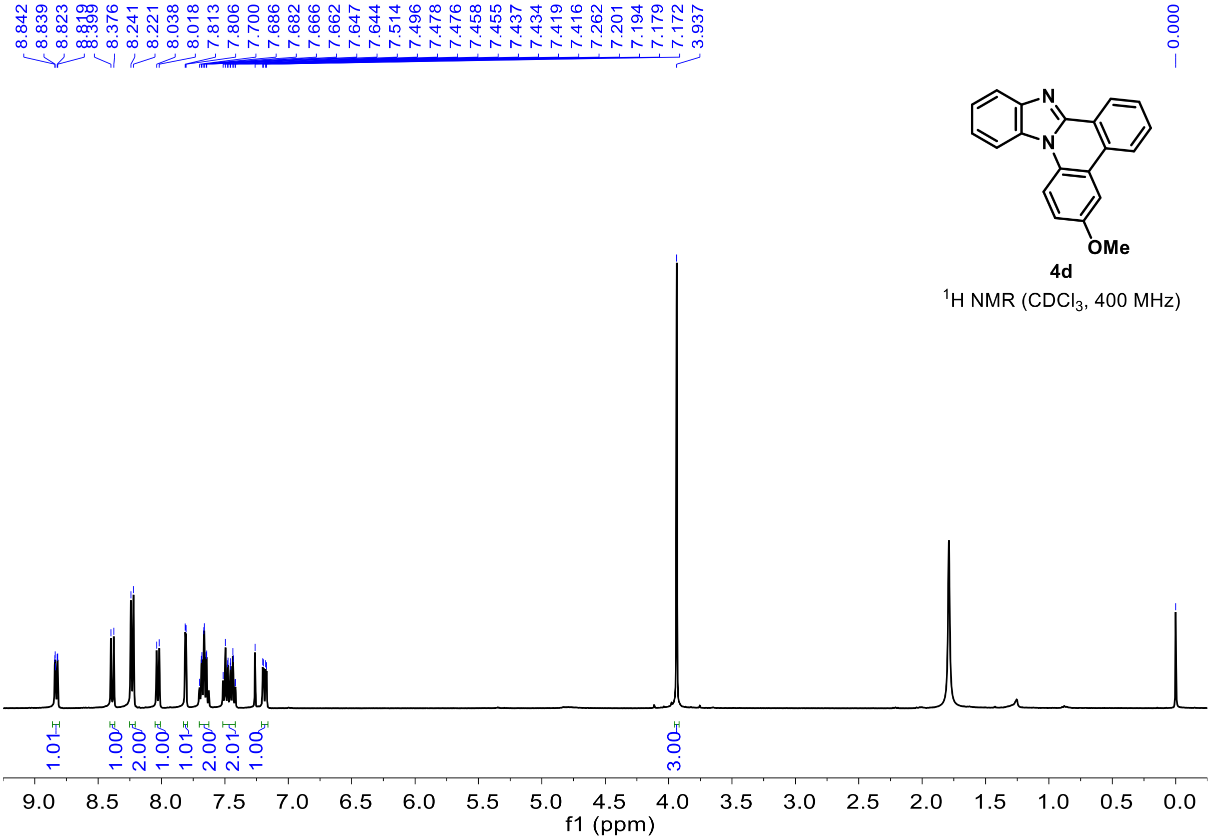
8.842
8.839
8.823
8.819
8.399
8.376
8.241
8.221
8.038
8.018
7.813
7.806
7.700
7.686
7.682
7.666
7.662
7.647
7.644
7.514
7.496
7.478
7.476
7.458
7.455
7.437
7.434
7.419
7.416
7.262
7.201
7.194
7.179
7.172
3.937
0.000
N
N
OMe
4d
1H NMR (CDCl3, 400 MHz)
1.01
1.00
2.00
1.00
1.01
2.00
2.01
1.00
3.00
9.0 8.5 8.0 7.5 7.0 6.5 6.0 5.5 5.0 4.5 4.0 3.5 3.0 2.5 2.0 1.5 1.0 0.5 0.0
f1 (ppm)

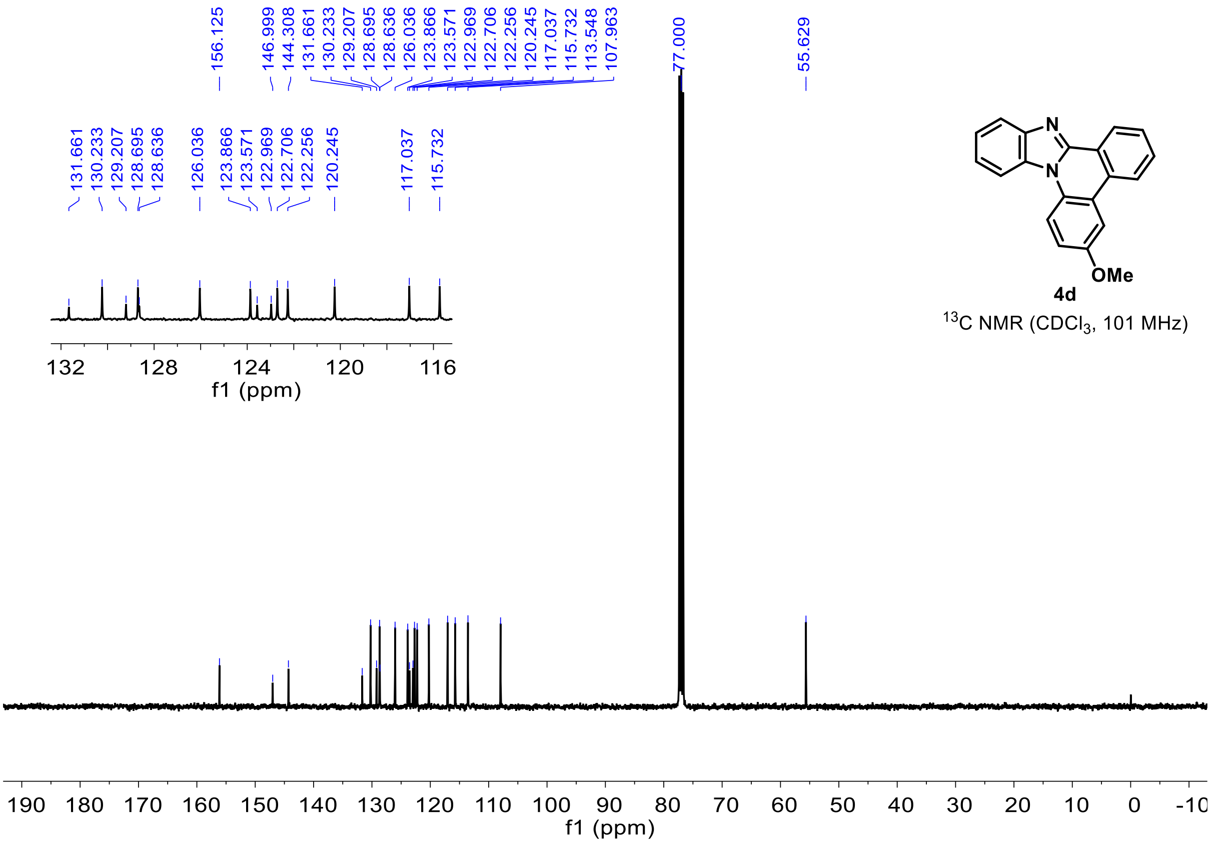
156.125
146.999
144.308
131.661
130.233
129.207
128.695
128.636
126.036
123.866
123.571
122.969
122.706
122.256
120.245
117.037
115.732
113.548
107.963
77.000
55.629
131.661
130.233
129.207
128.695
128.636
126.036
123.866
123.571
122.969
122.706
122.256
120.245
117.037
115.732
132 128 124 120 116
f1 (ppm)
N
N
OMe
4d
13C NMR (CDCl3, 101 MHz)
190 180 170 160 150 140 130 120 110 100 90 80 70 60 50 40 30 20 10 0 -10
f1 (ppm)

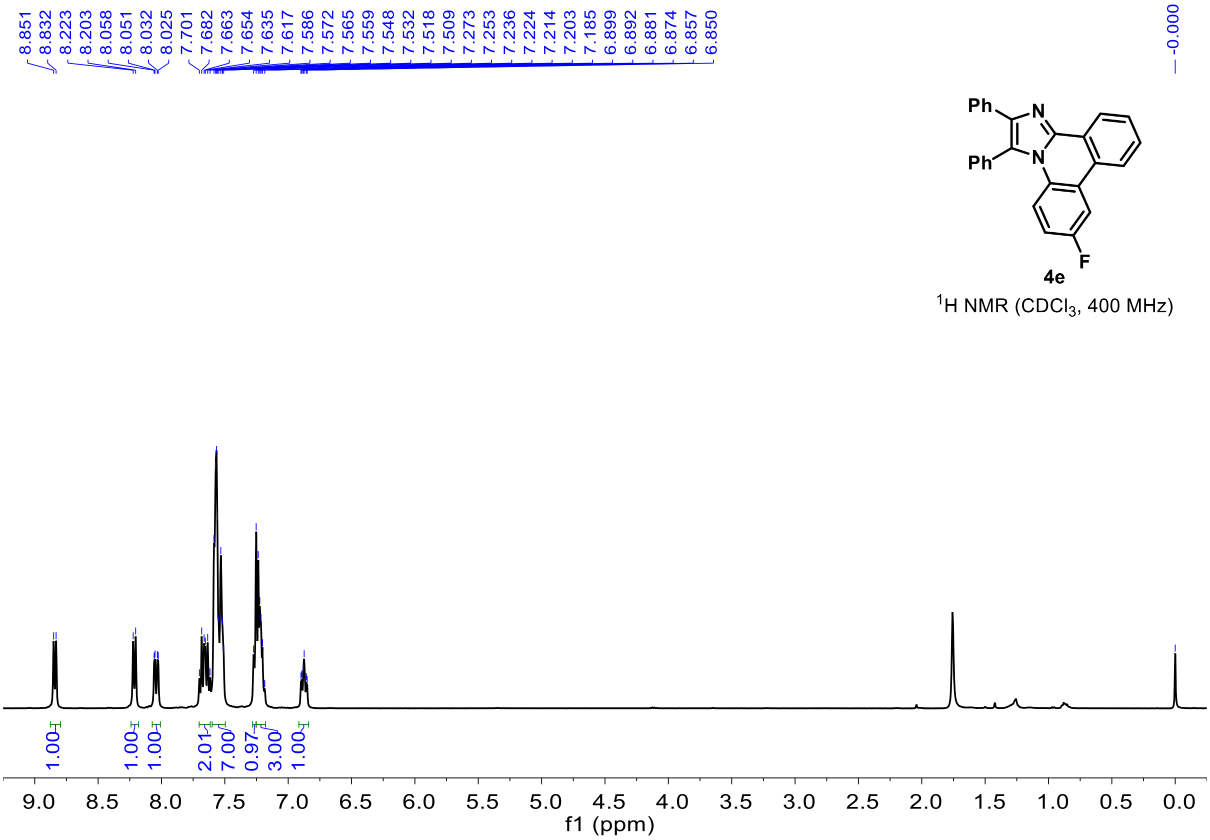

4e

^{1}H NMR ($CDCl_3$, 400 MHz)

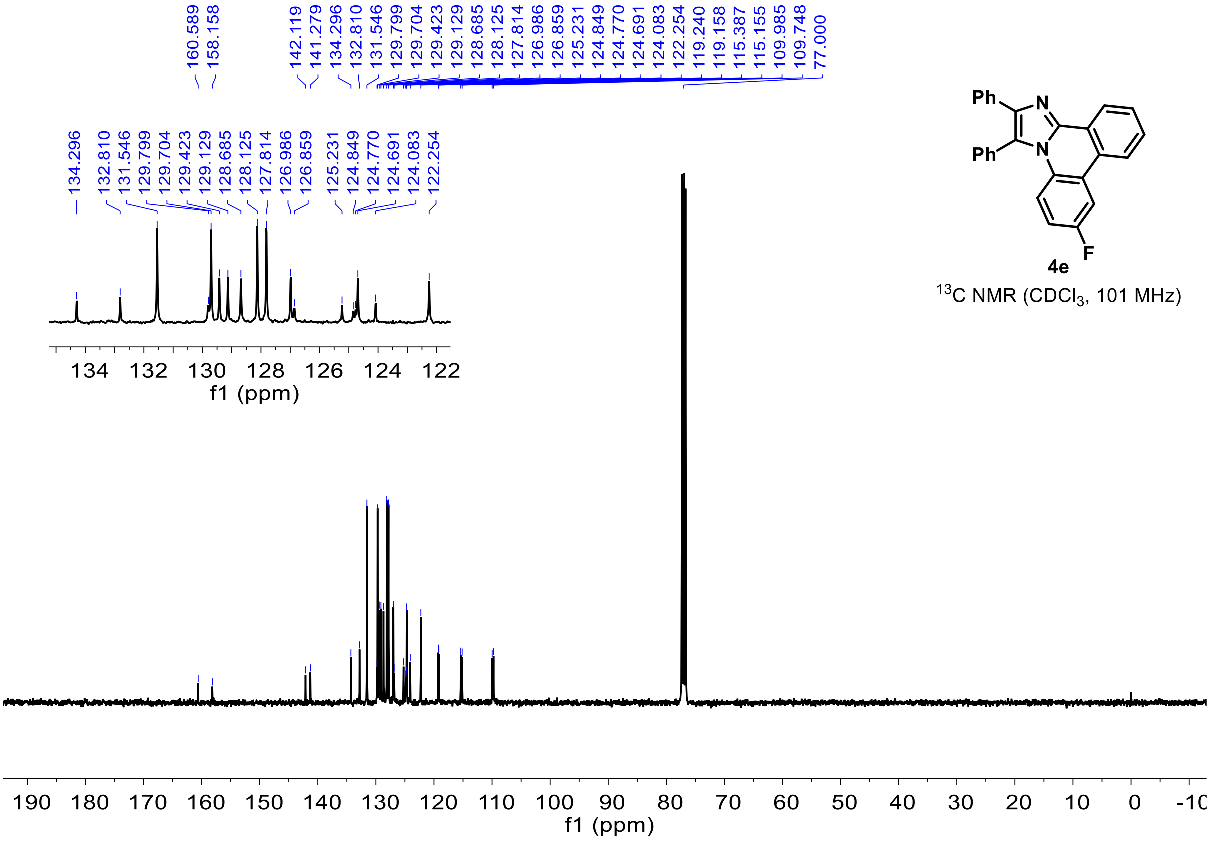

4e

^{13}C NMR ($CDCl_3$, 101 MHz)

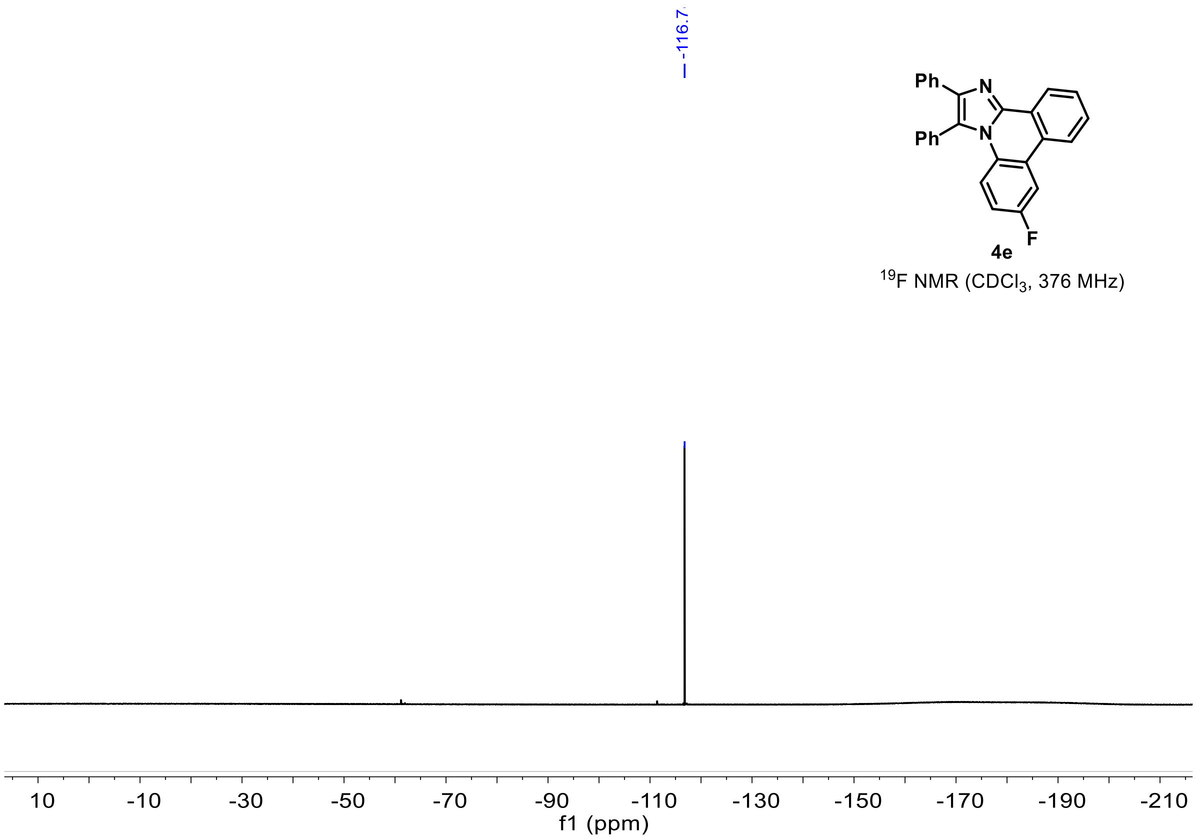

4e

^{19}F NMR ($CDCl_3$, 376 MHz)

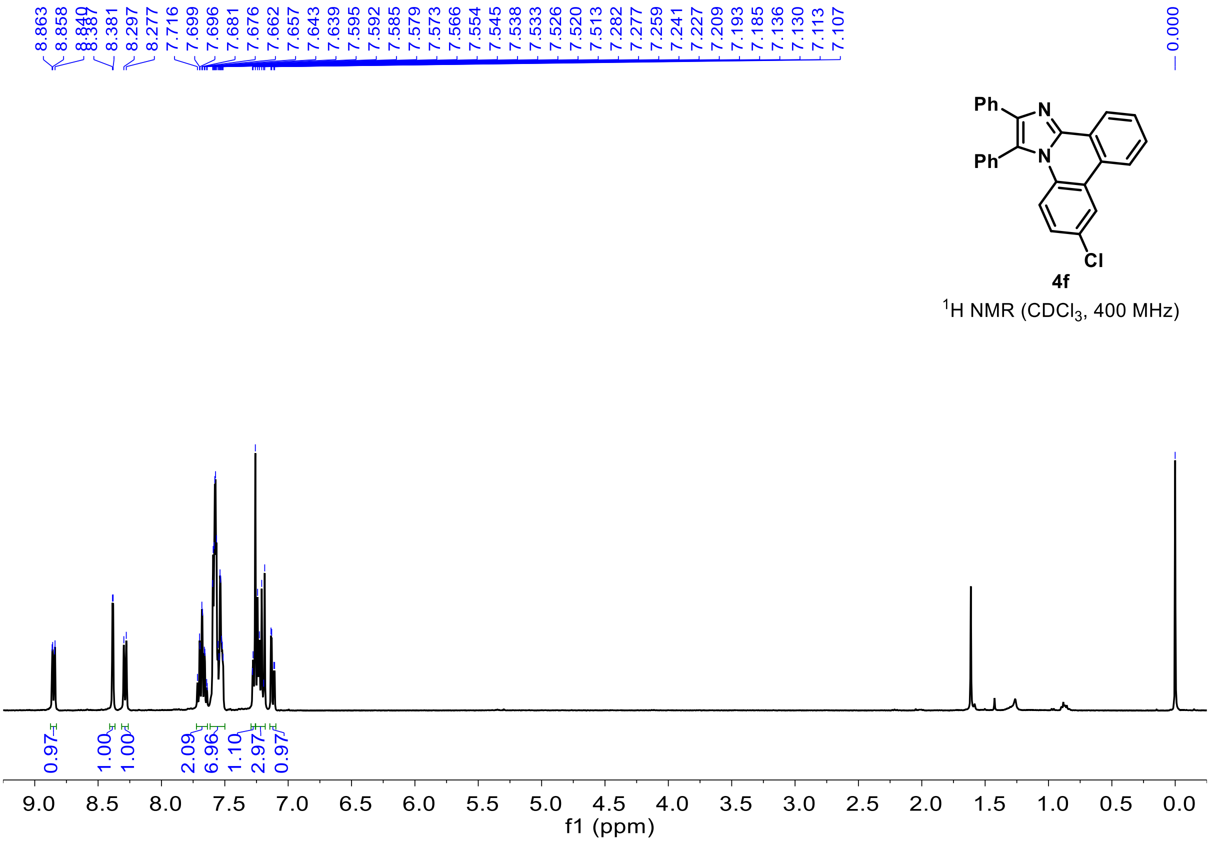
8.863
8.858
8.840
8.387
8.381
8.297
8.277
7.716
7.699
7.696
7.681
7.676
7.662
7.657
7.643
7.639
7.595
7.592
7.585
7.579
7.573
7.566
7.554
7.545
7.538
7.533
7.526
7.520
7.513
7.282
7.277
7.259
7.241
7.227
7.209
7.193
7.185
7.136
7.130
7.113
7.107
0.000
Ph
N
Ph
N
Cl
4f
^{1}H NMR (CDCl$_3$, 400 MHz)
0.97
1.00
1.00
2.09
6.96
1.10
2.97
0.97
f1 (ppm)

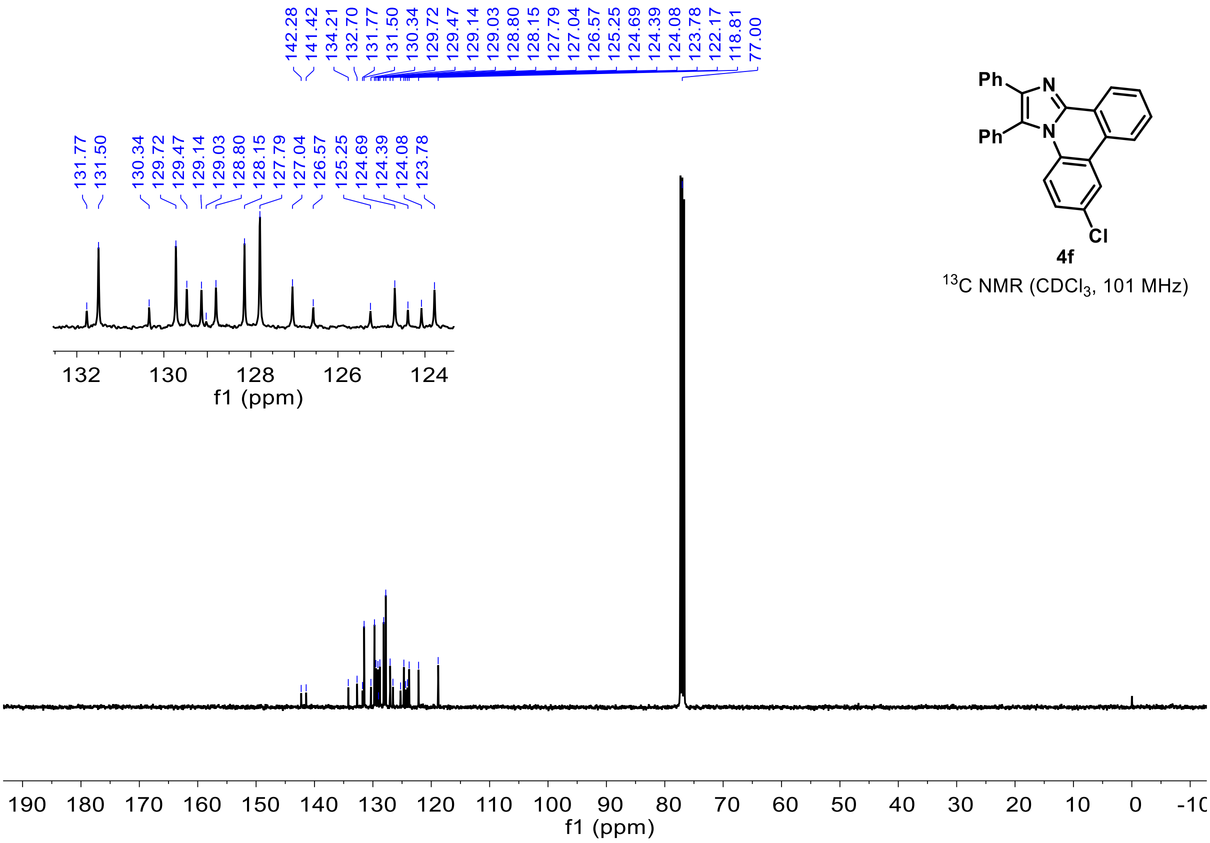
142.28
141.42
134.21
132.70
131.77
131.50
130.34
129.72
129.47
129.14
129.03
128.80
128.15
127.79
127.04
126.57
125.25
124.69
124.39
124.08
123.78
122.17
118.81
77.00
Ph
N
Ph
N
Cl
4f
^{13}C NMR (CDCl$_3$, 101 MHz)
f1 (ppm)

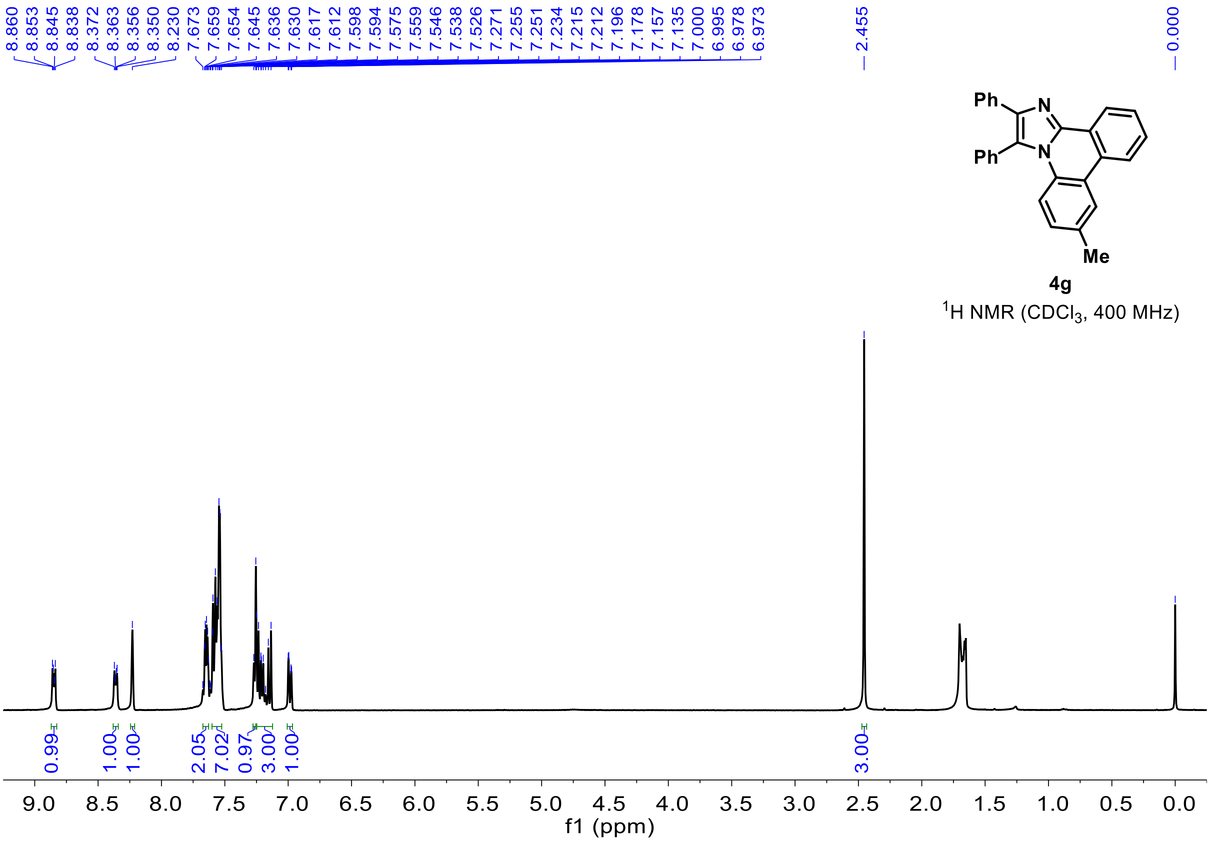

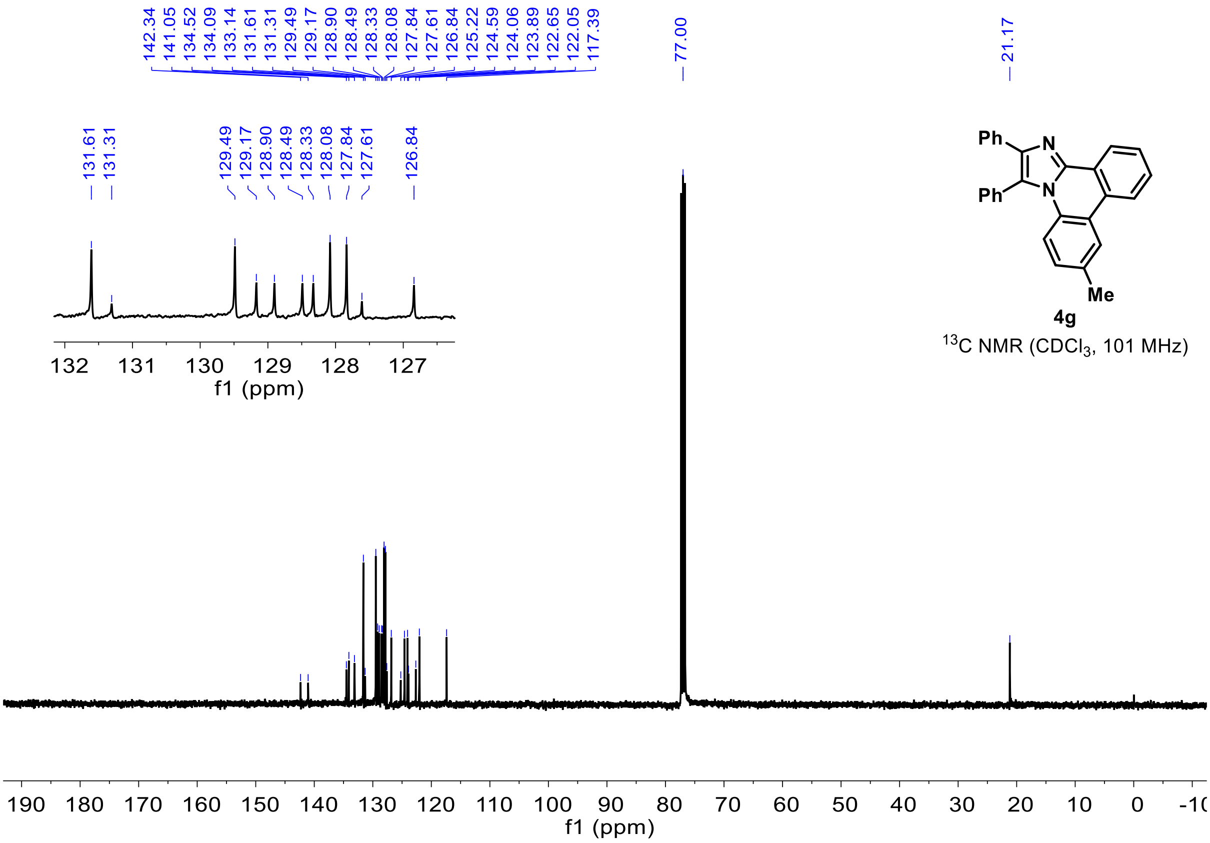

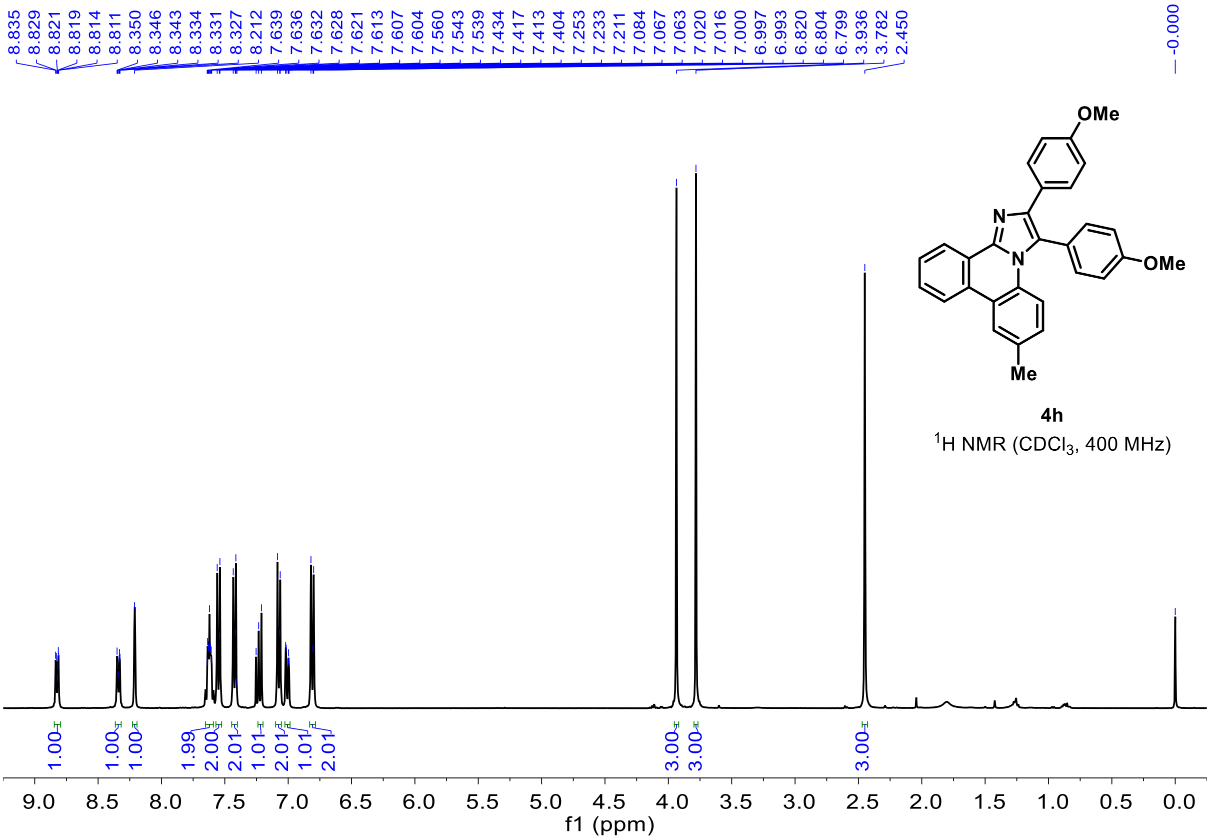

4h

^{1}H NMR ($CDCl_3$, 400 MHz)

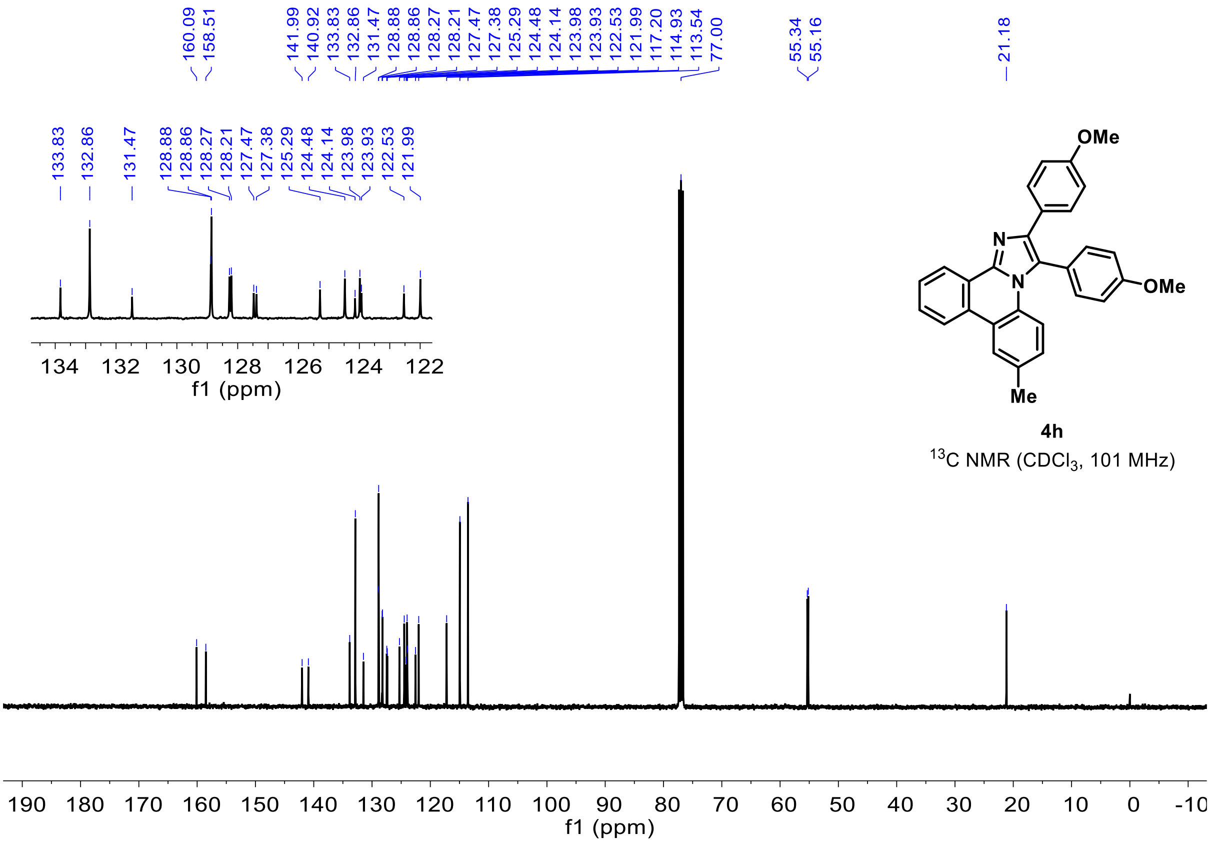

4h

^{13}C NMR ($CDCl_3$, 101 MHz)

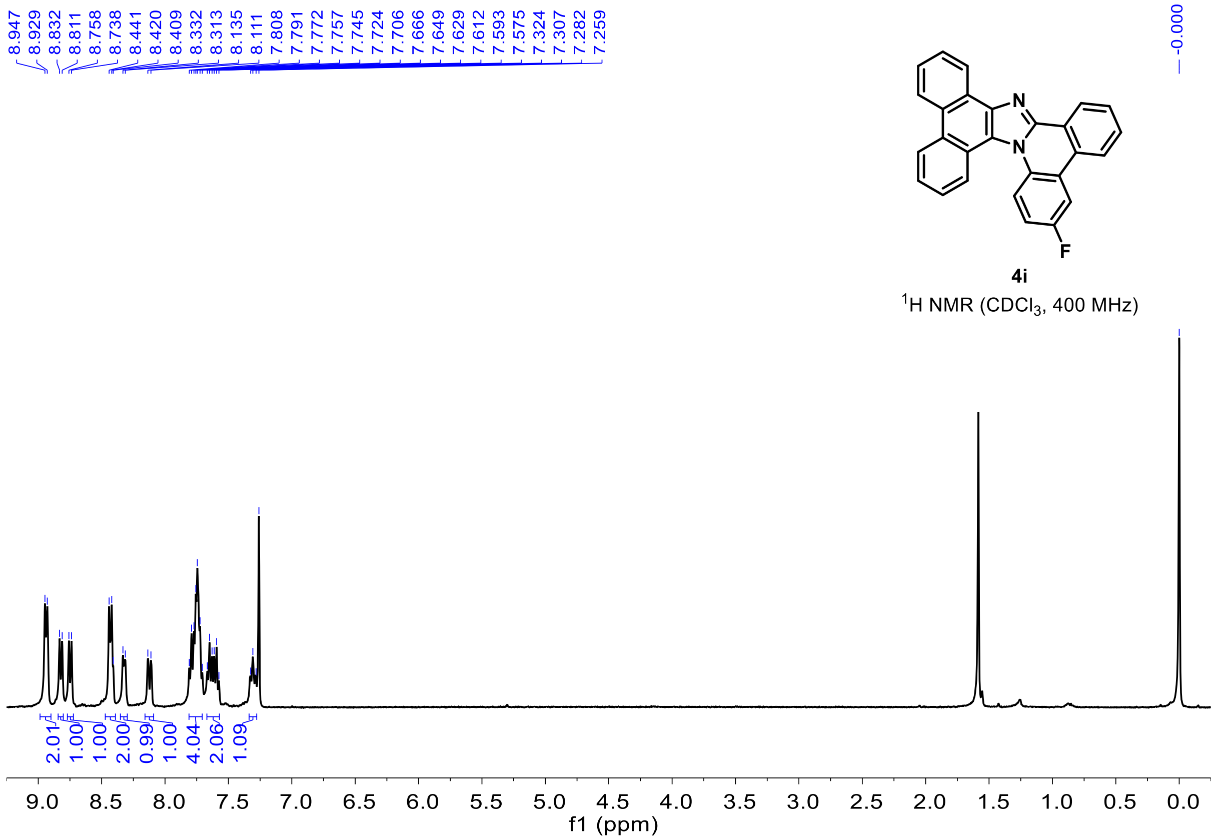

8.947
8.929
8.832
8.811
8.758
8.738
8.441
8.420
8.409
8.332
8.313
8.135
8.111
7.808
7.791
7.772
7.757
7.745
7.724
7.706
7.666
7.649
7.629
7.612
7.593
7.575
7.324
7.307
7.282
7.259
-0.000
N
N
F
4i
^{1}H NMR (CDCl$_3$, 400 MHz)
2.01
1.00
1.00
2.00
0.99
1.00
4.04
2.06
1.09
9.0 8.5 8.0 7.5 7.0 6.5 6.0 5.5 5.0 4.5 4.0 3.5 3.0 2.5 2.0 1.5 1.0 0.5 0.0
f1 (ppm)

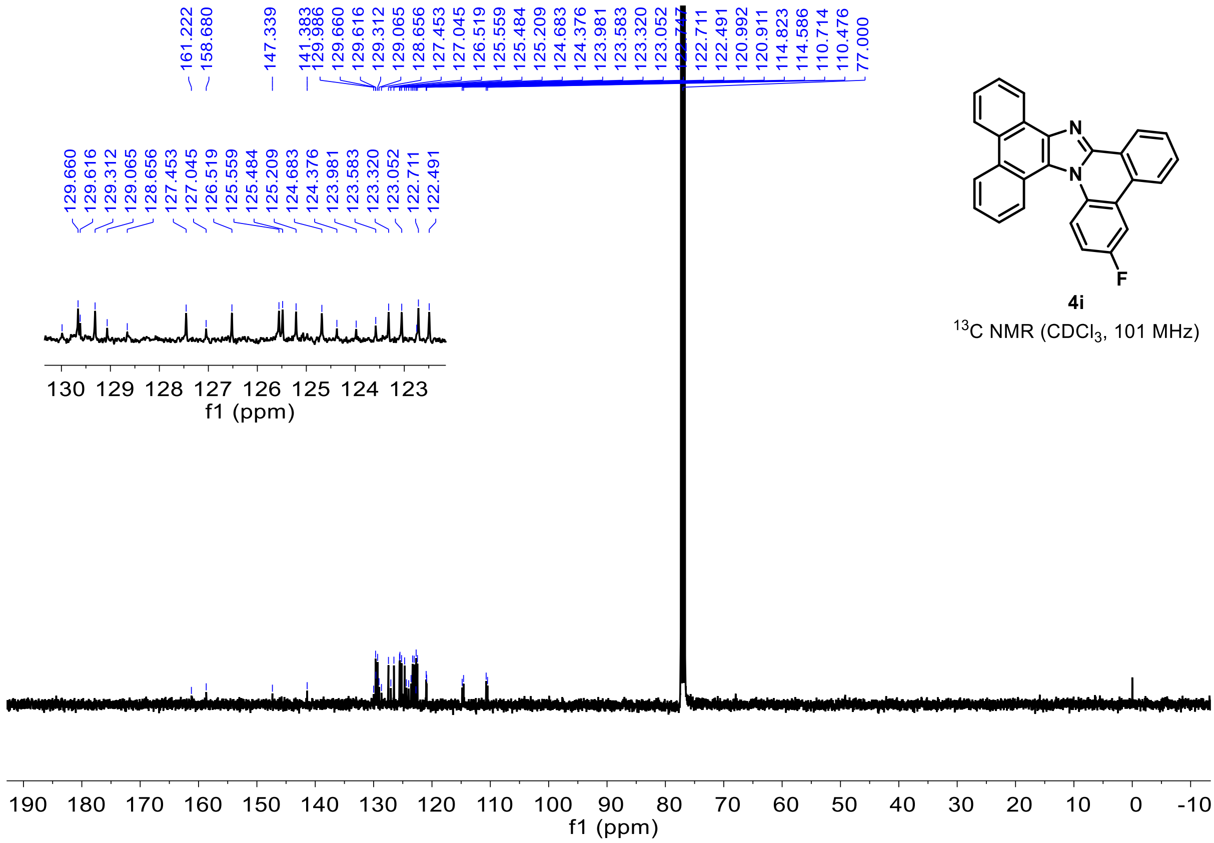

161.222
158.680
147.339
141.383
129.986
129.660
129.616
129.312
129.065
128.656
127.453
127.045
126.519
125.559
125.484
125.209
124.683
124.376
123.981
123.583
123.320
123.052
122.711
122.491
120.992
120.911
114.823
114.586
110.714
110.476
77.000
129.660
129.616
129.312
129.065
128.656
127.453
127.045
126.519
125.559
125.484
125.209
124.683
124.376
123.981
123.583
123.320
123.052
122.711
122.491
130 129 128 127 126 125 124 123
f1 (ppm)
N
N
F
4i
^{13}C NMR (CDCl$_3$, 101 MHz)
190 180 170 160 150 140 130 120 110 100 90 80 70 60 50 40 30 20 10 0 -10
f1 (ppm)

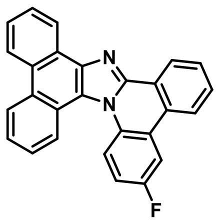

4i

^{19}F NMR ($CDCl_3$, 376 MHz)

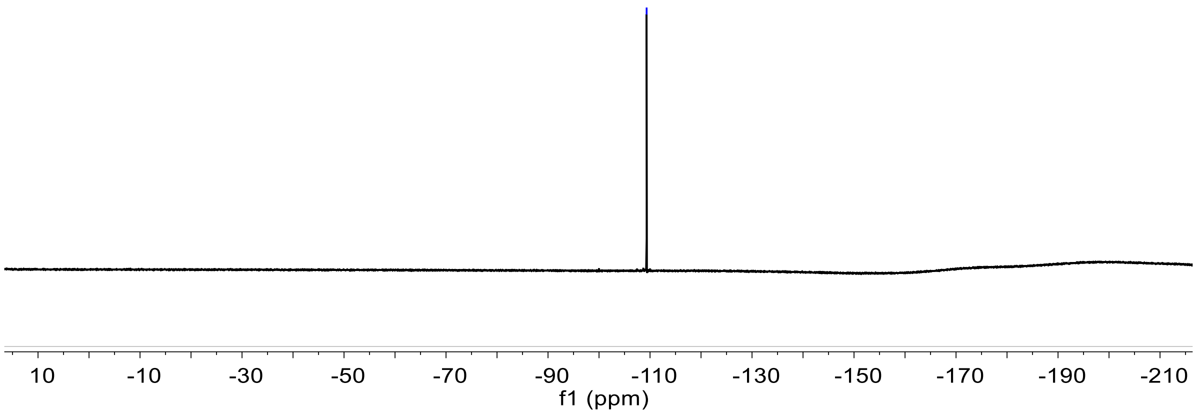

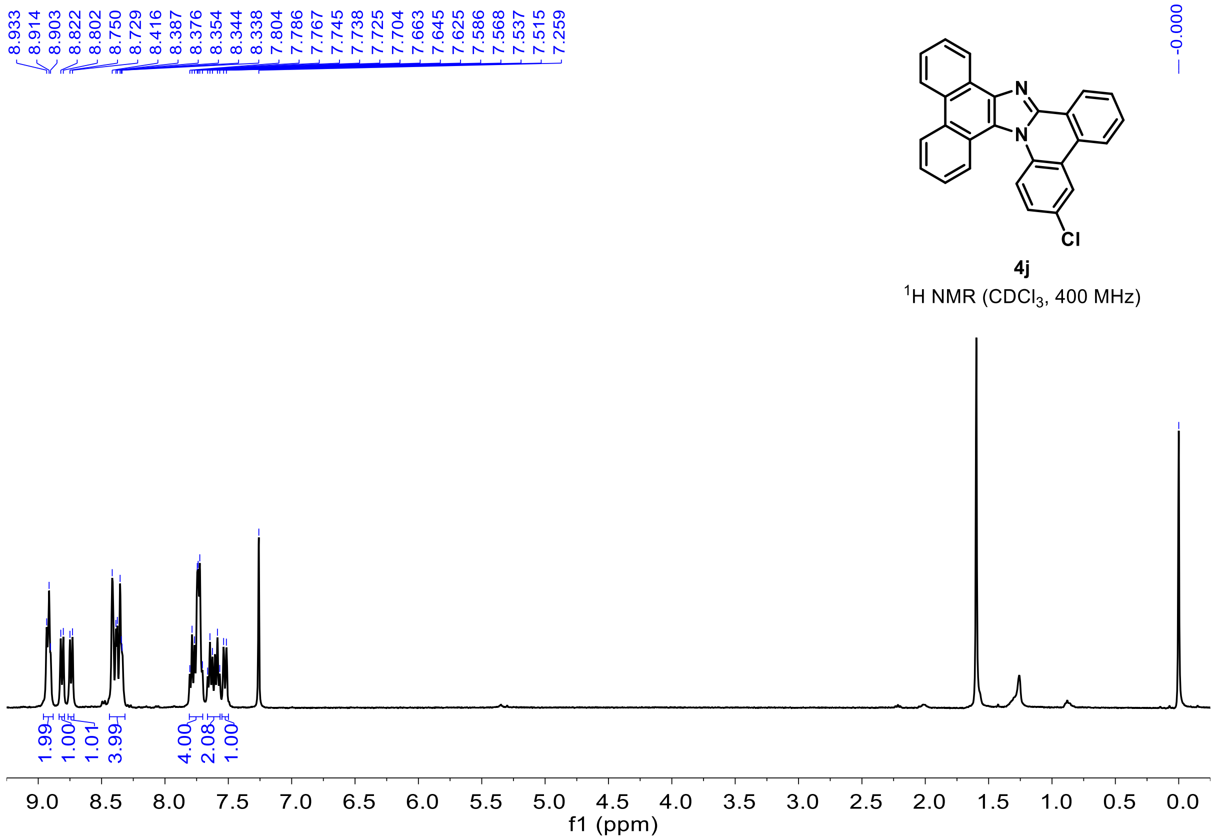

4j

^{1}H NMR ($CDCl_3$, 400 MHz)

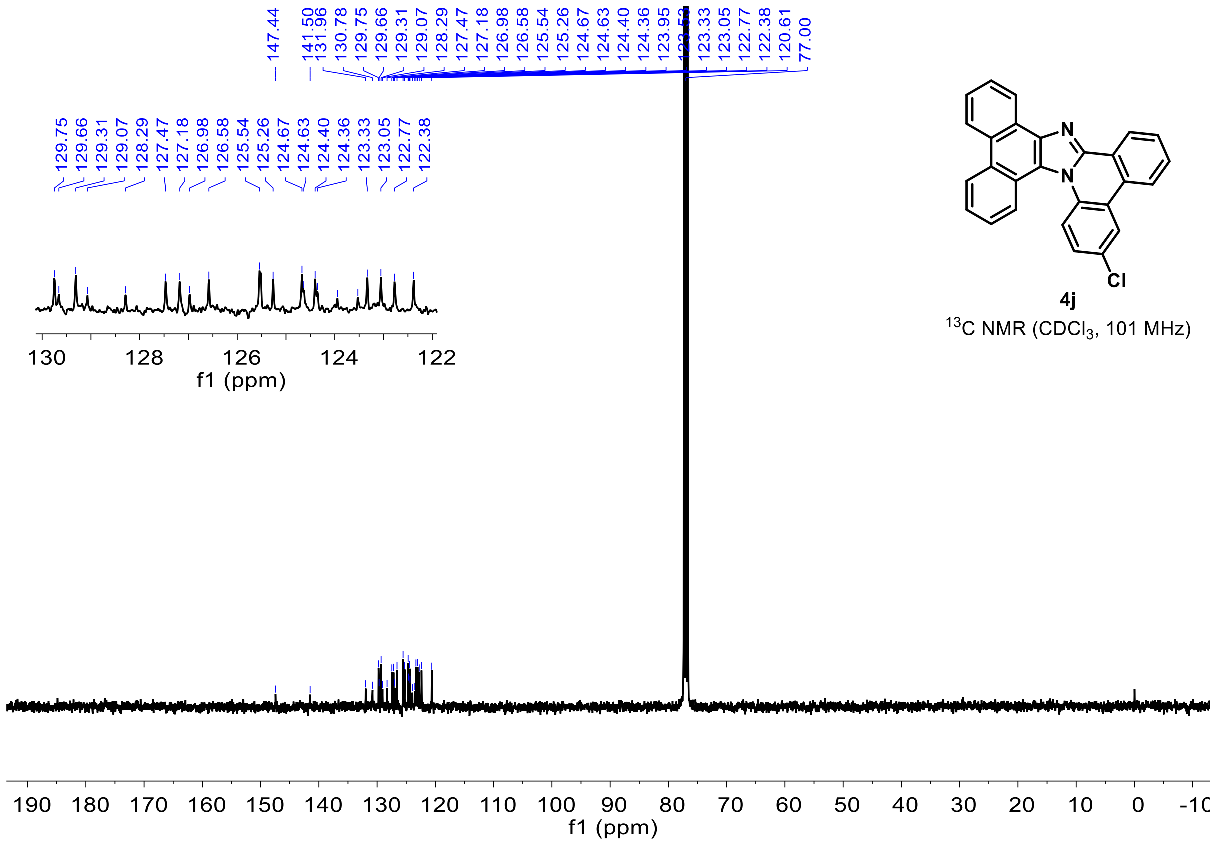

4j

^{13}C NMR ($CDCl_3$, 101 MHz)

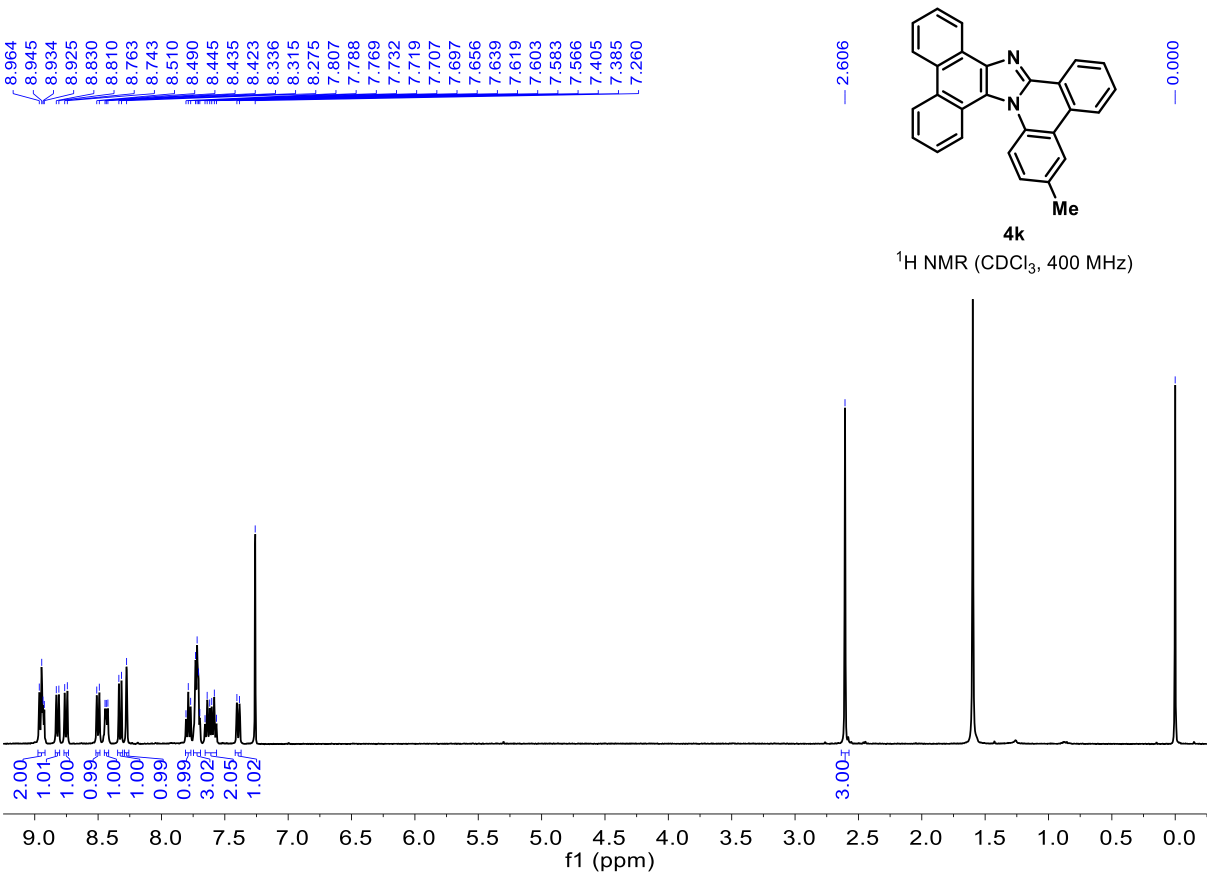
8.964 8.945 8.934 8.925 8.830 8.810 8.763 8.743 8.510 8.490 8.445 8.435 8.423 8.336 8.315 8.275 7.807 7.788 7.769 7.732 7.719 7.707 7.697 7.656 7.639 7.619 7.603 7.583 7.566 7.405 7.385 7.260
2.606
0.000
N
N
Me
4k
1H NMR (CDCl3, 400 MHz)
2.00 1.01 1.00 0.99 1.00 1.00 0.99 0.99 3.02 2.05 1.02
3.00
9.0 8.5 8.0 7.5 7.0 6.5 6.0 5.5 5.0 4.5 4.0 3.5 3.0 2.5 2.0 1.5 1.0 0.5 0.0
f1 (ppm)

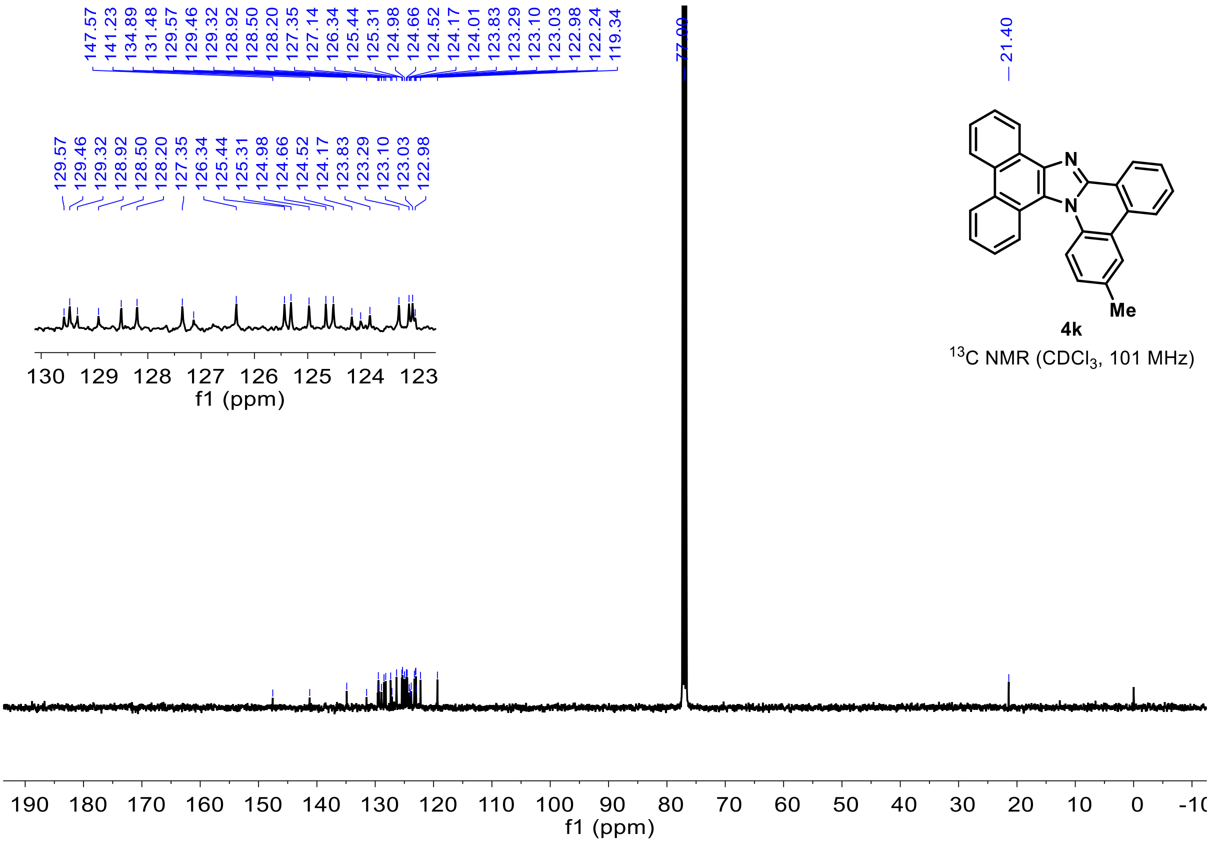
147.57 141.23 134.89 131.48 129.57 129.46 129.32 128.92 128.50 128.20 127.35 127.14 126.34 125.44 125.31 124.98 124.66 124.52 124.17 124.01 123.83 123.29 123.10 123.03 122.98 122.24 119.34
77.00
21.40
129.57 129.46 129.32 128.92 128.50 128.20 127.35 126.34 125.44 125.31 124.98 124.66 124.52 124.17 123.83 123.29 123.10 123.03 122.98
130 129 128 127 126 125 124 123
f1 (ppm)
N
N
Me
4k
13C NMR (CDCl3, 101 MHz)
190 180 170 160 150 140 130 120 110 100 90 80 70 60 50 40 30 20 10 0 -10
f1 (ppm)

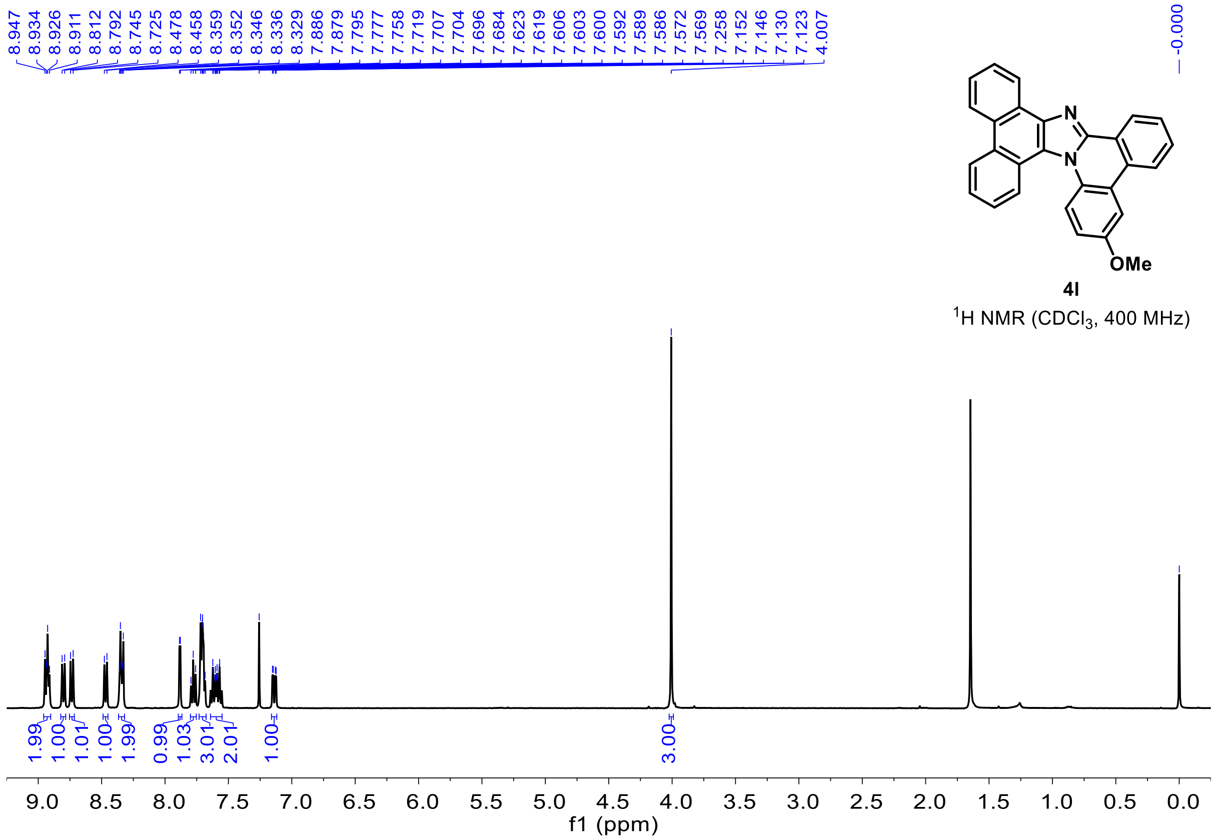

4l

^{1}H NMR ($CDCl_3$, 400 MHz)

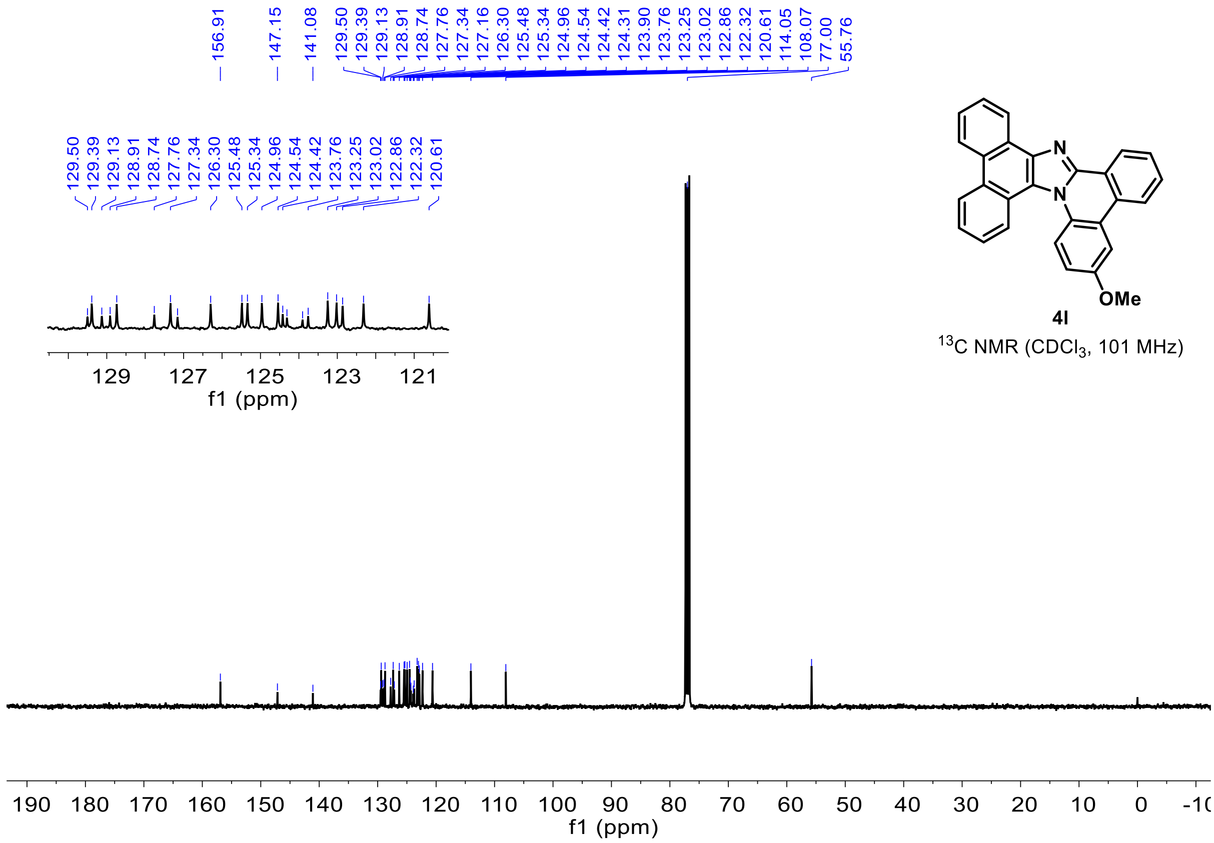

4l

^{13}C NMR ($CDCl_3$, 101 MHz)

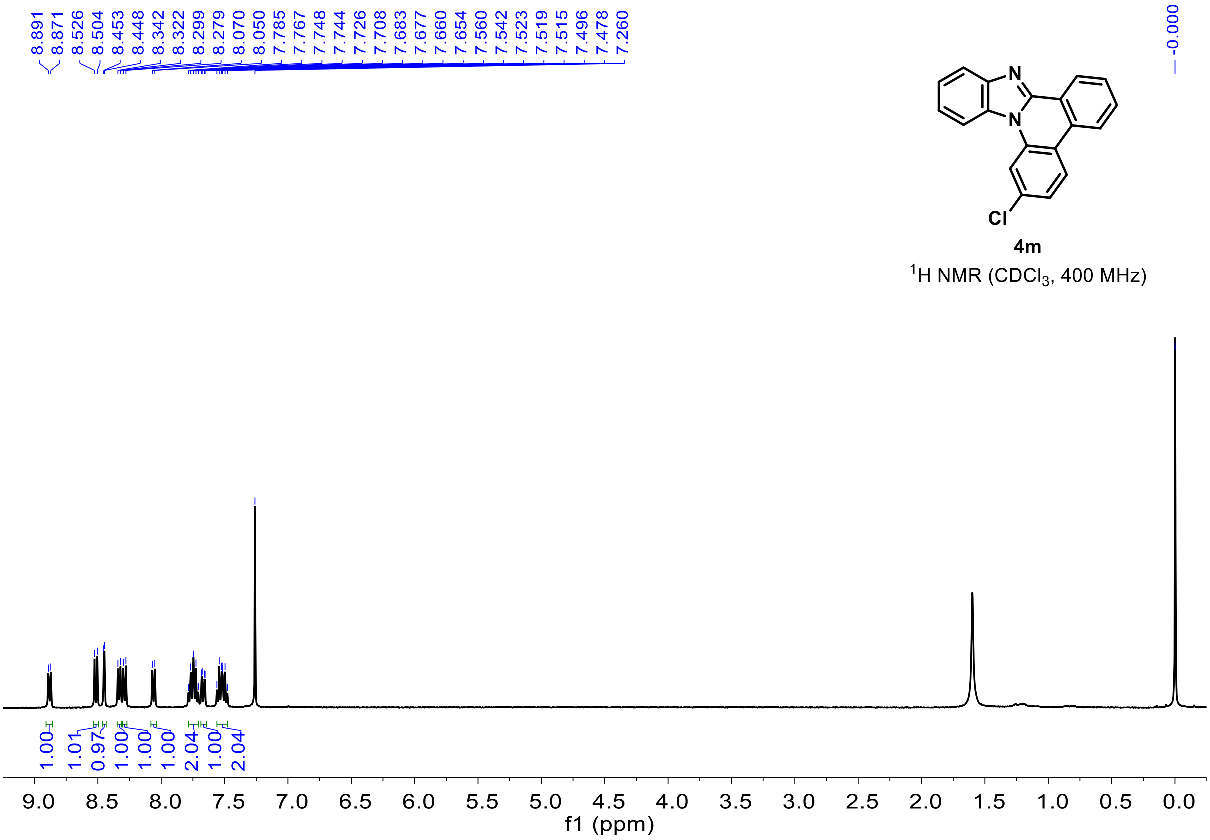
8.891
8.871
8.526
8.504
8.453
8.448
8.342
8.322
8.299
8.279
8.070
8.050
7.785
7.767
7.748
7.744
7.726
7.708
7.683
7.677
7.660
7.654
7.560
7.542
7.523
7.519
7.515
7.496
7.478
7.260
-0.000
N
N
Cl
4m
1H NMR (CDCl3, 400 MHz)
1.00
1.01
0.97
1.00
1.00
1.00
2.04
1.00
2.04
9.0 8.5 8.0 7.5 7.0 6.5 6.0 5.5 5.0 4.5 4.0 3.5 3.0 2.5 2.0 1.5 1.0 0.5 0.0
f1 (ppm)

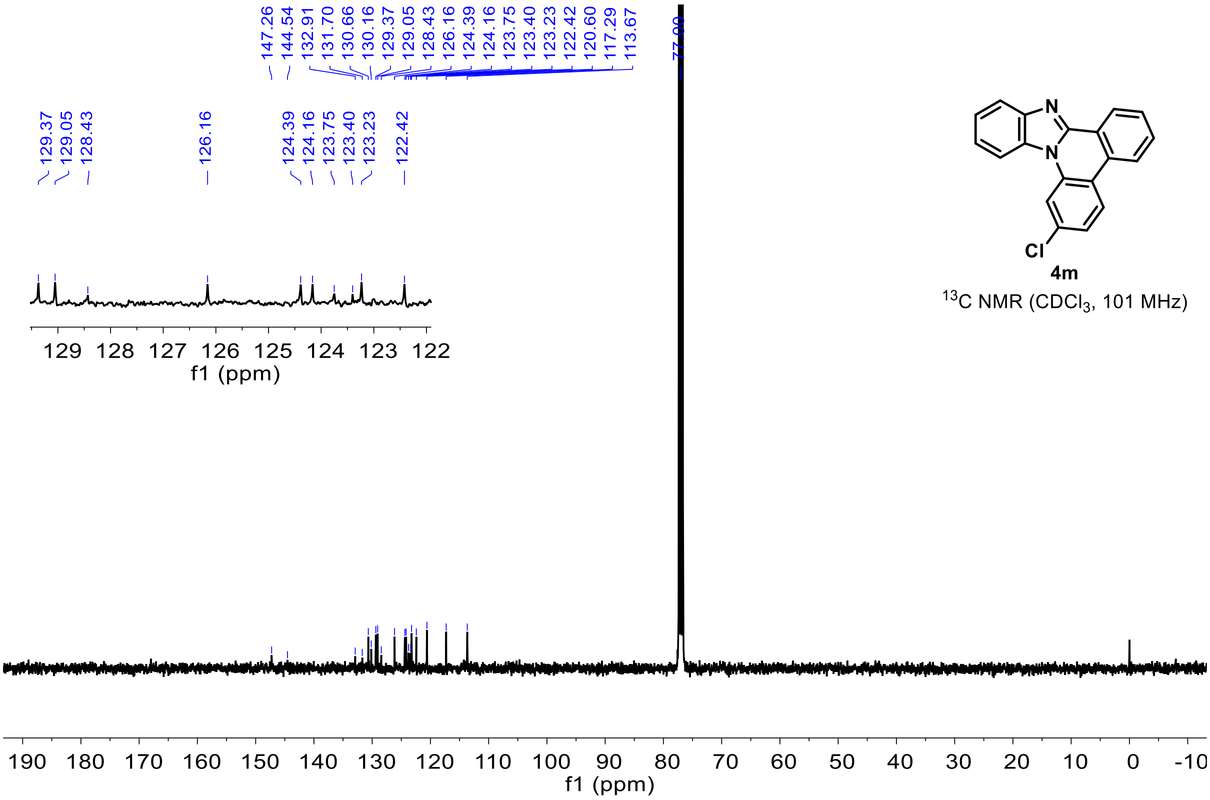
147.26
144.54
132.91
131.70
130.66
130.16
129.37
129.05
128.43
126.16
124.39
124.16
123.75
123.40
123.23
122.42
120.60
117.29
113.67
77.00
129.37
129.05
128.43
126.16
124.39
124.16
123.75
123.40
123.23
122.42
129 128 127 126 125 124 123 122
f1 (ppm)
N
N
Cl
4m
13C NMR (CDCl3, 101 MHz)
190 180 170 160 150 140 130 120 110 100 90 80 70 60 50 40 30 20 10 0 -10
f1 (ppm)

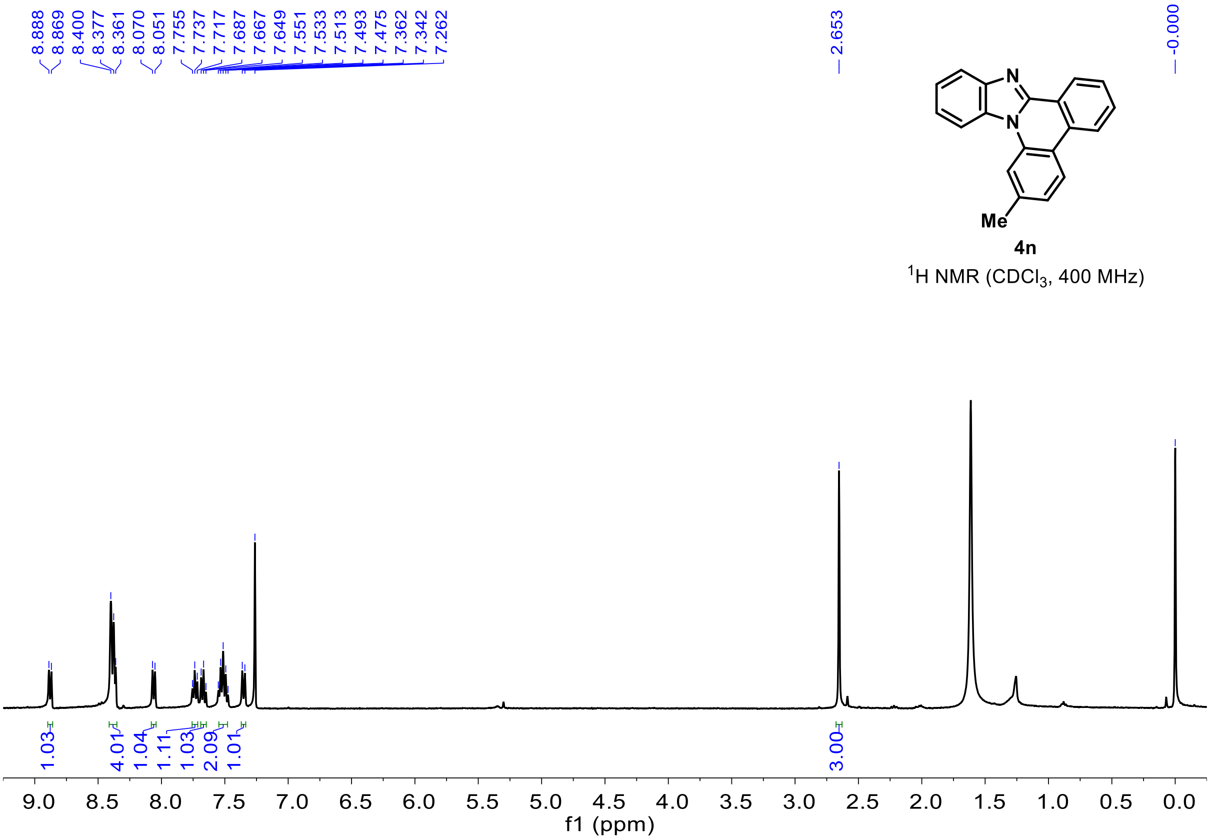
4n
^{1}H NMR (CDCl$_3$, 400 MHz)
8.888
8.869
8.400
8.377
8.361
8.070
8.051
7.755
7.737
7.717
7.687
7.667
7.649
7.551
7.533
7.513
7.493
7.475
7.362
7.342
7.262
2.653
-0.000
1.03
4.01
1.04
1.11
1.03
2.09
1.01
3.00
f1 (ppm)

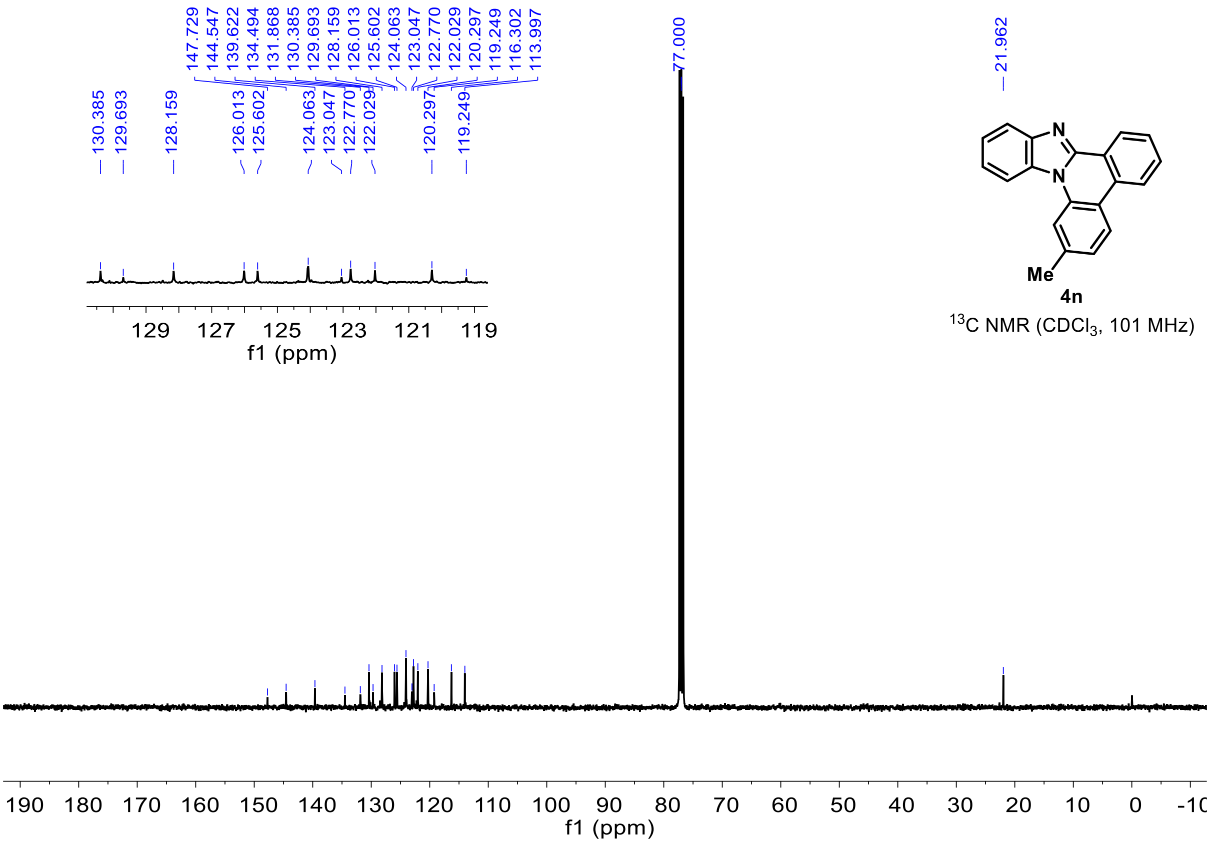
4n
^{13}C NMR (CDCl$_3$, 101 MHz)
147.729
144.547
139.622
134.494
131.868
130.385
129.693
128.159
126.013
125.602
124.063
123.047
122.770
122.029
120.297
119.249
116.302
113.997
77.000
21.962
f1 (ppm)

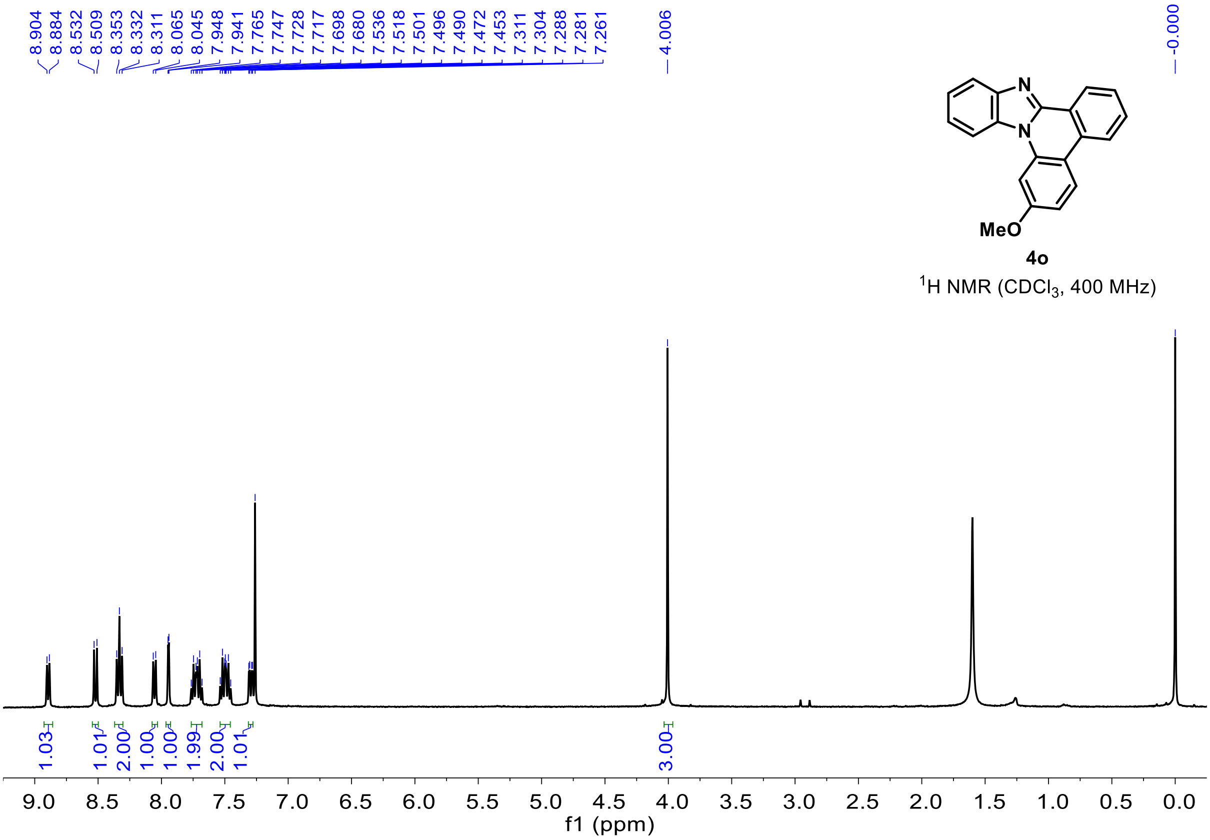
8.904
8.884
8.532
8.509
8.353
8.332
8.311
8.065
8.045
7.948
7.941
7.765
7.747
7.728
7.717
7.698
7.680
7.536
7.518
7.501
7.496
7.490
7.472
7.453
7.311
7.304
7.288
7.281
7.261
4.006
-0.000
N
N
MeO
4o
1H NMR (CDCl3, 400 MHz)
1.03
1.01
2.00
1.00
1.00
1.99
2.00
1.01
3.00
9.0 8.5 8.0 7.5 7.0 6.5 6.0 5.5 5.0 4.5 4.0 3.5 3.0 2.5 2.0 1.5 1.0 0.5 0.0
f1 (ppm)

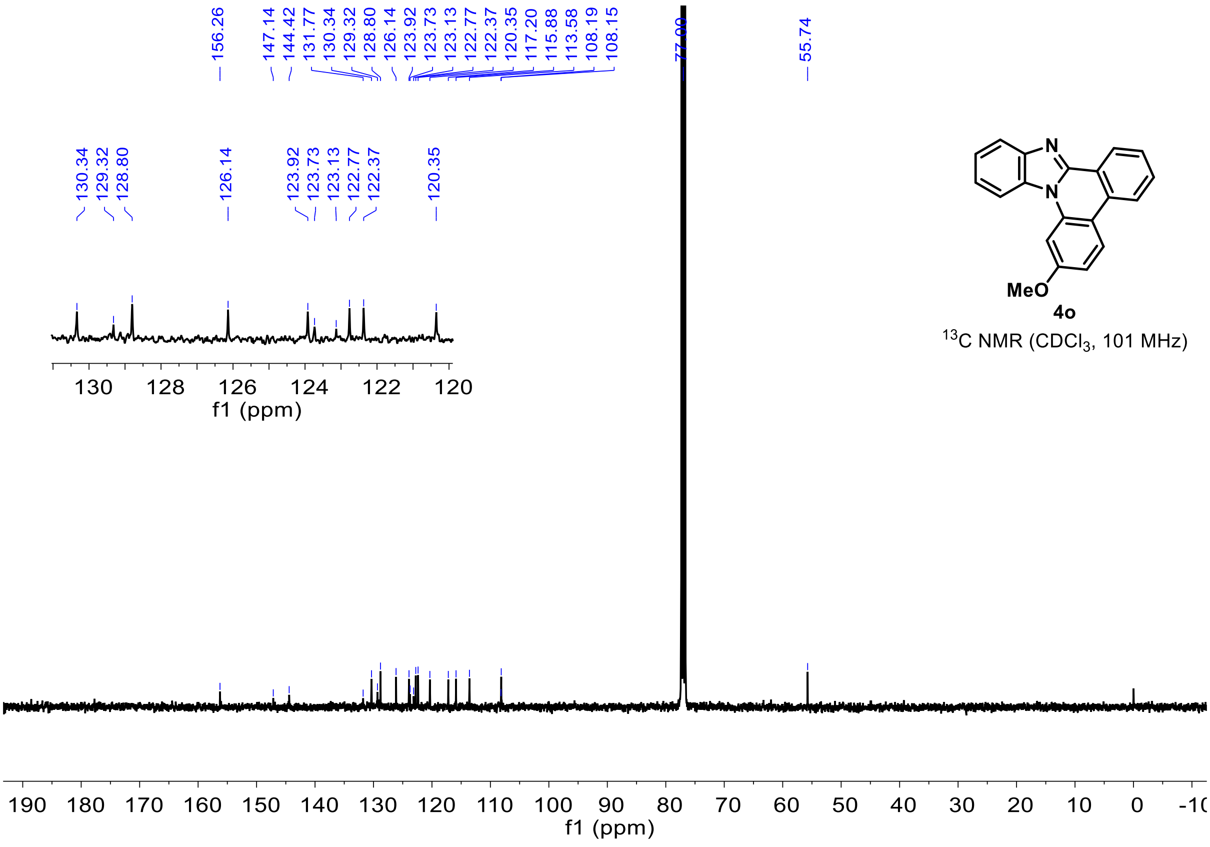
156.26
147.14
144.42
131.77
130.34
129.32
128.80
126.14
123.92
123.73
123.13
122.77
122.37
120.35
117.20
115.88
113.58
108.19
108.15
77.00
55.74
130.34
129.32
128.80
126.14
123.92
123.73
123.13
122.77
122.37
120.35
130 128 126 124 122 120
f1 (ppm)
N
N
MeO
4o
13C NMR (CDCl3, 101 MHz)
190 180 170 160 150 140 130 120 110 100 90 80 70 60 50 40 30 20 10 0 -10
f1 (ppm)

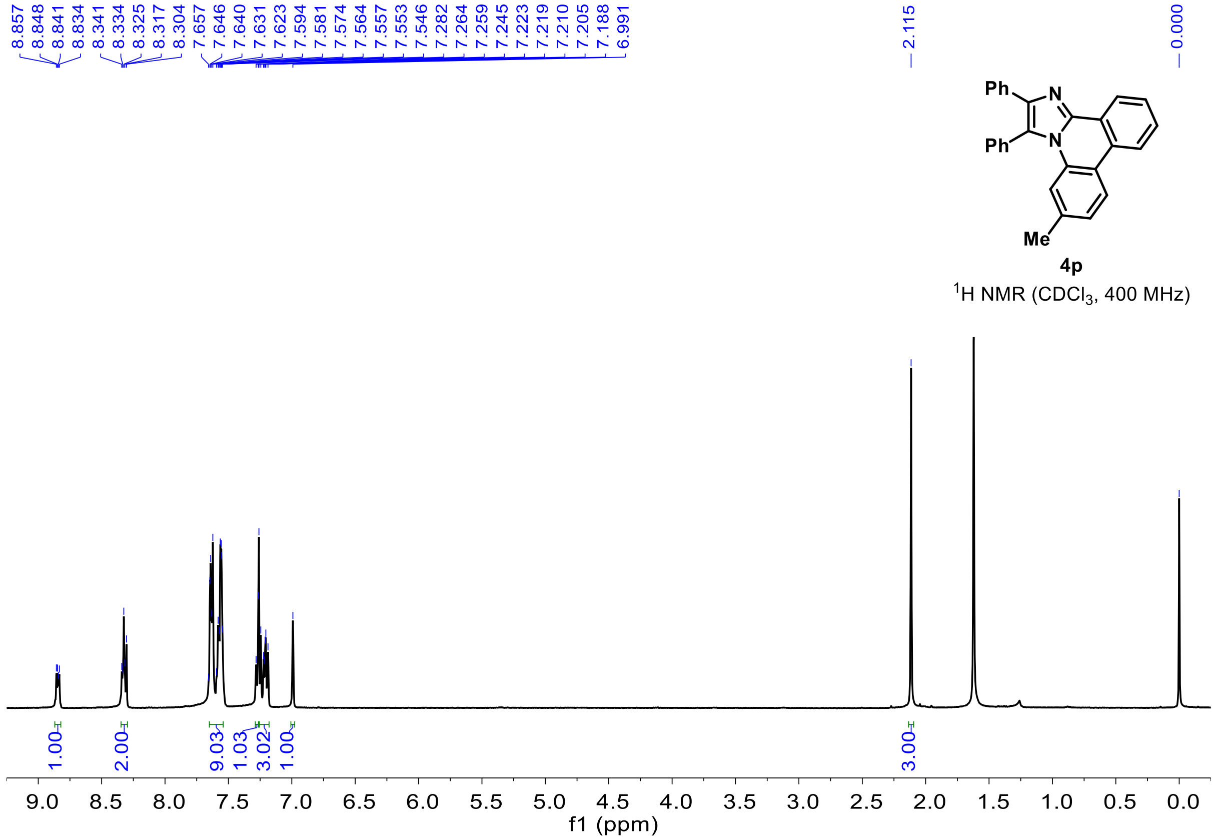
8.857 8.848 8.841 8.834 8.341 8.334 8.325 8.317 8.304 7.657 7.646 7.640 7.631 7.623 7.594 7.581 7.574 7.564 7.557 7.553 7.546 7.282 7.264 7.259 7.245 7.223 7.219 7.210 7.205 7.188 6.991
2.115
0.000
Ph
Ph
N
N
Me
4p
^{1}H NMR (CDCl$_3$, 400 MHz)
1.00 2.00 9.03 1.03 3.02 1.00 3.00
9.0 8.5 8.0 7.5 7.0 6.5 6.0 5.5 5.0 4.5 4.0 3.5 3.0 2.5 2.0 1.5 1.0 0.5 0.0
f1 (ppm)

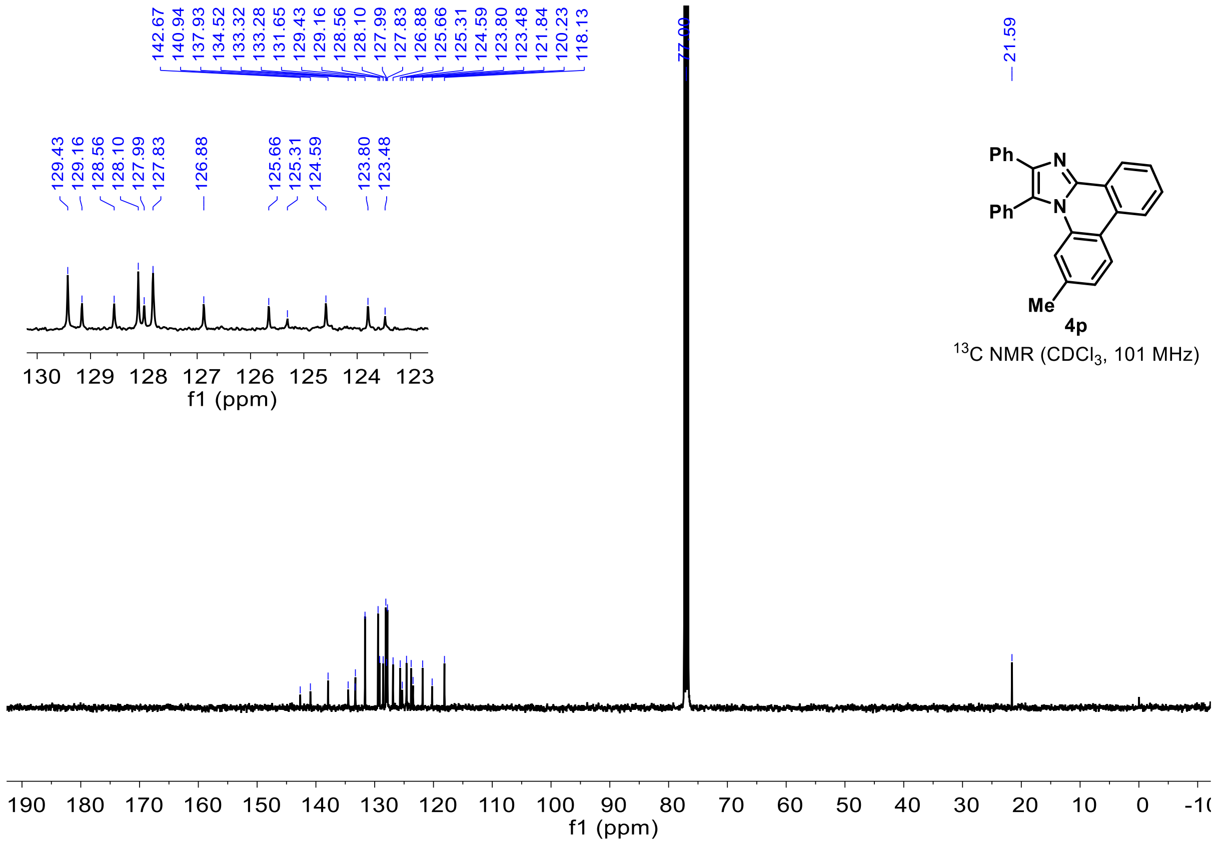
142.67 140.94 137.93 134.52 133.32 133.28 131.65 129.43 129.16 128.56 128.10 127.99 127.83 126.88 125.66 125.31 124.59 123.80 123.48 121.84 120.23 118.13
77.00
21.59
129.43 129.16 128.56 128.10 127.99 127.83 126.88 125.66 125.31 124.59 123.80 123.48
130 129 128 127 126 125 124 123
f1 (ppm)
Ph
Ph
N
N
Me
4p
^{13}C NMR (CDCl$_3$, 101 MHz)
190 180 170 160 150 140 130 120 110 100 90 80 70 60 50 40 30 20 10 0 -10
f1 (ppm)

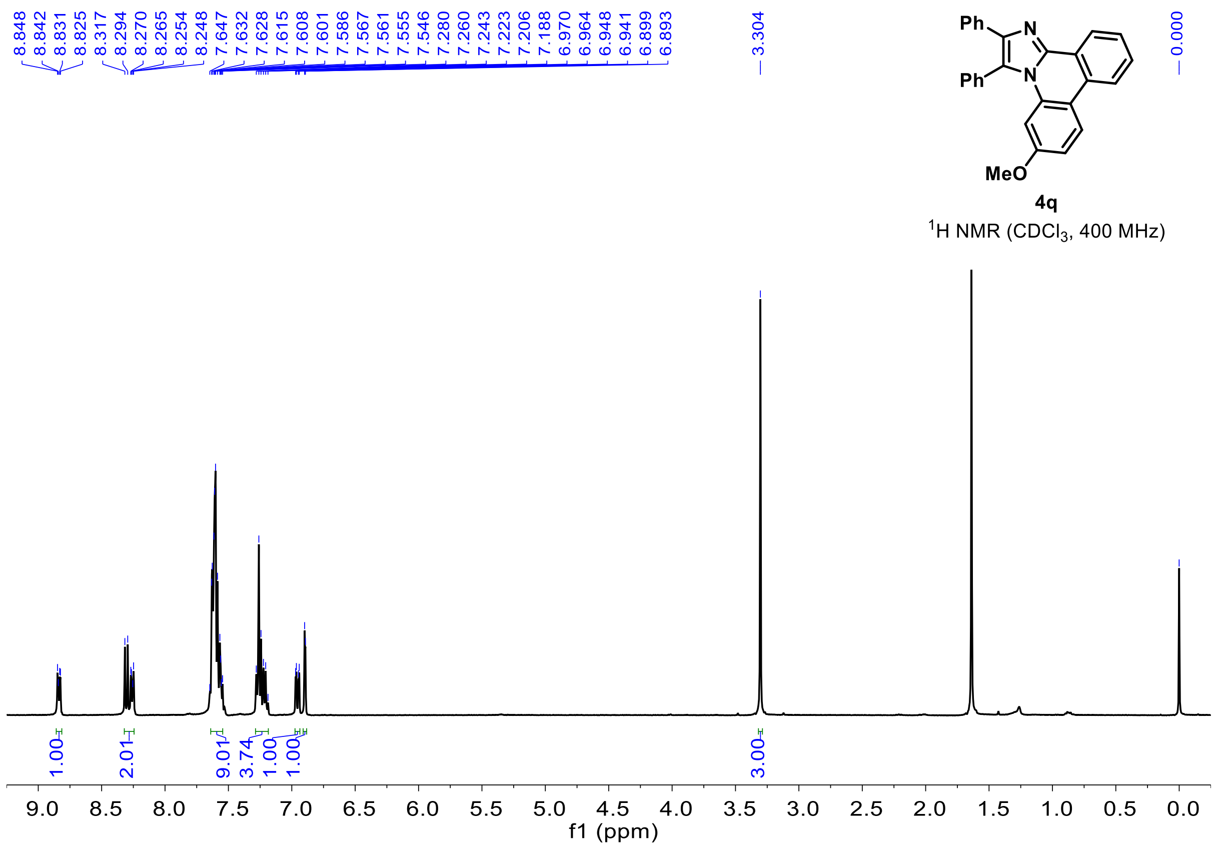
Ph
N
Ph
N
MeO
4q
^{1}H NMR (CDCl$_3$, 400 MHz)
f1 (ppm)

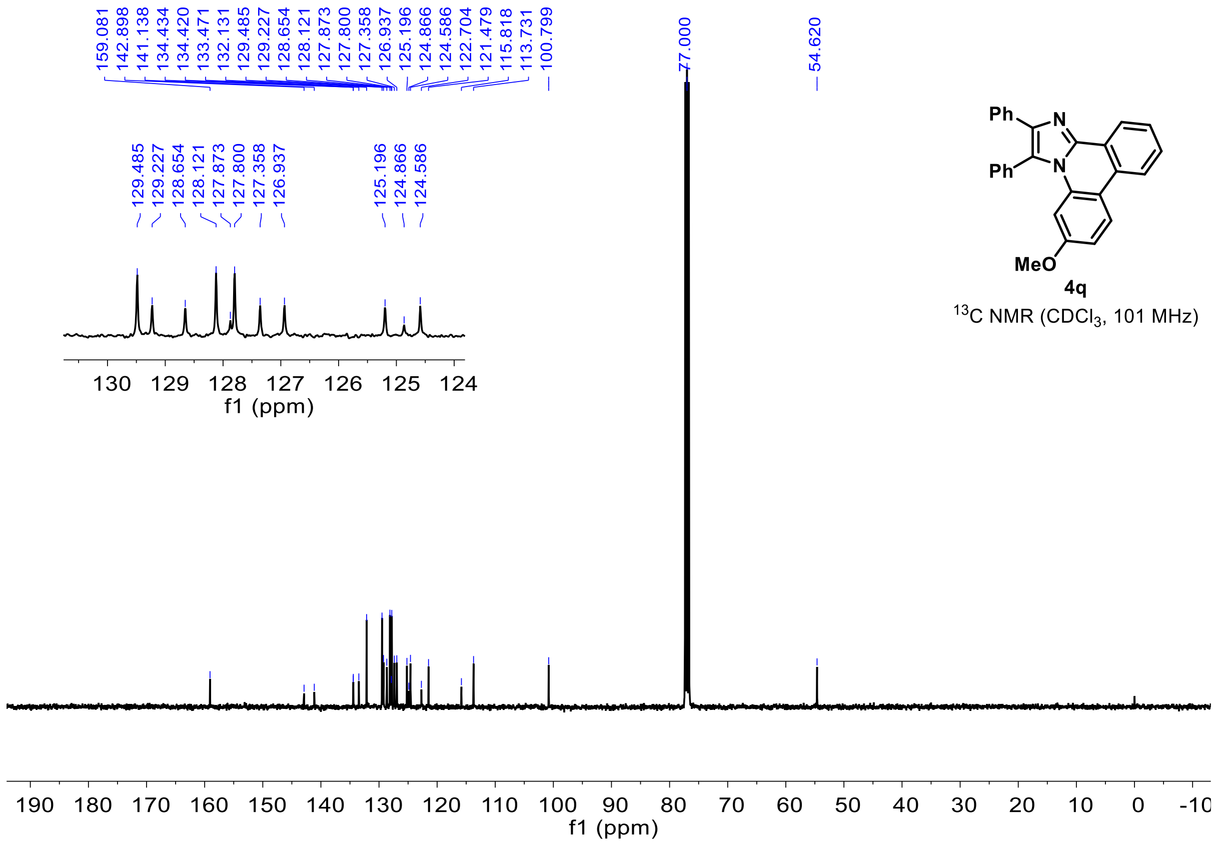
Ph
N
Ph
N
MeO
4q
^{13}C NMR (CDCl$_3$, 101 MHz)
f1 (ppm)

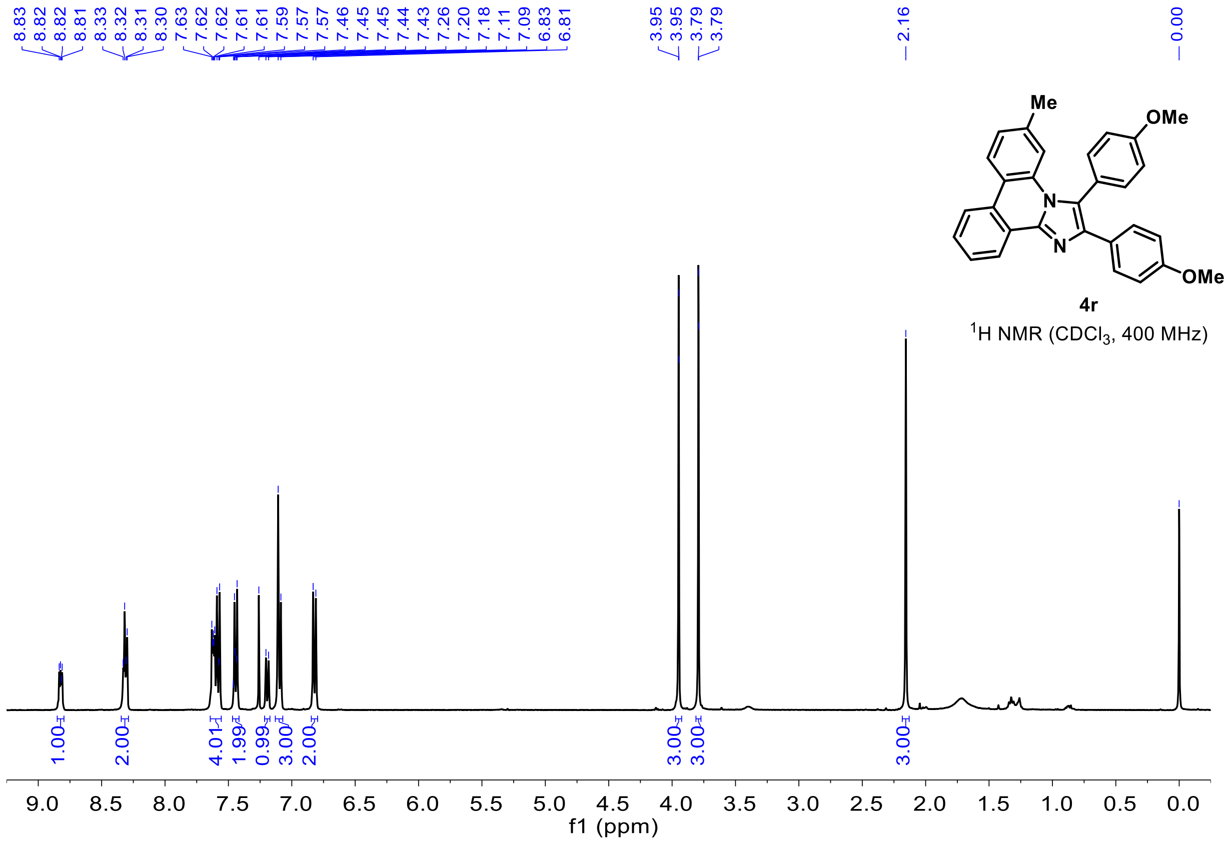

4r

^1H NMR ($CDCl_3$, 400 MHz)

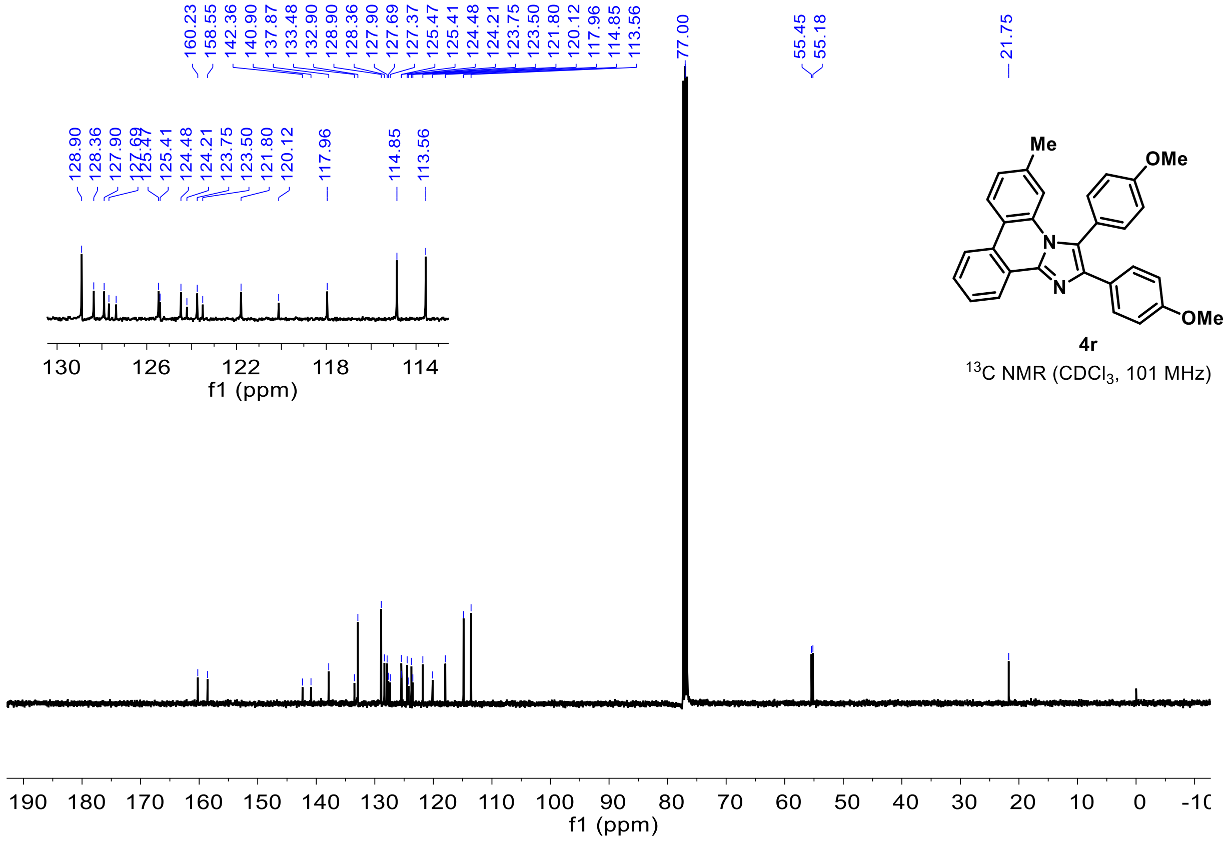

4r

^{13}C NMR ($CDCl_3$, 101 MHz)

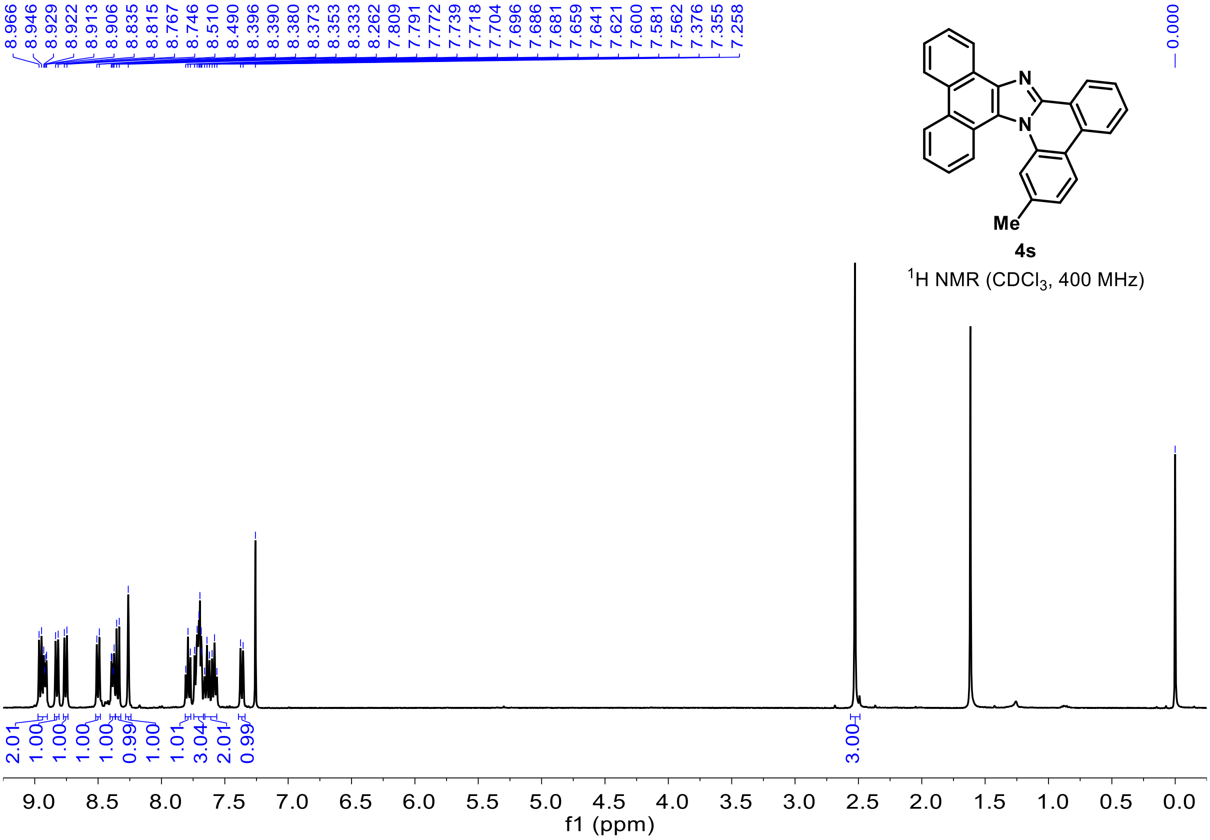
8.966 8.946 8.929 8.922 8.913 8.906 8.835 8.815 8.767 8.746 8.510 8.490 8.396 8.390 8.380 8.373 8.353 8.333 8.262 7.809 7.791 7.772 7.739 7.718 7.704 7.696 7.686 7.681 7.659 7.641 7.621 7.600 7.581 7.562 7.376 7.355 7.258
0.000
N
N
Me
4s
1H NMR (CDCl3, 400 MHz)
2.01 1.00 1.00 1.00 1.00 0.99 1.00 1.01 3.04 2.01 0.99
3.00
f1 (ppm)

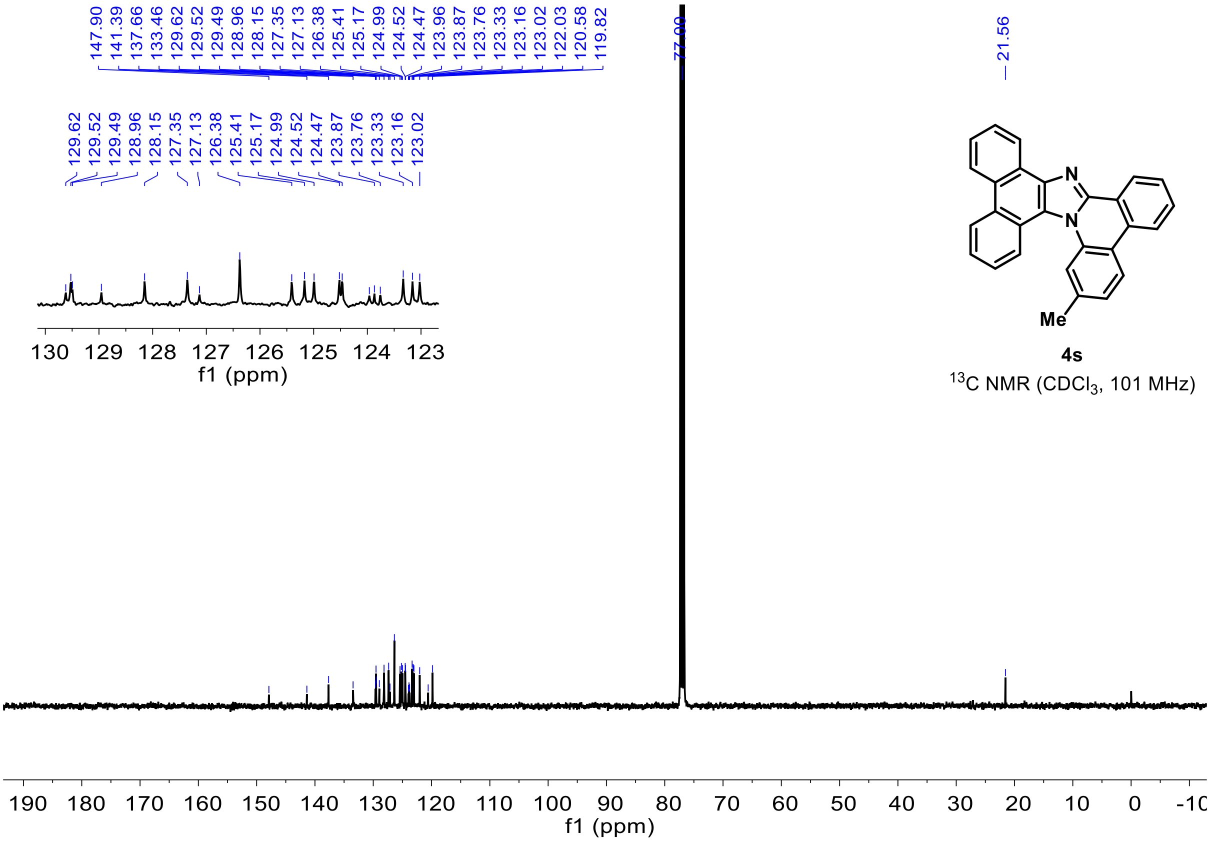
147.90 141.39 137.66 133.46 129.62 129.52 129.49 128.96 128.15 127.35 127.13 126.38 125.41 125.17 124.99 124.52 124.47 123.96 123.87 123.76 123.33 123.16 123.02 122.03 120.58 119.82
77.00
21.56
129.62 129.52 129.49 128.96 128.15 127.35 127.13 126.38 125.41 125.17 124.99 124.52 124.47 123.87 123.76 123.33 123.16 123.02
f1 (ppm)
N
N
Me
4s
13C NMR (CDCl3, 101 MHz)
f1 (ppm)

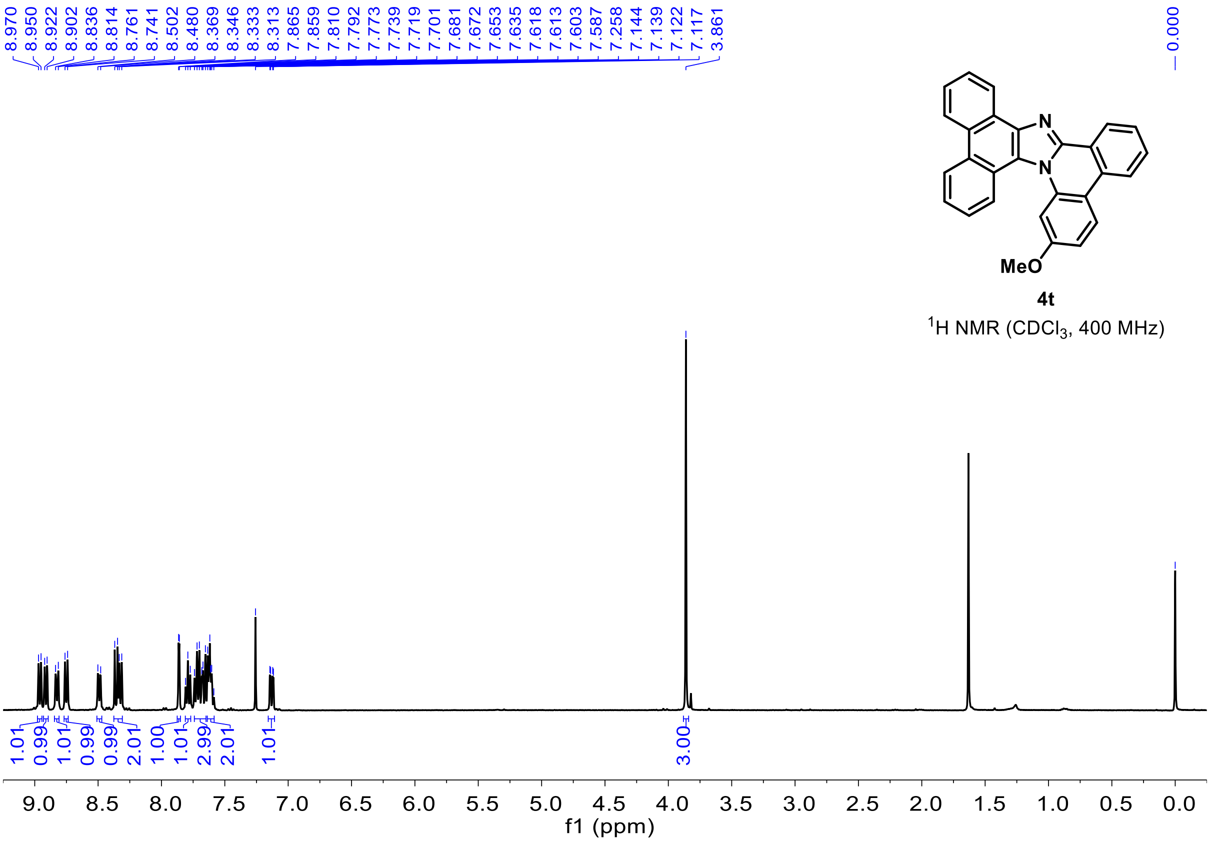
8.970
8.950
8.922
8.902
8.836
8.814
8.761
8.741
8.502
8.480
8.369
8.346
8.333
8.313
7.865
7.859
7.810
7.792
7.773
7.739
7.719
7.701
7.681
7.672
7.653
7.635
7.618
7.613
7.603
7.587
7.258
7.144
7.139
7.122
7.117
3.861
0.000
MeO
N
N
4t
^{1}H NMR ($CDCl_3$, 400 MHz)
1.01
0.99
1.01
0.99
0.99
2.01
1.00
1.01
2.99
2.01
1.01
3.00
9.0 8.5 8.0 7.5 7.0 6.5 6.0 5.5 5.0 4.5 4.0 3.5 3.0 2.5 2.0 1.5 1.0 0.5 0.0
f1 (ppm)

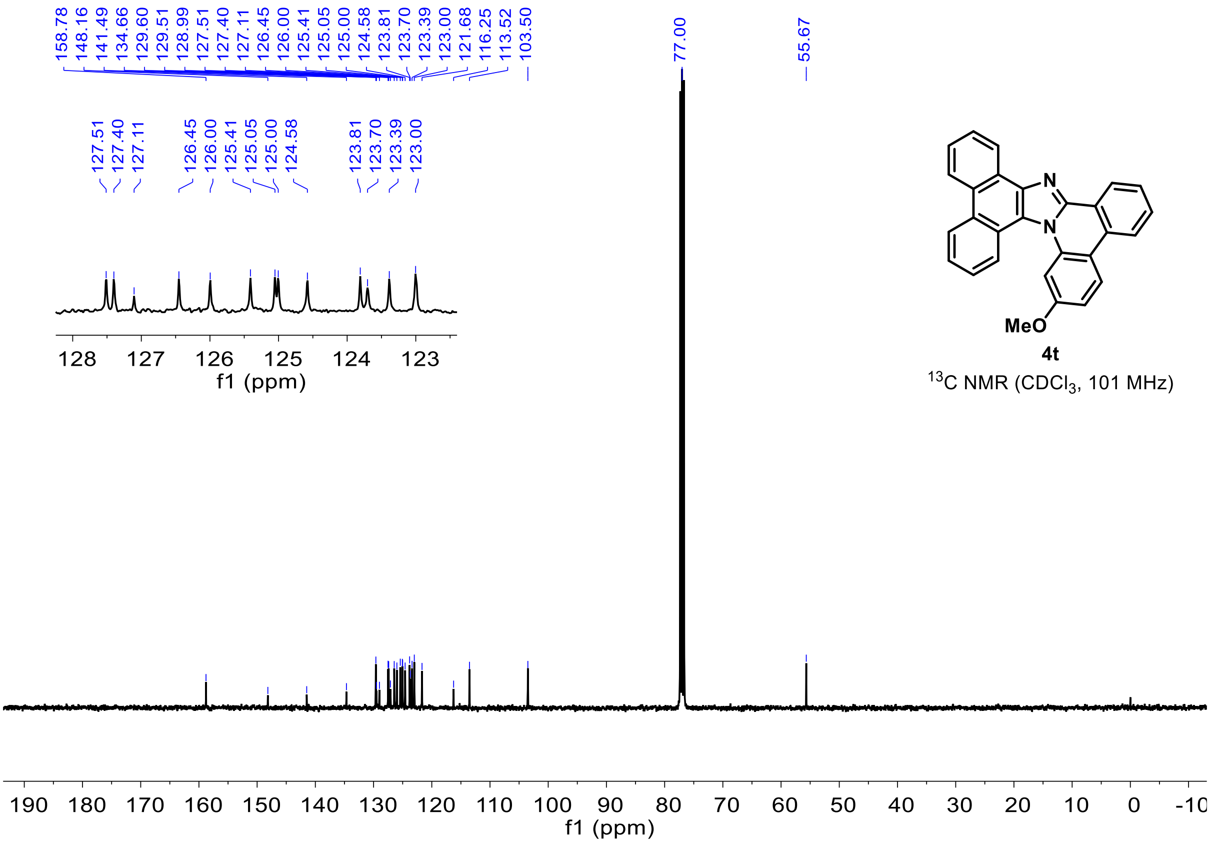
158.78
148.16
141.49
134.66
129.60
129.51
128.99
127.51
127.40
127.11
126.45
126.00
125.41
125.05
125.00
124.58
123.81
123.70
123.39
123.00
121.68
116.25
113.52
103.50
77.00
55.67
127.51
127.40
127.11
126.45
126.00
125.41
125.05
125.00
124.58
123.81
123.70
123.39
123.00
128 127 126 125 124 123
f1 (ppm)
MeO
N
N
4t
^{13}C NMR ($CDCl_3$, 101 MHz)
190 180 170 160 150 140 130 120 110 100 90 80 70 60 50 40 30 20 10 0 -10
f1 (ppm)

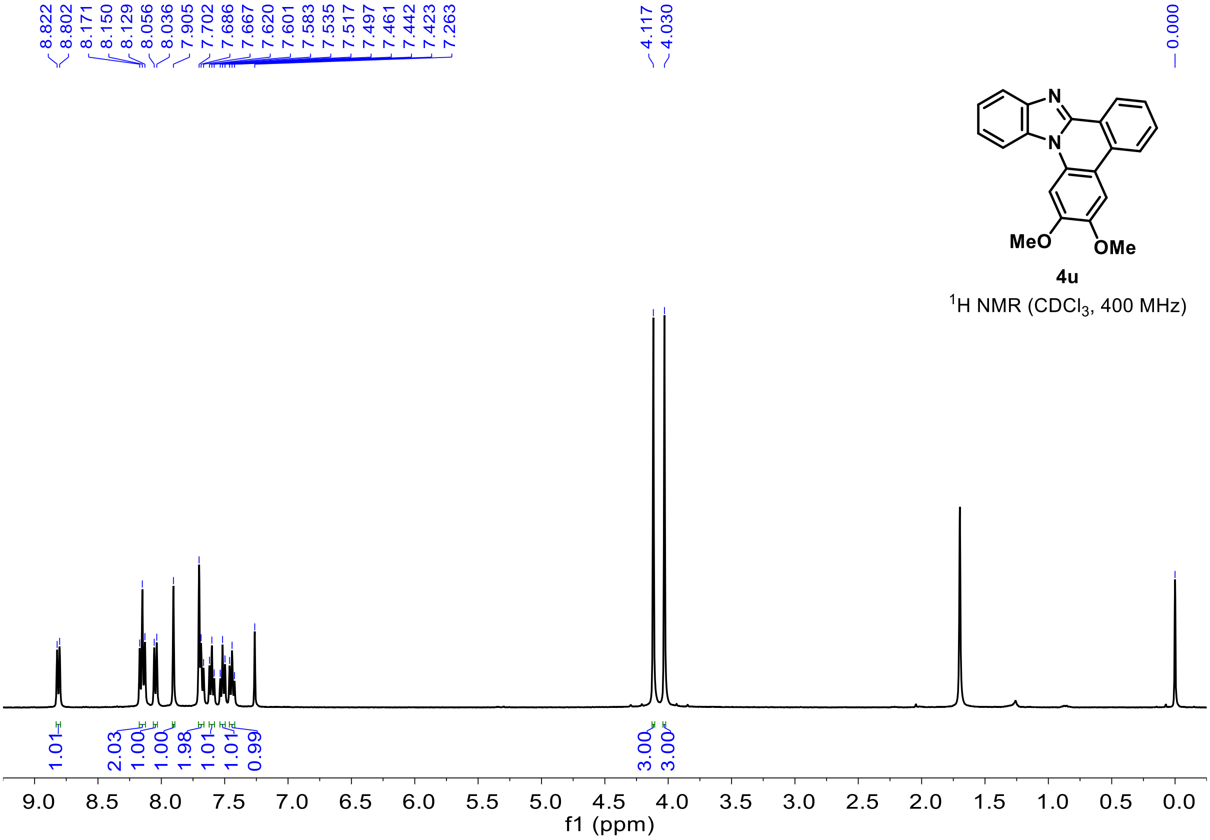
8.822
8.802
8.171
8.150
8.129
8.056
8.036
7.905
7.702
7.686
7.667
7.620
7.601
7.583
7.535
7.517
7.497
7.461
7.442
7.423
7.263
4.117
4.030
0.000
N
N
MeO
OMe
4u
^{1}H NMR ($CDCl_3$, 400 MHz)
1.01
2.03
1.00
1.00
1.98
1.01
1.01
0.99
3.00
3.00
9.0 8.5 8.0 7.5 7.0 6.5 6.0 5.5 5.0 4.5 4.0 3.5 3.0 2.5 2.0 1.5 1.0 0.5 0.0
f1 (ppm)

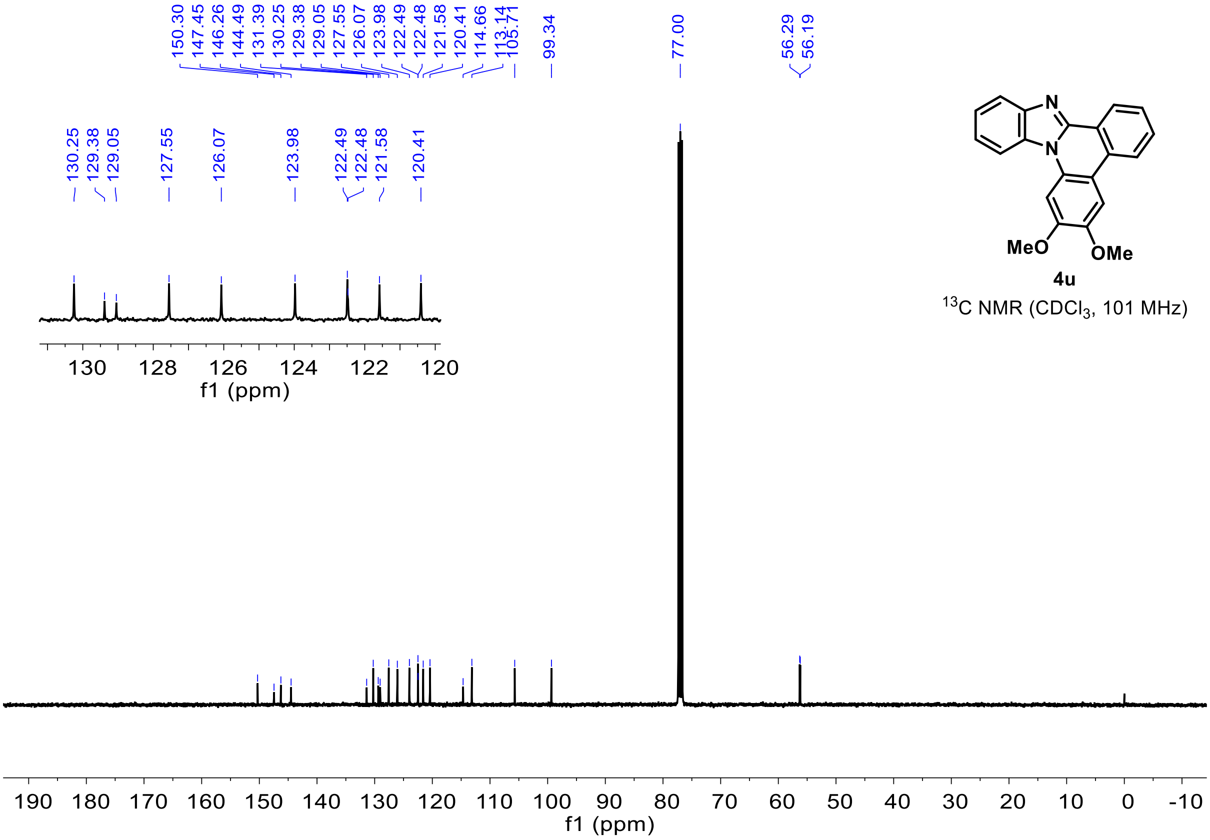
150.30
147.45
146.26
144.49
131.39
130.25
129.38
129.05
127.55
126.07
123.98
122.49
122.48
121.58
120.41
114.66
113.14
105.71
99.34
77.00
56.29
56.19
130.25
129.38
129.05
127.55
126.07
123.98
122.49
122.48
121.58
120.41
130 128 126 124 122 120
f1 (ppm)
N
N
MeO
OMe
4u
^{13}C NMR ($CDCl_3$, 101 MHz)
190 180 170 160 150 140 130 120 110 100 90 80 70 60 50 40 30 20 10 0 -10
f1 (ppm)

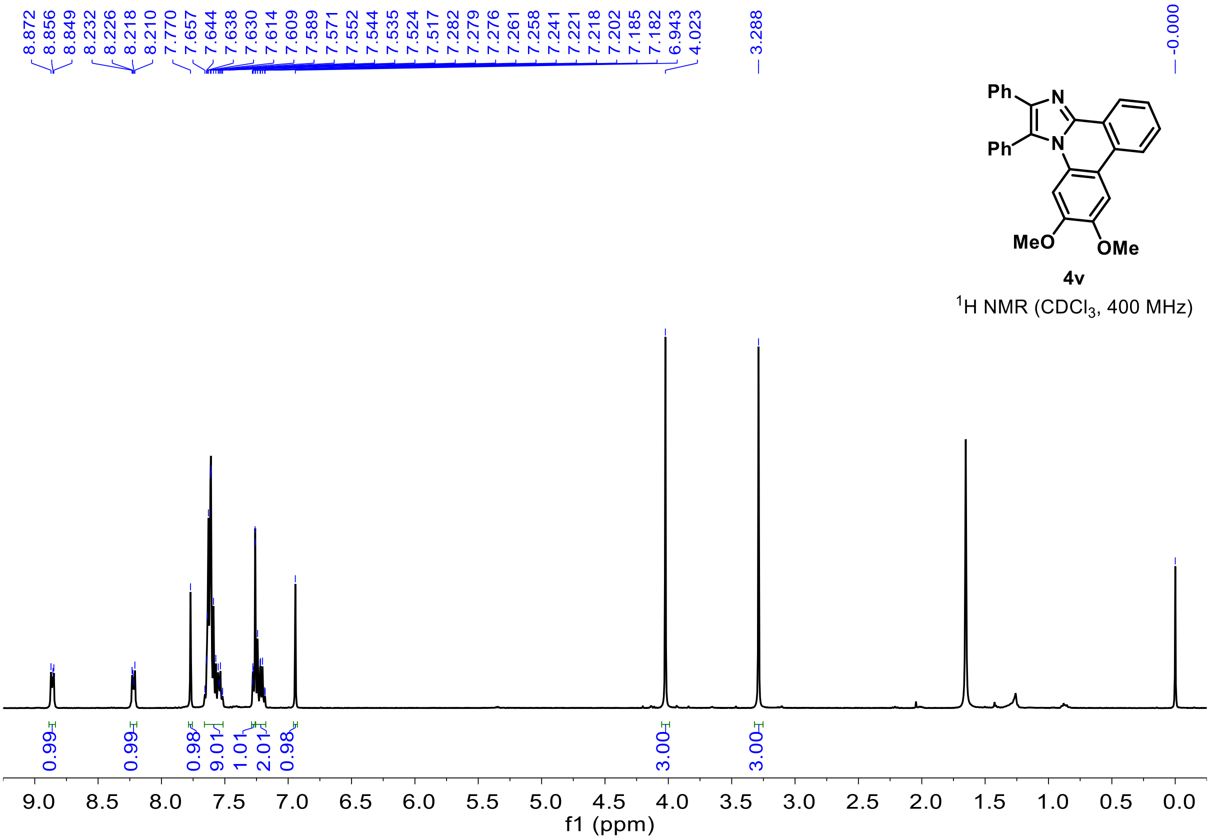

4v

^{1}H NMR (CDCl$_3$, 400 MHz)

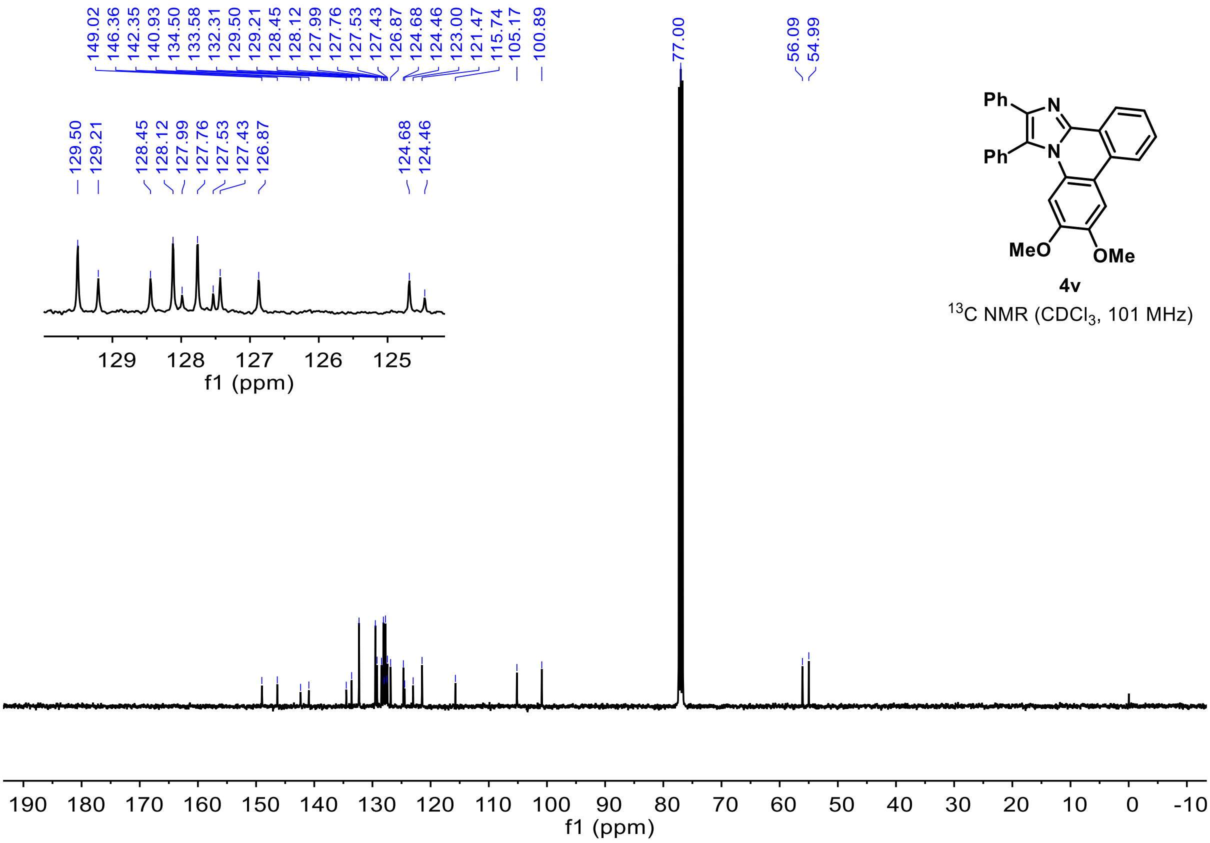

4v

^{13}C NMR (CDCl$_3$, 101 MHz)

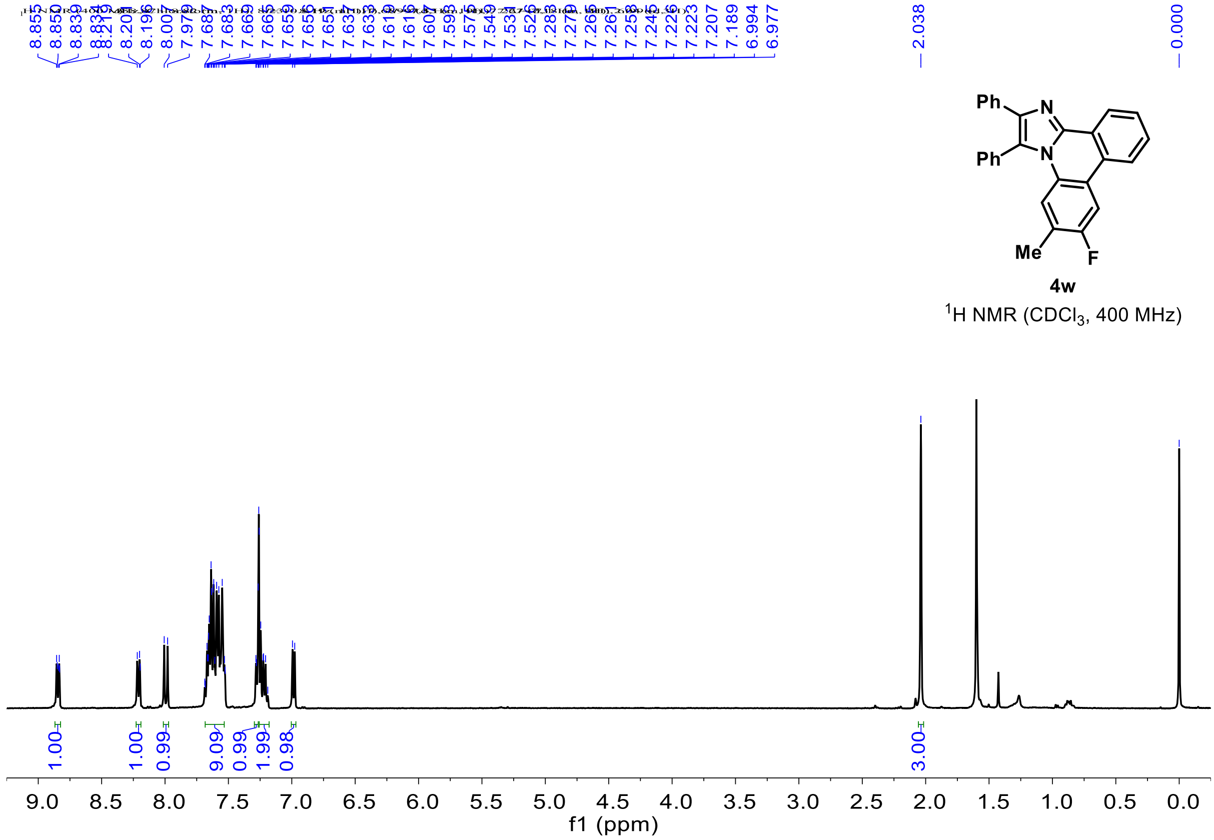

4w

^{1}H NMR ($CDCl_3$, 400 MHz)

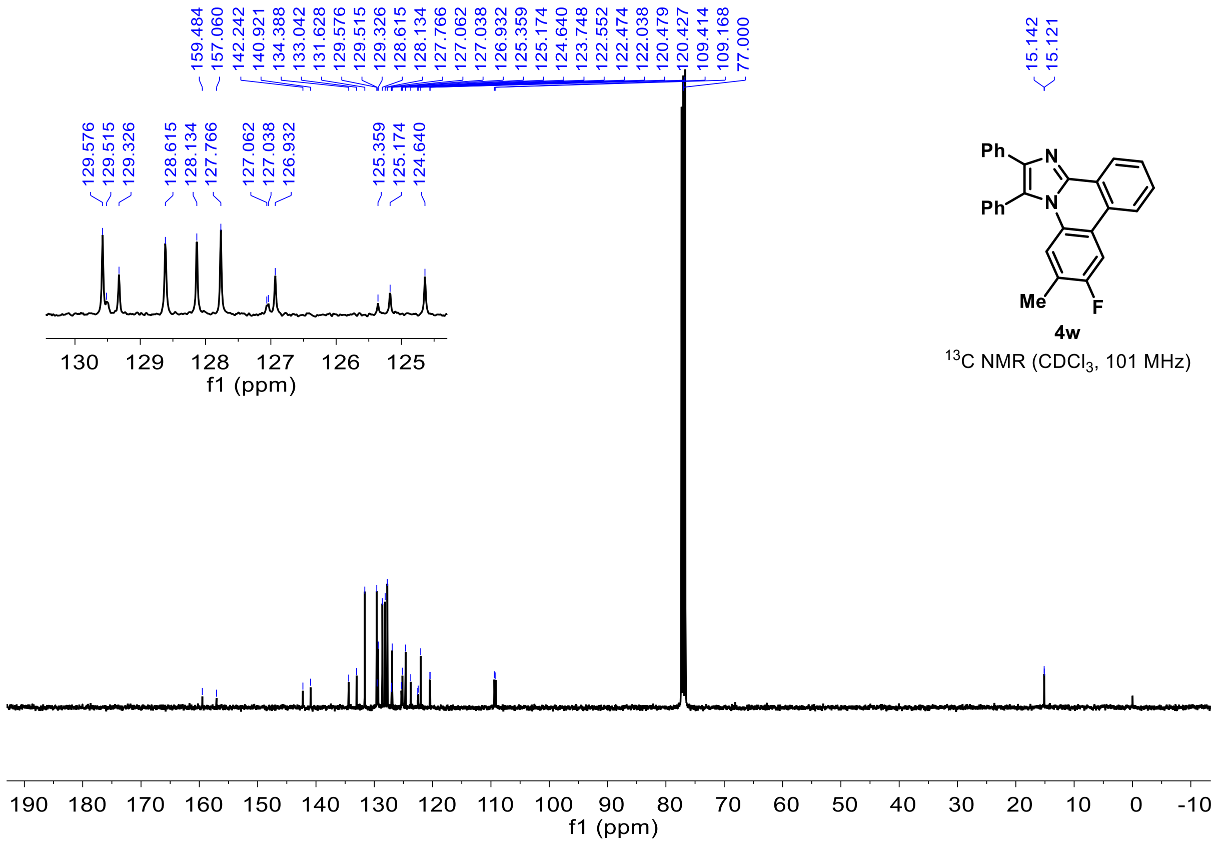

4w

^{13}C NMR ($CDCl_3$, 101 MHz)

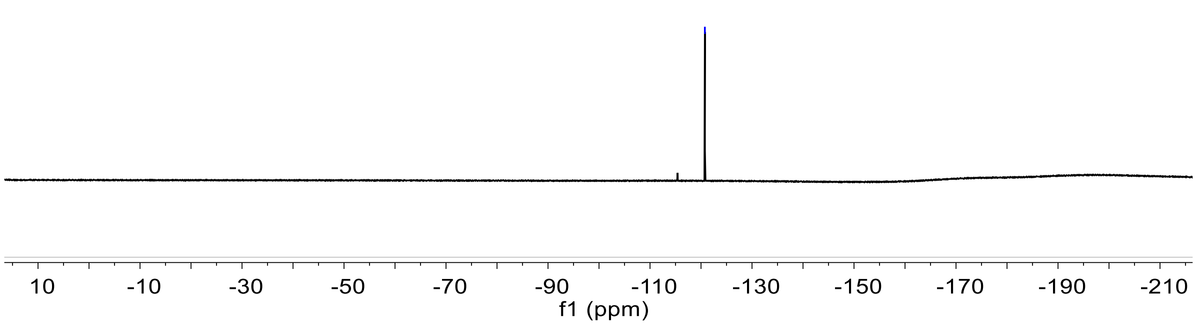

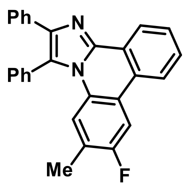

4w

^{19}F NMR ($CDCl_3$, 376 MHz)

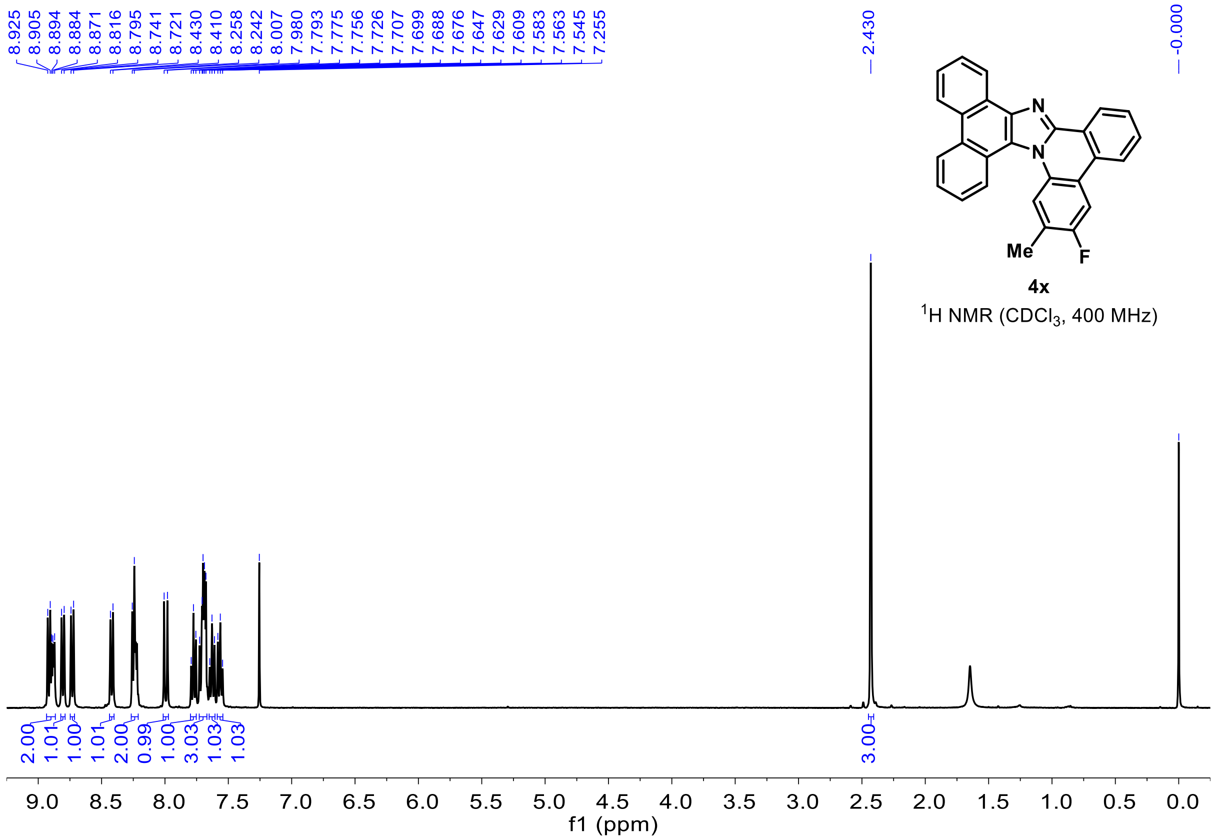

8.925 8.905 8.894 8.884 8.871 8.816 8.795 8.741 8.721 8.430 8.410 8.258 8.242 8.007 7.980 7.793 7.775 7.756 7.726 7.707 7.699 7.688 7.676 7.647 7.629 7.609 7.583 7.563 7.545 7.255
2.430
-0.000
N
N
Me
F
4x
^{1}H NMR (CDCl$_3$, 400 MHz)
2.00 1.01 1.00 1.01 2.00 0.99 1.00 3.03 1.03 1.03
3.00
9.0 8.5 8.0 7.5 7.0 6.5 6.0 5.5 5.0 4.5 4.0 3.5 3.0 2.5 2.0 1.5 1.0 0.5 0.0
f1 (ppm)

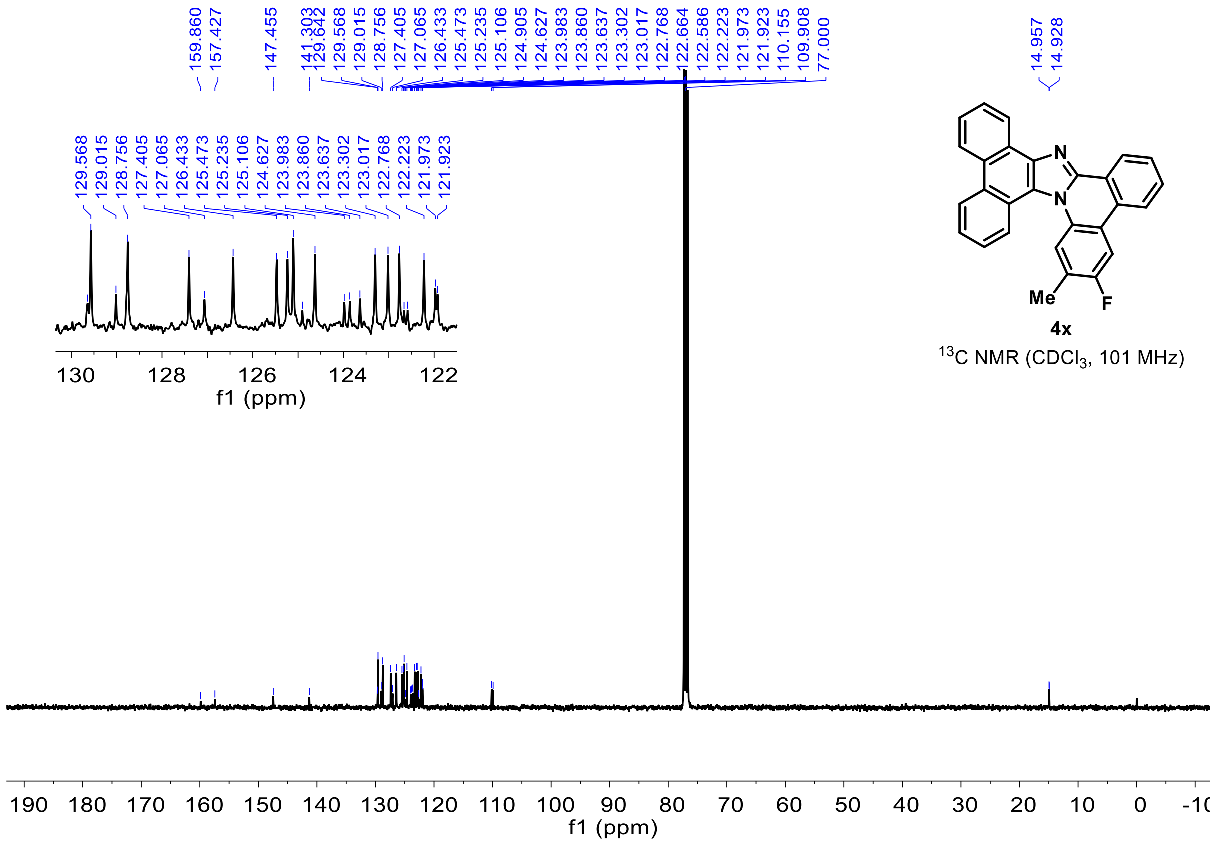

159.860 157.427
147.455
141.303 129.642 129.568 129.015 128.756 127.405 127.065 126.433 125.473 125.235 125.106 124.905 124.627 123.983 123.860 123.637 123.302 123.017 122.768 122.664 122.586 122.223 121.973 121.923 110.155 109.908 77.000
14.957 14.928
129.568 129.015 128.756 127.405 127.065 126.433 125.473 125.235 125.106 124.627 123.983 123.860 123.637 123.302 123.017 122.768 122.223 121.973 121.923
130 128 126 124 122
f1 (ppm)
N
N
Me
F
4x
^{13}C NMR (CDCl$_3$, 101 MHz)
190 180 170 160 150 140 130 120 110 100 90 80 70 60 50 40 30 20 10 0 -10
f1 (ppm)

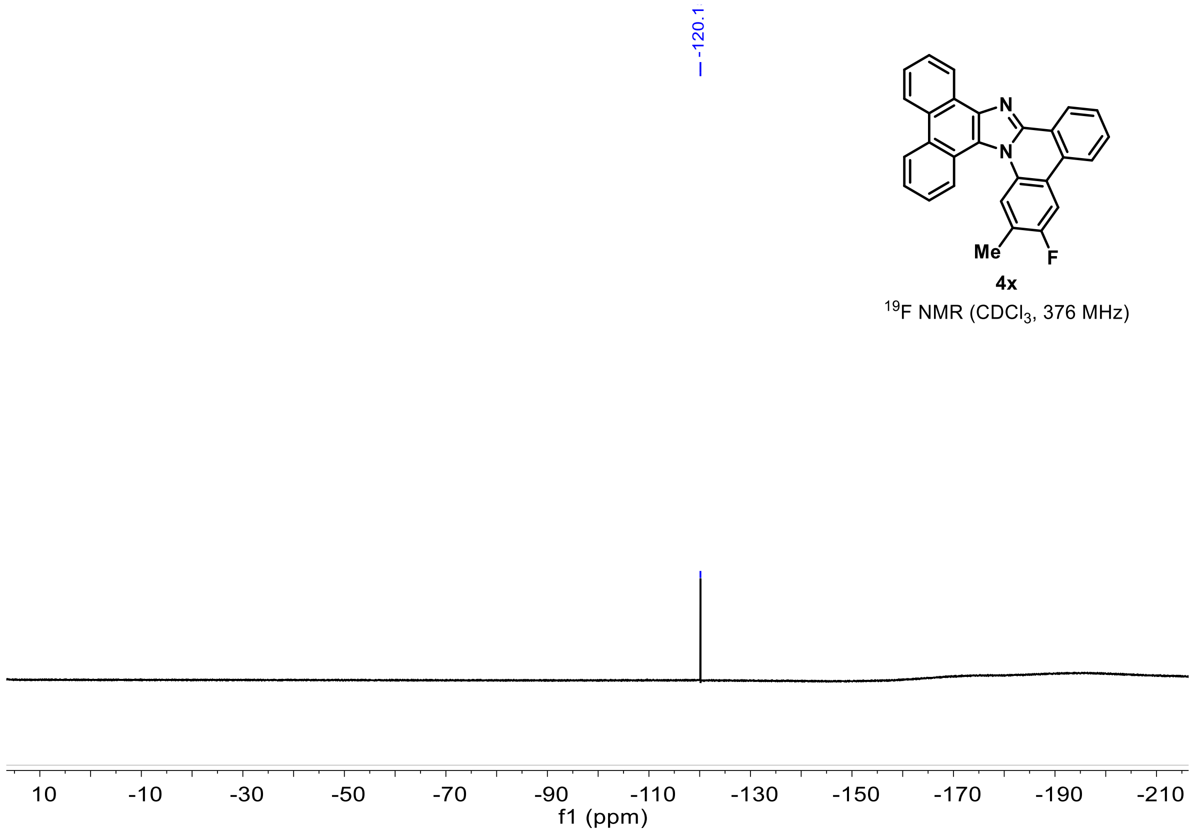
-120.1
N
N
Me
F
4x
19F NMR (CDCl3, 376 MHz)
10 -10 -30 -50 -70 -90 -110 -130 -150 -170 -190 -210
f1 (ppm)

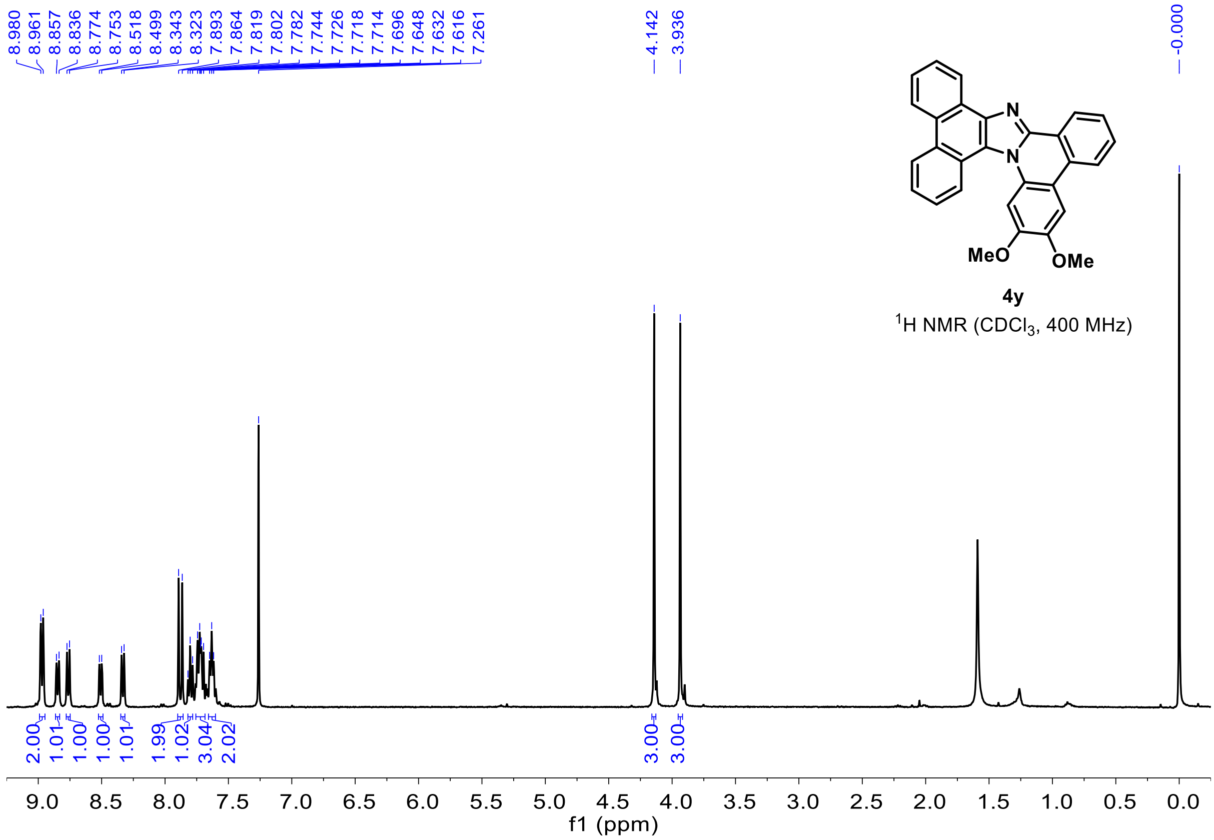
8.980
8.961
8.857
8.836
8.774
8.753
8.518
8.499
8.343
8.323
7.893
7.864
7.819
7.802
7.782
7.744
7.726
7.718
7.714
7.696
7.648
7.632
7.616
7.261
4.142
3.936
-0.000
N
N
MeO
OMe
4y
^{1}H NMR (CDCl$_3$, 400 MHz)
2.00
1.01
1.00
1.00
1.01
1.99
1.02
3.04
2.02
3.00
3.00
9.0 8.5 8.0 7.5 7.0 6.5 6.0 5.5 5.0 4.5 4.0 3.5 3.0 2.5 2.0 1.5 1.0 0.5 0.0
f1 (ppm)

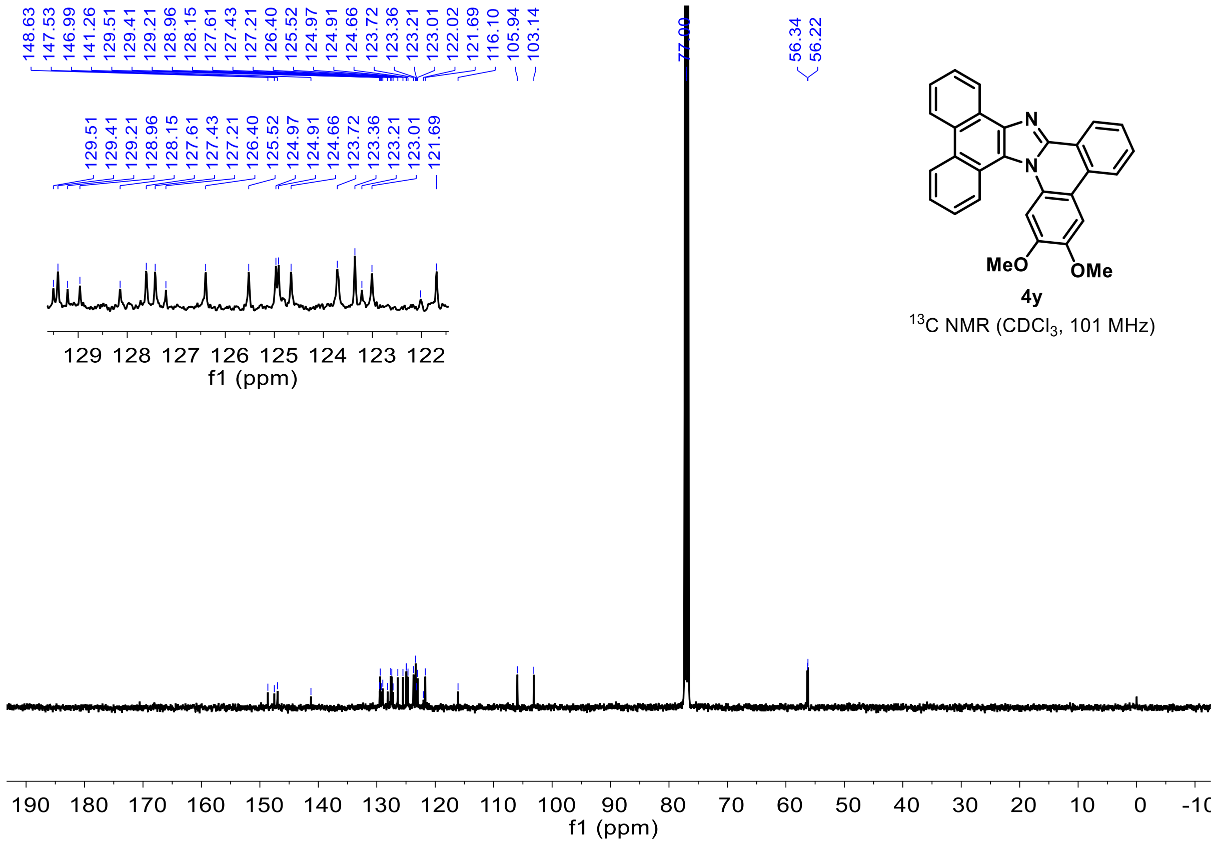
148.63
147.53
146.99
141.26
129.51
129.41
129.21
128.96
128.15
127.61
127.43
127.21
126.40
125.52
124.97
124.91
124.66
123.72
123.36
123.21
123.01
122.02
121.69
116.10
105.94
103.14
77.00
56.34
56.22
129.51
129.41
129.21
128.96
128.15
127.61
127.43
127.21
126.40
125.52
124.97
124.91
124.66
123.72
123.36
123.21
123.01
121.69
129 128 127 126 125 124 123 122
f1 (ppm)
N
N
MeO
OMe
4y
^{13}C NMR (CDCl$_3$, 101 MHz)
190 180 170 160 150 140 130 120 110 100 90 80 70 60 50 40 30 20 10 0 -10
f1 (ppm)

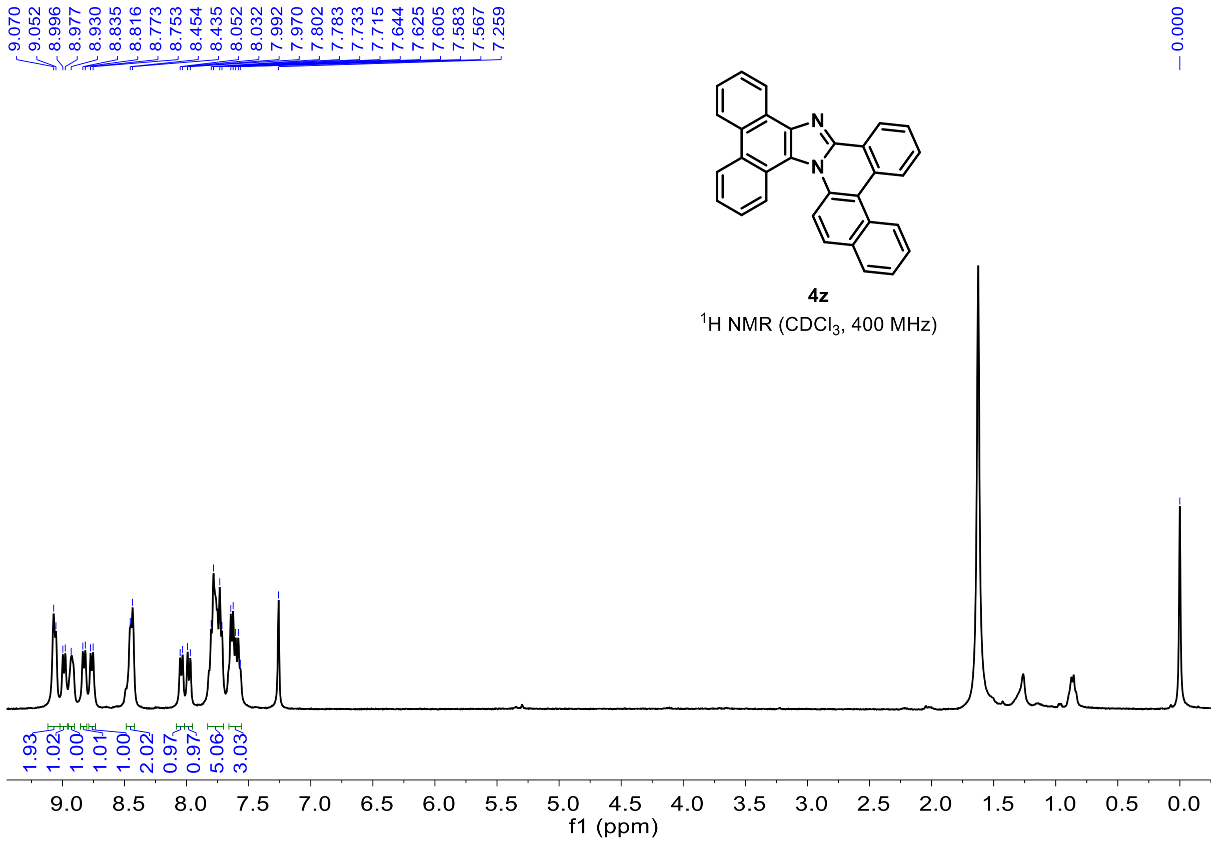
9.070
9.052
8.996
8.977
8.930
8.835
8.816
8.773
8.753
8.454
8.435
8.052
8.032
7.992
7.970
7.802
7.783
7.733
7.715
7.644
7.625
7.605
7.583
7.567
7.259
0.000
N
N
4z
1H NMR (CDCl3, 400 MHz)
1.93
1.02
1.00
1.01
1.00
2.02
0.97
0.97
5.06
3.03
9.0 8.5 8.0 7.5 7.0 6.5 6.0 5.5 5.0 4.5 4.0 3.5 3.0 2.5 2.0 1.5 1.0 0.5 0.0
f1 (ppm)

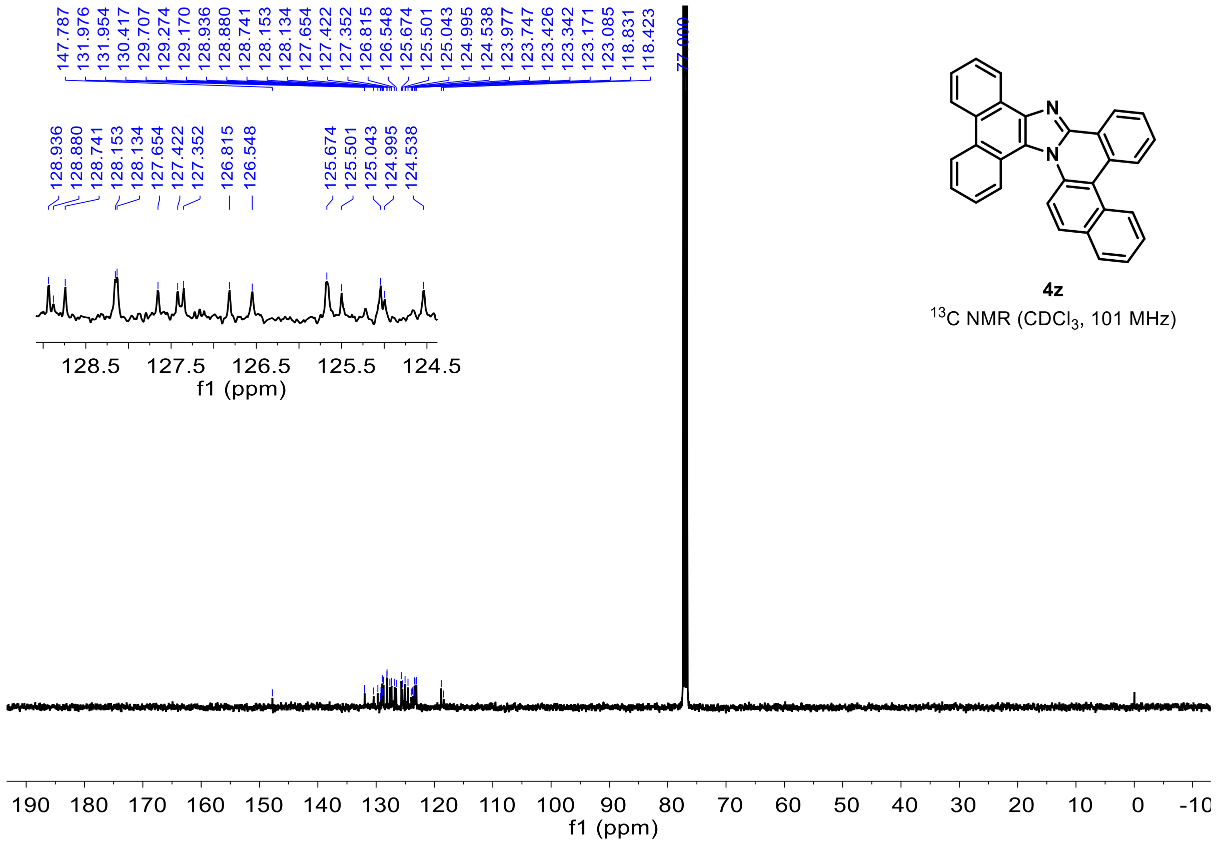
147.787
131.976
131.954
130.417
129.707
129.274
129.170
128.936
128.880
128.741
128.153
128.134
127.654
127.422
127.352
126.815
126.548
125.674
125.501
125.043
124.995
124.538
123.977
123.747
123.426
123.342
123.171
123.085
118.831
118.423
77.000
128.936
128.880
128.741
128.153
128.134
127.654
127.422
127.352
126.815
126.548
125.674
125.501
125.043
124.995
124.538
128.5 127.5 126.5 125.5 124.5
f1 (ppm)
N
N
4z
13C NMR (CDCl3, 101 MHz)
190 180 170 160 150 140 130 120 110 100 90 80 70 60 50 40 30 20 10 0 -10
f1 (ppm)

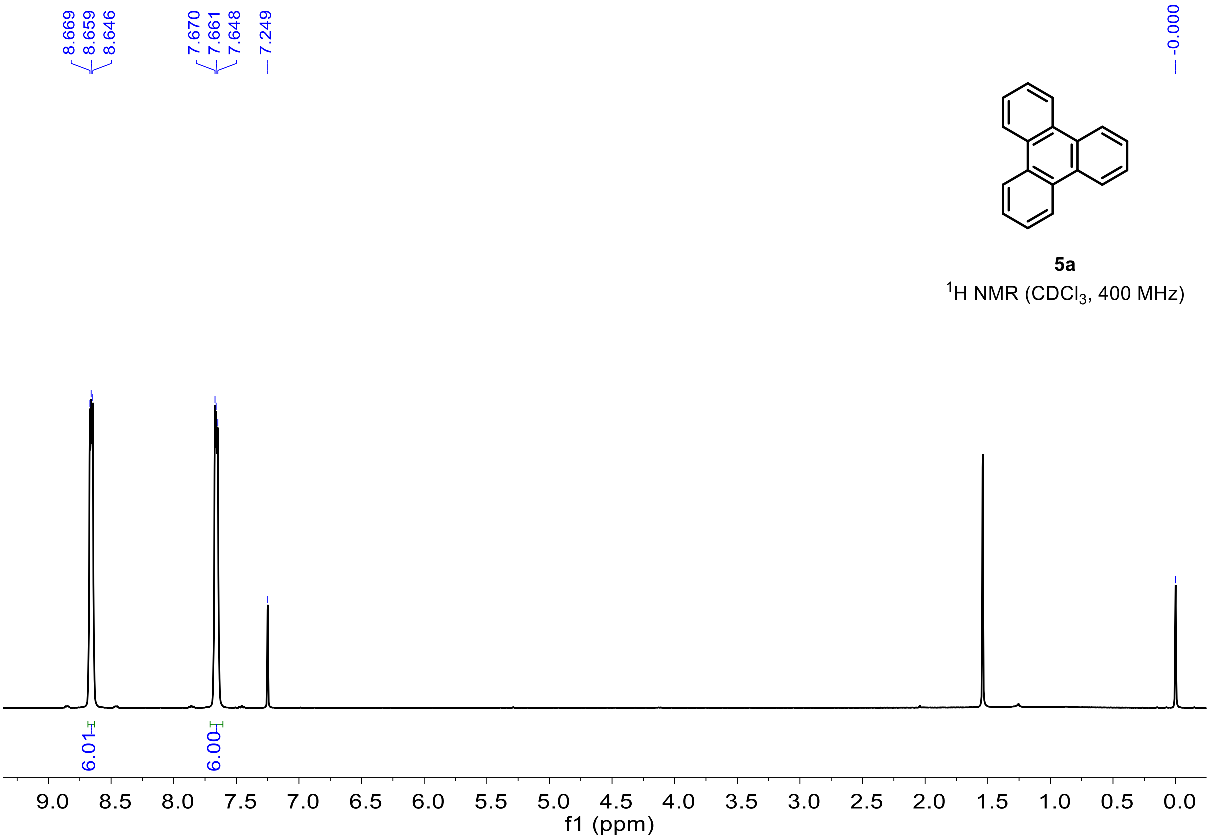

5a

^{1}H NMR ($CDCl_3$, 400 MHz)

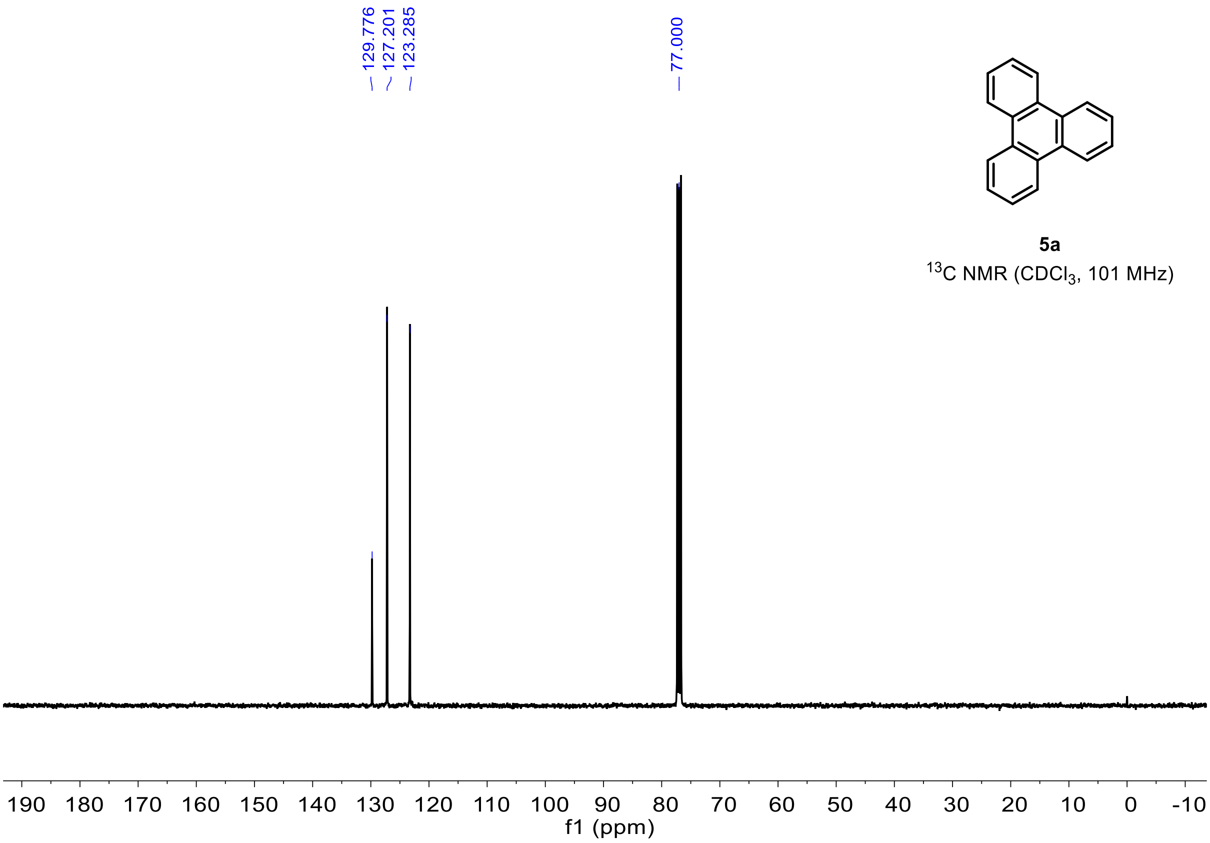

5a

^{13}C NMR ($CDCl_3$, 101 MHz)

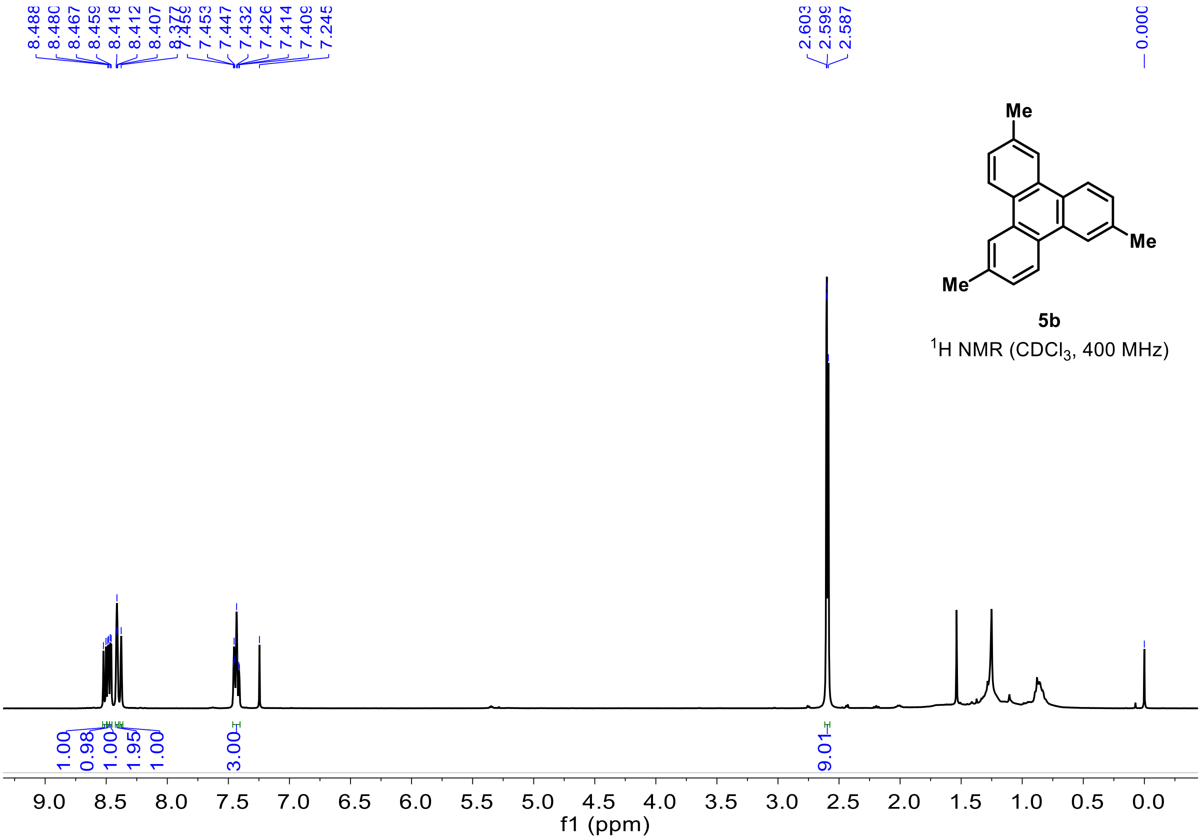
8.488
8.480
8.467
8.459
8.418
8.412
8.407
8.377
7.459
7.453
7.447
7.432
7.426
7.414
7.409
7.245
2.603
2.599
2.587
0.000
Me
Me
Me
5b
^{1}H NMR ($CDCl_3$, 400 MHz)
1.00
0.98
1.00
1.95
1.00
3.00
9.01
9.0 8.5 8.0 7.5 7.0 6.5 6.0 5.5 5.0 4.5 4.0 3.5 3.0 2.5 2.0 1.5 1.0 0.5 0.0
f1 (ppm)

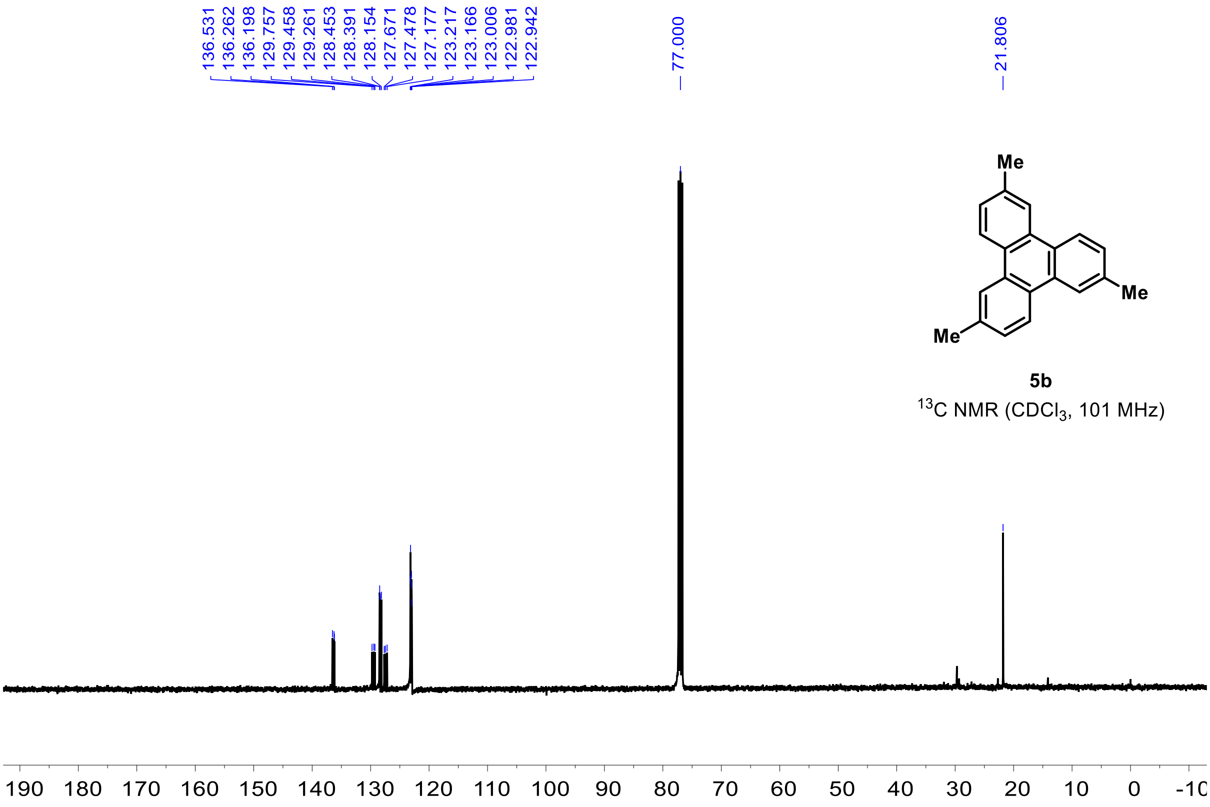
136.531
136.262
136.198
129.757
129.458
129.261
128.453
128.391
128.154
127.671
127.478
127.177
123.217
123.166
123.006
122.981
122.942
77.000
21.806
Me
Me
Me
5b
^{13}C NMR ($CDCl_3$, 101 MHz)
190 180 170 160 150 140 130 120 110 100 90 80 70 60 50 40 30 20 10 0 -10

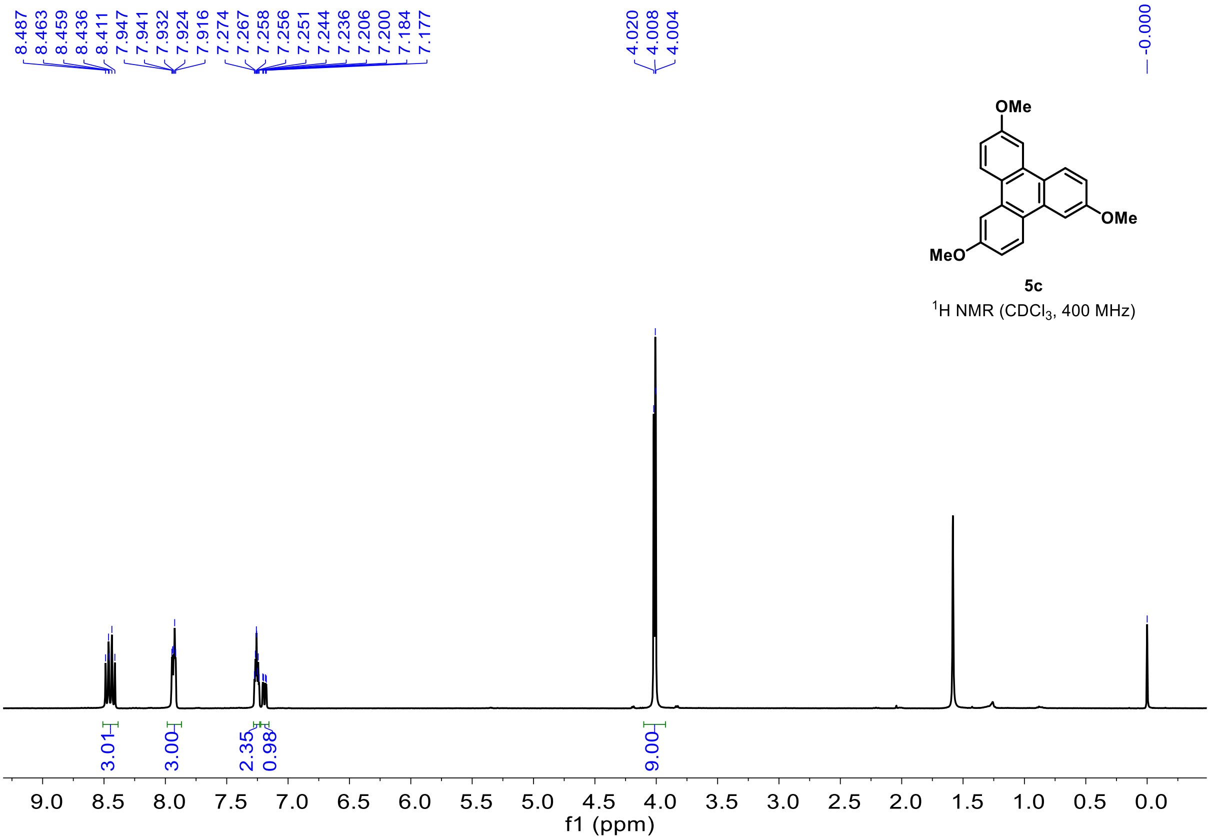
8.487
8.463
8.459
8.436
8.411
7.947
7.941
7.932
7.924
7.916
7.274
7.267
7.258
7.256
7.251
7.244
7.236
7.206
7.200
7.184
7.177
4.020
4.008
4.004
-0.000
OMe
OMe
MeO
5c
^{1}H NMR ($CDCl_3$, 400 MHz)
3.01
3.00
2.35
0.98
9.00
9.0 8.5 8.0 7.5 7.0 6.5 6.0 5.5 5.0 4.5 4.0 3.5 3.0 2.5 2.0 1.5 1.0 0.5 0.0
f1 (ppm)

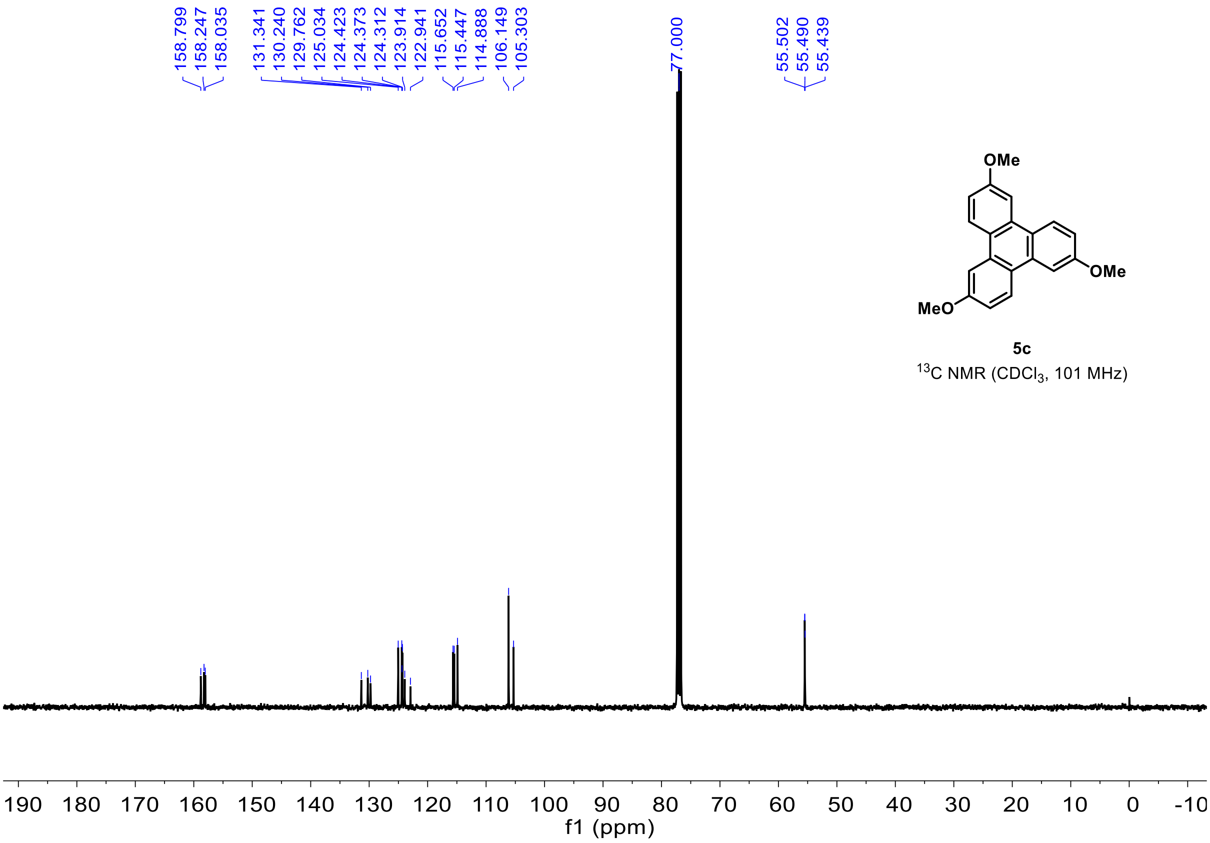
158.799
158.247
158.035
131.341
130.240
129.762
125.034
124.423
124.373
124.312
123.914
122.941
115.652
115.447
114.888
106.149
105.303
77.000
55.502
55.490
55.439
OMe
OMe
MeO
5c
^{13}C NMR ($CDCl_3$, 101 MHz)
190 180 170 160 150 140 130 120 110 100 90 80 70 60 50 40 30 20 10 0 -10
f1 (ppm)

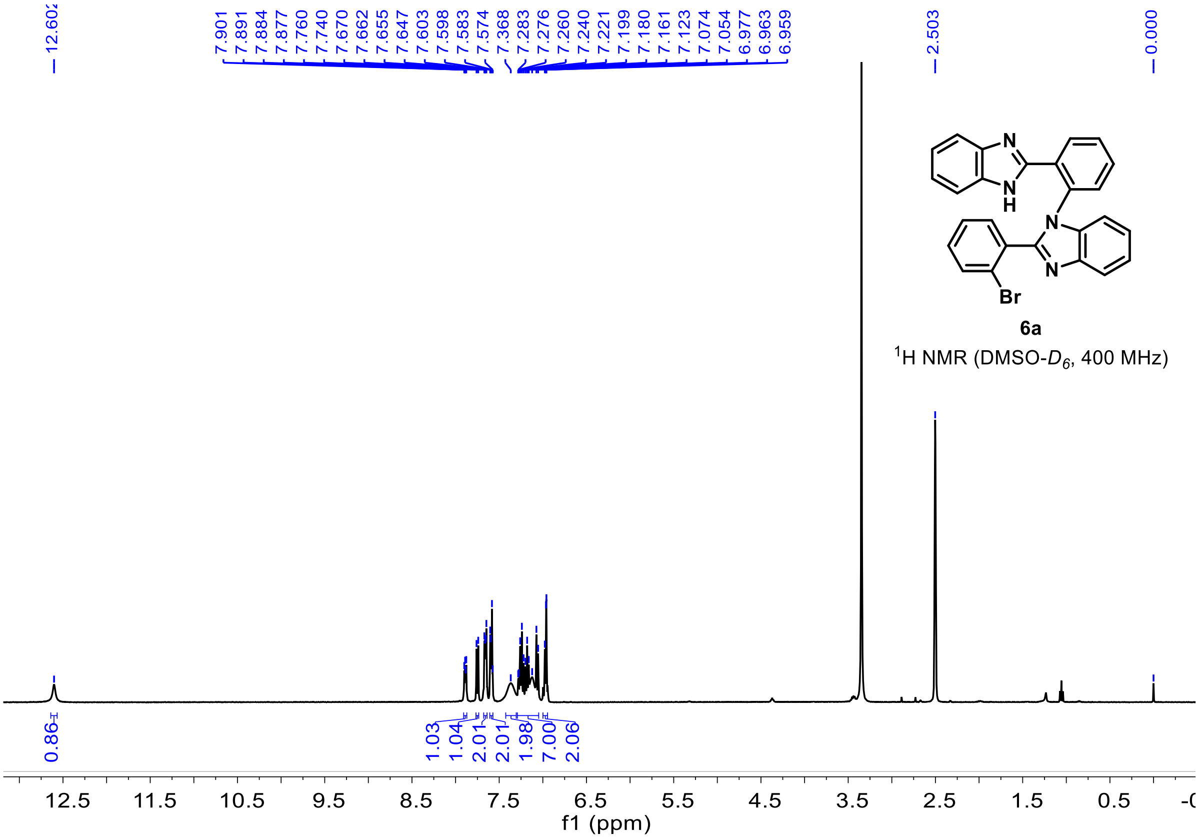
6a
^{1}H NMR (DMSO-D_6, 400 MHz)
f1 (ppm)

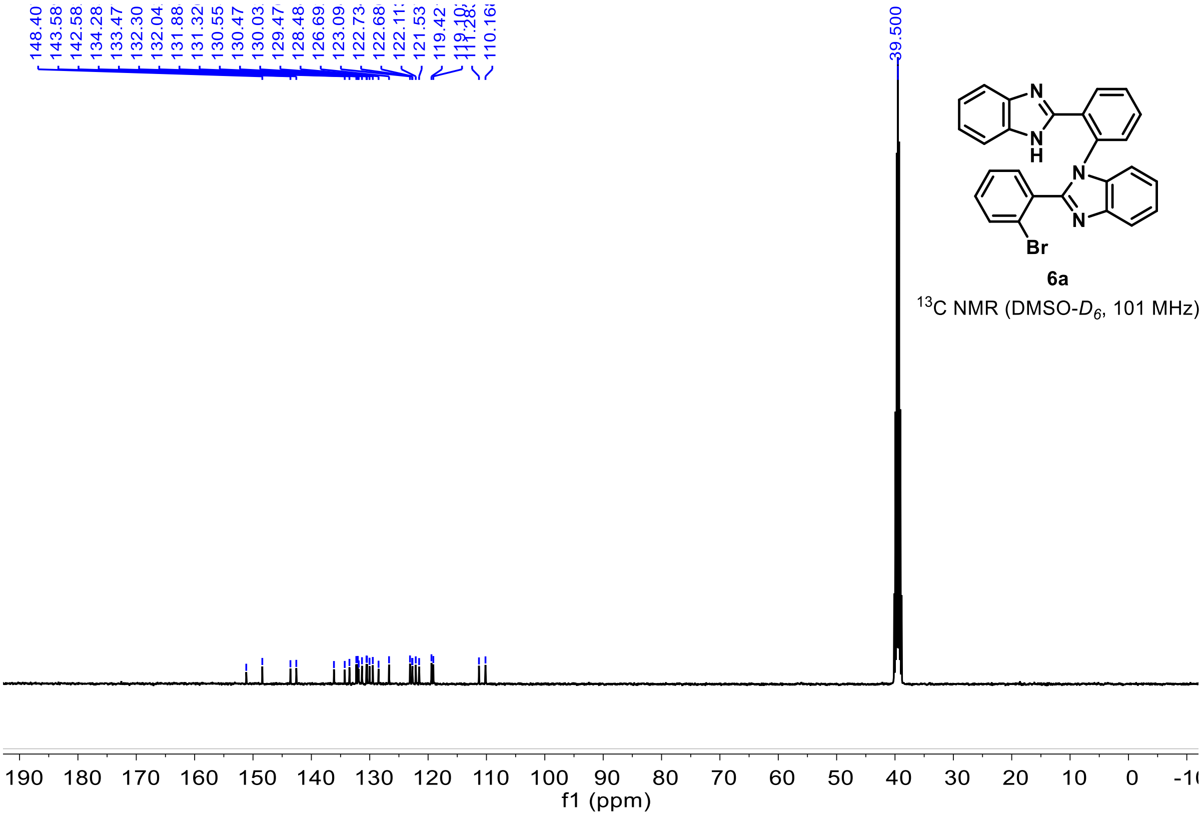
6a
^{13}C NMR (DMSO-D_6, 101 MHz)
f1 (ppm)

VI. References

(1) Jiang, Y.-q.; Jia, S.-h.; Li, X.-y.; Sun, Y.-m.; Li, W.; Zhang, W.-w.; Xu, G.-q. An Efficient $NaHSO_3$-Promoted Protocol for Chemoselective Synthesis of 2-Substituted Benzimidazoles in Water. *Chem. Pap.* **2018**, *72*, 1265–1276.

(2) Gang, M.-Y.; Liu, J.-Q.; Wang, X.-S. CuI-catalyzed Sonogashira Reaction for the Efficient Synthesis of 1H-imidazo[2,1-a]isoquinoline Derivatives. *Tetrahedron* **2017**, *73*, 4698–4705.

(3) Zhao, G.; Chen, C.; Yue, Y.; Yu, Y.; Peng, J. Palladium(II)-Catalyzed Sequential C–H Arylation/Aerobic Oxidative C–H Amination: One-Pot Synthesis of Benzimidazole-Fused Phenanthridines from 2-Arylbenzimidazoles and Aryl Halides. *J. Org. Chem.* **2015**, *80*, 2827–2834.

(4) Chen, C.; Shang, G.; Zhou, J.; Yu, Y.; Li, B.; Peng, J. Modular Synthesis of Benzimidazole-Fused Phenanthridines from 2-Arylbenzimidazoles and *o*-Dibromoarenes by a Palladium-Catalyzed Cascade Process. *Org. Lett.* **2014**, *16*, 1872–1875.

(5) Yan, L.; Zhao, D.; Lan, J.; Cheng, Y.; Guo, Q.; Li, X.; Wu, N.; You, J. Palladium-Catalyzed Tandem N–H/C–H Arylation: Regioselective Synthesis of N-Heterocycle-Fused Phenanthridines as Versatile Blue-Emitting Luminophores. *Org. Biomol. Chem.* **2013**, *11*, 7966–7977.

(6) Shi, J.; Li, J.; Zhao, W.; Cui, M.; Ni, W.; Li, L.; Liu, W.; Xu, K.; Zhang, S. Regioselective Intramolecular sp^2 C–H Amination: Direct vs. Mediated Electrooxidation. *Org. Chem. Front.* **2021**, *8*, 1581–1586.

(7) Saini, H. K.; Dhiman, S.; Pericherla, K.; Kumar, A. Synthesis of Imidazo[1,2-f]phenanthridines through Palladium-Catalyzed Intramolecular C–C Bond Formation. *Synthesis* **2015**, *47*, 3727–3732.

(8) Cant, A. A.; Roberts, L.; Greaney, M. F. Generation of Benzyne from Benzoic Acid Using C–H Activation. *Chem. Commun.* **2010**, *46*, 8671–8673.

(9) Kim, H. S.; Gowrisankar, S.; Kim, E. S.; Kim, J. N. A Brand-New Pd-Mediated Generation of Benzyne and Its [2 + 2 + 2] Cycloaddition: δ-Carbon Elimination and Concomitant Decarboxylation. *Tetrahedron Lett.* **2008**, *49*, 6569–6572.
